# Supplementary material for: Urban heat mitigation by green and blue infrastructure: Drivers, effectiveness, and future needs
Source: Innovation (Camb). 2024 Feb 7;5(2):100588. doi: 10.1016/j.xinn.2024.100588 (PMC10909648; doi:10.1016/j.xinn.2024.100588)
Supplement: Document S1. Figures S1–S7, Tables S1–S9, Sections S1, and S2 [file mmc1.pdf]

The Innovation, Volume *5*

Supplemental Information

Urban heat mitigation by green and blue infrastructure: Drivers, effectiveness, and future needs

Prashant Kumar, Sisay E. Debele, Soheila Khalili, Christos H. Halios, Jeetendra Sahani, Nasrin Aghamohammadi, Maria de Fatima Andrade, Maria Athanassiadou, Kamaldeep Bhui, Nerea Calvillo, Shi-Jie Cao, Frederic Coulon, Jill L. Edmondson, David Fletcher, Edmilson Dias de Freitas, Hai Guo, Matthew C. Hort, Madhusudan Katti, Thomas Rodding Kjeldsen, Steffen Lehmann, Giuliano Maselli Locosselli, Shelagh K. Malham, Lidia Morawska, Rajan Parajuli, Christopher D.F. Rogers, Runming Yao, Fang Wang, Jannis Wenk, and Laurence Jones

Supplementary Information (SI)

for

Urban heat mitigation by green and blue infrastructure: a review of drivers, effectiveness, and future needs

Prashant Kumar[1,2,10,*], Sisay Debele[1], Soheila Khalili[1], Christos H. Halios[3], Jeetendra Sahani[1], Nasrin Aghamohammadi[4,5], Maria de Fatima Andrade[6], Maria Athanassiadou[7], Kamaldeep Bhui[8] , Nerea Calvillo[9], Shi-Jie Cao[1,10], Frederic Coulon[11], Jill L. Edmondson[12], David Fletcher[13], Edmilson Dias de Freitas[6], Hai Guo[14], Matthew C Hort[7], Madhusudan Katti[15], Thomas Rodding Kjeldsen[16], Steffen Lehmann[17], Giuliano Maselli Locosselli[18], Shelagh K Malham[19], Lidia Morawska[1,20], Rajan Parajuli[21], , Christopher DF Rogers[22], Runming Yao[3,23], Fang Wang[24,25], Jannis Wenk[16], Laurence Jones[13,26]

[1]Global Centre for Clean Air Research (GCARE), School of Sustainability, Civil and Environmental Engineering, Faculty of Engineering and Physical Sciences, University of Surrey, Guildford GU2 7XH, United Kingdom

[2]Institute for Sustainability, University of Surrey, Guildford GU2 7XH, Surrey, United Kingdom

[3]School of Built Environment, University of Reading, Shinfield Rd, Whiteknights Rd, Reading RG6 6BU, United Kingdom

[4]School Design and the Built Environment, Curtin University Sustainability Policy Institute, Kent St, Bentley, 6102, Western Australia

[5]Harry Butler Institute, Murdoch University, Murdoch, 6150 Western Australia

[6]Atmospheric Sciences Department, Institute of Astronomy, Geophysics and Atmospheric Sciences, University of Sao Paulo, 05508-090, Sao Paulo, Brazil

[7]Met Office, FitzRoy Road, Exeter, EX1 3PB, United Kingdom
[8]Department of Psychiatry and Nuffield Department of Primary Care Health Sciences, Wadham College, University of Oxford, Oxford, United Kingdom
[9]Centre for Interdisciplinary Methodologies, University of Warwick, United Kingdom
[10]School of Architecture, Southeast University, 2 Sipailou, Nanjing, 210096, China
[11]Cranfield University, School of Water, Environment and Energy, Cranfield, MK43 0AL, United Kingdom
[12]Plants, Photosynthesis, Soil Cluster, School of Biosciences, University of Sheffield, Sheffield S10 2TN, United Kingdom
[13]UK Centre for Ecology & Hydrology, Environment Centre Wales, Deiniol Road, Bangor LL57 2UW, United Kingdom
[14]Air Quality Studies, Department of Civil and Environmental Engineering, The Hong Kong Polytechnic University, Hong Kong, China
[15]Department of Forestry and Environmental Resources, Faculty Excellence Program for Leadership in Public Science, North Carolina State University, Chancellor', Raleigh, NC, 27695, USA
[16]Departments of Architecture & Civil Engineering, and Chemical Engineering, University of Bath, Bath BA2 7AY, United Kingdom
[17]School of Architecture, University of Nevada, Las Vegas, NV 89154, USA
[18]Department of Tropical Ecosystems Functioning, Center of Nuclear Energy in Agriculture, University of São Paulo, Piracicaba, 13416-000, Sao Paulo, Brazil
[19]School of Ocean Sciences, Bangor University, Menai Bridge, Anglesey, LL59 5 AB, United Kingdom

[20]*International Laboratory for Air Quality and Health, Science and Engineering Faculty, Queensland University of Science and Technology, Queensland, Australia*

[21]*Department of Forestry and Environmental Resources, North Carolina State University, Raleigh NC 27695, USA*

[22]*Department of Civil Engineering, School of Engineering, University of Birmingham, Edgbaston, Birmingham B15 2TT, United Kingdom*

[23]*Joint International Research Laboratory of Green Buildings and Built Environments, School of the Civil Engineering, Chongqing University, China*

[24]*State Key Laboratory of Soil and Sustainable Agriculture, Institute of Soil Science, Chinese Academy of Sciences, Nanjing 210008, China*

[25]*University of Chinese Academy of Sciences, Beijing 100049, China*

[26]*Liverpool Hope University, Department of Geography and Environmental Science, Hope Park, Liverpool L16 9JD, United Kingdom*

This document includes:

Sections S1-S2

Figures S1-S7.

Tables S1-S9

S1 Methodology

We screened studies based on the following criteria: (a) addressing urban heat mitigation using one or more GBGI types, (b) clear identification of at least one GBGI sub-category under investigation, (c) a clear link between the primary GBGI category and heat mitigation performance, including temperature reduction and associated co-benefits, and (d) accessibility of full-text articles from the databases for further review and data extraction.

After removing duplicates, 25,974 publications that didn't meet these criteria were eliminated, leaving 1,512 publications for further screening (Figure 2b). We retrieved and assessed the full text of each paper for eligibility (Figure 2c). Articles not meeting inclusion criteria, such as those mainly focusing on heat causes and impacts, methodological aspects of heat mitigation without a direct GBGI connection, or discussions limited to general green and blue spaces without specific GBGI details and cooling effectiveness, were excluded. After this additional screening, 1,250 more papers were discarded, resulting in 262 publications for final review. Out of these, 60 more publications were excluded due to insufficient performance reporting or a failure to mention the GBGI used. In the end, 202 publications (1.8% of the originally identified 27,486 publications) were chosen for meta-analysis and further consideration in this review (Figure 2d, e). First we cataloged data from the selected studies, extracting information from 202 of them, including (1) the study's location (site, city, country, and region), (2) the specific type of GBGI, (3) the nature of the study (monitoring, modeling, remote sensing, or a combination), (4) whether single or multiple GBGIs were studied, (5) qualitative or quantitative data on co-benefits, air temperature, land surface temperature (LST) reduction (in °C), and day or night air temperature reduction (in °C), (6) any identified knowledge gaps, and (7) key findings. We developed a data form to capture this essential information from the selected studies (Table S1). This information was used to address key objectives, including when and where previous articles were published, the types of GBGIs used for urban heat mitigation, and the nature of co-benefits and maximum temperature reductions reported. Following this, we analysed and synthesised the data from selected studies to address the specific review objectives. The information on the benefits of various GBGI subcategories was examined using descriptive statistics with R-project software. To create an evaluation framework for GBGI types and their services in addressing

heat-related challenges and associated co-benefits, we used a min-max normalization approach, scaling the data from 1 (none) to 6 (very high) (see Table 2). This standardized method was then applied to the relevant publications within each GBGI category. Out of the 202 publications, 64.7% primarily focused on heat mitigation as their main ecosystem service, while the remaining 35.3% discussed co-benefits alongside direct heat mitigation benefits.

The review proceeds with a summary of how GBGI mitigates heat stress, followed by mapping study origins, exploring GBGI interventions, and presenting evidence of their cooling advantages and co/dis-benefits. It also provides a conceptual framework for GBGI implementation and addresses existing knowledge gaps. The review concludes with major findings and recommendations for effective GBGI implementation to mitigate urban heat.
Five additional co-benefits are identified, including enhanced recreational opportunities and improvements in air/water quality.

S1.1 GBGI classification, scope, and outline

The conceptualisation and classification of GBGI types can differ due to varying interpretations from country to country, contingent on research contexts and the primary objective of studies. Sustainability objectives are selectively applied within different GBGI development frameworks, leading to a plethora of definitions and interpretations.[1] These variations depend on whether GBGI is applied to ecological resources[2] or includes natural green spaces, or is confined to highly altered landscapes intended for public benefit.[3] Consequently, it becomes challenging to specifically distinguish between green and grey infrastructure, such as cycle paths passing through green areas that provide additional recreational benefits. Often, scientists categorise non-ecological resources as GBGI, for example, permeable pavements, rainwater barrels, and rain gardens. For this review, we

adopt the GBGI classification presented by Jones et al.[4] and expand it further by introducing two new categories, mixed (blue-green) and backyard irrigation,[5-7] giving a total of 51 GBGI types. This classification uses a green-blue-grey continuum to cover natural green or blue, engineered green, blue, or hybrid (green-blue-grey) combinations. The typology was designed to flexibly incorporate all GBGI types within a typical urban environment. Within the scope of this review, we examined 51 GBGI types grouped under 10 broad categories: gardens, parks, amenity areas, linear features/routes, constructed GI on infrastructure, hybrid GI (for water), water bodies, other non-sealed urban areas, other public spaces, and mixed – (green-blue).

The task of examining and integrating the diverse benefits of GBGIs was challenging due to their complex interactions and discipline-specific applications. For instance, public health is directly or indirectly tied to almost all evaluated benefits, encompassing psychological, social, and economic aspects.[8] Therefore, this study focuses on the direct GBGI cooling benefits (heat mitigation), whilst indirect benefits such as management of other natural hazards (floods, droughts), the creation of new infrastructure (such as interconnected green infrastructure corridors to support active travel; Rogers and Hunt[9]) or associated social costs avoided by using specific GBGI types are considered as a secondary objective. In the course of this review, five more co-benefits were identified: (1) enhanced recreational opportunities, (2) ambient noise reduction, (3) flood and drought risk mitigation, (4) improvements in air/water quality, and (5) biodiversity (Section 3.3.2). Detailed GBGI design and implementation principles, along with global GBGI challenges, have been covered in earlier reviews (Table 1) and therefore were beyond the scope of this paper.

The review commences with a concise summary of how GBGI mitigates heat stress (Section 3), followed by a spatial and temporal mapping of study origins, an exploration of various GBGI interventions, and a presentation of quantitative evidence supporting the direct cooling advantages and other co/dis-benefits (Section 4). Section 5 provides a nine-stage conceptual framework for GBGI implementation for heat mitigation based on the qualitative analysis of the reviewed literature and discusses practical recommendations for the design, implementation, monitoring, evaluation, and upscaling of GBGI to mitigate heat risks. Section 6 highlights the existing knowledge gaps. The review culminates with major conclusions and lays out a series of recommendations for the effective implementation of GBGI to mitigate urban heat (Section 7).

S1.2 Search and selection of relevant studies

The goal of this review was to offer an in-depth assessment review and analysis of GBGI's functions and benefits concerning urban cooling, as well as their potential co-benefits and drawbacks. This approach led to an expansion of the scope beyond that of previous reviews covered, enables us to uncover overlooked geographical patterns and examine the temporal trends in the origin of studies, and knowledge voids in the existing literature. PRISMA methodology was adopted for this systematic review.[10] Figure 1 provides a flowchart depicting our search and evaluation methodology, including its resultant findings. Our literature search consisted of five stages:

(1) Development of search terms: To identify a comprehensive range of studies related to urban heat mitigation, relevant search terms were determined based on research gaps, objectives, and predetermined categories and subcategories within the GBGI framework. This approach ensures the inclusion of a diverse set of studies that are pertinent to the field of heat

mitigation. A range of relevant search terms based on keywords for urban heat mitigation was identified based on research gaps, objectives, and predetermined GBGI categories and subcategories to allow the identification of a wide range of studies relevant to heat mitigation. Search term combinations of GBGI type and heat are listed in Supplementary Information (SI) Table S1.

(2) Search and identification of relevant studies: A peer-reviewed literature search was conducted via Boolean search term combinations (Table S1) utilising Web of Science (WoS), as the most comprehensive database with the ability to handle complex keyword searches. Studies published between 2010 and 2023 were included. The chosen timeframe was specifically selected to effectively manage the substantial number of search results, preventing an overwhelming amount of hits. Moreover, this time frame enables a more comprehensive examination, particularly in relation to the GBGI, which gained increased recognition as "nature-based solution" after the year 2010. Cross-checks were performed using other databases such as Science Direct, Scopus, and Google Scholar to verify that no relevant studies were missed from the analysis. To ensure inclusivity, we used the same keywords as in WoS (Table S1) when searching on Google Scholar. We reviewed multiple pages of search results initially, but the relevance of studies decreased as we continued. Therefore, we concluded the search after examining the first 20 pages. Finally, we compared the results with papers from WoS and included any relevant publications that were missed in our analysis. After excluding articles not written in English, the search terms yielded a total of 27,486 publications, including review and research papers (Figure 2a).

(3) Selection of studies: The identified studies were screened against the following criteria: (a) addresses mitigation and/or adaptation to urban heat using one or several types of GBGI,

(b) distinctly identifies at least one GBGI sub-category under investigation, (c) the main GBGI category is clearly linked with heat mitigation performance, including actual or percentage temperature reduction, and any associated co-benefits, (d) the full texts were accessible from the databases for further review and data extractions. After removing duplicates, the remaining articles were reviewed and 25,974 publications that did not meet inclusion criteria were removed, leaving 1512 publications for further screening (Figure 2b). The full text of each paper was retrieved and assessed for eligibility (Figure 2c). Any articles not meeting the inclusion criteria, like those majorly focusing on causes and impacts of heat, methodological aspects of heat mitigation benefits without a direct tie to GBGI categories and temperature reductions or discussions confined to general green and blue spaces or green corridors without specific descriptions of GBGI types and cooling efficacy, were excluded. After this further screening, an additional 1250 papers were discarded, leaving 262 publications for final screening. Out of these, 60 more publications were excluded due to non-reporting of performances or failure to mention the utilised GBGI. Eventually, a set of 202 publications (1.8% of the originally identified 27,486 publications) was chosen for meta-analysis and subsequent deliberation in this review (Figure 2d, e).

(4) Cataloguing the data: Relevant data (e.g., location, type of GBGI, co-and dis-benefits, and knowledge gaps; Section 2.3) were extracted from the selected studies. The following data were extracted from the selected 202 studies: (1) the location of the GBGI study including the site, city, country, and region, (2) the specific type of GBGI, (3) the nature of the study, whether it was monitoring, modelling, remote sensing, or a combination of these, (4) either single or multiple GBGI, (5) qualitative or quantitative information on co-benefits, air temperature and land surface temperature (LST) reduction (in °C), and a day or night time air temperature reduction (in °C), (6) any identified knowledge gaps, and (7) key findings.

This process involved developing a data form to capture key information from the selected studies (Table S1). This extracted information was used to address the key objectives including when and where previous articles were published, the types of GBGI they utilised as mitigation measures for urban heat and the nature of co-benefits and maximum temperature reductions they reported.

(5) Collating, summarising, and reporting the results: The data obtained from the selected studies were analysed and synthesised in a way that addressed the specific questions raised as a part of the review objectives (Section 1). The extracted information on the (co-)benefits of various subcategories of GBGI was analysed using descriptive statistics using R-project software.[11] To develop an evaluation framework for GBGI types and the services offered to tackle heat-related challenges and their associated co-benefits, we adopted a min-max normalisation approach.[12] This approach, also referred to as feature scaling, included a linear transformation of the original data on a scale ranging from 1 (none) to 6 (very high) (see Table 2). Subsequently, this standardised methodology was applied to the pertinent publications within each GBGI category. Of the 202 publications, 64.7% discussed heat mitigation as their main ecosystem service. The remaining 35.3% discussed the co-benefits alongside the direct heat mitigation benefits (Section 3).

S2 Mechanisms of temperature and heat stress regulation by GBGI

S2.1 Mechanisms of temperature and heat stress regulation by green infrastructure

The mechanisms by which GI such as street trees, parks, green roofs, and green walls reduce heat are multifaceted and interconnected. Trees and plants help in the reduction of heat by providing shade and reducing the amount of direct sunlight reaching the ground, therefore lowering surface temperatures and mitigating the urban heat island (UHI) effect via creating a cooler microclimate.[13-15] Additionally, during evapotranspiration plants release

moisture which further cools the surrounding air by converting sensible heat into latent heat.[16] Parks can act as natural air conditioners through several mechanisms,[17-19] including the formation of microscale centripetal thermal system (park-breeze) that generate low-level advection currents which draw air from cooler green towards warmer urban areas.[20] Other GI elements such as green roofs, green walls, and roof gardens provide insulation, reduce heat absorption by buildings, and promote evaporative cooling (heat absorption, as water changes from liquid to a gas state in the air stream.[21-24] Vegetation also contributes to the dissipation of heat by acting as windbreaks, modifying airflow patterns, and facilitating natural ventilation.

S2.2 Mechanisms of temperature and heat stress regulation by blue infrastructure

Blue infrastructure (BI), in the form of water-based natural or constructed features including ponds and wetlands, actively mitigates heat effects by cooling the surrounding environment.[20] This is achieved through processes such as evapotranspiration, shading, the albedo effect, groundwater recharge, and temperature buffering.[25,26] BI can provide cooling during the day (acts as a heat sink by absorbing and storing heat from the surrounding environment) whereas it may lead to warming at night (re-releasing the heat due to water's higher heat capacity compared to the land surface).[27] Evaporation from water bodies also helps cool the air, creating a microclimate with lower temperatures and thereby helping to mitigate the UHI effect.[28] Larger urban water bodies can also generate cool breezes that further lower the ambient temperature and provide relief during hot weather through evaporative cooling.[20] Furthermore, surfaces of blue infrastructure are often highly reflective, especially under calm conditions, thereby increasing surface reflectivity which, in turn, contributes to the reduction of heat absorption,[29] thus helping to mitigate heat build-up and contributing to the cooling of the surrounding area. Some of the blue infrastructure such as

wetlands, ponds/lakes, swales, and rain gardens also act as natural sponges, storing water and releasing it during high air temperatures, thereby moderating temperatures in the vicinity by increasing water availability for evaporation through groundwater recharge.[30]

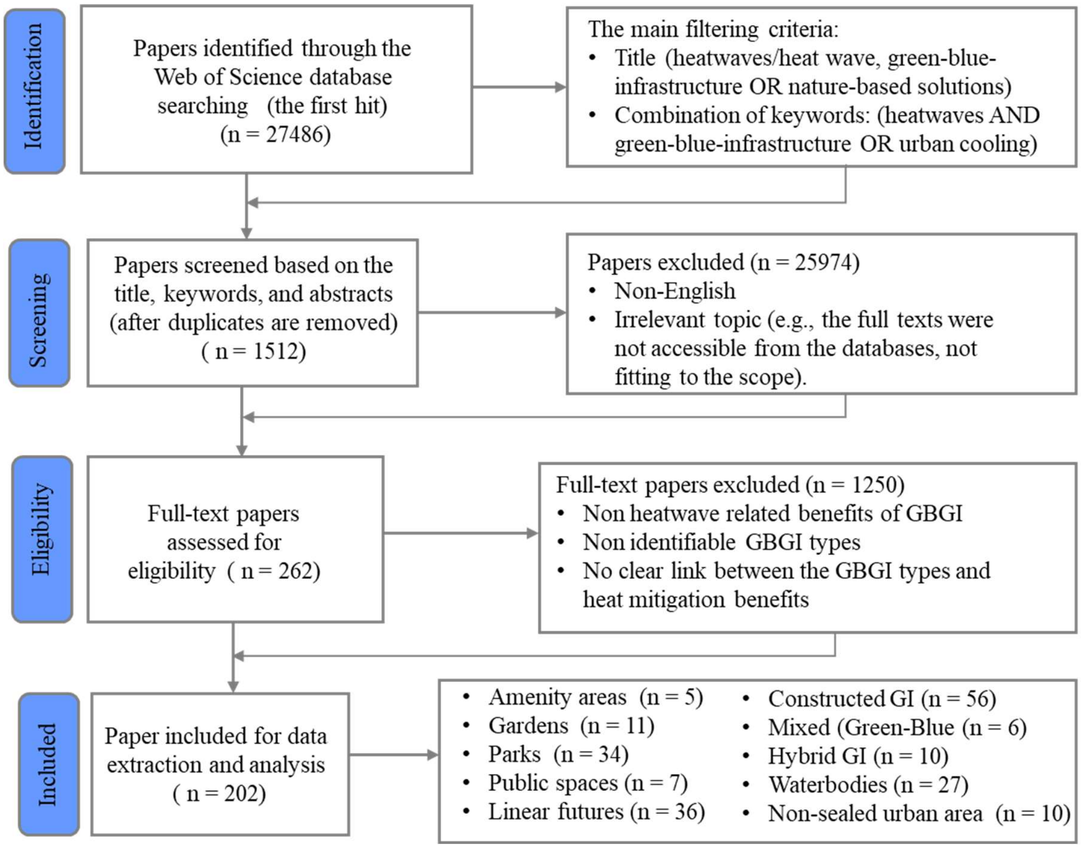

Figure S1. Flowchart of the inclusion and exclusion criteria (e.g. article identification, screening, eligibility check, and inclusion) process and the number of GBGI main categories included.

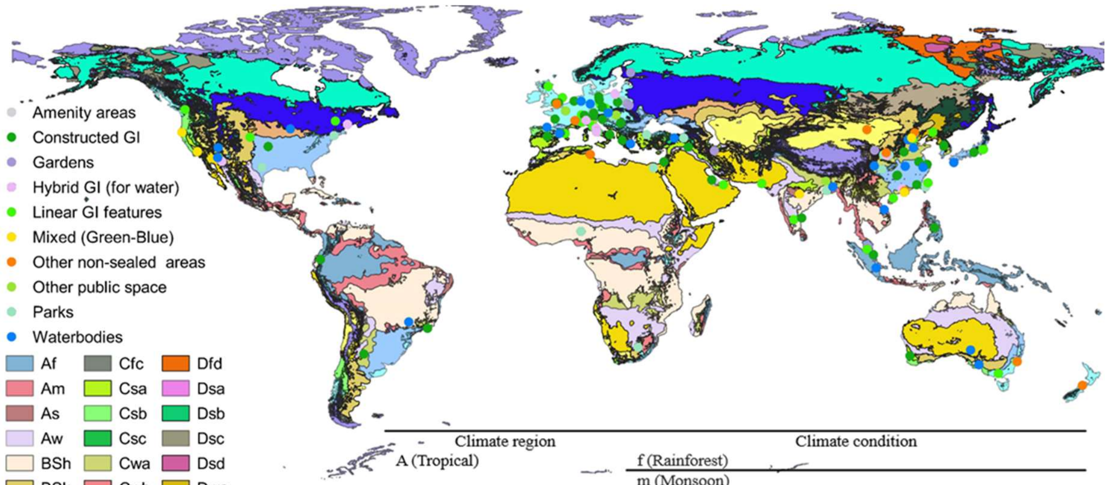

Climate region	Climate condition	
A (Tropical)	f (Rainforest)	
	m (Monsoon)<br>w (Savanna, dry winter)<br>s (Savanna, dry summer)	
B (Dry)	W (Arid Desert)<br>S (Semi-Arid or steppe)	h (Hot)<br>k (Cold)
C (Temperate)	w (Dry winter)<br>f (No dry season)<br>s (Dry summer)	a (Hot summer)<br>b (Warm summer)<br>c (Cold summer)
D (Continental)	w (Dry winter)<br>f (No dry season)<br>s (Dry summer)	a (Hot summer)<br>b (Warm summer)<br>c (Cold summer)<br>d (Very cold winter)

Figure S2. Köppen-Geiger climate classification: the main climate region (A-D) and detailed climate conditions (right column) where GBGs are implemented.

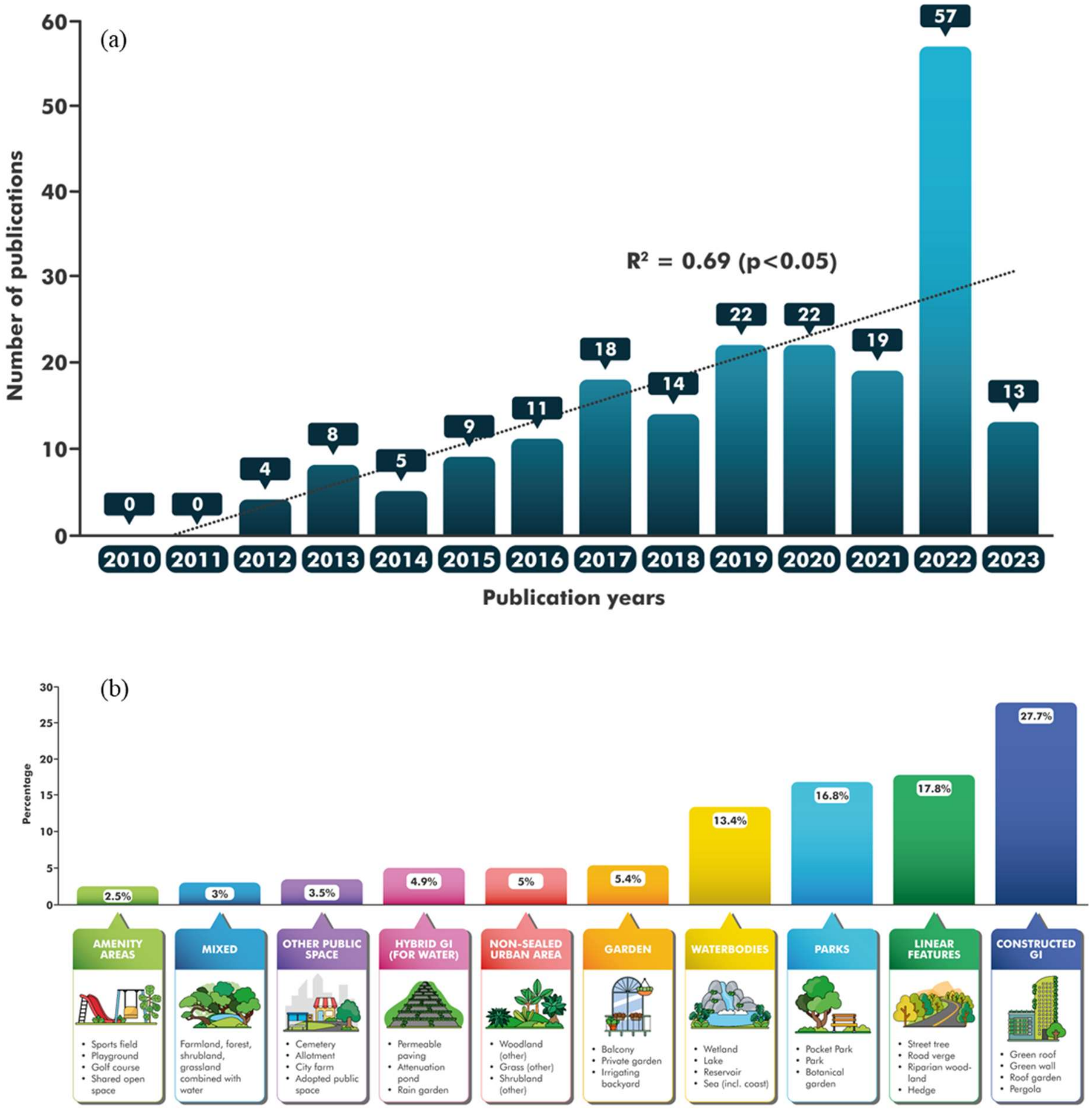

Figure S3. (a) The number of publications exhibiting a significant linear increase over time, with the linear trend (R^2=0.69; p=0.00043) providing the best fit among the various trends (exponential, linear, polynomial, power functions) analysed. Our search in 2023 was limited to the month of 30 March 2023, and the trend line did not incorporate the 2023 data as it did not cover the entire year. (b) The number of publications in each of the 10 main GBGI categories. The number of publications covering all the GBGI sub-categories is shown in Figure 1a.

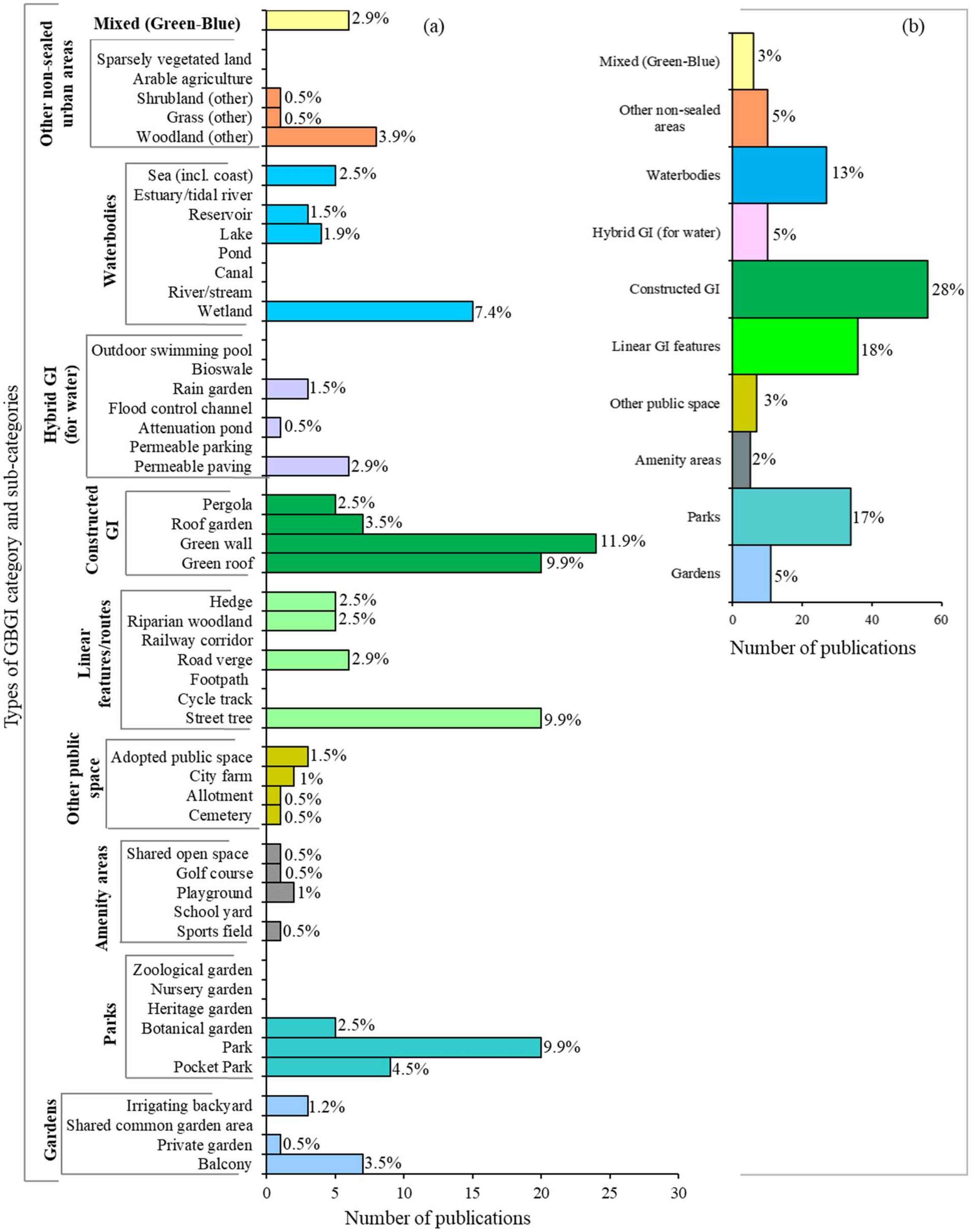

Figure S4. Relevant publications on the benefits of GBGI for heat adaptation and mitigation evidence gathered from the literature: (a) number of publications covering the main categories and sub-categories and (b) number of publications covering the main category (shown by the bold coloured text). The percentage values are printed on the top of each bar.

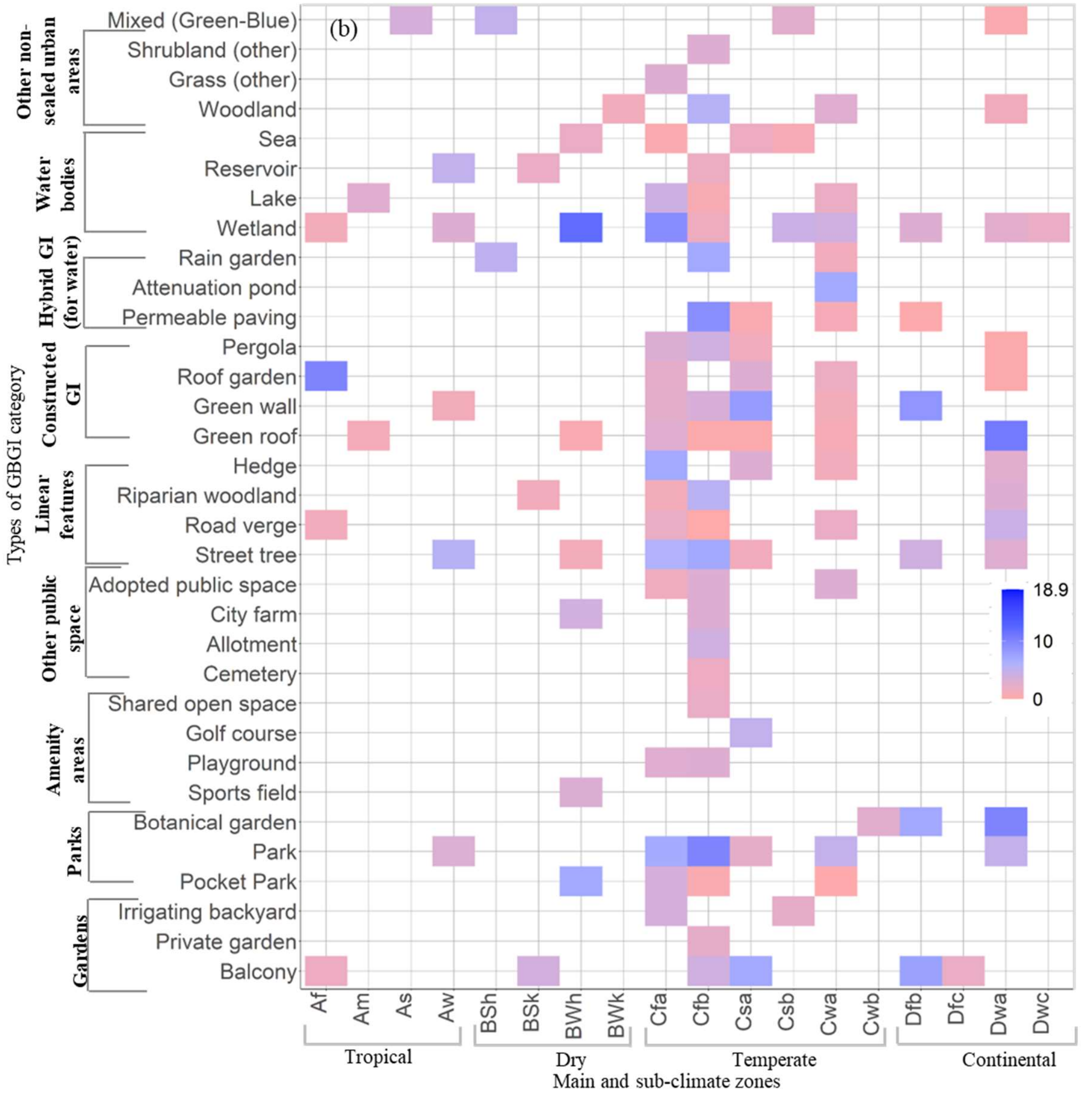

Figure S5. The effectiveness of the main and sub-GBGI categories implemented in tropical (n = 16), dry (n = 15), temperate (n = 137), and continental (n = 34) climate zones of Köppen-Geiger climate classification.

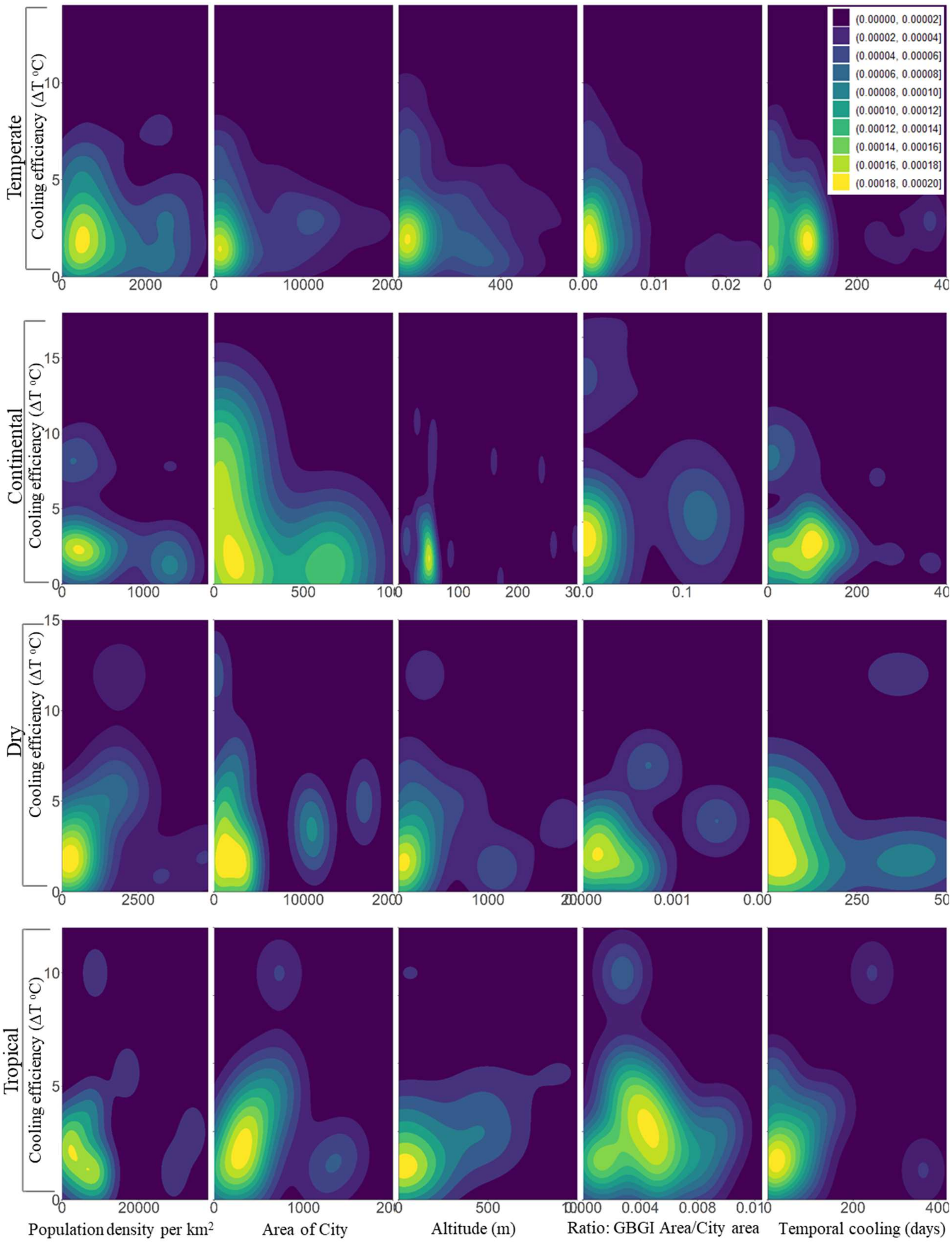

Figure S6. The density of GBGI cooling efficiency in different climate zones and against population density, area of the city, altitude, ratio: area of GBGI/area of the city, and temporal scale of cooling.

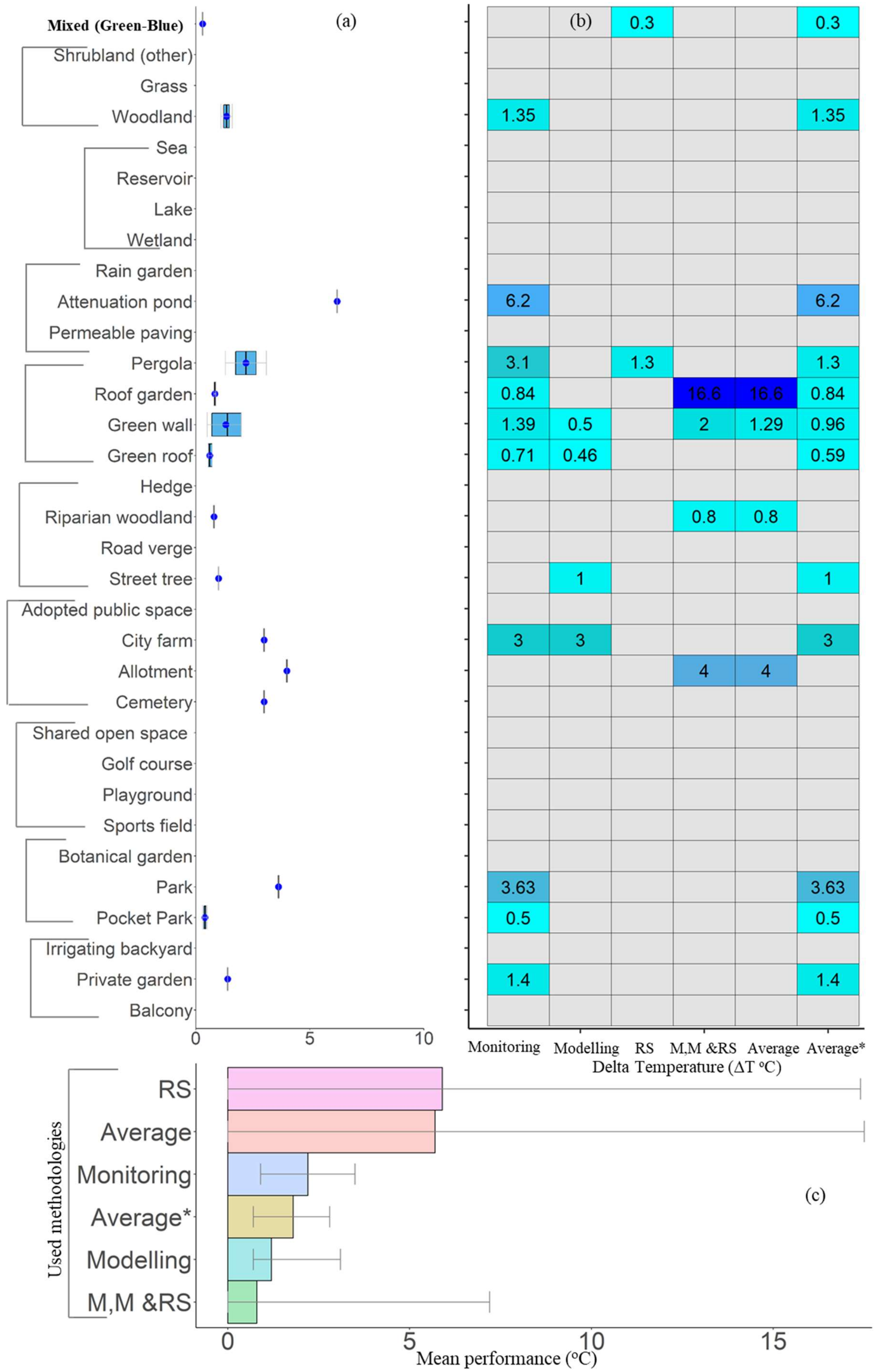

(a)
(b)
(c)
Mixed (Green-Blue)
Shrubland (other)
Grass
Woodland
Sea
Reservoir
Lake
Wetland
Rain garden
Attenuation pond
Permeable paving
Pergola
Roof garden
Green wall
Green roof
Hedge
Riparian woodland
Road verge
Street tree
Adopted public space
City farm
Allotment
Cemetery
Shared open space
Golf course
Playground
Sports field
Botanical garden
Park
Pocket Park
Irrigating backyard
Private garden
Balcony
Monitoring
Modelling
RS
M,M &RS
Average
Average*
Delta Temperature (ΔT ºC)
Used methodologies
Mean performance (ºC)

Figure S7. Night-time temperature reduction efficiency of GBGI sub-categories: (a) a summary of the overall performance of different GBGI types from all studies, (b) heatmap showing GBGI performances from for different methods and the average values, and (c) overall average of GBGI efficiency for urban heat mitigation. The 'Average' and 'Average*' values represent the average of all study types with and without RS data, respectively. M&M denotes combined monitoring and modelling studies. The colour gradient represents the performance, with grey cells representing studies that did not consider either monitoring, modelling, M&M, or RS. The figure uses a boxplot representation with the median indicated by a thick vertical black line, the mean represented by blue dots, and the upper and lower quartiles indicated by the box boundaries. The circle with a vertical line represents the GBGI categories with only one publication.

Table S1. The table below serves as a sample for organising the datasets obtained from the reviewed papers for each of the 51 sub-categories.

Paper Title	Source	Online Link	Study type (modelling, monitoring)	Location (City, Country)	Co-benefits	Multiple GBGIs	ΔT	Day-time ΔT °C	Night-time ΔT °C	Unit	Gap	Dis-benefits	Summary
Evaluating the vertical cooling performances of urban vegetation scenarios in a residential environment	Journal of Building Engineering	https://doi.org/10.1016/j.jobe.2021.102313	Modelling	Changsha, Hunan Province, China ()27°51′-28°40′ N, 111°53′-114°5′E	-	grass, shrubs, trees	0.49 °C	-	-	oC		-	The results of the study show that shadowing can directly affect the cooling effect of vegetation. The cooling effect of vegetation types that provide a large area of shadowing is better, and the green coverage rate cannot directly reflect the shadowing situation of the regional environment.
What's 'cool' in the world of green façades? How plant choice influences the cooling properties of green walls	Building and Environment	https://doi.org/10.1016/j.buildenv.2013.12.005	Monitor	Reading, UK			6.3 oC			oC			Artificial wall sections were used to provide replicated data sets in both outdoor and controlled environmental conditions.
Thermal behavior of a vertical green facade and its impact on the indoor and outdoor thermal environmen	Energy and Building	https://doi.org/10.1016/j.enbuild.2019.109502	Monitoring	Guangzhou, China,			2.7 °C			oC			Measurements and calculations of operative and WBGT temperatures, mass and heat fluxes and energy consumption in two westward identical rooms, one with a green wall system installed. The results indicated that transpiration could consume approximately 50% of solar radiation absorbed by the vegetation canopy.
Impacts of green walls on the characteristics of thermo-flow and photochemical reaction kinetics within street canyons	Urban Forestry and Urban Greening	https://doi.org/10.1016/j.ufug.2022.127568	Numerical modelling				1.02°C			oC			CFD looking on the effect of GWs on wind, temp, CO and NO2/NO/O3 fields in a street cayon. Four scenarios. Results are discussed in tamp reduction and co-benefits cells
A Hedera green façade – Energy performance and saving under different maritime-temperate, winter weather conditions	Building and Environment	https://doi.org/10.1016/j.buildenv.2015.04.011	Monitoring	University of Reading, UK			3 oC			oC			Temperature differences were affected by weather parameters, aspect, diurnal time and canopy density. Largest savings in energy due to vegetation were associated with more extreme weather, such as cold temperatures, strong wind or rain.

Table S2. The string of keywords used to record literature for the review of the efficiency of 51 GBGI categories to mitigate heat.

**GBGI Type**	**Keywords**	**Number of publications Identified**
		**Web of Science**
**Pocket park**	pocket park AND heat waves	-
	pocket park AND urban heat island	14
	pocket park AND temperature reduction	3
	pocket park AND cooling	10
	**Pocket Park (Total)**	27
**Park**	park NOT pocket park AND heat waves	1623
	park NOT pocket park AND urban heat island	907
	park NOT pocket park AND temperature reduction	6804
	park NOT pocket park AND cooling	9741
	**Park (Total)**	**19075**
**Botanical garden**	botanical garden OR arboretum AND heat waves	1
	botanical garden OR arboretum AND urban heat island	10
	botanical garden OR arboretum AND temperature reduction	10
	botanical garden OR arboretum AND cooling	20
	**Botanical garden (Total)**	**41**
**Heritage garden**	heritage garden AND heat waves	3
	heritage garden AND urban heat island	3
	heritage garden AND temperature reduction	1
	heritage garden AND cooling	6
	**Heritage garden (Total)**	13

**Nursery garden**	nursery garden AND heat waves	-
	nursery garden AND urban heat island	-
	nursery garden AND temperature reduction	12
	nursery garden AND cooling	3
	**Nursery garden (Total)**	15
**Zoological garden**	zoo OR zoos OR zoological garden AND heat waves	2
	zoo OR zoos OR zoological garden AND urban heat island	1
	zoo OR zoos OR zoological garden AND temperature reduction	12
	zoo OR zoos OR zoological garden AND cooling	31
	**Zoological garden (Total)**	46
**Street Tree**	street tree AND heat waves	51
	street tree AND urban heat island	326
	street tree AND temperature reduction	123
	street tree AND cooling	246
	**Street Tree (Total)**	746
**Cycle track**	cycle path OR cycle track AND heat waves	6
	cycle path OR cycle track AND urban heat island	14
	cycle path OR cycle track AND temperature reduction	421
	cycle path OR cycle track AND cooling	896
	**Cycle track (Total)**	1337
**Footpath**	footpath AND heat waves	-
	footpath AND urban heat island	6
	footpath AND temperature reduction	0
	footpath AND cooling	1
	**Footpath (Total)**	7

**Road verge**	roadside OR verge AND heat waves	6
	roadside OR verge AND urban heat island	26
	roadside OR verge AND temperature reduction	48
	roadside OR verge AND cooling	103
	**Road verge (Total)**	183
**Railway corridor**	rail AND heat waves	31
	rail AND urban heat island	7
	rail AND temperature reduction	300
	rail AND cooling	456
	**Railway corridor (Total)**	794
**Riparian woodland**	riparian tree OR riparian wood OR riparian forest AND heat waves	4
	riparian tree OR riparian wood OR riparian forest AND urban heat island	4
	riparian tree OR riparian wood OR riparian forest AND temperature reduction	16
	riparian tree OR riparian wood OR riparian forest AND cooling	28
	**Riparian woodland (Total)**	52
**Hedge**	hedge AND heat waves	3
	hedge AND urban heat island	9
	hedge AND temperature reduction	47
	hedge AND cooling	52
	**Hedges (Total)**	111
**Green Roof**	green roof AND heat waves	104
	green roof AND urban heat island	806
	green roof AND temperature reduction	360
	green roof AND cooling	886

	**Green Roof (Total)**	2156
**Green Wall**	green wall OR green facade AND heat waves	57
	green wall OR green façade AND urban heat island	295
	green wall OR green façade AND temperature reduction	298
	green wall OR green façade AND cooling	536
	**Green Wall (Total)**	1186
**Roof garden**	roof garden OR roof terrace AND heat waves	12
	roof garden OR roof terrace AND urban heat island	57
	roof garden OR roof terrace AND temperature reduction	22
	roof garden OR roof terrace AND cooling	80
	**Roof garden (Total)**	171
**Pergola**	pergola AND heat waves	-
	pergola AND urban heat island	5
	pergola AND temperature reduction	5
	pergola AND cooling	7
	**Pergola (Total)**	17
**Road verge**	Road verge AND heat waves	0
	Road verge AND urban heat island	0
	Road verge AND temperature reduction	1
	Road verge AND cooling	2
	(roadside* OR verge*) AND cooling	207
	(roadside* OR verge*) AND heat waves	12
	(roadside* OR verge*) AND urban heat island	29
	(roadside* OR verge*) AND temperature reduction	62
	**Road verge (Total)**	313

**Permeable paving**	Permeable Paving AND Heatwaves	0
	Permeable Paving AND Urban Heat Island	26
	Permeable Paving AND cooling	18
	Permeable Paving AND Temperature reduction	8
	Permeable parking/roadway AND Heatwaves	0
	Permeable parking/roadway AND Urban Heat Island	0
	Permeable parking/roadway AND Temperature reduction	0
	"permeable park*" OR "permeable road Heatwaves reduction"*	13
	"permeable park*" OR "permeable road Urban Heat Island"*	13
	Permeable park*" OR "permeable road Temperature reduction	13
	Permeable Paving AND cooling	18
	**Permeable Paving (Total)**	109
**Attenuation pond**	Attenuation pond AND Heatwaves	0
	Attenuation pond AND Urban Heat Island	1
	Attenuation pond AND Temperature reduction	8
	Attenuation pond AND cooling	6
	**Permeable Paving (Total)**	15
**Permeable paving**	Flood control channel AND Heatwaves	0
	Flood control channel AND Urban Heat Island	1
	Flood control channel AND Temperature reduction	22
	flood* OR channel or Heatwaves*	1
	flood* OR channel or Urban Heat Island*	2
	flood* OR channel or Temperature reduction*	35
	Flood control channel AND cooling	30
	**Flood Control Channel (Total)**	91

**Rain garden**	Rain garden AND Heatwaves	2
	Rain garden AND Urban Heat Island	23
	Rain garden AND Temperature reduction	22
	Rain garden AND cooling	14
	**Rain Garden (Total)**	61
**Bioswale**	Bioswale AND cooling	1
	Bioswale AND Heatwaves	0
	Bioswale AND Urban Heat Island	1
	Bioswale AND Temperature reduction	1
	**Bioswale (Total)**	3
**Outdoor swimming pool**	Outdoor swimming pool AND cooling	16
	Outdoor swimming pool AND Heatwaves	0
	Outdoor swimming pool AND Urban Heat Island	3
	Outdoor swimming pool AND Temperature reduction	3
	**Outdoor swimming pool (Total)**	22
**Canal**	Canal AND cooling	338
	Canal AND Heatwaves	1
	Canal AND Urban Heat Island	4
	Canal AND Temperature reduction	160
	**Canal (Total)**	503
**Estuary/ tidal river**	Estuary/tidal river AND cooling	0
	Estuary/tidal river AND Heatwaves	0
	Estuary/tidal river AND Urban Heat Island	0
	Estuary/tidal river AND Temperature reduction	0
	**Estuary/ tidal river (Total)**	0
**River/ Stream**	River/stream AND cooling	8
	River/stream AND Heatwaves	0

	River/stream AND Urban Heat Island	0
	River/stream AND Temperature reduction	7
	river* OR stream* AND Heatwaves	0
	river* OR stream* AND Urban Heat Island	0
	river* OR stream*AND Temperature reduction	0
	**River/ Stream (Total)**	15
**River/ Stream**	Reservoir AND cooling	3955
	Reservoir AND Heatwaves	15
	Reservoir AND Urban Heat Island	44
	Reservoir AND Temperature reduction	0
	**Reservoir (Total)**	4014
**Lake**	Lake AND Heatwaves	53
	Lake AND Urban Heat Island	167
	Lake AND Temperature reduction	0
	Lake AND cooling	4715
	**Lake (Total)**	4935
**Sea**	Sea (incl. coast) AND Heatwaves	0
	Sea (incl. coast) AND Urban Heat Island	495
	Sea (incl. coast) AND Temperature reduction	6957
	Sea AND cooling	15381
	(sea OR seaside OR coast* OR beach* OR shore and Heatwaves*)	0
	**Sea (Total)**	22833
**Pond**	Pond AND Heatwaves	0
	Pond AND Urban Heat Island	0
	Pond AND cooling	945
	Pond AND Temperature reduction	0

	**Pond (Total)**	945
**Balcony/terrace**	Balcony AND Heatwaves	1
	Balcony AND Urban Heat Island	7
	Balcony AND Temperature reduction	21
	terrace AND Urban Heat Island	16
	terrace AND Urban Heatwaves	1
	terrace AND Temperature reduction	233
	**Balcony/terrace (Total)**	279
**Road verge**	Riparian woodland AND heat waves	1
	Riparian woodland AND urban heat island	0
	Riparian woodland AND temperature reduction	7
	Riparian woodland AND cooling	20
	("riparian tree*" OR "riparian wood*" OR "riparian forest*") AND cooling	66
	("riparian tree*" OR "riparian wood*" OR "riparian forest*") AND heat waves	3
	("riparian tree*" OR "riparian wood*" OR "riparian forest*") AND urban heat island	4
	("riparian tree*" OR "riparian wood*" OR "riparian forest*") AND temperature reduction	30
	**Riparian woodland (Total)**	131
**Playground**	Playground AND Heatwaves	1
	Playground AND Urban Heat Island	9
	Playground AND Temperature reduction	19
	Playground AND cooling	33
	**Playground (Total)**	62
**Golf course**	Golf course AND Heatwaves	0
	Golf course AND Urban Heat Island	4

	Golf course AND Temperature reduction	11
	Golf course AND cooling	111
	**Golf course(Total)**	126
**Shared open space**	Shared open space AND Heatwaves	1
	Shared open space AND Urban Heat Island	4
	Shared open space AND Temperature reduction	10
	Shared open space AND cooling	44
	**Shared open space(Total)**	59
**Cemetery**	Cemetery AND Heatwaves	0
	Cemetery AND Urban Heat Island	5
	Cemetery AND Temperature reduction	1
	Cemetery AND cooling	12
	**Cemetery (Total)**	18
**Allotment**	Allotment AND Temperature reduction	5
	Allotment AND Urban Heat Island	6
	Allotment AND Temperature reduction	5
	Allotment AND cooling	15
	**Allotment (Total)**	31
**City farm**	City farm AND Heatwaves	1
	City farm AND Urban Heat Island	23
	City farm AND Temperature reduction	20
	City farm AND cooling	44
	**City farm (Total)**	88
**Adopted public space**	Adopted public space AND Heatwaves	1
	Adopted public space AND Urban Heat Island	13
	Adopted public space AND Temperature reduction	8

	Adopted public space AND cooling	18
	**Adopted public space (Total)**	40
**Woodland**	Woodland (other) AND Heatwaves	3
	Woodland (other) AND Urban Heat Island	8
	Woodland (other) AND Temperature reduction	34
	Woodland AND cooling	457
	**Woodland (Total)**	502
**Grass (other)**	Grass (other) AND Heatwaves	9
	Grass (other) AND Urban Heat Island	40
	Grass (other) AND Temperature reduction	155
	Grass (other) AND cooling	3041
	**Grass (other) (Total)**	3245
**Arable agriculture**	Arable agriculture AND Heatwaves	0
	Arable agriculture AND Urban Heat Island	0
	Arable agriculture AND Temperature reduction	0
	Arable agriculture AND cooling	24
	**Arable agriculture (Total)**	24
**Private Garden**	Private garden AND Heatwaves	0
	Private garden AND Urban Heat Island	5
	Private garden AND Temperature reduction	1
	Private garden AND cooling	13
	**Private Garden (Total)**	19
**Shared common garden**	Shared common garden area AND Heatwaves	0
	Shared common garden area AND Urban Heat Island	1
	Shared common garden area AND Temperature reduction	0

	Shared common garden area AND cooling	0
	**Shared Common garden (Total)**	1
**Wetland**	Wetland AND Heatwaves	15
	Wetland area AND Urban Heat Island	55
	Wetland AND Temperature reduction	589
	Wetland AND cooling	587
	**Wetland (Total)**	1246
**Estuary**	Estuary AND Heatwaves	22
	Estuary AND Urban Heat Island	13
	Estuary AND Temperature reduction	474
	Estuary AND cooling	428
	**Estuary (Total)**	937
**Sports fields**	Sports field AND Heatwaves	1
	Sports field AND Urban Heat Island	2
	Sports field AND Temperature reduction	25
	Sports field AND cooling	102
	**Sports field (Total)**	130
**School yard**	School yard AND Heatwaves	0
	School yard AND Urban Heat Island	2
	School yard AND Temperature reduction	0
	School yard AND cooling	3
	**School yard (Total)**	5
**Shrubland**	Shrubland (other) AND Heatwaves	0
	Shrubland (other) AND Urban Heat Island	0
	Shrubland (other) AND Temperature reduction	0
	Shrubland (other) AND cooling	16

	**Shrubland (Total)**	16
**Sparsely vegetated land**	Sparsely vegetated land AND Heatwaves	0
	Sparsely vegetated land AND Urban Heat Island	0
	Sparsely vegetated land AND Temperature reduction	0
	Sparsely vegetated land AND cooling	8
	**Sparsely vegetated land (Total)**	8

Table S3. Classification matrix to categorise the number of studies available for each of the GBGI sub-categories in terms of heat mitigation. Zero values indicate that there is negligible empirical evidence available for GBGI's against urban heat mitigation, including heatwaves.

Scale	Conditional performance (%)[a]	Evidence-based classification (%)[b]	Number of GBGI types[c]
1	None	0	18
2	Very low	≥0 ≤20	15
3	Low	≥20 ≤40	9
4	Medium	≥40 ≤ 60	4
5	High	≥60 ≤80	1
6	Very High	≥80	4

[a]The number of publications from negligible to very high under the six-classification scale. [b]The percentage availability of publications for each classification scale. [c]The number of GBGI types found in each of the classification scales.

Table S4. The best performing GBGI types in each climate zone and sub-climate type with reported magnitude and measured scale (i.e. inside/outside of GBGI) of cooling providing details of surroundings.

	GBGI (Sub climate#1, and del T)	GBGI (sub climate#2, and del T)	Inside/outside (scale)	Nearby surrounding
Temperate	Wetland (Cfa, 10)	Park (Cfb, 9.2)	Wetland works well at meso scale (mostly	Near built-up area (park),

			inside) than micro, on the contrary park works better on micro-scale (inside and outside both).	Near nature (wetland)
Continental	Greenwall, Botanical garden (Dfb, 8.7 & 10)	Green roof and Botanical garden (Dwa, 10.8 & 10)	Works well at Microscale but not at meso and macro but the park can be suggested at the mesoscale.<br><br>Green roof (inside, outside and top) botanical garden (inside-outside), green wall (near)	Built-up area (green wall and botanical garden)<br><br>Green roof (mixed environment of grey and nature)
Dry	Wetland, Pocket park (BWh, 12 & 7))	-	Microscale wetland (inside) pocket park (inside-outside)	Wetland (near nature)<br><br>Pocket park (built-up area)
Tropical	Roof garden (Af, 10)	-	Microscale<br><br>(inside and top)	Built up area

Table S5. Sample size (n), correlation of population and p-value density, city area, altitude, ratio of GBGI and city area, and temporal cooling with reported cooling by GBGI in four different climatic conditions i.e. continental, dry, temperate and tropical.

Climate Zone		Correlation	P-value
Population density (n = 34)	Continental	0.0183	0.919
(n = 15)	Dry	0.193	0.4791
(n = 137)	Temperate	-0.013	0.8827
(n = 16)	Tropical	0.2241	0.404
City Area	Continental	0.018	0.9189

(n = 15)	Dry	0.1981	0.4791
(n = 137)	Temperate	-0.013	0.8827
(n = 16)	Tropical	0.22	0.404
Altitude	Continental	0.135	0.4449
(n = 15)	Dry	0.0158	0.9552
(n = 137)	Temperate	-0.089	0.2975
(n = 16)	Tropical	0.325	0.2188
Ratio GBGI/city	Continental	0.0917	0.606
(n = 15)	Dry	-0.117	0.6768
(n = 137)	Temperate	-0.017	0.8386
(n = 16)	Tropical	-0.328	0.2142
Temporal cooling	Continental	0.208	0.2367
(n = 15)	Dry	0.1132	0.6879
(n = 137)	Temperate	0.0399	0.6427
(n = 16)	Tropical	0.2824	0.2892

Table S6. The location, study types (in-situ, modelling, combined (in-situ and modelling), and remote sensing), and performance in reducing temperature (**ΔT °C**)) of different types of GBGI categories against extreme heat extracted from 202 papers.

**GBGI Type**		**GBGI Categories**	**Location (city, country)**	**Study type**	**Performance ΔT (°C)**	**Reference (Year)**
Gardens		Balcony	Vienna	Monitoring	4	Teichmann et al.[31]
			Malaysia	Monitoring	1.7	Toe and Kubota[32]
			Tehran, Iran	Modelling	7	Aghasizadeh et al.[33]
			China	Modelling	3.8	Cui and Zheng[34]
			Lublin,	Monitoring	0.22	Grudzińska[35]

			Poland			
			Tampere, Finland	Monitoring	2	Hilliaho et al.[36]
			Zamo, Poland	Modelling	7.6	Grudzińska[37]
		Private garden	Melbourne, Australia	Monitoring	2.3	Cheung et al.[38]
		Irrigating backyard	Sydney, Australia	Modelling	3	Gao et al.[39]
			Adelaide, Australia	Modelling	2.3	Broadbent et al.[40]
			United States	Remote Sensing	3.74	Wang et al.[41]
Parks		Pocket Park	Hong Kong	Monitoring	0.38	Lau et al.[42]
			Hong Kong	Monitoring	1.09	Lin et al.[433]
			New York	Monitoring	0.5	Rosso et al.[44]
			Veszprém, Hungary	Modelling	0.6	Trájer et al.[45]
			Xi'an, China	Modelling	1.1	Hou et al.[46]
			Xi'an, China	Monitoring & Modelling	0.43	Ma et al.[47]
			Shanghai, China	Monitoring	3.6	Wu et al.[48]
			Cairo Metropolitan Area, Egypt	Modelling	7	Ibrahim[49]
			Hong Kong	Monitoring & Modelling	0.13	Huang et al.[50]
		Park	Chongqing, Southwest China	Modelling	0.8	Lu et al.[51]

			Abuja, Nigeria	Remote Sensing	2.04	Chibuike et al.[52]
			Shenzhen City, China	Monitoring	5.15	Zhang et al.[53]
			Yreb, China	Remote Sensing	2.34	Shi et al.[54]
			Xian, China	Monitoring	0.78	Du et al.[55]
			Shenzhen, China	Remote Sensing	3.02	Peng et al.[56]
			Taipei, Taiwan	Monitoring & Modelling	2.42	Yang et al.[57]
			Wuhan, China	Monitoring	3.5	Chen et al.[58]
			Kolkata Metropolitan Area, India	Remote Sensing	3.15	Das et al.[59]
			Beijing, China	Monitoring	1.38	Zhou et al.[60]
			Melbourne, Australia	Remote Sensing	3.28	Algretawee[61]
			Beijing, China	Remote Sensing	1.71	Qiu and Jia[62]
			Beijing, China	Monitoring	1.09	Li et al.[63]
			Austin, US	Remote Sensing	6.89	Gao et al.[64]
			Taiwan	Monitoring & Modelling	2.42	Yang et al.[65]
			Özgürlük Park, Istanbul, Turkey	Monitoring & Modelling	2.3	Şimşek et al.[66]
			Beijing, China	Monitoring	2.71	Li et al.[67]

			Hong Kong	Monitoring	4.9	Cheung er al.[68]
			Melbourne, Australia	Remote Sensing	10	Algretawee[69]
			Beijing, China	Monitoring	4.8	Yan et al.[70]
		Botanical garden	Baoji, China	Monitoring	2.7	Chang and Li[71]
			Erzurum, Turkey	Monitoring	2.6	Irmak et al.[72]
			Beijing, China	Monitoring	10	Su [73]
			Erzurum, Turkey	Monitoring & Modelling	2.2	Yilmaz et al.[74]
			Erzurum, Turkey	Monitoring	7.1	Yilmaz et al.[75]
Amenity areas		Sports field	Khalifa stadium in Doha, Qatar	Monitoring & Modelling	3.1	Ghani et al.[76]
		Playgroun d	Warsaw, Poland	Monitoring	5	Kuchcik et al.[77]
			United States	Remote Sensing	5.5	Vanos et al.[78]
		Golf course	Perth, Australia	Remote Sensing	6	Nguyen et al.[79]
		Shared open space	Maxvorstadt, Munich	Modelling	2.1	Zölch et al.[80]
Other public spaces		Cemetery	Budapest's, Hungary	Monitoring	1.7	Sallay et al.[81]
		Allotment	Berlin, Germany	Remote Sensing	4	Rost et al.[82]
		City farm	Phoenix, Arizona	Monitoring	3.9	Hawkins et al.[83]
			Paris, France	Modelling	3	Masson et al.[84]

		Adopted public space	Raiganj, West Bengal, India	Monitoring & Modelling	3	Basu and Das[85]
			Bologna, Italy	Modelling	3	Boeri et al.[86]
			Liverpool, NSW, Australia	Modelling	1.5	Abdollahzadeh and Biloria[87]
Linear features/routes		Street tree	Hangzhou city, Zhejiang Province, China	Monitoring	1.8	Cai et al.[88]
			Turin, Italy	Monitoring	0.5	Morabito et al.[89]
			Florence, Italy	Modelling	9.4	Napoli et al.[90]
			Nanjing , Jiangsu Province, China	Modelling	5.5	Xi et al.[91]
			Karachi, Pakistan	Modelling	1.2	Zeeshan et al.[92]
			Abu Dhabi	Monitoring & Modelling	0.9	Abu Ali et al.[93]
			Rome, Italy	Remote Sensing	3.2	Marando et al.[94]
			Karachi, Pakistan	Modelling	1.2	Zeeshan et al.[95]
			Montreal, Canada	Modelling	4	Wang et al.[96]
			Vancouver, Canada	Remote Sensing	12	Lachapelle et al.[97]
			Barcelona, Spain	Modelling	1.3	Segura et al.[98]
			Shenyang,	Monitoring	2.9	Miao et al.[699]

			China			
			Prague Czech Republic	Modelling	5	Geletic et al.[100]
			Basel, Switzerland	Monitoring & Modelling	2	Mussetti et al.[101]
			Bangalore, India	Monitoring	5.6	Valishery et al.[102]
			Dresden, Germany	Monitoring	2.22	Gillner et al.[103]
			Melbourne, Australia	Monitoring	1.5	Coutts et al.[104]
			Richmond, Australia	Monitoring	2.1	Sanusi et al.[105]
			Vancouver, Canada	Modelling	7.1	Aminipour et al.[106]
			Tsukuba City, Japan	Monitoring	5.9	Kusaka et al.[107]
		Road verge	Jongro, Seoul, Republic of Korea	Monitoring	4.44	Cho[108]
			Taipei, Taiwan	Monitoring	0.68	Huang and Li[109]
			New Belgrade, Serbia	Monitoring	2.1	Stojanovic et al.[110]
			Kuala Lumpur, Malaysia	Monitoring	1.3	Zaki et al.[111]
			Haikou, China	Modelling	2	Zheng et al.[8112]
			Czech Republic	Monitoring & Modelling	0.05	Žižlavská et al.[113]

		Riparian woodland	Sydney, Australia	Monitoring	1.16	Adams and Smith[1144]
			Ejina basin, China	Monitoring	1.28	Yonghong et al.[115]
			Yorkshire, England	Monitoring	3	Tsai et al.[116]
			Glen Girnock, UK	Remote sensing	5.4	Dugdale et al.[117]
			Beijing, China	Monitoring	3	Zheng et al.[118]
		Hedge	Beijing, China	Modelling	2.68	Zhang and Hu[119]
			Lazio, Italy	Modelling	3	Peluso et al.[120]
			Rome, Italy	Modelling	3	Del Serrone et al.[121]
			Shenzhen, China	Monitoring & Modelling	1.29	Zou et al.[1222]
			Sakai, Japan	Remote Sensing	7	Yoshida et al.[123]
		Green roof	Berlin, Germany	Modelling	0.44	Wang et al.[124]
			Mandaue, Philippines	Modelling	1.1	Cortes et al.[125]
			Sydney, Australia	Monitoring	9.63	Fleck et al.[126]
			Xiamen, China	Remote Sensing	0.91	Dong et al.[127]
			Belgrade, Serbia	Monitoring	5.5	Kostadinovic et al.[128]
			Nanjing, China	Monitoring	1.1	Peng et al.[129]
			Tseung Kwan O New Town,	Monitoring	4.9	Lee and Jim[130]

			Hong Kong, China			
			Neubrandenburg, Germany	Monitoring	1.5	Kohler and Kaiser[131]
			Gangnam-gu, Seoul, South Korea	Monitoring	10.8	Park et al.[132]
			Jerusalem and Tel Aviv	Monitoring & Modelling	0.4	Lynn and Lynn[133]
			Shenzhen, China	Monitoring	4.03	Chen et al.[134]
			Mandaue, Philippines	Modelling	1.1	Cortes et al.[135]
			Utrecht, The Netherlands	Monitoring	0.2	Solcerova[136]
			Lodz, Poland	Modelling	0.19	Bochenek and Klemm[137]
			Guangzhou, China	Modelling	0.1	Chen et al.[138]
			Hamad, Northern Bahrain	Modelling	0.72	Elnabawi and Saber[139]
			Chengdu, China	Monitoring	0.94	Zuo et al.[140]
			Rome, Italy	Modelling	0.16	Iaria and Susca[141]
			Sydney, Australia	Monitoring	2.92	Fleck et al.[142]
			Cordoba, Argentina	Monitoring	0.892	Robbiati et al.[143]
Constructed GI on infrastructure		Green wall	Changsha, Hunan Province, China	Modelling	0.49	Liao et al.[144]

			Reading, UK	Remote Sensing	6.3	Cameron et al.[145]
			Guangzhou, China	Monitoring	3.6	Zhang et al.[146]
			Shanghai, China	Modelling	1.02	Liu et al.[147]
			Reading, UK	Monitoring	3	Cameron et al.[148]
			Madrid, Spain	Monitoring	2.7	Jesus et al.[149]
			Hong-Kong	Monitoring	1.19	Lee and Jim[150]
			Rio de Janeiro, Brazil	Modelling	1.16	Feitosa and Wilkinson[151]
			Zürich, Switzerland	Modelling	0.1	Li et al.[152]
			Prague, Czech Republic	Monitoring & modelling	2	Geletič et al.[153]
			Ljubljana, Slovenia	Remote Sensing	18.9	Šuklje et al.[154]
			Tyrol, Austria	Monitoring	8.7	Medl et al.[155]
			Sydney, Australia	Monitoring	7.7	Feitosa and Wilkinson[156]
			Bari,Valenzano, Italy	Monitoring	7	Blanco et al.[157]
			Pertth, Western Australia	Monitoring	8.1	Bakhshoodeh et al.[158]
			Quito, Ecuador	Modelling	1.43	Davis et al.[159]
			London Olympic Park	Monitoring	1.5	Hosseinzadeh et al.[160]

			La Rochelle, France	Modelling	1.9	Djedjig et al.[161]
			Chennai, India	Modelling	1.2	Pragati et al.[162]
			Guangzhou, China	Monitoring	8	Lin et al.[163]
			United States	Monitoring	4.3	Price et al.[164]
			Hong Kong	Monitoring	1.2	Lee and Jim [165]
			Munich, Germany	Modelling	3.5	Lin et al.[166]
			Chenzhou, Hunan, China	Modelling	2.56	Li et al.[167]
		Roof garden	Nanjing, China	Monitoring	1	Peng et al.[168]
			Xinxiang, Henan, China	Monitoring	1	Shen [169]
			Duhok, Iraq	Monitoring	3	AbdulBaqi [170]
			Seoul, South Korea	Modelling	0.3	Kim et al.[171]
			Hong Kong	Monitoring	1.8	Lee and Jim [172]
			Singapore	Monitoring	17.7	Tan et al.[173]
			Singapore	Remote Sensing	10	Tan et al.[174]
		Pergola	Nagoya, Japan	Remote Sensing	16.2	Watanabe et al.[175]
			Arta, Greece	Monitoring & Modelling	1.3	Katsoulas et al.[176]
			Lleida, Spain	Monitoring	3.1	Chafer et al.[177]

			Suwon, Republic of Korea	Monitoring	0.2	Kong et al.[178]
			Vienna, Austria	Monitoring & Modelling	4	Teichmann et al.[179]
Hybrid GI		Permeable paving	Vertemate con Minoprio, CO, Italy	Monitoring	2.8	Fini et al.[180]
			Perugia, Italy	Monitoring	9.2	Kousis et al.[181]
			Zhouzhi County, Xi'An, Shaanxi, China	Monitoring	6	Lu et al.[182]
			Rome, Italy	Modelling	0.6	Moretti et al.[183]
			Guangzhou, China	Monitoring	1	Wang et al.[184]
			Changping China	Monitoring	0.19	Wang et al.[185]
		Attenuation pond	Guangzhou, China	Monitoring	7	Yang et al.[186]
		Rain garden	Yau Tsim Mong district, Hong Kong	Modelling	1.3	An et al.[187]
			Tucson, Arizona	Monitoring	5.2	Buzzard et al.[188]
			Gdansk, Poland	Monitoring	7	Kasprzyk et al.[189]
Waterbodies		Wetland	Zoige Plateau, China	Monitoring	2	Bai et al.[190]
			Beijing, China	Remote Sensing	7.83	Cai et al.[191]

			Vienna, Austria	Monitoring	3.4	Pucher et al.[192]
			Avondale, Arizona	Remote Sensing	12	Ruiz-Aviles et al.[193]
			Dhaka, Bangladesh, Anatolia	Modelling	3	Shahjahan et al.[194]
			Central Anatolia, Turkey	Remote Sensing	4.38	Şimşek and Ödül[195]
			Beijing, China	Monitoring	3.15	Sun et al.[196]
			Eastern Germany	Modelling	1.6	Sušnik et al.[197]
			Palembang City, Indonesia	Monitoring	1.2	Triyuly et al.[198]
			Chengdu, China	Monitoring	4.08	Wu et al.[199]
			Wuhan, China	Monitoring	4.8	Xu et al.[200]
			Hangzhou, China	Remote Sensing	9.27	Zhang et al.[201]
			Northeast China	Remote Sensing	8.15	Wenguang et al.[202]
			Prairie Pothole Region, North America	Remote Sensing	3	Zhang et al.[203]
			Beijing, China	Remote Sensing	2.6	Sun et al.[204]
		Lake	Hue Citadel, Hue City, Vietnam	Remote Sensing	2.82	Le Phuc et al.[205]
			Altenberge, Germany	Modelling	0.8	Theeuwes et al.[206]

			Wuhan, China	Monitoring	4.2	Xu et al.[207]
			Daming lake, Jinan, China	Monitoring	1.9	Yang et al.[208]
		Reservoir	São José do Rio Preto, Brazil	Monitoring	5	Masiero and de Souza[209]
			Northern, Spain	Monitoring	2	Novo et al.[210]
			Santander, Spain	Monitoring	1.82	Novo et al.[2111]
		Sea	Athens, Greece	Monitoring & Modelling	1.7	Dandou et al.[212]
			Sendai, Japan	Monitoring	1.3	Zhou et al.[213]
			Adelaide, Australia	Monitoring	0.9	Zhou et al.[214]
			South Australia	Monitoring	2	Zhou et al.[215]
			Wuhan, China	Modelling	0.4	Zhu et al.[216]
Other non-sealed urban area		Woodland	Guildford, UK	Monitoring	5.7	Sahani et al.[217]
			Hong Kong Golf Course	Monitoring	1.43	Fung and Jim[218]
			Hong Kong Golf Course	Monitoring	4.2	Fung and Jim[219]
			Ejina basin	Monitoring & Modelling	1.28	Yonghong et al.[220]
			Baoji Botanical Garden	Monitoring	2.7	Chang and Li[221]
			Beijing, China	Monitoring	1.32	Liu et al.[222]

			Xi'an, China	Remote Sensing	4.32	Ma et al.[223]
			Hong Kong	Monitoring	2.9	Fung and Jim[224]
		Grass (other)	Sydney, Australia	Monitoring	2.94	Adams and Smith[225]
		Shrubland (other)	Howard Valley, South Island, New Zealand	Monitoring	3	Callard et al.[226]
Mixed (Green-Blue)		Mixed (Green-Blue)	Olympic Forest Park, Beijing, China	Monitoring	0.4	Amani-Beni et al.[227]
			Beijing, China	Remote Sensing	1.32	Liu et al.[228]
			Nagpur, Maharashtra	Remote Sensing	3.6	Jain et al.[229]
			Igapó Lak, Latin American city	Monitoring	2.63	Targino et al.[230]
			Beijing, China	Monitoring & Modelling	0.4	Cheung and Jim[231]
			Olympic Area, Beijing, China	Remote Sensing	4.95	Dai et al.[232]

Table S7. The average performance of different types of GBGI categories, which were evaluated using in-situ, modelling, in-situ combined modelling, and remote sensing techniques for heat risk adaptation and mitigations. The '-' symbol indicates 'no data available'.

GBGI	GBGI Category	Monitoring ΔT (°C)	Modelling ΔT (°C)	RS ΔT (°C)	MM ΔT (°C)	Overall ΔT (°C)	Availability
Gardens	Balcony	2.0	6.1	-	-	4.06	Medium
	Private garden	2.3	-	-	-	2.30	Very low
	Irrigating backyard	-	2.7	3.7	-	3.20	Very low
Parks	Pocket Park	1.4	2.9	4.1	0.28	2.16	Medium
	Park	3.0	0.8	-	2.38	2.07	Very High
	Botanical garden	5.6	-	-	2.2	3.90	Low
Amenity areas	Sports field	-	-	-	3.1	3.10	Very low
	Playground	3.0	-	2.8	-	2.90	Very low
	Golf course	-	-	5.0	-	5.00	Very low
	Shared open space	-	2.1	-	-	2.10	Very low
Other public space	Cemetery	1.7	-	-	-	1.70	Very low
	Allotment	-	-	4.0	-	4.00	Very low
	City farm	3.9	3.0	-	-	3.45	Very low
	Adopted public space	-	2.3	-	3	2.63	Very low
Linear GI features/ routes	Street tree	2.8	4.3	7.6	1.45	4.05	Very High
	Road verge	2.1	2.0	-	0.05	1.39	Low

	Riparian woodland	2.1	-	5.4	-	3.76	Low
	Hedge	-	2.9	7.0	1.29	3.73	Low
Constructed GI on infrastructure	Green roof	3.9	0.5	0.9	0.4	1.43	Very High
	Green wall	4.7	1.5	12.6	2	5.21	Very High
	Roof garden	2.1	0.3	7.1	-	3.13	Medium
	Pergola	1.7	-	18.2	2.65	7.50	Low
Hybrid GI (for water)	Permeable paving	3.8	0.6	-	-	2.22	Low
	Attenuation pond	7.0	-	-	-	7.00	Very low
	Rain garden	6.1	1.3	-	-	3.70	Very low
Waterbodies	Wetland	3.1	2.3	6.7	-	4.05	High
	Lake	3.1	0.8	2.8	-	2.22	Low
	Reservoir	2.9	-	-	-	2.94	Very low
	Sea (incl. coast)	1.4	0.4	-	1.7	1.17	Low
Other non-sealed urban areas	Woodland (other)	3.1	-	4.3	1.28	2.89	Medium
	Grass (other)	2.9	-	-	-	2.94	Very low
	Shrubland (other)	3.0	-	-	-	3.00	Very low
Mixed (Green-Blue)	Mixed (Green-Blue)	1.5	-	3.3	0.4	1.74	Low

Table S8. The projected influence of future climate change on the choice of GBGI in various climate zones.

Climate zone	Previous/Current climate	Future climate	Present GBGI	Future GBGI
Continent	Dfa	Dfb	Street trees<br>Permeable paving	Wetland
	Dfb	BSk	Botanical garden	Green wall<br>Street trees
Dry	BSk	BSh	Balcony	Green wall, street trees
	BSk	BWk	Wetland	Woodland
Temperate (Europe)	Cfb	Cfa	Green roofs,<br>Green walls,<br>Woodland<br>Reservoir<br>City farm<br>Riparian woodland	Parks<br>Pocket parks<br>Green walls<br>Green roofs<br>Lakes<br>Grass
	Dfb	Cfa	Green roof<br>Balcony<br>Road verge<br>Playground	Parks<br>Pocket parks<br>Green walls<br>Green roofs<br>Lakes<br>Grass
Temperate (China)	Cwa	Am	Park<br>Green wall<br>Green roof<br>Rain garden	Lakes<br>Road verge
	Dwa	Cwa	Woodland<br>Lake<br>Green roof<br>Road verge<br>Pergola<br>Roof garden	Green roof<br>Adopted space<br>wetland

338 **Table 9**. Summary of key stages and action points for implementing, replicating, and
339 upscaling GBGI to mitigate urban heat.

Stages	Action points
Stakeholder engagement[a]	● Engage stakeholders early on, and from various sectors such as urban planning, public health, environmental agencies, and community organisations to identify and frame the heat risk problem and understand their concerns and priorities.<br>● Foster collaboration and participatory decision-making processes to ensure diverse perspectives are considered.<br>● Conduct workshops, interviews, and surveys to gather input and feedback from stakeholders.<br>● Involve residents, local businesses, and community groups to increase awareness and support.
Feasibility study of GBGI[b]	● Conduct a preliminary cost-benefit analysis to assess the feasibility and potential effectiveness of different GBGI measures.<br>● Consider factors such as implementation costs, maintenance requirements, technical feasibility, and expected benefits in terms of heat reduction and other co-benefits.<br>● Identify suitable locations for implementation based on the analysis of UHI intensity and vulnerability maps/zones.<br>● Explore funding options and potential partnerships to support implementation.
Assess co-benefits and dis-benefits of the GBGI[c]	● Consider the multiple co-benefits associated with GBGI, such as improved air quality, reduced stormwater runoff, enhanced biodiversity, and increased recreational opportunities.<br>● Assess potential dis-benefits, such as increased maintenance requirements, potential conflicts with existing infrastructure, allergic reactions, and displacement of vulnerable populations due to gentrification.<br>● Conduct a comprehensive cost-benefit analysis to evaluate the overall value and trade-offs of implementing GBGI.

Design GBGI measures[d]	● Select suitable GBGI measures based on the local context, including the climate, topography, available space, and community preferences.<br>● Incorporate GBGI elements such as trees, green roofs, green walls, and permeable surfaces to maximise shade, evapotranspiration, and cooling effects.<br>● Consider the use of native and drought-tolerant plant species for long-term sustainability and reduced water demand.<br>● Ensure proper placement and spacing of vegetation to optimise shading and air movement.
Policy and planning[e]	● Integrate GBGI strategies into urban planning and policy frameworks, such as comprehensive plans, zoning ordinances, and building codes.<br>● Develop heat mitigation plans that prioritise the implementation of GBGI measures in high-risk areas.<br>● Provide incentives, regulations, and guidelines to encourage the adoption of GBGI in private and public developments.<br>● Collaborate with relevant organisations to ensure coordination and alignment of policies, goals, and levelling up of sustainability agenda (e.g., SDGs, European Green Deal, Paris Climate Agreement).
Implementation[f]	● Establish partnerships and collaborations between governmental agencies, private sector organisations, and community groups for effective implementation.<br>● Allocate sufficient resources, including funding, staff, and technical expertise, for the installation and maintenance of the selected GBGI measures.<br>● Ensure proper construction practices and quality control to maximise the performance and longevity of implemented measures.<br>● Incorporate community engagement and education programs to foster stewardship and long-term support for the solutions in place.

Monitoring[g]	● Utilise relevant or a combination of in-situ measurements, remote sensing, and modelling methods to monitor the performance and effectiveness of GBGI used against heatwaves.<br>● Deploy and use weather stations, sensors, and satellite imagery to evaluate the efficacy of the GBGI measures.<br>● Collect data on temperature, humidity, air quality, and vegetation health to evaluate the impact of implemented measures.<br>● Employ modelling tools to simulate the cooling effects and assess potential future scenarios.
Evaluation[h]	● Conduct a comprehensive evaluation of the implemented GBGI measures to assess their effectiveness and cost-effectiveness.<br>● Compare the heat risk before and after implementation using temperature data, health indicators, and energy consumption.<br>● Analyse the economic, social, and environmental benefits achieved through the implementation of GBGI.<br>● Incorporate feedback from stakeholders and learn from the implementation process to inform future improvements.
Upscaling and replication[i]	● Develop strategies for upscaling and replicating successful GBGI measures in different neighbourhoods and cities.<br>● Share successful case studies and best practices to encourage replication in other areas and facilitate upscaling of GBGI measures.<br>● Adapt the GBGI approach to suit local contexts, considering factors like climate, social dynamics, and available resources.<br>● Develop training programs and capacity-building initiatives to support the replication and upscaling of GBGI measures.<br>● Foster knowledge exchange among cities and regions.

[a]Sherman and Ford[233]; O'Brien et al.[234]; [b]Coutts et al.[235]; [c]Curt et al.[236]; Ommer et al.[237]; [d]Dumitru et al.[238]; Kumar et al.[239]; [e]Davies et al.[240]; [e]European Green Deal[241]; [f]Di Pirro et al.[242]; Topal et al.[243]; [g]Augusto et al.[244]; [h]Frantzeskaki[245]; [i]Cortinovis et al.[246].

References

1. Mell, I., Allin, S., Reimer, M., & Wilker, J. (2017). Strategic green infrastructure planning in Germany and the UK: a transnational evaluation of the evolution of urban greening policy and practice. International Planning Studies 22, 333–349.
2. Wright, H. (2011). Understanding green infrastructure: the development of a contested concept in England. Local Environment 16, 1003-1019.
3. Byrne, J.A., Lo, A.Y., & Jianjun, Y. (2015). Residents' understanding of the role of green infrastructure for climate change adaptation in Hangzhou, China. Landscape and urban planning 138, 132-143.
4. Jones, L., Anderson, S., Læssøe, J., Banzhaf, E., Jensen, A., Bird, D.N., Miller, J., Hutchins, M.G., Yang, J., Garrett, J., & Taylor, T. (2022). A typology for urban green infrastructure to guide multifunctional planning of nature-based solutions. Nature-Based Solutions 2, 100041.
5. Debele, S.E., Kumar, P., Sahani, J., Marti-Cardona, B., Mickovski, S.B., Leo, L.S., Porcù, F., Bertini, F., Montesi, D., Vojinovic, Z., & Di Sabatino, S. (2019). Nature-based solutions for hydro-meteorological hazards: Revised concepts, classification schemes and databases. Environmental Research 179, 108799.
6. Cheung, P.K., Jim, C.Y., Tapper, N., Nice, K.A., & Livesley, S.J. (2022). Daytime irrigation leads to significantly cooler private backyards in summer. Urban Climate 46, 101310.
7. Castellar, J.A., Popartan, L.A., Pueyo-Ros, J., Atanasova, N., Langergraber, G., Säumel, I., Corominas, L., Comas, J., & Acuna, V. (2021). Nature-based solutions in the urban context: Terminology, classification and scoring for urban challenges and ecosystem services. Science of the Total Environment 779, 146237.
8. Demuzere, M., Orru, K., Heidrich, O., Olazabal, E., Geneletti, D., Orru, H., Bhave, A.G., Mittal, N., Feliú, E., & Faehnle, M. (2014). Mitigating and adapting to climate change: Multi-functional and multi-scale assessment of green urban infrastructure. Journal of Environmental Management 146, 107-115.
9. Rogers, C.D.F., & Hunt, D.V.L. (2019). Realising Visions for Future Cities: An Aspirational Futures Methodology. Proceedings of the Institution of Civil Engineers – Urban Design and Planning 172, 125-140.
10. Moher, D., Liberati, A., Tetzlaff, J., & Altman, D.G. (2009). Preferred reporting items for systematic reviews and meta-analyses: the PRISMA statement. Annals of Internal Medicine 151, 264-269.
11. R Core Team (2022). R: A language and environment for statistical computing. R Foundation for Statistical Computing, Vienna, Austria. URL https://www.R-project.org/.
12. Mazziotta, M., & Pareto, A. (2022). Normalization methods for spatio-temporal analysis of environmental performance: Revisiting the Min–Max method. Environmetrics 33, e2730.
13. Cai, Y., Li, C., Ye, L., Xiao, L., Gao, X., Mo, L., Du, H., Zhou, Y., & Zhou, G. (2022). Effect of the roadside tree canopy structure and the surrounding on the daytime urban air temperature in summer. Agricultural and Forest Meteorology 316, 108850.
14. Morabito, M., Crisci, A., Guerri, G., Messeri, A., Congedo, L. & Munafò, M. (2021). Surface urban heat islands in Italian metropolitan cities: Tree cover and impervious surface influences. Science of the Total Environment 751, 142334.
15. Xi, C., Wang, D., & Cao, S.J. (2023). Impacts of trees-grass area ratio on thermal environment, energy saving, and carbon benefits. Urban Climate 47, 101393.

16. Windbourne, J., Jones, T., Garvey, S., Harrison, J., Wang, L., Li, D., Templer, P., & Hutyra, L. (2020). Tree Transpiration and Urban Temperatures: Current Understanding, Implications, and Future Research Directions. BioScience 70, 576–588.
17. Lu, J., Li, Q., Zeng, L., Chen, J., Liu, G., Li, Y., Li, W., & Huang, K. (2017). A micro-climatic study on cooling effect of an urban park in a hot and humid climate. Sustainable cities and society 32, 513-522.
18. Chibuike, E.M., Ibukun, A.O., Abbas, A., & Kunda, J.J. (2018). Assessment of green parks cooling effect on Abuja urban microclimate using geospatial techniques. Remote Sensing Applications: Society and Environment 11, 11-21.
19. Peng, J., Dan, Y., Qiao, R., Liu, Y., Dong, J., & Wu, J. (2021). How to quantify the cooling effect of urban parks? Linking maximum and accumulation perspectives. Remote Sensing of Environment 252, 112135.
20. Gunawardena, K.R., Wells, M.J., & Kershaw, T. (2017). Utilising green and bluespace to mitigate urban heat island intensity. Science of the Total Environment 584, 1040-1055.
21. Lynn, B.H. & Lynn, I.M. (2020). The impact of cool and green roofs on summertime temperatures in the cities of Jerusalem and Tel Aviv. Science of the Total Environment 743, 140568.
22. Iaria, J., & Susca, T. (2022). Analytic Hierarchy Processes (AHP) evaluation of green roof-and green wall-based UHI mitigation strategies via ENVI-met simulations. Urban Climate 46, 101293.
23. Li, H., Zhao, Y., Sützl, B., Kubilay, A., & Carmeliet, J. (2022). Impact of green walls on ventilation and heat removal from street canyons: Coupling of thermal and aerodynamic resistance. Building and Environment 214, 108945.
24. Feitosa, R.C., & Wilkinson, S.J. (2020). Small-scale experiments of seasonal heat stress attenuation through a combination of green roof and green walls. Journal of Cleaner Production 250, 119443.
25. Sun, R., Chen, A., Chen, L., & Lü, Y. (2012). Cooling effects of wetlands in an urban region: The case of Beijing. Ecological Indicators 20, 57-64.
26. Zhang, Z., Chen, F., Barlage, M., Bortolotti, L.E., Famiglietti, J., Li, Z., Ma, X., & Li, Y. (2022). Cooling Effects Revealed by Modeling of Wetlands and Land-Atmosphere Interactions. Water Resources Research 58, e2021WR030573.
27. Ampatzidis, P, & Kershaw, T. (2020). A review of the impact of blue space on the urban microclimate. Science of the Total Environment 730, 139068.
28. Hathway, E.A., & Sharples, S. (2012). The interaction of rivers and urban form in mitigating the Urban Heat Island effect: A UK case study. Building and Environment 58, 14-22.
29. Wu, C., Li, J., Wang, C., Song, C., Chen, Y., Finka, M., & La Rosa, D. (2019). Understanding the relationship between urban blue infrastructure and land surface temperature. Science of the Total Environment 694, 133742.
30. Wu, S., Yang, H., Luo, P., Luo, C., Li, H., Liu, M., Ruan, Y., Zhang, S., Xiang, P., Jia, H., & Cheng, Y. (2021). The effects of the cooling efficiency of urban wetlands in an inland megacity: A case study of Chengdu, Southwest China. Building and Environment 204, 108128.
31. Teichmann, F., Horvath, A., Luisser, M., & Korjenic, A. (2022). The Impact of Small-Scale Greening on the Local Microclimate—A Case Study at Two School Buildings in Vienna. Sustainability 14, 13089.

32. Toe, D. H. C., & Kubota, T. (2015). Comparative assessment of vernacular passive cooling techniques for improving indoor thermal comfort of modern terraced houses in hot–humid climate of Malaysia. Solar Energy 114, 229–258.
33. Aghasizadeh, S., Kari, B. M., & Fayaz, R. (2022). Thermal performance of balcony thermal bridge solutions in reinforced concrete and steel frame structures. Journal of Building Engineering 48, 103984.
34. Cui, Y., & Zheng, H.-C. (2016). Impact of Three-Dimensional Greening of Buildings in Cold Regions in China on Urban Cooling Effect. Procedia Engineering 169, 297–302.
35. Grudzińska, M. (2021). Overheating assessment in flats with glazed balconies in warm-summer humid continental climate. Building Services Engineering Research and Technology 42, 583–602.
36. Hilliaho, K., Köliö, A., Pakkala, T., Lahdensivu, J., Vinha, J. (2016). Effects of added glazing on Balcony indoor temperatures: Field measurements. Energy and Buildings 128, 458-472.
37. Grudzińska, M., 2021. Overheating assessment in flats with glazed balconies in warm-summer humid continental climate. Building Services Engineering Research and Technology 42, 583-602.
38. Cheung, P. K., Jim, C. Y., Tapper, N., Nice, K. A., & Livesley, S. J. (2022). Daytime irrigation leads to significantly cooler private backyards in summer. Urban Climate 46, 101310.
39. Gao, K., Santamouris, M., & Feng, J. (2020). On the cooling potential of irrigation to mitigate urban heat island. Science of the Total Environment 740, 139754.
40. Broadbent, A. M., Coutts, A. M., Tapper, N. J., & Demuzere, M. (2018). The cooling effect of irrigation on urban microclimate during heatwave conditions. Urban Climate 23, 309–329.
41. Wang, C., Wang, Z.-H., & Yang, J. (2019). Urban water capacity: Irrigation for heat mitigation. Computers, Environment and Urban Systems 78, 101397.
42. Lau, S. S., Lin, P., & Qin, H. (2012). A preliminary study on environmental performances of pocket parks in high-rise and high-density urban context in Hong Kong. International Journal of Low-Carbon Technologies 7, 215–225.
43. Lin, P., Lau, S. S. Y., Qin, H., & Gou, Z. (2017). Effects of urban planning indicators on urban heat island: a case study of pocket parks in high-rise high-density environment. Landscape and Urban Planning 168, 48–60.
44. Rosso, F., Pioppi, B., & Pisello, A. L. (2022). Pocket parks for human-centered urban climate change resilience: Microclimate field tests and multi-domain comfort analysis through portable sensing techniques and citizens' science. Energy and Buildings 260, 111918.
45. Trájer, A. J., Sebestyén, V., Domokos, E., & Abonyi, J. (2022). Indicators for climate change-driven urban health impact assessment. Journal of Environmental Management 323, 116165.
46. Hou, J., Wang, Y., Zhou, D., & Gao, Z. (2022). Environmental Effects from Pocket Park Design According to District Planning Patterns—Cases from Xi'an, China. Atmosphere 13, 300.
47. Ma, D., Wang, Y., Zhou, D., & Zhu, Z. (2023). Cooling effect of the pocket park in the built-up block of a city: a case study in Xi'an, China. Environmental Science and Pollution Research 30, 23135–23154.

48. Wu, C., Li, J., Wang, C., Song, C., Haase, D., Breuste, J., & Finka, M. (2021). Estimating the Cooling Effect of Pocket Green Space in High Density Urban Areas in Shanghai, China. Frontiers in Environmental Science 9, 657969.
49. Ibrahim, M. S. (2021). Mitigation strategies of the urban heat island over Greater Cairo Metropolitan Area, Egypt utilizing ENVI-met model. Catrina: The International Journal of Environmental Sciences 24, 35-47.
50. Huang, J., Hao, T., Wang, Y., & Jones, P. (2022). A street-scale simulation model for the cooling performance of urban greenery: Evidence from a high-density city. Sustainable Cities and Society 82, 103908.
51. Lu, J., Li, Q., Zeng, L., Chen, J., Liu, G., Li, Y., Li, W., & Huang, K. (2017). A micro-climatic study on cooling effect of an urban park in a hot and humid climate. Sustainable Cities and Society 32, 513–522.
52. Chibuike, E. M., Ibukun, A. O., Abbas, A., & Kunda, J. J. (2018). Assessment of green parks cooling effect on Abuja urban microclimate using geospatial techniques. Remote Sensing Applications: Society and Environment 11, 11–21.
53. Zhang, Z., Lv, Y., & Pan, H. (2013). Cooling and humidifying effect of plant communities in subtropical urban parks. Urban Forestry & Urban Greening 12, 323–329.
54. Shi, M., Chen, M., Jia, W., Du, C., & Wang, Y. (2023). Cooling effect and cooling accessibility of urban parks during hot summers in China's largest sustainability experiment. Sustainable Cities and Society 93, 104519.
55. Du, C., Jia, W., Chen, M., Yan, L., & Wang, K. (2022). How can urban parks be planned to maximize cooling effect in hot extremes? Linking maximum and accumulative perspectives. Journal of Environmental Management 317, 115346.
56. Peng, J., Dan, Y., Qiao, R., Liu, Y., Dong, J., & Wu, J. (2021). How to quantify the cooling effect of urban parks? Linking maximum and accumulation perspectives. Remote Sensing of Environment 252, 112135.
57. Yang, A.-S., Juan, Y.-H., Wen, C.-Y., & Chang, C.-J. (2017). Numerical simulation of cooling effect of vegetation enhancement in a subtropical urban park. Applied Energy 192, 178–200.
58. Chen, M., Jia, W., Yan, L., Du, C., & Wang, K. (2022). Quantification and mapping cooling effect and its accessibility of urban parks in an extreme heat event in a megacity. Journal of Cleaner Production 334, 130252.
59. Das, M., Das, A., & Momin, S. (2022). Quantifying the cooling effect of urban green space: A case from urban parks in a tropical mega metropolitan area (India). Sustainable Cities and Society 87, 104062.
60. Zhou, Y., Zhao, H., Mao, S., Zhang, G., Jin, Y., Luo, Y., Huo, W., Pan, Z., An, P., & Lun, F. (2022). Studies on urban park cooling effects and their driving factors in China: Considering 276 cities under different climate zones. Building and Environment 222, 109441.
61. Algretawee, H. (2022). The effect of graduated urban park size on park cooling island and distance relative to land surface temperature (LST). Urban Climate 45, 101255.
62. Qiu, K., & Jia, B. (2020). The roles of landscape both inside the park and the surroundings in park cooling effect. Sustainable Cities and Society 52, 101864.
63. Li, Y., Fan, S., Li, K., Zhang, Y., Kong, L., Xie, Y., & Dong, L. (2021). Large urban parks summertime cool and wet island intensity and its influencing factors in Beijing, China. Urban Forestry and Urban Greening 65.

64. Gao, Z., Zaitchik, B. F., Hou, Y., & Chen, W. (2022). Toward park design optimization to mitigate the urban heat Island: Assessment of the cooling effect in five U.S. cities. Sustainable Cities and Society 81, 103870.
65. Yang, A.S., Juan, Y.H., Wen, C.Y., Chang, C.J. (2017). Numerical simulation of cooling effect of vegetation enhancement in a subtropical urban park. Applied energy 192, 178-200.
66. Şimşek, Ç. K., Serter, G., & Ödül, H. (2022). A Study on the Cooling Capacities of Urban Parks and Their Interactions with the Surrounding Urban Patterns. Applied Spatial Analysis and Policy 15, 1287–1317.
67. Li, Y., Fan, S., Li, K., Zhang, Y., & Dong, L. (2021). Microclimate in an urban park and its influencing factors: a case study of Tiantan Park in Beijing, China. Urban Ecosystems 24, 767-778.
68. Cheung, P. K., Jim, C. Y., & Siu, C. T. (2021). Effects of urban park design features on summer air temperature and humidity in compact-city milieu. Applied Geography 129, 102439.
69. Algretawee, H., Rayburg, S., & Neave, M. (2019). Estimating the effect of park proximity to the central of Melbourne city on Urban Heat Island (UHI) relative to Land Surface Temperature (LST). Ecological Engineering 138, 374–390.
70. Yan, H., Wu, F., & Dong, L. (2018). Influence of a large urban park on the local urban thermal environment. Science of the Total Environment 662, 882–891.
71. Chang, N., & Li, D. W. (2016). A study of the temperature-humidity effect and luminous environment design for urban green space. Chemical Engineering Transactions 51, 103–108.
72. Irmak, M. A., Yilmaz, S., & Dursun, D. (2017). Effect of different pavements on human thermal comfort conditions. Atmósfera 30, 355–366.
73. Su, X. (2018). Study on Humidification and Cooling Effect of Garden Plants. 4th International Conference on Education, Management and Information Technology (ICEMIT 2018).
74. Yilmaz, S., Mutlu, E., & Yilmaz, H. (2018). Alternative scenarios for ecological urbanizations using ENVI-met model. Environmental Science and Pollution Research 25, 26307–26321.
75. Yilmaz, S., Irmak, M. A., & Qaid, A. (2022). Assessing the effects of different urban landscapes and built environment patterns on thermal comfort and air pollution in Erzurum city, Turkey. Building and Environment 219, 109210.
76. Ghani, S., ElBialy, E. A., Bakochristou, F., Gamaledin, S. M. A., Rashwan, M. M., & Hughes, B. (2017). Thermal performance of stadium's Field of Play in hot climates. Energy and Buildings 139, 702–718.
77. Kuchcik, M., Dudek, W., Błażejczyk, K., Milewski, P., & Błażejczyk, A. (2016). Two faces to the greenery on housing estates–mitigating climate but aggravating allergy. A Warsaw case study. Urban Forestry & Urban Greening 16, 170–181.
78. Vanos, J. K., Middel, A., McKercher, G. R., Kuras, E. R., & Ruddell, B. L. (2016). Hot playgrounds and children's health: A multiscale analysis of surface temperatures in Arizona, USA. Landscape and Urban Planning 146, 29–42.
79. Nguyen, T. T., Eslick, H., Barber, P., Harper, R., & Dell, B. (2022). Cooling effects of urban vegetation: The role of golf courses. Remote Sensing 14, 4351.
80. Zölch, T., Maderspacher, J., Wamsler, C., & Pauleit, S. (2016). Using green infrastructure for urban climate-proofing: An evaluation of heat mitigation measures at the micro-scale. Urban Forestry & Urban Greening 20, 305–316.

81. Sallay, Á., Mikházi, Z., Gecséné Tar, I., & Takács, K. (2022). Cemeteries as a Part of Green Infrastructure and Tourism. Sustainability 14, 2918.
82. Rost, A. T., Liste, V., Seidel, C., Matscheroth, L., Otto, M., Meier, F., & Fenner, D. (2020). How cool are allotment gardens? A case study of nocturnal air temperature differences in Berlin, Germany. Atmosphere 11, 500.
83. Hawkins, T. W., Brazel, A. J., Stefanov, W. L., Bigler, W., & Saffell, E. M. (2004). The role of rural variability in urban heat island determination for Phoenix, Arizona. Journal of Applied Meteorology and Climatology 43, 476-486.
84. Masson, V., Lion, Y., Peter, A., Pigeon, G., Buyck, J., & Brun, E. (2013). "Grand Paris": regional landscape change to adapt city to climate warming. Climatic Change 117, 769-782.
85. Basu, T., & Das, A. (2023). Urbanization induced degradation of urban green space and its association to the land surface temperature in a medium-class city in India. Sustainable Cities and Society 90, 104373.
86. Boeri, A. N. D. R. E. A., Gaspari, J. A. C. O. P. O., Gianfrate, V. A. L. E. N. T. I. N. A., & Longo, D. A. N. I. L. A. (2017). Accelerating urban transition: An approach to greening the built environment. WIT Transactions on Ecology and the Environment 223, 3-14.
87. Abdollahzadeh, N., & Biloria, N. (2021). Outdoor thermal comfort: Analyzing the impact of urban configurations on the thermal performance of street canyons in the humid subtropical climate of Sydney. Frontiers of Architectural Research 10, 394–409.
88. Cai, Y., Li, C., Ye, L., Xiao, L., Gao, X., Mo, L., Du, H., Zhou, Y., & Zhou, G. (2022). Effect of the roadside tree canopy structure and the surrounding on the daytime urban air temperature in summer. Agricultural and Forest Meteorology 316, 108850.
89. Morabito, M., Crisci, A., Guerri, G., Messeri, A., Congedo, L., & Munafò, M. (2021). Surface urban heat islands in Italian metropolitan cities: Tree cover and impervious surface influences. Science of The Total Environment 751, 142334.
90. Napoli, M., Massetti, L., Brandani, G., Petralli, M., & Orlandini, S. (2016). Modeling Tree Shade Effect on Urban Ground Surface Temperature. Journal of Environmental Quality 45, 146–156.
91. Xi, C., Wang, D., & Cao, S.-J. (2023). Impacts of trees-grass area ratio on thermal environment, energy saving, and carbon benefits. Urban Climate 47, 101393.
92. Zeeshan, M., Ali, Z., Sajid, M., Ali, M., & Usman, M. (2022). Modelling the cooling effectiveness of street trees with actual canopy drag and real transpiration rate under representative climatic conditions. Journal of Building Performance Simulation, 1–14.
93. Abu Ali, M., Alawadi, K., & Khanal, A. (2021). The role of green infrastructure in enhancing microclimate conditions: A case study of a low-rise neighborhood in Abu Dhabi. Sustainability 13, 4260.
94. Marando, F., Salvatori, E., Sebastiani, A., Fusaro, L., & Manes, F. (2019). Regulating Ecosystem Services and Green Infrastructure: assessment of Urban Heat Island effect mitigation in the municipality of Rome, Italy. Ecological Modelling 392, 92–102.
95. Zeeshan, M., Ali, Z., Ud Din, E. (2023). Thermal performance prediction of street trees inside isolated open spaces–evaluations from real scale retrofitting project. Journal of Building Performance Simulation 16, 381-397.
96. Wang, Y., & Akbari, H. (2016). The effects of street tree planting on Urban Heat Island mitigation in Montreal. Sustainable Cities and Society 27, 122–128.

97. Lachapelle, J. A., Scott Krayenhoff, E., Middel, A., Coseo, P., & Warland, J. (2023). Maximizing the pedestrian radiative cooling benefit per street tree. Landscape and Urban Planning 230, 104608.
98. Segura, R., Krayenhoff, E. S., Martilli, A., Badia, A., Estruch, C., Ventura, S., & Villalba, G. (2022). How do street trees affect urban temperatures and radiation exchange? Observations and numerical evaluation in a highly compact city. Urban Climate 46, 101288.
99. Miao, C., Li, P., Huang, Y., Sun, Y., Chen, W., & Yu, S. (2023). Coupling outdoor air quality with thermal comfort in the presence of street trees: a pilot investigation in Shenyang, Northeast China. Journal of Forestry Research 34, 831-839.
100. Geletič, J., Lehnert, M., Resler, J., Krč, P., Middel, A., Krayenhoff, E. S., & Krüger, E. (2022). High-fidelity simulation of the effects of street trees, green roofs and green walls on the distribution of thermal exposure in Prague-Dejvice. Building and Environment 223, 109484.
101. Mussetti, G., Brunner, D., Henne, S., Allegrini, J., Scott Krayenhoff, E., Schubert, S., Feigenwinter, C., Vogt, R., Wicki, A., & Carmeliet, J. (2020). COSMO-BEP-Tree v1.0: A coupled urban climate model with explicit representation of street trees. Geoscientific Model Development 13, 1685–1710.
102. Vailshery, L. S., Jaganmohan, M., & Nagendra, H. (2013). Effect of street trees on microclimate and air pollution in a tropical city. Urban Forestry & Urban Greening 12, 408–415.
103. Gillner, S., Vogt, J., Tharang, A., Dettmann, S., & Roloff, A. (2015). Role of street trees in mitigating effects of heat and drought at highly sealed urban sites. Landscape and Urban Planning 143, 33–42.
104. Coutts, A. M., White, E. C., Tapper, N. J., Beringer, J., & Livesley, S. J. (2016). Temperature and human thermal comfort effects of street trees across three contrasting street canyon environments. Theoretical and Applied Climatology 124, 55–68.
105. Sanusi, R., Johnstone, D., May, P., & Livesley, S. J. (2016). Street Orientation and Side of the Street Greatly Influence the Microclimatic Benefits Street Trees Can Provide in Summer. Journal of Environmental Quality 45, 167–174.
106. Aminipouri, M., Knudby, A. J., Krayenhoff, E. S., Zickfeld, K., & Middel, A. (2019). Modelling the impact of increased street tree cover on mean radiant temperature across Vancouver's local climate zones. Urban Forestry & Urban Greening 39, 9–17.
107. Kusaka, H., Nakamura, Y., & Asano, Y. (2022). UV Parasol, Dry-Mist Spraying, and Street Trees as Tools for Heat Stress Mitigation. Journal of the Meteorological Society of Japan. Ser. II 100, 677–685.
108. Cho, H. (2022). Effects of Road Components and Roadside Vegetation on Temperature Reduction in Seoul Considering Air, Wet-Bulb Globe, and Surface Temperatures. Sustainability 14, 16663.
109. Huang, K. T., & Li, Y. J. (2017). Impact of street canyon typology on building's peak cooling energy demand: A parametric analysis using orthogonal experiment. Energy and Buildings 154, 448-464.
110. Stojanovic, N., Vasiljevic, N., Mešicek, M., & Lisica, A. (2018). The influence of roadside green spaces on thermal conditions in the urban environment. Journal of Architectural and Planning Research 35, 165-178.
111. Zaki, S. A., Toh, H. J., Yakub, F., Mohd Saudi, A. S., Ardila-Rey, J. A., & Muhammad-Sukki, F. (2020). Effects of roadside trees and road orientation on thermal environment in a tropical city. Sustainability 12, 1053.

112. Zheng, X., Chen, L., & Yang, J. (2023). Simulation framework for early design guidance of urban streets to improve outdoor thermal comfort and building energy efficiency in summer. Building and Environment 228, 109815.
113. Žižlavská, N., Mikita, T., & Patočka, Z. (2021). The Effects of Roadside Woody Vegetation on the Surface Temperature of Cycle Paths. Land 10, 483.
114. Adams, M. P., & Smith, P. L. (2014). A systematic approach to model the influence of the type and density of vegetation cover on urban heat using remote sensing. Landscape and Urban Planning 132, 47-54.
115. Yonghong, S., Fandi, L., Gaofeng, Z., Zhang, K., & Qi, Z. (2023). The biophysical climate mitigation potential of riparian forest ecosystems in arid Northwest China. Science of The Total Environment 862, 160856.
116. Tsai, C. W., Young, T., Warren, P. H., & Maltby, L. (2017). Riparian thermal conditions across a mixed rural and urban landscape. Applied geography 87, 106-114.
117. Dugdale, S.J., Malcolm, I.A., Hannah, D.M. (2019). Drone-based Structure-from-Motion provides accurate forest canopy data to assess shading effects in river temperature models. Science of the Total Environment 678, 326-340.
118. Zheng, X., Zhang, N., & Wang, X. (2022). Development of a modified thermal humidity index and its application to human thermal comfort of urban vegetation patches. Ecosystem Health and Sustainability 8, 2130095.
119. Zhang, J., & Hu, D. (2022). Research of practical heat mitigation strategies in a residential district of Beijing, North China. Urban Climate 46, 101314.
120. Peluso, P., Persichetti, G., & Moretti, L. (2022). Effectiveness of Road Cool Pavements, Greenery, and Canopies to Reduce the Urban Heat Island Effects. Sustainability 14, 16027.
121. Del Serrone, G., Peluso, P., & Moretti, L. (2022). Evaluation of Microclimate Benefits Due to Cool Pavements and Green Infrastructures on Urban Heat Islands. Atmosphere 13, 1586.
122. Zou, Z., Yang, Y., Qiu, G. Y. (2019). Quantifying the evapotranspiration rate and its cooling effects of urban hedges based on three-temperature model and infrared remote sensing. Remote Sensing 11, 202.
123. Yoshida, A., Hayashi, D., Shimazaki, Y., & Kinoshita, S. (2019). Evaluation of thermal sensation in various outdoor radiation environments. Architectural Science Review 62, 261–270.
124. Wang, X., Li, H., & Sodoudi, S. (2022). The effectiveness of cool and green roofs in mitigating urban heat island and improving human thermal comfort. Building and Environment 217, 109082.
125. Cortes, A., Rejuso, A. J., Santos, J. A., & Blanco, A. (2022). Evaluating mitigation strategies for urban heat island in Mandaue City using ENVI-met. Journal of Urban Management 11, 97–106.
126. Fleck, R., Gill, R. L., Saadeh, S., Pettit, T., Wooster, E., Torpy, F., & Irga, P. (2022). Urban green roofs to manage rooftop microclimates: A case study from Sydney, Australia. Building and Environment 209, 108673.
127. Dong, J., Lin, M., Zuo, J., Lin, T., Liu, J., Sun, C., & Luo, J. (2020). Quantitative study on the cooling effect of green roofs in a high-density urban Area—A case study of Xiamen, China. Journal of Cleaner Production 255, 120152.
128. Kostadinović, D., Jovanović, M., Bakić, V., Stepanić, N., & Todorović, M. (2022). Experimental investigation of summer thermal performance of the green roof system with mineral wool substrate. Building and Environment 217, 109061.

129. Peng, L. L. H., Yang, X., He, Y., Hu, Z., Xu, T., Jiang, Z., & Yao, L. (2019). Thermal and energy performance of two distinct green roofs: Temporal pattern and underlying factors in a subtropical climate. Energy and Buildings 185, 247–258.
130. Lee, L. S. H., & Jim, C. Y. (2019). Urban woodland on intensive green roof improved outdoor thermal comfort in subtropical summer. International Journal of Biometeorology 63, 895–909.
131. Köhler, M., & Kaiser, D. (2019). Evidence of the climate mitigation effect of green roofs-A 20-year weather study on an Extensive Green Roof (EGR) in Northeast Germany. Buildings 9, 157.
132. Park, J., Kim, J.-H., Dvorak, B., & Lee, D. K. (2018). The role of green roofs on microclimate mitigation effect to local climates in summer. International Journal of Environmental Research 12, 671–679.
133. Lynn, B. H., & Lynn, I. M. (2020). The impact of cool and green roofs on summertime temperatures in the cities of Jerusalem and Tel Aviv. Science of The Total Environment 743, 140568.
134. Chen, X., Jeong, S., Zheng, Y., Park, H., Park, C. E., Joo, J., Choi, W., Chen, X., & Zheng, C. (2020). Evaluation of Different Roof Materials for the Mitigation of Urban Warming in a Subtropical Monsoon Climate. Journal of Geophysical Research: Atmospheres 125, e2019JD031972.
135. Cortes, A., Rejuso, A. J., Santos, J. A., & Blanco, A. (2022). Evaluating mitigation strategies for urban heat island in Mandaue City using ENVI-met. Journal of Urban Management 11, 97–106.
136. Solcerova, A., van de Ven, F., Wang, M., Rijsdijk, M., & van de Giesen, N. (2017). Do green roofs cool the air? Building and Environment 111, 249–255.
137. Bochenek, A. D., & Klemm, K. (2020). The impact of passive green technologies on the microclimate of historic urban structures: The case study of Lodz. Atmosphere 11, 974.
138. Chen, B., Wang, W., You, Y., Zhu, W., Dong, Y., Xu, Y., Chang, M., & Wang, X. (2023). Influence of rooftop mitigation strategies on the thermal environment in a subtropical city. Urban Climate 49, 101450.
139. Elnabawi, M. H., & Saber, E. (2023). A numerical study of cool and green roof strategies on indoor energy saving and outdoor cooling impact at pedestrian level in a hot arid climate. Journal of Building Performance Simulation 16, 72–89.
140. Zuo, J., Ma, J., Lin, T., Dong, J., Lin, M., & Luo, J. (2022). Quantitative valuation of green roofs' cooling effects under different urban spatial forms in high-density urban areas. Building and Environment 222, 109367.
141. Iaria, J., & Susca, T. (2022). Analytic Hierarchy Processes (AHP) evaluation of green roof- and green wall-based UHI mitigation strategies via ENVI-met simulations. Urban Climate 46, 101293.
142. Fleck, R., Gill, R. L., Saadeh, S., Pettit, T., Wooster, E., Torpy, F., & Irga, P. (2022). Urban green roofs to manage rooftop microclimates: A case study from Sydney, Australia. Building and Environment 209, 108673.
143. Robbiati, F. O., Cáceres, N., Hick, E. C., Suarez, M., Soto, S., Barea, G., Matoff, E., Galetto, L., & Imhof, L. (2022). Vegetative and thermal performance of an extensive vegetated roof located in the urban heat island of a semiarid region. Building and Environment 212, 108791.

144. Liao, J., Tan, X., & Li, J. (2021). Evaluating the vertical cooling performances of urban vegetation scenarios in a residential environment. Journal of Building Engineering 39, 102313.
145. Cameron, R. W., Taylor, J. E., & Emmett, M. R. (2014). What's ‘cool’in the world of green façades? How plant choice influences the cooling properties of green walls. Building and environment 73, 198-207.
146. Zhang, L., Deng, Z., Liang, L., Zhang, Y., Meng, Q., Wang, J., & Santamouris, M. (2019). Thermal behavior of a vertical green facade and its impact on the indoor and outdoor thermal environment. Energy and Buildings 204, 109502.
147. Liu, Y., Huang, Y., Zhang, Z., Wang, K., Luo, Y., & Cui, P. (2022). Impacts of green walls on the characteristics of thermo-flow and photochemical reaction kinetics within street canyons. Urban Forestry & Urban Greening 72, 127568.
148. Cameron, R. W., Taylor, J., & Emmett, M. (2015). A Hedera green façade–energy performance and saving under different maritime-temperate, winter weather conditions. Building and Environment 92, 111-121.
149. Jesus, M. P., Lourenço, J. M., Arce, R. M., & Macias, M. (2017). Green façades and in situ measurements of outdoor building thermal behaviour. Building and Environment 119, 11–19.
150. Lee, L. S. H, & Jim, C. Y. (2017). Subtropical summer thermal effects of wirerope climber green walls with different air-gap depths. Building and Environment 126, 1-12.
151. Feitosa, R.C., Wilkinson, S.J. (2018). Attenuating heat stress through green roof and green wall retrofit. Building and Environment 140, 11-22.
152. Li, H., Zhao, Y., Sützl, B., Kubilay, A., & Carmeliet, J. (2022). Impact of green walls on ventilation and heat removal from street canyons: Coupling of thermal and aerodynamic resistance. Building and Environment 214, 108945.
153. Geletič, J., Lehnert, M., Resler, J., Krč, P., Middel, A., Krayenhoff, E.S. and Krüger, E. (2022). High-fidelity simulation of the effects of street trees, green roofs and green walls on the distribution of thermal exposure in Prague-Dejvice. Building and Environment 223, 109484.
154. Šuklje, T., Medved, S., & Arkar, C. (2016). On detailed thermal response modeling of vertical greenery systems as cooling measure for buildings and cities in summer conditions. Energy 115, 1055-1068.
155. Medl, A., Mayr, S., Rauch, H. P., Weihs, P., & Florineth, F. (2017). Microclimatic conditions of ‘Green Walls’, a new restoration technique for steep slopes based on a steel grid construction. Ecological engineering 101, 39-45.
156. Feitosa, R. C., & Wilkinson, S. J. (2020). Small-scale experiments of seasonal heat stress attenuation through a combination of green roof and green walls. Journal of Cleaner Production 250, 119443.
157. Blanco, I., Schettini, E., & Vox, G. (2019). Predictive model of surface temperature difference between green façades and uncovered wall in Mediterranean climatic area. Applied Thermal Engineering 163, 114406.
158. Bakhshoodeh, R., Ocampo, C., & Oldham, C. (2022). Exploring the evapotranspirative cooling effect of a green façade. Sustainable Cities and Society 81, 103822.
159. Davis, M. M., Vallejo Espinosa, A. L., & Ramirez, F. R. (2019). Beyond green façades: active air-cooling vertical gardens. Smart and Sustainable Built Environment 8, 243-252.

160. Hosseinzadeh, A., Bottacin-Busolin, A., & Keshmiri, A. (2022). A parametric study on the effects of green roofs, green walls and trees on air quality, temperature and velocity. Buildings 12, 2159.
161. Djedjig, R., Bozonnet, E., & Belarbi, R. (2015). Experimental study of the urban microclimate mitigation potential of green roofs and green walls in street canyons. International Journal of Low-Carbon Technologies 10, 34-44.
162. Pragati, S., Shanthi Priya, R., Pradeepa, C., & Senthil, R. (2023). Simulation of the Energy Performance of a Building with Green Roofs and Green Walls in a Tropical Climate. Sustainability 15, 2006.
163. Lin, H., Xiao, Y., Musso, F., & Lu, Y. (2019). Green façade effects on thermal environment in transitional space: Field measurement studies and computational fluid dynamics simulations. Sustainability 11, 5691.
164. Price, A., Jones, E. C., & Jefferson, F. (2015). Vertical greenery systems as a strategy in urban heat island mitigation. Water, Air, & Soil Pollution 226, 1-11.
165. Lee, L. S. H, & Jim, C. Y. (2020). Multidimensional analysis of temporal and layered microclimatic behavior of subtropical climber green walls in summer. Urban Ecosystems 23, 389-402.
166. Lin, H., Musso, F., & Xiao, Y. (2018). Shading Effect and Heat Reflection of the Green Façade. PLEA 2018: Smart and Healthy Within the Two-Degree Limit, 931.
167. Li, J., Zheng, B., Shen, W., Xiang, Y., Chen, X., & Qi, Z. (2019). Cooling and energy-saving performance of different green wall design: A simulation study of a block. Energies 12, 2912.
168. Peng, L.L., Yang, X., He, Y., Hu, Z., Xu, T., Jiang, Z., Yao, L. (2019). Thermal and energy performance of two distinct green roofs: Temporal pattern and underlying factors in a subtropical climate. Energy and Buildings 185, 247-258.
169. Shen, Z. (2022). Green Roof Design of Residential Area Based on Sponge City Theory. Wireless Communications and Mobile Computing, 2022.
170. AbdulBaqi, F. K. (2022). The effect of afforestation and green roofs techniques on thermal reduction in Duhok city. Trees, Forests and People 8, 100267.
171. Kim, J., Lee, S. Y., & Kang, J. (2020). Temperature reduction effects of rooftop garden arrangements: A case study of Seoul National University. Sustainability 12, 6032.
172. Lee, L. S. H., & Jim, C. Y. (2018). Thermal-cooling performance of subtropical green roof with deep substrate and woodland vegetation. Ecological Engineering 119, 8–18.
173. Tan, C. L., Tan, P. Y., Wong, N. H., Takasuna, H., Kudo, T., Takemasa, Y., Lim, C. V. J., & Chua, H. X. V. (2017). Impact of soil and water retention characteristics on green roof thermal performance. Energy and Buildings 152, 830–842.
174. Tan, C. L., Wong, N. H., Jusuf, S. K., & Chiam, Z. Q. (2015). Impact of plant evapotranspiration rate and shrub albedo on temperature reduction in the tropical outdoor environment. Building and Environment 94, 206–217.
175. Watanabe, S., Nagano, K., Ishii, J., & Horikoshi, T. (2014). Evaluation of outdoor thermal comfort in sunlight, building shade, and pergola shade during summer in a humid subtropical region. Building and Environment 82, 556–565.
176. Katsoulas, N., Antoniadis, D., Tsirogiannis, I. L., Labraki, E., Bartzanas, T., & Kittas, C. (2017). Microclimatic effects of planted hydroponic structures in urban environment: measurements and simulations. International Journal of Biometeorology 61, 943–956.

177. Chàfer, M., Pisello, A. L., Piselli, C., & Cabeza, L. F. (2020). Greenery system for cooling down outdoor spaces: Results of an experimental study. Sustainability 12, 5888.
178. Kong, H., Choi, N., & Park, S. (2021). Thermal environment analysis of landscape parameters of an urban park in summer - A case study in Suwon, Republic of Korea. Urban Forestry and Urban Greening 65, 127377.
179. Teichmann, F., Horvath, A., Luisser, M. and Korjenic, A. (2022). The Impact of Small-Scale Greening on the Local Microclimate—A Case Study at Two School Buildings in Vienna. Sustainability 14, 13089.
180. Fini, A., Frangi, P., Mori, J., Donzelli, D., & Ferrini, F. (2017). Nature based solutions to mitigate soil sealing in urban areas: Results from a 4-year study comparing permeable, porous, and impermeable pavements. Environmental research 156, 443-454.
181. Kousis, I., Fabiani, C., & Pisello, A. L. (2022). Could a bio-resin and transparent pavement improve the urban environment? An in field thermo-optical investigation and life-cycle assessment. Sustainable Cities and Society 79, 103597.
182. Lu, R., Jiang, W., Xiao, J., Xing, C., Ruan, C., Li, Y., & Wu, W. (2022). Temperature characteristics of permeable asphalt pavement: Field research. Construction and Building Materials 332, 127379.
183. Moretti, L., Cantisani, G., Carpiceci, M., D'Andrea, A., Del Serrone, G., Di Mascio, P., & Loprencipe, G. (2021). Effect of sampietrini pavers on urban heat islands. International journal of environmental research and public health 18, 13108.
184. Wang, J., Meng, Q., Tan, K., Zhang, L., & Zhang, Y. (2018). Experimental investigation on the influence of evaporative cooling of permeable pavements on outdoor thermal environment. Building and Environment 140, 184-193.
185. Wang, X., Wang, X., Chen, Y., & Berlyn, G. P. (2019). Photosynthetic parameters of urban greening trees growing on paved land. iForest-Biogeosciences and Forestry 12, 403.
186. Yang, W., Wang, Z., & Zhao, X. (2015). Experimental investigation of the thermal isolation and evaporative cooling effects of an exposed shallow-water-reserved roof under the sub-tropical climatic condition. Sustainable Cities and Society 14, 293-304.
187. An, K. J., Lam, Y. F., Hao, S., Morakinyo, T. E., & Furumai, H. (2015). Multi-purpose rainwater harvesting for water resource recovery and the cooling effect. Water Research 86, 116-121.
188. Buzzard, V., Gil-Loaiza, J., Grachet, N. G., Talkington, H., Youngerman, C., Tfaily, M. M., & Meredith, L. K. (2021). Green infrastructure influences soil health: biological divergence one year after installation. Science of The Total Environment 801, 149644.
189. Kasprzyk, M., Szpakowski, W., Poznańska, E., Boogaard, F. C., Bobkowska, K., & Gajewska, M. (2022). Technical solutions and benefits of introducing rain gardens–Gdańsk case study. Science of The Total Environment 835, 155487.
190. Bai, J., Lu, Q., Zhao, Q., Wang, J., & Ouyang, H. (2013). Effects of alpine wetland landscapes on regional climate on the Zoige Plateau of China. Advances in Meteorology, 2013.
191. Cai, Z., Guldmann, J. M., Tang, Y., & Han, G. (2022). Does city-water layout matter? Comparing the cooling effects of water bodies across 34 Chinese megacities. Journal of Environmental Management 324, 116263.
192. Pucher, B., Zluwa, I., Spörl, P., Pitha, U., & Langergraber, G. (2022). Evaluation of the multifunctionality of a vertical greening system using different irrigation strategies on

cooling, plant development and greywater use. Science of The Total Environment 849, 157842.
193. Ruiz-Aviles, V., Brazel, A., Davis, J. M., & Pijawka, D. (2020). Mitigation of urban heat island effects through “green infrastructure”: integrated design of constructed wetlands and neighborhood development. Urban Science 4, 78.
194. Shahjahan, A. T. M., Ahmed, K. S., & Said, I. B. (2020). Study on Riparian Shading Envelope for Wetlands to Create Desirable Urban Bioclimates. Atmosphere 11, 1348.
195. Şimşek, Ç. K., & Ödül, H. (2018). Investigation of the effects of wetlands on micro-climate. Applied geography 97, 48-60.
196. Sun, R., Chen, A., Chen, L., & Lü, Y. (2012). Cooling effects of wetlands in an urban region: The case of Beijing. Ecological Indicators 20, 57-64.
197. Sušnik, J., Masia, S., Kravčík, M., Pokorný, J., & Hesslerová, P. (2022). Costs and benefits of landscape-based water retention measures as nature-based solutions to mitigating climate impacts in eastern Germany, Czech Republic, and Slovakia. Land Degradation & Development 33, 3074-3087.
198. Triyuly, W., Triyadi, S., & Wonorahardjo, S. (2021). Synergising the thermal behaviour of water bodies within thermal environment of wetland settlements. International Journal of Energy and Environmental Engineering 12, 55-68.
199. Wu, S., Yang, H., Luo, P., Luo, C., Li, H., Liu, M., Ruan, Y., Zhang, S., Xiang, P., Jia, H., & Cheng, Y. (2021). The effects of the cooling efficiency of urban wetlands in an inland megacity: A case study of Chengdu, Southwest China. Building and Environment 204, 108128.
200. Xu, H., Chen, H., Zhou, X., Wu, Y., & Liu, Y. (2020). Research on the relationship between urban morphology and air temperature based on mobile measurement: A case study in Wuhan, China. Urban Climate 34, 100671.
201. Zhang, W., Jiang, J., & Zhu, Y. (2015). Change in urban wetlands and their cold island effects in response to rapid urbanization. Chinese Geographical Science 25, 462-471.
202. Wenguang, Z., Wenjuan, W., Guanglei, H., Chao, G., Ming, J., & Xianguo, L. (2020). Cooling effects of different wetlands in semi-arid rural region of Northeast China. Theoretical and Applied Climatology 141, 31-41.
203. Zhang, Z., Chen, F., Barlage, M., Bortolotti, L. E., Famiglietti, J., Li, Z., Ma. X., & Li, Y. (2022). Cooling effects revealed by modeling of wetlands and land-atmosphere interactions. Water Resources Research 58, e2021WR030573.
204. Sun, R., Chen, A., Chen, L., Lü, Y. (2012). Cooling effects of wetlands in an urban region: The case of Beijing. Ecological Indicators 20, 57-64.
205. Le Phuc, C. L., Nguyen, H. S., Dao Dinh, C., Tran, N. B., Pham, Q. B., & Nguyen, X. C. (2022). Cooling island effect of urban lakes in hot waves under foehn and climate change. Theoretical and Applied Climatology 149, 817-830.
206. Theeuwes, N. E., Solcerova, A., & Steeneveld, G. J. (2013). Modeling the influence of open water surfaces on the summertime temperature and thermal comfort in the city. Journal of Geophysical Research: Atmospheres 118, 8881-8896.
207. Xu, H., Chen, H., Zhou, X., Wu, Y., Liu, Y. (2020). Research on the relationship between urban morphology and air temperature based on mobile measurement: A case study in Wuhan, China. Urban Climate 34, 100671.
208. Yang, S. J., Ran, G. P., Zhang, W., & Wang, Z. H. (2020). The cooling effect of an urban lake landscape on the urban heat island: a case study in Jinan, China. Applied Ecology & Environmental Research 18, 2197-2211.

209. Masiero, É., & de Souza, L. C. L. (2016). Improving urban thermal profile with trees and water features. Proceedings of the Institution of Civil Engineers-Urban Design and Planning 169, 66-77.
210. Novo, A. V., Bayon, J. R., Castro-Fresno, D., & Rodriguez-Hernandez, J. (2013). Temperature performance of different pervious pavements: rainwater harvesting for energy recovery purposes. Water resources management 27, 5003-5016.
211. Novo, A. V., Bayon, J. R., Castro-Fresno, D., & Rodriguez-Hernandez, J. (2013). Monitoring and evaluation of the thermal behavior of permeable pavements for energy recovery purposes in an experimental parking lot: Preliminary results. Journal of Energy Engineering 139, 230-237.
212. Dandou, A., Papangelis, G., Kontos, T., Santamouris, M., & Tombrou, M. (2021). On the cooling potential of urban heating mitigation technologies in a coastal temperate city. Landscape and Urban Planning 212, 104106.
213. Zhou, X., Okaze, T., Ren, C., Cai, M., Ishida, Y., Watanabe, H., & Mochida, A. (2020). Evaluation of urban heat islands using local climate zones and the influence of sea-land breeze. Sustainable Cities and Society 55, 102060.
214. Zhou, Y., Guan, H., Gharib, S., Batelaan, O., & Simmons, C. T. (2021). Cooling power of sea breezes and its inland penetration in dry-summer Adelaide, Australia. Atmospheric Research 250, 105409.
215. Zhou, Y., Guan, H., Huang, C., Fan, L., Gharib, S., Batelaan, O., & Simmons, C. (2019). Sea breeze cooling capacity and its influencing factors in a coastal city. Building and Environment 166, 106408.
216. Zhu, D., Zhou, X., & Cheng, W. (2022). Water effects on urban heat islands in summer using WRF-UCM with gridded urban canopy parameters — A case study of Wuhan. Building and Environment 225, 109528.
217. Sahani, J., P, Kumar., SE., Debele. (2023). Efficacy assessment of green-blue nature-based solutions against environmental heat mitigation. Environment International. Under review.
218. Fung, C. K., & Jim, C. Y. (2017). Assessing the cooling effects of different vegetation settings in a Hong Kong golf course. Procedia Environmental Sciences 37, 626-636.
219. Fung, C. K., & Jim, C. Y. (2019). Microclimatic resilience of subtropical woodlands and urban-forest benefits. Urban Forestry & Urban Greening 42, 100-112.
220. Yonghong, S., Fandi, L., Gaofeng, Z., Zhang, K., Qi, Z. (2023). The biophysical climate mitigation potential of riparian forest ecosystems in arid Northwest China. Science of The Total Environment 862, 160856.
221. Chang, N., Li, D.W. (2016). A study of the temperature-humidity effect and luminous environment design for urban green space. Chemical Engineering Transactions 51, 103-108.
222. Liu, W., Zhao, H., Sun, S., Xu, X., Huang, T., & Zhu, J. (2022). Green space cooling effect and contribution to mitigate heat island effect of surrounding communities in beijing metropolitan area. Frontiers in Public Health 10, 870403.
223. Ma, Y., Zhao, M., Li, J., Wang, J., & Hu, L. (2021). Cooling effect of different land cover types: a case study in Xi'an and Xianyang, China. Sustainability 13, 1099.
224. Fung, C.K., Jim, C.Y. (2017). Assessing the cooling effects of different vegetation settings in a Hong Kong golf course. Procedia Environmental Sciences 37, 626-636.
225. Adams, M.P., Smith, P.L. (2014). A systematic approach to model the influence of the type and density of vegetation cover on urban heat using remote sensing. Landscape and Urban Planning 132, 47-54.

226. Callard, S. L., Newnham, R. M., Vandergoes, M. J., Alloway, B. V., & Smith, C. (2013). The vegetation and climate during the Last Glacial cold period, northern South Island, New Zealand. Quaternary Science Reviews 74, 230-244.
227. Amani-Beni, M., Zhang, B., Xie, G. D., & Shi, Y. (2019). Impacts of urban green landscape patterns on land surface temperature: Evidence from the adjacent area of Olympic Forest Park of Beijing, China. Sustainability 11, 513.
228. Liu, W., Zhao, H., Sun, S., Xu, X., Huang, T., Zhu, J. (2022). Green space cooling effect and contribution to mitigate heat island effect of surrounding communities in beijing metropolitan area. Frontiers in Public Health 10, 870403.
229. Jain, S., Sannigrahi, S., Sen, S., Bhatt, S., Chakraborti, S., & Rahmat, S. (2020). Urban heat island intensity and its mitigation strategies in the fast-growing urban area. Journal of Urban Management 9, 54-66.
230. Targino, A. C., Coraiola, G. C., & Krecl, P. (2019). Green or blue spaces? Assessment of the effectiveness and costs to mitigate the urban heat island in a Latin American city. Theoretical and Applied Climatology 136, 971-984.
231. Cheung, P. K., & Jim, C. Y. (2019). Differential cooling effects of landscape parameters in humid-subtropical urban parks. Landscape and urban planning 192, 103651.
232. Dai, Z., Guldmann, J. M., & Hu, Y. (2019). Thermal impacts of greenery, water, and impervious structures in Beijing's Olympic area: A spatial regression approach. Ecological indicators 97, 77-88.
233. Sherman, M.H., & Ford, J. (2014). Stakeholder engagement in adaptation interventions: an evaluation of projects in developing nations. Climate Policy 14, 417-441.
234. O'Brien, R.M., Phelan, T.J., Smith, N.M., & Smits, K.M. (2021). Remediation in developing countries: A review of previously implemented projects and analysis of stakeholder participation efforts. Critical Reviews in Environmental Science and Technology 51, 1259-1280.
235. Coutts, J., White, T., Blackett, P., Rijswijk, K., Bewsell, D., Park, N., Turner, J.A., & Botha, N. (2017). Evaluating a space for co-innovation: Practical application of nine principles for co-innovation in five innovation projects. Outlook on Agriculture 46, 99-107.
236. Curt, C., Di Maiolo, P., Schleyer-Lindenmann, A., Tricot, A., Arnaud, A., Curt, T., Parès, N., & Taillandier, F. (2022). Assessing the environmental and social co-benefits and disbenefits of natural risk management measures. Heliyon 8, e12465.
237. Ommer, J., Bucchignani, E., Leo, L.S., Kalas, M., Vranić, S., Debele, S., Kumar, P., Cloke, H.L. & Di Sabatino, S. (2022). Quantifying co-benefits and disbenefits of Nature-based Solutions targeting Disaster Risk Reduction. International Journal of Disaster Risk Reduction 75, 102966.
238. Dumitru, A., Frantzeskaki, N., & Collier, M. (2020). Identifying principles for the design of robust impact evaluation frameworks for nature-based solutions in cities. Environmental Science & Policy 112, 107-116.
239. Kumar, P., Debele, S. E., Sahani, J., Rawat, N., Marti-Cardona, B., Alfieri, S. M., Basu, B., Basu, A. S., Bowyer, P., Charizopoulos, N., Gallotti, G., Jaakko, J., Leo, L. S., Loupis, M., Menenti, M., Mickovski, S. B., Mun, S.-J., Gonzalez-Ollauri, A., Pfeiffer, J., Pilla, F., Proll, J., Rutzinger, M., Santo, M. A., Sannigrahi, S., Spyrou, C., Tuomenvirta, H., & Zieher, T. (2021a). Nature-based solutions efficiency evaluation against natural hazards: Modelling methods, advantages and limitations. Science of The Total Environment 784, 147058.

240. Davies, C., Chen, W.Y., Sanesi, G., & Lafortezza, R. (2021). The European Union roadmap for implementing nature-based solutions: A review. Environmental Science & Policy 121, 49-67.
241. European Green Deal (2021). The European Green Deal and cohesion policy. Available at: https://www.europarl.europa.eu/RegData/etudes/BRIE/2021/698058/EPRS_BRI(2021)698058_EN.pdf (retrieved on 23 June 2023).
242. Di Pirro, E., Sallustio, L., Sgrigna, G., Marchetti, M., & Lasserre, B. (2022). Strengthening the implementation of national policy agenda in urban areas to face multiple environmental stressors: Italy as a case study. Environmental Science & Policy 129, 1-11.
243. Topal, H.F., Hunt, D.V.L., & Rogers, C.D.F. (2021). Exploring urban sustainability understanding and behavior: A systematic review towards a conceptual framework. Sustainability 13, 1139.
244. Augusto, B., Roebeling, P., Rafael, S., Ferreira, J., Ascenso, A., & Bodilis, C. (2020). Short and medium-to long-term impacts of nature-based solutions on urban heat. Sustainable Cities and Society 57, 102122.
245. Frantzeskaki, N. (2019). Seven lessons for planning nature-based solutions in cities. Environmental Science & Policy 93, 101-111.
246. Cortinovis, C., Olsson, P., Boke-Olén, N., & Hedlund, K. (2022). Scaling up nature-based solutions for climate-change adaptation: Potential and benefits in three European cities. Urban Forestry & Urban Greening 67, 127450.
