# Supplementary material for: Urban heat mitigation by green and blue infrastructure: Drivers, effectiveness, and future needs
Source: Innovation (Camb). 2024 Feb 7;5(2):100588. doi: 10.1016/j.xinn.2024.100588 (PMC10909648; doi:10.1016/j.xinn.2024.100588)
Supplement: Document S2. Article plus supplemental information [file mmc2.pdf]

Urban heat mitigation by green and blue infrastructure: Drivers, effectiveness, and future needs

Prashant Kumar,[1,2,10,*] Sisay E. Debele,[1] Soheila Khalili,[1] Christos H. Halios,[3] Jeetendra Sahani,[1] Nasrin Aghamohammadi,[4,5] Maria de Fatima Andrade,[6] Maria Athanassiadou,[7] Kamaldeep Bhui,[8] Nerea Calvillo,[9] Shi-Jie Cao,[1,10] Frederic Coulon,[11] Jill L. Edmondson,[12] David Fletcher,[13] Edmilson Dias de Freitas,[6] Hai Guo,[14] Matthew C. Hort,[7] Madhusudan Katti,[15] Thomas Rodding Kjeldsen,[16] Steffen Lehmann,[17] Giuliano Maselli Locosselli,[18] Shelagh K. Malham,[19] Lidia Morawska,[1,20] Rajan Parajuli,[21] Christopher D.F. Rogers,[22] Runming Yao,[3,23] Fang Wang,[24,25] Jannis Wenk,[16] and Laurence Jones[13,26]

*Correspondence: p.kumar@surrey.ac.uk

Received: August 7, 2023; Accepted: February 5, 2024; Published Online: February 7, 2024; https://doi.org/10.1016/j.xinn.2024.100588

© 2024 The Author(s). This is an open access article under the CC BY license (http://creativecommons.org/licenses/by/4.0/).

GRAPHICAL ABSTRACT

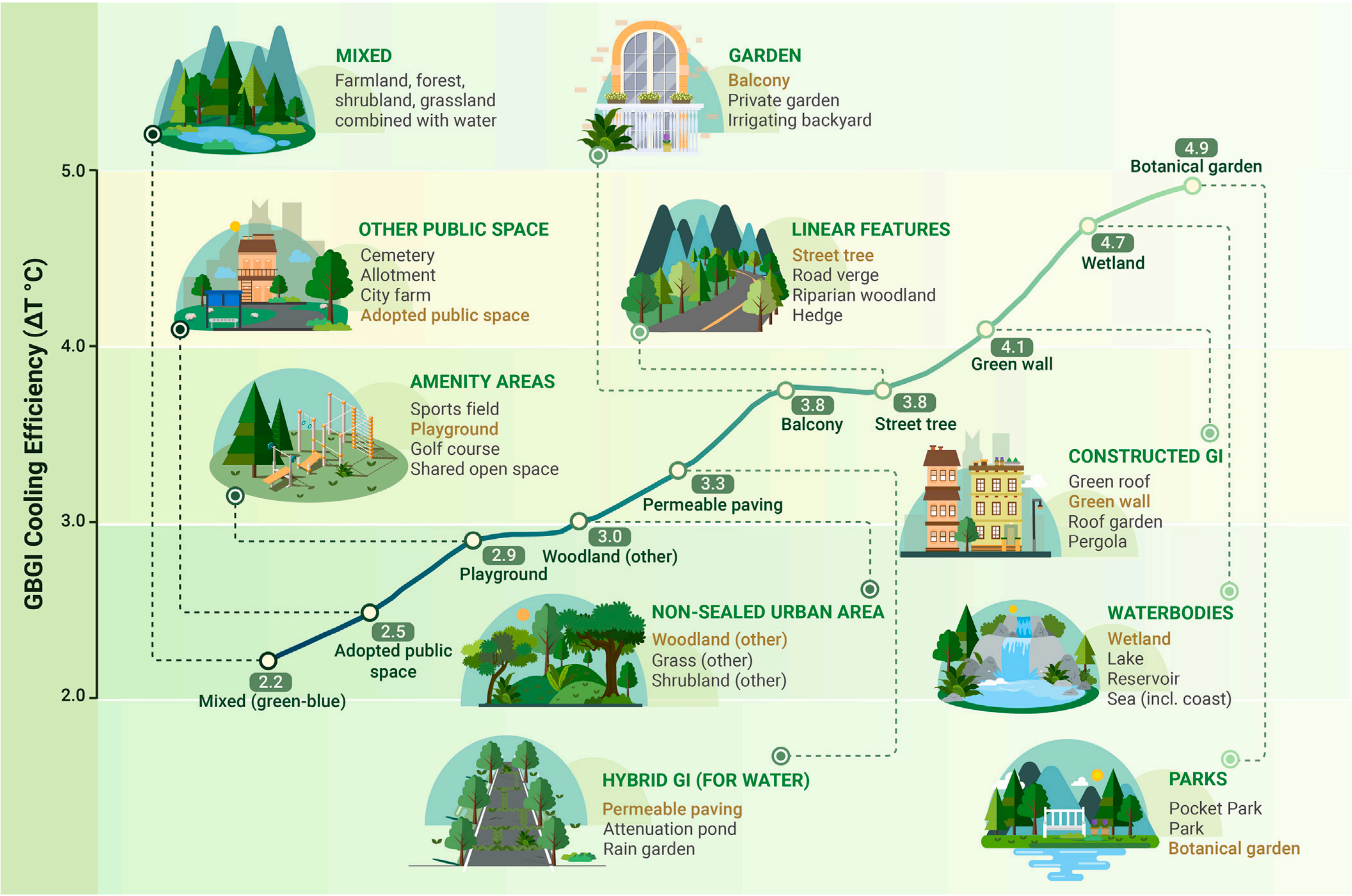

PUBLIC SUMMARY

- This review focuses on how to mitigate the risk of urban overheating by green-blue-grey infrastructure (GBGI).
- Fifty-one GBGI types in 10 key categories assessed by monitoring > modeling > remote sensing > mixed methods.
- Highest cooling efficiency: botanical garden > wetland > green wall > street trees.
- New GBGI implementation should consider future climate impact, multifunctional co-benefits, and unintended consequences.

www.the-innovation.org

Urban heat mitigation by green and blue infrastructure: Drivers, effectiveness, and future needs

Prashant Kumar,[1,2,10,*] Sisay E. Debele,[1] Soheila Khalili,[1] Christos H. Halios,[3] Jeetendra Sahani,[1] Nasrin Aghamohammadi,[4,5] Maria de Fatima Andrade,[6] Maria Athanassiadou,[7] Kamaldeep Bhui,[8] Nerea Calvillo,[9] Shi-Jie Cao,[1,10] Frederic Coulon,[11] Jill L. Edmondson,[12] David Fletcher,[13] Edmilson Dias de Freitas,[6] Hai Guo,[14] Matthew C. Hort,[7] Madhusudan Katti,[15] Thomas Rodding Kjeldsen,[16] Steffen Lehmann,[17] Giuliano Maselli Locosselli,[18] Shelagh K. Malham,[19] Lidia Morawska,[1,20] Rajan Parajuli,[21] Christopher D.F. Rogers,[22] Runming Yao,[3,23] Fang Wang,[24,25] Jannis Wenk,[16] and Laurence Jones[13,26]

[1]Global Centre for Clean Air Research (GCARE), School of Sustainability, Civil and Environmental Engineering, Faculty of Engineering and Physical Sciences, University of Surrey, Guildford GU2 7XH, UK
[2]Institute for Sustainability, University of Surrey, Guildford GU2 7XH, Surrey, UK
[3]School of Built Environment, University of Reading, Whiteknights, Reading RG6 6BU, UK
[4]School Design and the Built Environment, Curtin University Sustainability Policy Institute, Kent St, Bentley 6102, Western Australia
[5]Harry Butler Institute, Murdoch University, Murdoch 6150, Western Australia
[6]Atmospheric Sciences Department, Institute of Astronomy, Geophysics and Atmospheric Sciences, University of Sao Paulo, Sao Paulo 05508-090, Brazil
[7]Met Office, FitzRoy Road, Exeter EX1 3PB, UK
[8]Department of Psychiatry and Nuffield Department of Primary Care Health Sciences, Wadham College, University of Oxford, Oxford, UK
[9]Centre for Interdisciplinary Methodologies, University of Warwick, Warwick, UK
[10]School of Architecture, Southeast University, 2 Sipailou, Nanjing 210096, China
[11]Cranfield University, School of Water, Environment and Energy, Cranfield MK43 0AL, UK
[12]Plants, Photosynthesis, Soil Cluster, School of Biosciences, University of Sheffield, Sheffield S10 2TN, UK
[13]UK Centre for Ecology & Hydrology, Environment Centre Wales, Deiniol Road, Bangor LL57 2UW, UK
[14]Air Quality Studies, Department of Civil and Environmental Engineering, The Hong Kong Polytechnic University, Hong Kong, China
[15]Department of Forestry and Environmental Resources, Faculty Excellence Program for Leadership in Public Science, North Carolina State University, Chancellor, Raleigh, NC 27695, USA
[16]Departments of Architecture & Civil Engineering, and Chemical Engineering, University of Bath, Bath BA2 7AY, UK
[17]School of Architecture, University of Nevada, Las Vegas, NV 89154, USA
[18]Department of Tropical Ecosystems Functioning, Center of Nuclear Energy in Agriculture, University of São Paulo, Piracicaba 13416-000, Sao Paulo, Brazil
[19]School of Ocean Sciences, Bangor University, Menai Bridge, Anglesey LL59 5 AB, UK
[20]International Laboratory for Air Quality and Health, Science and Engineering Faculty, Queensland University of Science and Technology, QLD, Australia
[21]Department of Forestry and Environmental Resources, North Carolina State University, Raleigh, NC 27695, USA
[22]Department of Civil Engineering, School of Engineering, University of Birmingham, Edgbaston, Birmingham B15 2TT, UK
[23]Joint International Research Laboratory of Green Buildings and Built Environments, Ministry of Education, School of the Civil Engineering, Chongqing University, Chongqing, China
[24]State Key Laboratory of Soil and Sustainable Agriculture, Institute of Soil Science, Chinese Academy of Sciences, Nanjing 210008, China
[25]University of Chinese Academy of Sciences, Beijing 100049, China
[26]Liverpool Hope University, Department of Geography and Environmental Science, Hope Park, Liverpool L16 9JD, UK
*Correspondence: p.kumar@surrey.ac.uk

Received: August 7, 2023; Accepted: February 5, 2024; Published Online: February 7, 2024; https://doi.org/10.1016/j.xinn.2024.100588

© 2024 The Author(s). This is an open access article under the CC BY license (http://creativecommons.org/licenses/by/4.0/).

Citation: Kumar P., Debele S.E., Khalili S., et al., (2024). Urban heat mitigation by green and blue infrastructure: Drivers, effectiveness, and future needs. The Innovation **5(2)**, 100588.

The combination of urbanization and global warming leads to urban overheating and compounds the frequency and intensity of extreme heat events due to climate change. Yet, the risk of urban overheating can be mitigated by urban green-blue-grey infrastructure (GBGI), such as parks, wetlands, and engineered greening, which have the potential to effectively reduce summer air temperatures. Despite many reviews, the evidence bases on quantified GBGI cooling benefits remains partial and the practical recommendations for implementation are unclear. This systematic literature review synthesizes the evidence base for heat mitigation and related co-benefits, identifies knowledge gaps, and proposes recommendations for their implementation to maximize their benefits. After screening 27,486 papers, 202 were reviewed, based on 51 GBGI types categorized under 10 main divisions. Certain GBGI (green walls, parks, street trees) have been well researched for their urban cooling capabilities. However, several other GBGI have received negligible (zoological garden, golf course, estuary) or minimal (private garden, allotment) attention. The most efficient air cooling was observed in botanical gardens (5.0 ± 3.5°C), wetlands (4.9 ± 3.2°C), green walls (4.1 ± 4.2°C), street trees (3.8 ± 3.1°C), and vegetated balconies (3.8 ± 2.7°C). Under changing climate conditions (2070–2100) with consideration of RCP8.5, there is a shift in climate subtypes, either within the same climate zone (e.g., Dfa to Dfb and Cfb to Cfa) or across other climate zones (e.g., Dfb [continental warm-summer humid] to BSk [dry, cold semi-arid] and Cwa [temperate] to Am [tropical]). These shifts may result in lower efficiency for the current GBGI in the future. Given the importance of multiple services, it is crucial to balance their functionality, cooling performance, and other related co-benefits when planning for the future GBGI. This global GBGI heat mitigation inventory can assist policymakers and urban planners in prioritizing effective interventions to reduce the risk of urban overheating, filling research gaps, and promoting community resilience.

INTRODUCTION

Heatwaves are of great concern to society as they negatively impact human health, economy, and natural ecosystems.[1–4] While there is no universal definition of heatwaves, they are generally defined as episodes where the air temperature exceeds certain thresholds over days or weeks.[5,6] These hazards are extensive meteorological phenomena, typically spanning continents and involving vast amounts of heated air. The extreme heat phenomenon arises from a combination of factors, including rising urban density, elevated summer air temperatures, and intensified anthropogenic activities, resulting in surplus heat generation and reduced albedo and transpiration cooling. Consequently, it leads to elevated daytime temperatures during summer and, notably, warmer nights as well.[7] Globally, heatwaves have substantially increased in frequency and intensity since the 1950s due to climate change, and are now considered by scientific and other professional communities to be a direct consequence of rising greenhouse gas concentrations in the Earth's atmosphere.[2,8–11] Between 1998 and 2017, heatwaves were responsible for the global deaths of more than 166,000 individuals, with more than 70,000 fatalities, concomitant drought and subsequent crop failure, leading to an economic loss of EUR 16 billion[12,13] during the August 2003 heatwave in Europe.[14] There was a significant increase in the annual number of hours experiencing heat stress in South America between 1979 and 2020.[15] The rate of increase varied depending on the Köppen-Geiger class, ranging from +1.16 h per year to +8.25 h per year. In the last two decades, all the cities analyzed not only

witnessed more consecutive hours under heat stress compared with the preceding two decades, but also experienced a greater persistence of these conditions. A heat-related mortality burden of 62,862 deaths was attributed to the record hot summer of 2022 in Europe, emphasizing the need for enhanced heat surveillance, prevention plans, and long-term adaptation strategies.[16] A record-breaking air temperature of 40.3°C was noted on July 19, 2022, in the UK.[17] In July 2023, the world experienced the hottest month on record, with widespread heatwaves across many countries in the northern hemisphere.[18] Wu et al.[19] analyzed data from 717 locations across 36 countries and reported the increased mortality rates due to climate change-induced temperature variability and temperature rise, while Campbell et al.[20] in their global review of health impacts focused on vulnerable populations. Xu et al.[21] studied mortality under different heatwave definitions and accounting for different climatic contexts and different socio-demographic characteristics of the study populations to provide a more nuanced perspective than a simple heatwave duration and intensity analysis would yield. For example, the health of a given population is a strong determinant of the outcome of heatwave events and the severity should be viewed through the lens of a health-based metric.[22] However, while health impacts are critically important, there is a greater impact on human wellbeing that reaches into altered daily patterns of behavior and myriad social, and ultimately economic, consequences.[23] More frequent and intense hot extremes are expected to persist in the 21st century.[24] On land, temperature extremes will increase faster compared with the increase in global mean (land and ocean combined) temperature due to accelerating global climate change from anthropogenic emissions. This necessitates transitioning to renewables, fortifying carbon sinks, and resilient adaptation strategies.[25] Thus, the increasing frequency, amplitude, and duration of heatwaves forces governments to take action against the increasing risk of heat-related mortality and morbidity. The increasing implementation of cooling centers[26–29] is a clear indicator that governments are beginning to take action to protect their populations from extreme heat.

As acknowledged by the International Panel on Climate Change,[24] green and blue urban infrastructure elements are particularly effective in reducing air temperatures in cities. Green-blue-grey infrastructure (GBGI), which includes vegetation-based (green = trees, grass, hedges, etc.), water-based (blue = pools, ponds, lakes, rivers, etc.), and engineered (gray = green walls, green facades, and roofs) structures, have been widely proposed to mitigate the impact of urban overheating and decrease energy consumption.[30] They have the potential to play a vital role in improving the quality of life for urban residents, enhancing biodiversity, mitigating climate change impacts, and promoting overall sustainability.[31] GBGI, especially its green component, can regulate urban heat and may promote a more comfortable and cooler urban environment[32–35] through various mechanisms such as evaporation,[36,37] transpiration, shading,[38,39] and thermal insulation.[40,41] Blue infrastructure, which includes water bodies such as ponds, canals, rivers, streams, lakes, and wetlands, absorbs heat and cools the surrounding area through evaporation. Different forms of green infrastructure have been found effective in keeping urban environments cool. For example, Tan et al.[42] used a regional modeling approach for the Chicago metropolitan area and reported that green roofs reduce the near-surface temperature by 14% compared with solar panel roofs. Likewise, Blanco et al.[43] found that green vegetated walls recorded up to 7.7°C lower surface temperature than the uncovered concrete wall during summertime in Valenzano (Bari), Italy. Coutts et al.[44] highlighted that street trees can cool summer daytime air temperatures by up to 1.5°C, yet reported that the cooling effect of trees during extreme heat events was not significant between streets with and without trees. Street trees were also shown to significantly lower surface temperatures in cities across Europe.[45] Several urban heat mitigation studies have also assessed the potential of urban water management for improving urban cooling through the retention of water in the urban environment.[29–31,46–48] As an example, the integration of natural water features within cities can effectively cool surrounding areas such as lakes and ponds (10%–50% cooler inside between 30 and 200 m than at edges), rivers (5%–15% cooling effect), and urban wetlands (5%–20% cooling effect).[49,50] High amounts of tree canopy cover, green space, green roofs and walls, and open space have been reported to decrease urban heat through evapotranspiration cooling.[51,52] The combined use of multiple heat mitigation measures has been proposed to substantially decrease urban air temperatures.[52–54] Haddad et al.[54] quantified the benefits of city-scale heat mitigation measures to human health, energy consumption, and peak electricity demand. They reported that the best-performing mitigation scenarios were those that combined cool materials, shading, and greenery to reduce the peak ambient air temperature by 2.7°C in comparison with areas without GBGI interventions. Sadeghi et al.[55] analyzed the impact of urban greening infrastructure strategies (tree cover, green roofs, and green areas) on the Universal Thermal Climate Index (UTCI) under 3 scenarios across 10 weather stations in Sydney, Australia. UTCI measures the impact of urban heat on human comfort and health, considering meteorological variables (air temperature, humidity, wind speed, and radiation). It helps to explain and address the challenges posed by heat in cities. Their simulation study showed that planting 2 million well watered trees in the Sydney Basin could decrease the urban daily average UTCI by 0.2°C–1.7°C during a heatwave. Additionally, the health impact assessment revealed a potential decrease in heat-related deaths by up to 11.7 per day across Sydney. This is in addition to the numerous other health and wellbeing benefits that GGBI provides through ecological ecosystem services.[56]

Table 1 provides an overview of past review articles that focused on the heat mitigation potential of green (e.g., trees, parks, grass, pocket park, sports field, golf course, city farms, playgrounds, riparian woodland), blue (e.g., ponds, sea, reservoir, wetlands, lakes, rivers, canals, and streams), or engineered (e.g., green roofs, green walls, roof garden) interventions. The majority of these past reviews have focused on the cooling effect of different forms of green infrastructure[42,57–59] while others examined blue infrastructure in the context of urban heat mitigation.[60] However, none of these studies has systematically assessed the direct urban cooling benefits of the many various forms of urban GBGI alongside the co-benefits these GBGI interventions offer. Most reviews focused on investigating trees, followed by green roofs, parks (or other large green areas), and vertical greenings while numerous other GBGI such as gardens (botanical, heritage, nursery, zoological, rain, backyard), sports fields, vegetated balconies, water-based solutions (wetland, reservoir, estuary/tidal, river, lake, pond, sea, water canal/ditch) were not within their scope. Therefore, the novelty of this paper lies in the systematic assessment of a comprehensive list of 51 GBGI types under 10 main categories, based on the typology of Jones et al.[61] Furthermore, this review offers a comprehensive analysis of GBGI effectiveness against heatwaves in various climate zones and sub-climate conditions; and it explores the dependence of GBGI effectiveness on multiple potentially influencing factors (e.g., population density, altitude, city area, spatial scale, GBGI-area to city-area ratio, temporal trends, monitoring location, and surrounding environment). This allows us to draw insights for optimizing GBGI strategies for different conditions.

This review article aims to comprehensively consolidate and interpret the existing scientific studies related to GBGI, with the ultimate goal of creating a global GBGI database that encompasses their direct advantages such as urban heat mitigation, as well as their co-benefits, which include managing other natural hazards, addressing societal issues, or enhancing biodiversity. The specific objectives are to (1) conduct a systematic review of the literature assessing GBGI's effectiveness in urban heat mitigation and the availability of information about their co-benefits and potential drawbacks, (2) emphasize the most effective and extensively researched GBGI approach for addressing urban heating out of the 51 GBGI types that were evaluated, (3) identify areas of knowledge that are currently underdeveloped, and (4) suggest guidelines for the planning, implementation, monitoring, and evaluation of GBGI for maximum urban cooling benefits. We aim to emphasize that our systematic review is designed to provide a comprehensive analysis of the entire spectrum of research in this domain, rather than focusing solely on recent papers. Our systematic review is designed to address specific research questions (i.e., what is the efficacy of the broad range of GBGI used in urban environments? Which GBGI types are most and least efficient and what are the challenges associated with the GBGI intervention assessments for urban heating?) through a stepwise analysis of the data extracted from the relevant research papers that were identified through a predefined search and a consistent data extraction criterion.

MATERIALS AND METHODS

The review adopts a classification presented by Jones et al.[61] and expands it to 51 GBGI types under 10 broad categories: gardens, parks, amenity areas, linear features/routes, constructed green infrastructure (GI) on infrastructure, hybrid GI (for water), water bodies, other non-sealed urban areas, other public spaces, and mixed (green-blue). Apart

Table 1. Summary of the most relevant review articles since 2019 on the multiple benefits of GBGI for urban heat adaptation and mitigation

GBGI type (location)	Key findings	Authors
Green roofs and green walls (Mexico)	Examined green roofs and green walls, along with their energy, thermal, and environmental benefits, considering factors such as vegetation, climate, substrate, configuration, and green roof policies in Mexico. The primary focus was the assessment of surface and interior temperatures as critical parameters.	Ávila-Hernández et al.[62]
Green roofs (US)	Examined the impact of cool roofs, green roofs, and solar panel roofs on near-surface temperature and cooling energy demand. The effectiveness of green roofs reduced temperature by 14% compared with solar panel roofs.	Tan et al.[42]
Urban and peri-urban forests (Global)	Confirms dendrochronology as a valuable tool for evidence-based decision-making in urban planning. It has broad geographical applicability and diverse applications, including climate risk assessment, cultural heritage preservation, environmental pollution evaluation, and tree management.	Miyahara et al.[63]
GI shading, water-sensitive urban design (Australia)	Reviewed different components of heat vulnerability (e.g., exposure, sensitivity, and adaptive capacity) and mitigation options in Australia. GI and water-sensitive urban design have proven to be efficient in reducing the impacts of heat in Australia.	Adnan et al.[64]
Economic, social, environmental and cultural benefits of BGI	Emphasizes the ability of GBGI to deliver multifunctionality, meeting a number of needs, priorities, and objectives on various scales, from communities to cities to strategies, making it a critical infrastructure that is heavily dependent on design and planning. It introduces the 'four capitals' approach to help frame engineering that is synergistic with system interdependency.	Bader et al.[31]
Gardens, green roofs, vertical greening systems, public parks, urban trees, and forests (Nigeria).	Examined the current state of urban GI in Nigeria, such as domestic gardening, green roofs, vertical greening systems, public parks, and urban forests and highlighted the benefits, disadvantages, barriers, and opportunities of GI to improve environmental quality and enhance the quality of life in Nigeria's rapidly expanding cities.	Adegun et al.[59]
Trees, green roofs, vertical greenings, and water bodies (Global)	Evaluated papers for their modeling, validation, and scenario simulation process for the heatwave mitigation benefits of urban GI and BI and concluded that GBGI design should incorporate appropriate implantation location, arrangement, and orientation to optimize the shaded area for improving the cooling effect.	Liu et al.[65]
Tree canopy cover (Global)	Collected empirical data at ground level for below-canopy surface temperature and transpiration cooling of trees' canopy density. Tree canopy cover can provide shading, reduce local air temperatures, and create a cooler and more comfortable environment, particularly for pedestrians. Trees that provide dense shade at least over paved surfaces should be prioritized since every unit of leaf area index led to around 4°C of surface cooling.	Rahman et al.[58]
Green-blue (waterbodies, greenspaces, and parks) (Global)	Reviewed cooling efficiency of GBI (waterbodies, greenspaces, and parks) and identified influencing factors on the cooling effect of GBGI like size, shape, connectivity, and climate variations.	Yu et al.[66]
Blue space (ponds, lakes, rivers, canals streams) (Global)	Analyzed thermal effects of static blue spaces on the UHI and showed that the size and shape of blue spaces are important variables for the cooling achieved in an urban environment.	Ampatzidis and Kershaw[60]
Small, medium, and large-sized sized urban parks (Global)	Reviewed the cooling effects of urban green spaces in recent years and reported that the highest cooling effect distance and cooling effect intensity are for large urban parks with an area of more than 10 ha.	Aram et al.[57]
Nature-based solutions (Global)	Assessed the impact of extreme hydro-meteorological hazards, such as floods, landslides, droughts, heatwaves, and storm surges, and highlighted the significant risk reduction achieved through the implementation of GBGI. Specifically, the hybrid approach for flood mitigation and the green approach for heat mitigation emerged as the most effective solutions. However, the effectiveness of GBGI depends on its architecture, typology, green species, and environmental conditions.	Debele et al.[32]
Nature-based solutions (Global)	Reviewed different methodologies incorporating exposure, vulnerability, and adaptation interaction for hydro-meteorological risk (flood, drought, and heatwaves) assessment, focusing on mitigation effectiveness of GBGI.	Sahani et al.[33]
Nature-based solutions (Global)	Underscores the importance of incorporating ecological principles into urban planning, with a focus on integrating GI, biodiversity conservation, and NBS to promote resilient and sustainable cities and highlighted the critical role of collaborative stakeholder engagement in ensuring effective implementation, fostering urban sustainability, and maintaining ecological integrity	Heymans et al.[67]

from heat mitigation, five more co-benefits were identified: enhanced recreational opportunities, ambient noise reduction, flood and drought risk mitigation, improvements in air and water quality, and biodiversity. Detailed GBGI design and implementation principles, along with global GBGI challenges, have been covered in earlier reviews (Table 1) and, therefore, are beyond the scope of our review. The PRISMA methodology[68] was adopted for the systematic review of the literature (Figure S1). This methodology helped us to uncover geographical and temporal trends in the origin of studies, as well as knowledge voids in the existing literature. The sample for organizing the datasets obtained from the reviewed papers for each of the 51 sub-categories is presented in Table S1. Our literature search consisted of five stages (Section S1). (1) Search terms were developed based on objectives and GBGI categories (Table S2). (2) peer-reviewed literature in the English language and published between 2010 and 2023 were searched via Boolean search term combinations (Table S2) utilizing Web of Science, Science Direct, Scopus, and Google Scholar for their first 20 pages of results. This yielded a total of 27,486 publications (Figure 1A). (3) Removing duplicates and screening based on GBGI inclusion criteria left 1,512 publications for further screening (Figure 1B) and full-text retrieval eligibility (Figure 1C). Eventually, 202 publications (0.74% of the originally identified 27,486 publications) were chosen for meta-analysis (Figures 1D and 1E). (4) Relevant data (e.g., location, type of GBGI, co- and dis-benefits, and knowledge gaps) were extracted from the selected studies (Table S2). (5) The number of studies available for each of the GBGI sub-categories was categorized into six scale conditional performance classes (Table S3). The data hence obtained were analyzed using descriptive statistics on R project software.[69] Of the selected 202 publications, 64.7% solely discussed heat mitigation as their main ecosystem service; the rest discussed the co-benefits.

We categorized all the papers into four main Köppen-Geiger climate classification zones: temperate, continental, dry, and tropical (Figure S2). Each zone was further divided into three spatial scales based on the cooling observed: micro-scale (<2 km), meso-scale (>2 km <1,000 km), and macro-scale (>1,000 km). Furthermore, we tagged each study location

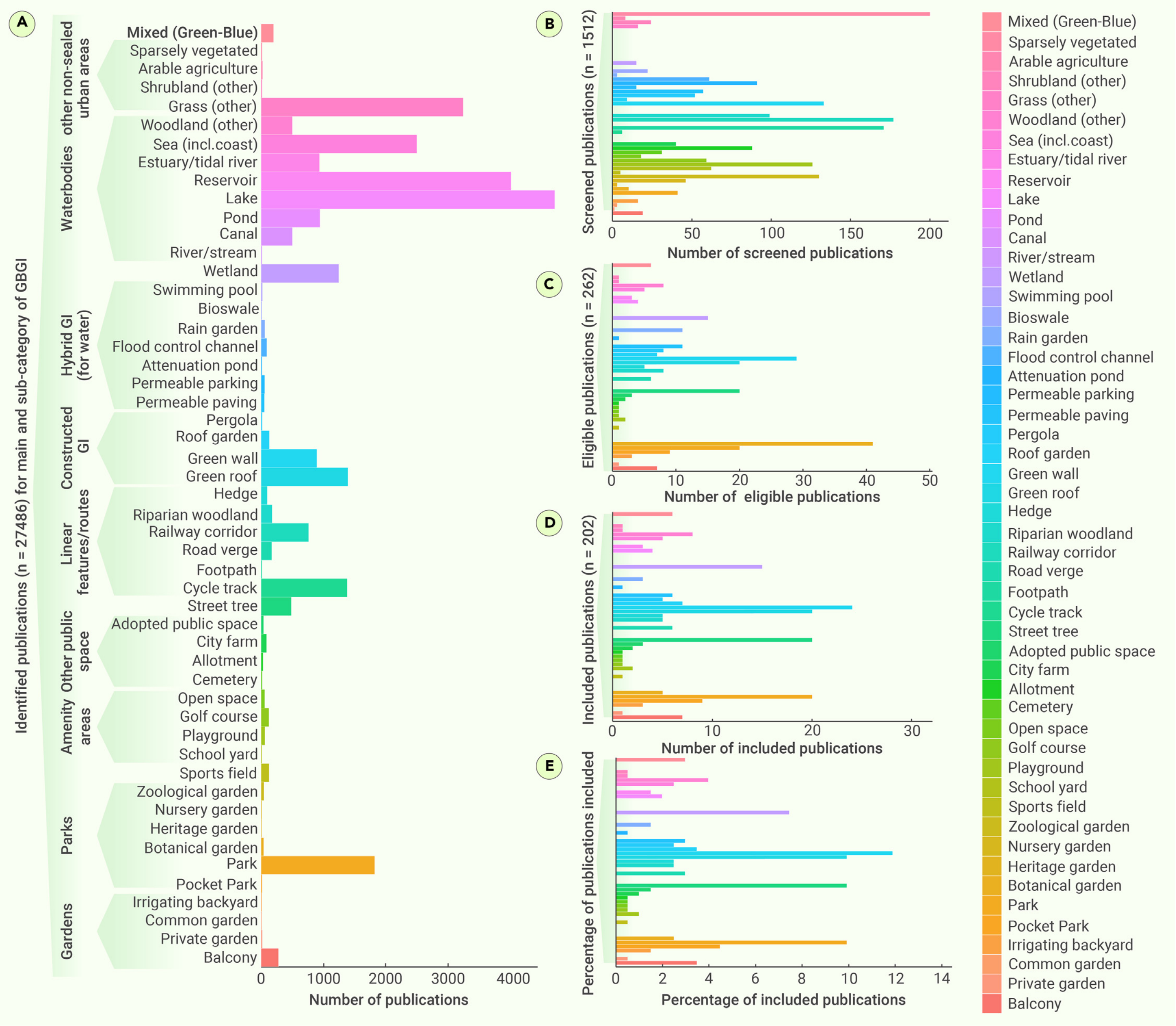

Figure 1. The literature availability across the 10 primary types of GBGI and their 51 subcategories The number of (A) identified, (B) screened, (C) eligible, and (D) included publications for meta-analysis, and (E) the percentage of included publications for each of the 51 GBGI sub-categories (shown at the y axis of A), falling under the 10 main GBGI categories (shown as bold text in A). A detailed list of the GBGI main and sub-categories is listed in Table S3.

of monitored or modeled temperatures with specific types of GBGI. To assess the potential effects of climate change on cooling efficiency, we categorized each paper into two groups based on their study time frames: past and future. Moreover, we conducted an analysis comparing the migration of GBGI systems between different Köppen-Geiger climate zones in the present and future climate conditions (Section urban GBGI and climate change). The future climate condition is analyzed under the largest emission scenario of RCP8.5[70] which covers 2071–2100.

To characterize the impact of various parameters on the cooling efficiency of GBGI under different climate zones, four main factors are considered: (a) population density, (b) the ratio of the area covered by GBGI intervention to the total town/city area, (c) altitudes (above mean sea level), and (d) temporal scale. These parameters are correlated with the cooling potential of GBGI interventions within four distinct climate zones. Population density (per square kilometer) and the altitude of each location where GBGI is implemented are obtained from the World Cities Database.[71] Additionally, the areas of GBGI and the respective city or town, as well as temporal scales, are extracted from each reviewed paper. The cooling ratio is then calculated by dividing the area of GBGI intervention by the total town or city area, providing insights into the spatial scale of the intervention in relation to the urban environment.

MECHANISMS OF TEMPERATURE AND HEAT STRESS REGULATION BY GBGI

GBGI, especially its green component, can regulate urban heat[35] through evaporation,[36,37] transpiration, shading,[38,39] and thermal insulation.[40,41] Blue infrastructure absorbs heat and cools the surrounding area through evaporation (Section S2).

Mechanisms of temperature and heat stress regulation by GI

Trees and plants help in the reduction of heat by providing shade and decreasing the amount of direct sunlight reaching the ground, therefore lowering surface temperatures and mitigating the urban heat island (UHI) effect via creating a cooler micro-climate.[38,39,72] Additionally, during evapotranspiration plants release moisture, which further cools the surrounding air by converting sensible heat into latent heat.[73] Parks can act as natural air conditioners through several mechanisms,[74–76] including the formation of micro-scale centripetal thermal system (park breeze) that generate low-level advection currents that draw air from cooler green toward warmer urban areas.[77] Other GI elements,

such as green roofs, green walls, and roof gardens, provide insulation, decrease heat absorption by buildings, and promote evaporative cooling (heat absorption, as water changes from liquid to a gas state in the air stream).[78–81] Vegetation also contributes to the dissipation of heat by acting as windbreaks, modifying airflow patterns, and facilitating natural ventilation.

Mechanisms of temperature and heat stress regulation by blue infrastructure

Blue infrastructure (BI) actively mitigates heat effects by cooling the surrounding environment[77] through processes such as evapotranspiration, shading, the albedo effect, groundwater recharge, and temperature buffering.[82,83] BI can provide cooling during the day (acts as a heat sink by absorbing and storing heat from the surrounding environment), whereas it may lead to warming at night (re-releasing the heat due to water's higher heat capacity compared with the land surface).[60] Evaporation from water bodies also helps to cool the air, creating a micro-climate with lower temperatures and thereby helping to mitigate the UHI effect.[84] Larger urban water bodies can also generate cool breezes that further lower the ambient temperature and provide relief during hot weather through evaporative cooling.[77] Furthermore, surfaces of BI are less reflective and more absorptive for solar radiation due to low albedo (0.05–0.10) than forests (0.1–0.2) or snow (8.8–0.95), especially under calm conditions; thus, there is less heating of the immediate surroundings,[85] helping to mitigate heat build-up and contributing to the cooling of the surrounding area. However, water's lower albedo does not always guarantee cooler surrounding areas as it absorbs heat, affecting the local climate. It retains more solar energy, leading to increased humidity and moderated temperatures nearby. The overall impact relies on factors like water's heat capacity, air movements, and local weather complexities, making direct temperature comparisons challenging. Some of the BI such as wetlands, ponds, lakes, swales, and rain gardens also act as natural sponges, storing water and releasing it during high air temperatures, thereby moderating temperatures in the vicinity by increasing water availability for evaporation through groundwater recharge.[37]

GLOBAL MAPPING OF GBGI STUDIES FOR URBAN HEAT MITIGATION

Temporal and spatial trends

Analysis of publication trends offers insight into how knowledge evolves, aids in understanding context, identifies gaps, ensures credibility, and supports well informed decisions, enhancing the depth and accuracy of analysis. Figure S3A illustrates the chronological trends of publications from 2010 to 2023 included in this review. A general increase in the number of publications investigating GBGI portrays a growing interest and research activity on the topic for urban heat mitigation and other benefits. This can be attributed to heightened concerns about climate change and impacts on urban heat, propelling a global shift toward sustainable urban planning and environmental considerations.[86] This surge might also be driven by the proven effectiveness of GBGI in mitigating heat and its associated benefits, prompting increased attention and research efforts.[87] While the earlier years (2010–2016) saw a relatively low count of publications, with a range of 1.9% (n = 4) in 2010 to 5.5% (n = 11) in 2016, a substantial linear increase (R^2 = 0.69; $p < 0.05$) was observed worldwide after 2016 with 8.9% (n = 18) in 2017, peaking at 28.2% (n = 57) in 2022. Overall, the trend indicates a growing interest and research activity for the use of GBGI for urban heat mitigation and associated benefits. The peak in 2022 is likely partially attributable to the coronavirus disease 2019 pandemic, with many countries imposing lockdown and movement restrictions, which raised awareness of the benefits and motivating research into GBGI.[88–90]

The spatial trends of GBGI studies were scrutinized to systematically evaluate and help identify geographical areas with varying research focuses the distribution of GBGI studies, focusing on their effectiveness against heat within different global regions (Figure 2). This analysis offers insights into specific regional needs, effective practices, and potential transferability of solutions across different locations. Most of the GBGI studies originated from Asia (51.1%), primarily from China (29.95%), followed by Europe (30.4%), Australia (7.5%), and North America (7.0%). Far fewer studies have been carried out in South America (1.8%), Africa (1.8%), and Oceania (New Zealand, 0.4%) (Figure 2). The prevalence of GBGI in Asia and Europe (81%) can be attributed to various factors.

The type of GBGI studied varied across continents. For example, in Asia, wetlands (14.7%) and parks (12.9%) were the most frequently studied GBGI types for combating urban overheating. In contrast, the most common GBGI measures reported in Europe were street trees (18.8%), green walls (15.9%), and green roofs (13.0%) (Figure 2). In Australia, green wall studies were prominent (17.6%), alongside irrigating backyards, parks, street trees, and green roofs, which constituted 58.8% of the total GBGI studies. In North America, parks (31.3%), street trees (18.8%), and wetlands (12.5%) made up approximately 62.5% of GBGI interventions described. The disparity in types of GBGI studied across continents likely stems from diverse regional contexts, climate conditions, and urban planning priorities. This reflects the varying environmental needs and urban challenges specific to each continent, shaping the focus on different types of GBGI interventions best suited to tackle urban overheating in their respective regions. For instance, in Asia, the extensive development of GBGI (wetlands, parks, and green spaces) are a response to challenges posed by rapid urbanization, cultural preferences that prioritize green areas for community activities,[91] and environmental goals focused on biodiversity conservation, improved air quality, and mitigating the UHI effect.[92] Government initiatives in Asian countries prioritize large park creation as part of urban planning and environmental policies,[93] reflecting a comprehensive approach to addressing challenges associated with urban development. In Europe, GBGI strategies involve diverse approaches, such as integrating GI into urban areas, implementing river restoration projects for water quality improvement, adopting agroecology and sustainable agriculture practices, and emphasizing biodiversity conservation through protected areas and Natura 2000 networks. These strategies align with broader EU environmental goals, notably Horizon 2020 projects and the European Green Deal.[94] Interestingly, this shows an increasing interest in the widespread application of the heat mitigation benefits provided by GBGI in low-income and lower-middle-income, relatively less developed, and highly populated continents such as Asia and Africa (total 52.9%). Comparatively, the most developed continents such as Europe, Australia, and North America (total 44.9%) demonstrated a lower but similar number of studies. The varying representation in GBGI research by different continents may be attributed to various factors, such as resource availability for research, different competing regional priorities, socio-economic challenges, lack of GBGI benefits awareness, and number of established academic or research institutions focusing on GBI research.[95] Africa and South America have initiated some interest in GBGI research for urban heat mitigation. Williams et al.[96] reported on the effectiveness of adaptive responses to climate change in Africa, finding that the fewest actions were reported for cities, with only a 5% response rate.

Types of GBGI interventions

Figure S4A summarizes the number of studies available for each GBGI type and the corresponding studies for each of the 10 GBGI are shown in Figures S3B and S4B. More than three-quarters (75.7%) of all studies focused on only four GBGI types: constructed GI on gray infrastructure (27.7%), linear GI features and routes (17.8%), parks (16.8%), and waterbodies (13.4%). The remaining 24.2% of studies focused on six other GBGI categories: gardens (5.5%), other non-sealed urban areas (4.9%), hybrid GI for water (4.9%), other public space (3.5%), mixed (2.9%), and amenity areas (2.5%). Among the studied GBGI categories, green walls (11.9%), green roofs (9.9%), street trees (9.9%), and parks (9.9%) were the most studied (41.6% of the total). The second most studied (46.0%) GBGI sub-categories included woodland, lakes, wetlands, permeable paving, pergola (with vegetation), hedges, riparian woodlands, botanical gardens, road verges, pocket parks, and balconies. The least studied categories (12.4%) included private gardens, irrigated backyards, sports fields, playgrounds, golf courses, shared open spaces, cemeteries, allotments, city farms, adopted public spaces, permeable paving, attenuation ponds, reservoirs, shrublands, and grass. This distribution of research focus on different GBGI types highlights unequal attention to specific categories and underscores the need for more comprehensive research across various GBGI types to ensure a balanced understanding of their environmental impacts and benefits.[95,97]

Effect of various parameters on the cooling efficiency of GBGI under different climate zones

Climate zones classified by the Köppen[98] encompass a range of environmental conditions that shape the characteristics of various regions across the globe. In this classification, there are five primary climate zones and 30

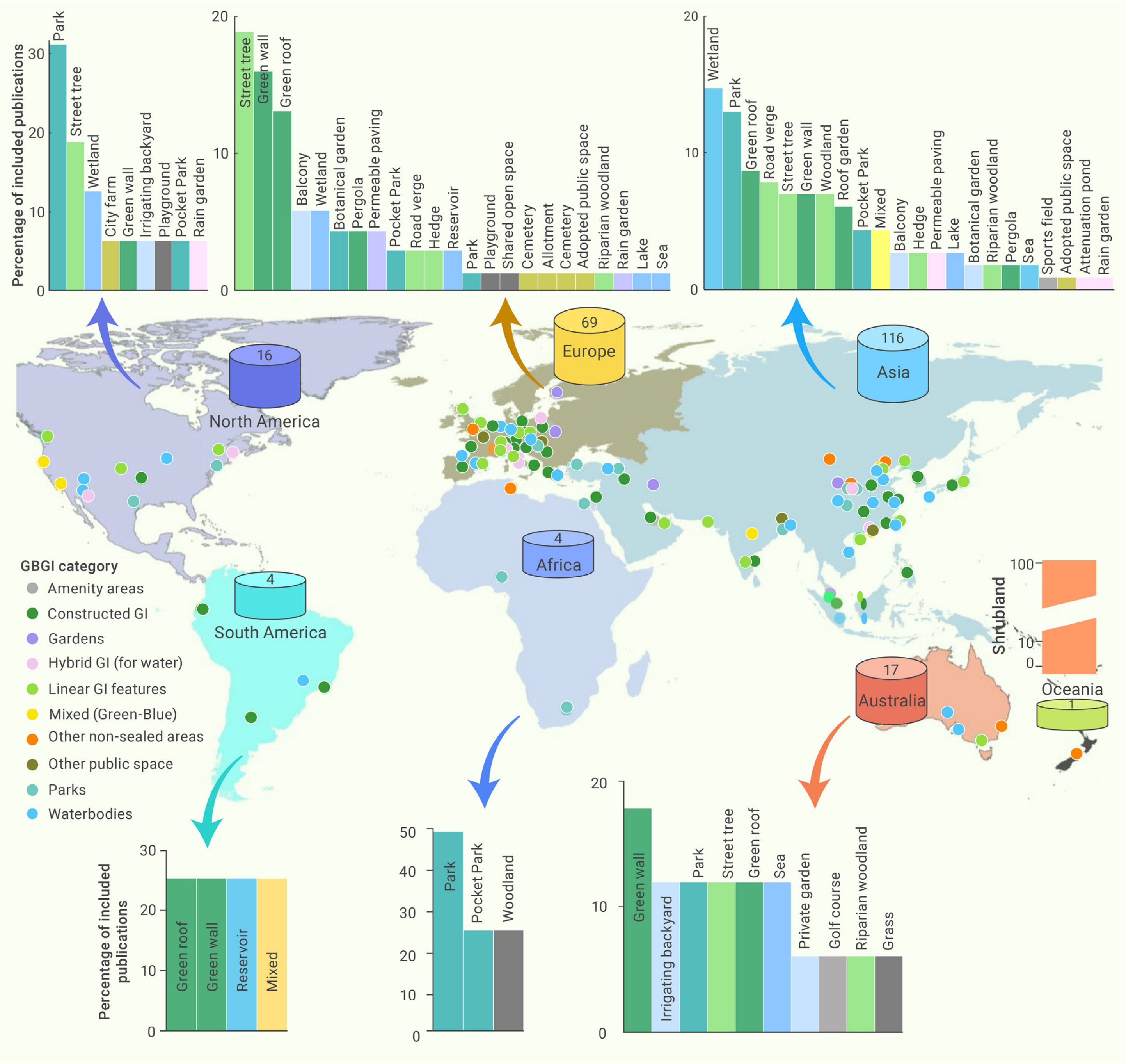

Figure 2. Geographical distribution of reviewed papers based on the number of GBGI categories, their location (latitude and longitude) by continent and number of publications by year The number in the magnetic disk and bar plot shows the number and percentage of GBGI sub-categories and types in each continent.

sub-types. Because heatwaves are not a problem in the polar climate, which is characterized by extremely cold temperatures, no GBGI interventions are retrieved from this climate zone (Figure S5). Therefore, the GBGI presented in this study only covers 4 primary climate zones and 18 subtypes. Noteworthy examples (Table S4) were found in the analysis for specific climates. The majority (87%) of GBGI were found to be located in the temperate (67%) and continental (20%) climate zones, encompassing categories such as Cfb, Cfa, Cwa, and Dwa, characterized by warm, hot, and rainy summers (Figure S2). Within these climate categories, GBGI constructs such as GI on infrastructure, parks, and linear features and routes were the most frequently used, while the other 13% of GBGIs are located in tropical (Aw, Af) and dry (BWh, BSh) climate zones. In temperate climates (Cfa and Cfb sub-climate), wetlands and parks were the most effective for cooling due to evapotranspiration, shade, waterbodies, and the impact of green space, reducing temperatures by approximately 9°C–10°C (Figure S5). Wetlands showed efficient cooling at the meso-scale, often in natural surroundings, while parks excelled in micro-scale cooling, particularly near built-up areas emphasizing the crucial role of wetlands and parks in urban GI for temperate climates.[99]

For continental climates (Dfb sub-climate), green walls and botanical gardens were notably efficient, achieving cooling of approximately 9°C–10°C nearby, likely due to their localized impact (Figure S5). Their effectiveness was observed mainly at the micro-scale but not at the meso-scale and/or macro-scale. However, parks also showed efficient cooling at the meso-scale due to their larger size and broader coverage, which allows them to exert a more significant influence over a wider area, thus demonstrating better cooling performance in the meso-scale environment compared with other GBGI types. Green roofs, situated within a mix of built and natural areas, and botanical gardens monitored inside and outside within built-up areas, showed significant cooling in the Dwa sub-climate of continental climates at an efficiency of approximately 10°C due to their ability to provide insulation, decrease heat absorption, and support evaporative

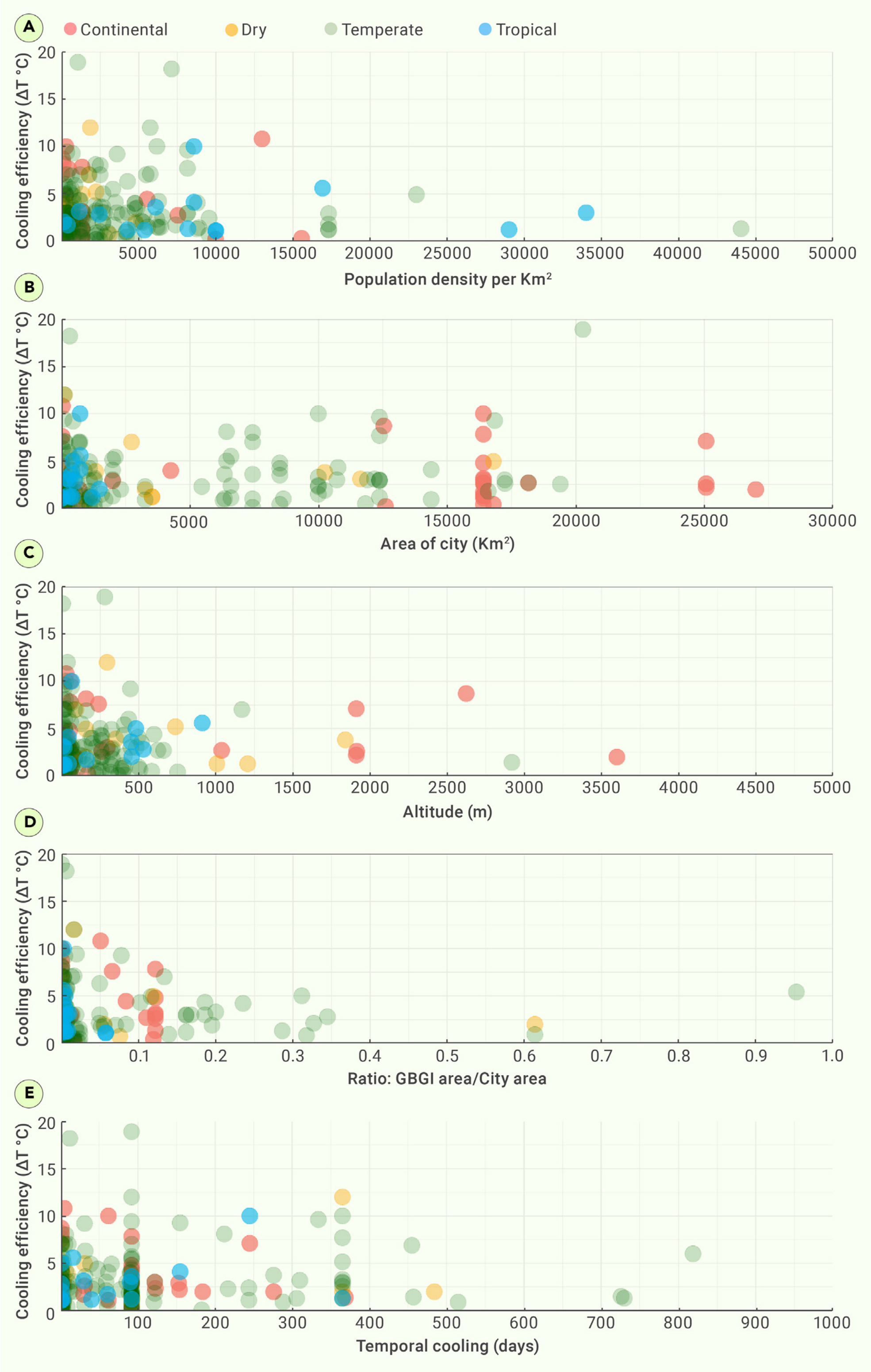

Figure 3. Scatterplot of GBGI cooling efficiency Scatterplot of GBGI cooling efficiency in different climate zones and against population density (A), area of city (B), ratio: area of GBGI/area of city (C), altitude (D), and temporal scale of cooling (E).

cooling processes, especially in such particular climatic conditions.[100] In dry climates (BWh sub-climate), pocket parks and wetlands were the most effective, reducing temperatures at the micro-scale by 7°C and 12°C (Figure S5), respectively, allowing for a concentrated cooling effect of GI within arid environmental conditions.[101] The high efficiency of roof gardens in tropical climates (Af sub-climate) was achieved up to approximately 10°C cooling. This is primarily due to their localized micro-scale impact, particularly when situated within or on top of buildings in densely built-up areas.[102] In conclusion, the variability in GBGI effectiveness across climate zones underscores the need for region-specific strategies. Furthermore, GBGI effectiveness for urban cooling was associated and discussed against population density, city area, altitude, and temporal duration of study (Figures 3 and S6; Table S5).

Population density. For the studied GBGIs, the population density ranges from 0.05 to 111,227/km^2, with a correlation value of 0.02 and a p value of 0.7 for cooling performance. Optimal heat management performance observed in low-population density cities suggests a balance between urban development and sustainable cooling strategies.[103] Studies conducted in tropical climates with dense urban populations demonstrated relatively reduced cooling efficacy (Figure 3A). In dry climates, such as those found in Mediterranean regions, the importance of BI becomes paramount because of less favorable conditions for GI.[104] Hence, the effectiveness of GBGI in this area may depend on water management strategies. Understanding the intricate relationship between cooling efficiency, population density, and climate zones is essential for tailoring sustainable urban development strategies that accommodate the unique characteristics of different regions.

GBGI versus city area. The larger urban areas, especially in continental, dry, and temperate climates, exhibited a positive correlation with noteworthy average cooling effects of 3.3°C (Figure 3B), suggesting substantial potential for GBGI implementation. In dry climates, the spatial extent of larger city areas amplifies opportunities for implementing GBGI, such as green roofs and permeable pavements,[105] enhancing average cooling efficiency to 3.4°C. GBGI interventions in tropical climates were primarily confined to smaller city areas, reaching up to 3°C cooling efficiency on average. This indicates the potential for expansion to larger extents for more significant cooling benefits. Tropical climates present distinctive challenges to GBGI implementation in larger city areas, where certain GBGI interventions may initially be confined to smaller urban spaces due to elevated humidity and heavy rainfall.[106] Nevertheless, the potential for expansion to larger extents hints at the prospect of significant cooling benefits in tropical climates. Figure 3C presents a comparison of the GBGI area with the studied city area, with cooling efficiency ratios ranging from 0.0009 to 0.95. The correlation (*r*) value is 0.02, with a p-value of 0.8. A smaller ratio indicated a reduced GBGI area in comparison with the city area, with the most significant cooling effect observed for ratios of less than 0.3, especially evident in tropical regions. This signifies the optimal functioning of GBGI at the micro-scale, indicating the potential for expansion of GBGI interventions across different climatic contexts to cover the entire city area. The observed relationship between the GBGI area to city area/ratio and cooling efficiency aligns with past research in urban climatology.[107] The concept that a smaller ratio, indicative of GBGI smaller 0.2–0.3km^2 can provide effective cooling distribution,[77] results in greater cooling

benefits is consistent with the idea that localized greenery and shade provision can have a substantial impact on micro-climate regulation.[108] The prominence of enhanced cooling effects in tropical regions, particularly when the GBGI area-to-city area ratio falls below 0.3, resonates with research emphasizing the effectiveness of GI in equatorial climates.[109,110] These findings suggest that the micro-climatic benefits derived from GBGI interventions are particularly pronounced in regions where the need for heat mitigation is more acute. Conversely, the diminishing cooling efficiency observed with increasing city size in temperate regions corresponds with studies highlighting the challenges of scaling up GI in larger urban areas.[111] The 99th percentile reaching a cooling ratio of 0.52 in temperate regions confirms the challenges posed by urban sprawl and the difficulty in maintaining a balance between urban development and environmental sustainability.[112]

Altitude. A considerable cooling impact was observed up to an altitude of 500 m across various climates (Figure 3D). The altitude ranges from 0 m to 3,600 m above mean sea level, with a correlation value of 0.02 and a p value of 0.8. However, a further increase in altitude did not yield a subsequent increase in the cooling efficiency of GBGI. Previous research suggested that high altitude is likely associated with a strong cooling effect of urban green space, but further research need to be conducted to confirm the findings.[113] The impact of altitude on cooling by urban interventions, including GBGI, is complicated by many factors. Humidity decreases with altitude, and in drier climates the cooling effect of GBGI is stronger.[114] Temperature also decreases with altitude, so urban environments in mountainous regions are cooler in general. In contrast, the downward solar radiation (irradiance) increases with altitude, as it is absorbed by clouds and other species in the atmosphere.[115] So buildings and other urban surfaces in higher altitudes receive more direct heating from the sun than those at sea level. The dominant factor or net result will vary from place to place and will depend on local topography and climate conditions that also introduce variability.[116] Urban planning and climate mitigation efforts should consider altitudes of the places for heat management by GBGI.[24] Water scarcity in dry climates poses challenges for sustainable urban cooling incorporating GBGI. Here, altitude may influence the availability of water resources for BI elements, thereby impacting the overall effectiveness of GBGI. While altitude may provide relief from heat in hot and humid tropical climates, the success of GBGI implementation could hinge on factors such as the prevalence of vegetation and water bodies.[117]

Temporal scale. Examining the temporal scale of cooling, the reported duration of cooling effects varied, ranging from a few hundred days to approximately 800 days, with the majority falling under 100 days. The correlation between cooling efficiency and temporal scale is 0.08, with a p value of 0.3. The association between the temporal scale and cooling efficiency shows a weak correlation, with data points scattered around the mean value of 136.1 days. For continental, dry, and temperate climates, the mean reported GBGI cooling efficiency is approximately 3.4°C over a monitored period of approximately 100–200 days in accordance to the typical duration of the summer or warmest season in these climate zones, lasting 3–5 months. GBGIs such as parks, green roofs, and green walls exhibited optimal cooling efficiency within these time frames, highlighting the relevance of considering climatic conditions in the design and planning of such interventions.[118] Similarly, for the tropical climate zone, the mean reported GBGI cooling efficiency is approximately 3.0°C monitored over a duration of approximately 75 summer/hot days (Figure 3E). Furthermore, the cooling efficiency shows a better association with tropical climates, with a correlation coefficient of 0.3, although it is not statistically significant.

Studies conducted for longer periods were predominantly observed in temperate and a few dry climates. Notably, GBGI efficiency seemed to decrease when studied for more than 400 days. This might be due to the degrading health of GBGI after summer. In arid or dry climates, water-efficient GBGI solutions can limit the hindrance in GBGI health caused due to water availability issues. In particular, the combination of gray infrastructure supporting water conservation and drought-resistant GI becomes paramount for localized cooling in water-scarce regions.

This comprehensive analysis reveals that the effectiveness of GBGI for urban cooling varies significantly based on climatic zones, population density, city area, altitude, and temporal duration of study. The findings indicate a preference for GBGI interventions in cities with lower density and larger areas, showcasing the potential for scalability and enhanced effectiveness of GBGI, especially in tropical regions when expanded to cover larger city extents. The limitations in denser urban settings and the reduced effectiveness over longer temporal scales highlight key areas for future research and considerations in implementing GBGI for urban cooling strategies.

Co- and dis-benefits of GBGI intervention

Figures 4A–4C show the availability of studies based on a six-point scale evidence-based classification on co-benefits and dis-benefits, respectively. GBGI interventions possess the potential to deliver multiple co-benefits, encompassing, for example, stormwater management, carbon sequestration, and improved air quality and urban resilience to hazards such as urban overheating. However, GBGI may occasionally have dis-benefits (e.g., social exclusion, increased pollen, or mosquitoes, etc.) as well, and these dual effects were reflected in the reviewed studies.[119] Co-benefits were reported for 30.2% of studies, while 7.9% also reported potential dis-benefits associated with GBGI implementation for heat mitigation. Street trees had the highest number of publications (n = 11) reporting co-benefits, followed by green roofs (n = 10), wetlands (n = 6), and botanical gardens (n = 5). Other measures such as hedges, green walls, pocket parks, and rain gardens also reported co-benefits beyond heat mitigation, although in fewer instances.

Reported dis-benefits for certain GBGI, such as playgrounds, street trees, and green roofs, included higher maintenance costs, conflicts arising from land use, and unintended environmental consequences. For instance, densely planted trees can limit dispersion, which can increase pollution levels in certain conditions,[120] while certain flora can induce pollen-related allergic reactions.[121] The volume of publications discussing the dis-benefits was noticeably less than those highlighting the co-benefits, suggesting either that the overarching benefits of using GBGI for heat mitigation significantly outbalance any potential adverse outcomes or that there is a bias in the literature toward focusing on the positive effects of GBGI, while neglecting possible undesired effects.[119] Further research on evaluation and monitoring is necessary to quantify the compound and interlinked co/dis-benefits of GBGI and ensure that any implementation options are evidence-based and adopt a holistic approach to optimize their multifunctional benefits.

Efficacy quantification of GBGI cooling

The effectiveness of different GBGI sub-categories in mitigating urban heating was evaluated in four separate classes of studies: *in situ* monitoring, modeling, remote sensing, and a combination of monitoring and modeling techniques. Their efficiency was assessed in terms of temperature reduction (ΔT) in degrees Celsius, along with 95% lower confidence intervals (LCIs) and upper confidence intervals (UCIs) as a result of GBGI intervention against a reference case (without the GBGI interventions). Throughout the paper, the *in situ* monitoring and modeling studies primarily examine air temperature, while the remote sensing (RS) studies focus on land surface temperature (LST). These terms are consistently used to represent temperature in the paper. In the subsequent texts, daytime temperature is reported in Table S6 and the corresponding night-time temperature differences are presented in Figures S7A–S7C. Night-time temperature reduction efficiency of GBGI sub-categories (Figure S7B) have been sub-classified by the type of methodology (monitoring, modeling, and/or RS with averages) used for the reported cooling efficiency. The attenuation pond (monitored, approximately 6°C cooling) and roof garden (monitored, modeled, and remotely sensed, approximately 17°C cooling) reported the most efficient cooling at night time. The highest cooling was reported by RS methods owing to measuring LST rather the air temperature, followed by monitoring, modeling, and their combinations. Attenuation pond (monitored, approximately 6°C cooling) and roof garden (monitored, modeled, and RS, approximately 17°C cooling) reported the most efficient cooling at night time. The most effective cooling was observed by RS, followed by monitoring, modeling, and their combinations. The calculation of the 95% LCIs and UCIs is performed using the *t*-distribution, which is appropriate for situations involving relatively small sample sizes.[122]

In situ monitoring. *In situ* monitoring involves the use of equipment or sensors placed directly in the natural environment to collect air temperature data continuously or periodically without causing disturbance to the subjects or surroundings.[123] One-half of the studies (50%; n = 101) reported evidence from *in situ*-based monitoring, suggesting the dominance of observation-based heat mitigation assessment of GBGIs. Monitoring studies were evaluated for the efficacy of a wide range of GBGI types (n = 26) compared with

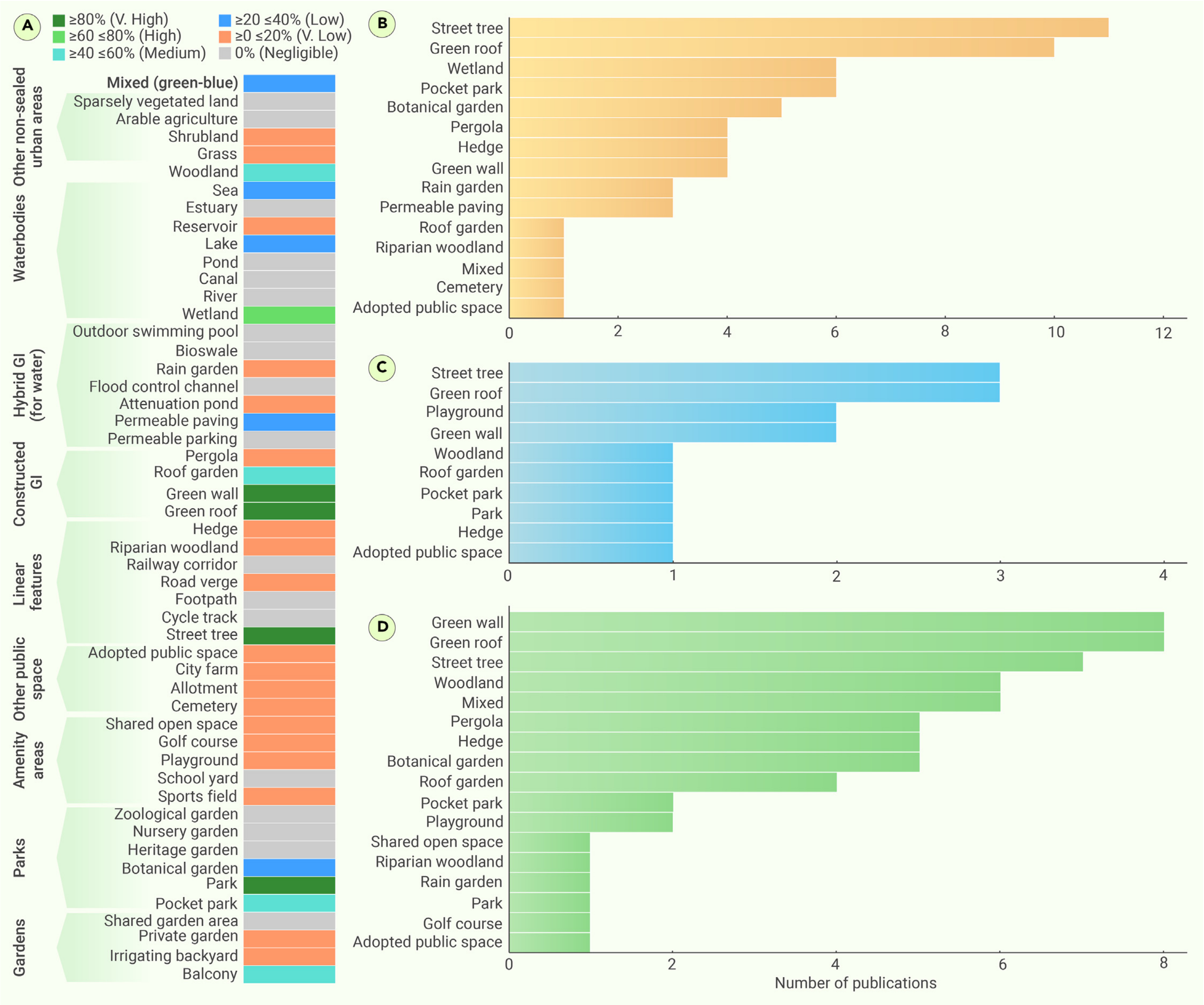

Figure 4. Number of studies under each GBGI category (A) A six-point-scale evidence-based classification of the number of studies under each GBGI category, (B) co-benefits, (C) dis-benefits, and (D) multiple interventions of GBGI within the main category of GBGI for heat mitigation. Gray cells indicate that there was no evidence found in the online database (Table S3).

modeling (n = 18), mixed monitoring and modeling (n = 14), and RS (n = 16) in Figure 5A. Based on the number of publications in each classification scale, green walls, green roofs, wetlands, parks, and street trees showed high to moderate efficiency in reducing the air temperature by up to 4.8°C, 3.9°C, 3.1°C, 3.1°C, and 2.8°C, respectively (Figure 5B). The other GBGI sub-categories, such as pocket parks, vegetated balconies, roof gardens, and woodland, are categorized as medium class, offering significant temperature decreases that range from 1.4°C to 3.07°C. Furthermore, certain GBGI types, such as sea, mixed solutions, road verge, and riparian woodland, provided relatively low (1.4°C) to moderate (3.1°C) cooling efficiency, with relatively high uncertainty (UCI, 2.8°C–4.8°C). This was presumably due to the small sample size and the consequent low level of confidence in these results. Conversely, GBGI types like rain gardens and attenuation ponds offered the highest temperature reduction (6.1°C–7°C). However, they are associated with high uncertainty due to the very limited information found within categories with low data availability. Overall monitoring-based data provided a moderate level of confidence (CI, 0.017–6.6°C).

In summary, green walls, green roofs, wetlands, parks, and street trees were found to be the most effective in mitigating heat as opposed to those showing lower efficiency such as mixed solutions, and road verges. However, the performance results exhibit considerable variability, as reflected by their CIs (Figure 5B) and the number of available publications (Figure 4A). This variability depends on numerous study-specific factors, such as local climatic conditions, the types of GBGI and their design, and upkeep, as well as the uneven distribution of studies for each GBGI type. Therefore, a cautious interpretation is needed when making any direct comparisons.[124]

Modeling. Approximately 24% (n = 48) of the reviewed literature used a modeling approach to evaluate the efficiency of different GBGIs against heat. Tree planting along streets was the most effective GBGI category, modeling results showed a temperature decrease of 4.3°C with very relatively low uncertainty (CI, 1.8°C–6.7°C). Similarly, pocket parks, hedges, irrigated backyards, wetlands, adopted public spaces, shared open spaces, road verges, green walls, rain gardens, parks, lakes, permeable paving's, and green roofs exhibit a range of modeled air temperature reduction, spanning from 0.5°C to 2.9°C (Figure 5C). The other GBGI categories, such as roof gardens, sea, green roofs, permeable paving, parks, lakes, rain gardens, and green walls, exhibited lower temperature reduction, ranging from 0.3°C to 1.48°C. Vegetated balconies demonstrated the most significant decrease in air temperature, with a decrease of 6.1°C. However, only a limited number of publications was available (n = 7) for this conclusion.

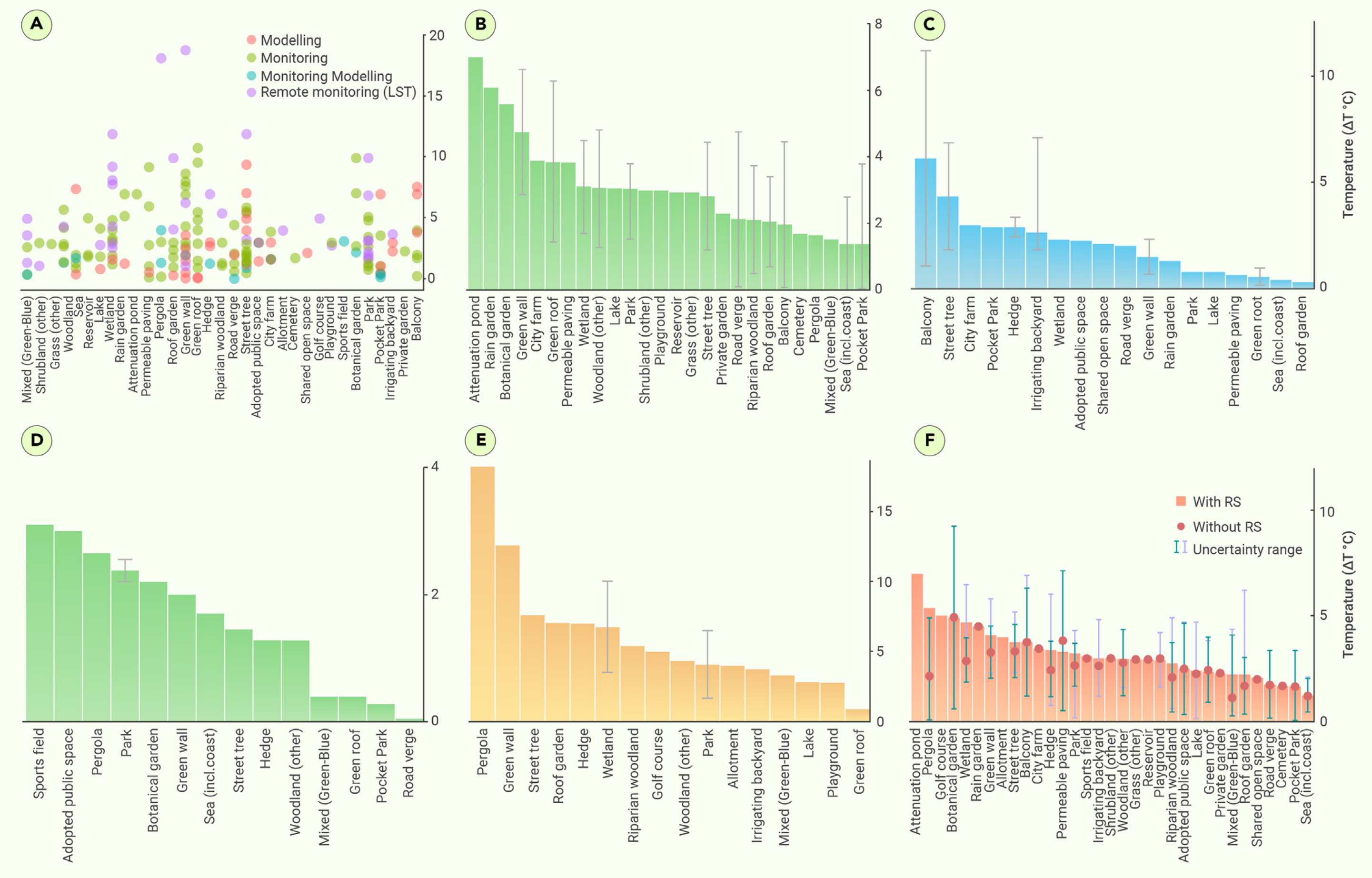

Figure 5. Performance of all GBGI sub-categories (A) Scatter representation showing the performance of all GBGI sub-categories assessed from 202 papers through the following methodologies in the reviewed publications: (B) *in situ* monitoring, (C) numerical modeling, (D) monitoring and numerical modeling (MM), (E) RS, and (F) the overall performance with and without RS (average of a-d) for each of the GBGI categories. The error bars in all plots represent 95% LCIs and UCIs as computed using the t-test. The CI is not applicable for GBGI sub-categories with very low publication availability. The data presented above from individual studies are summarized in Table S3.

Model-based data provide a reasonably narrow level of confidence (CI, approximately 0 to 0.5°C–6.4°C) when assessing the effectiveness of GBGI measures in decreasing air temperatures. This might be attributed to the use of models that work with extrapolated datasets for heat evaluation, which compensate for the limitations arising from limited coverage and the uneven distribution of data from weather stations and monitoring networks. However, it is essential to validate and calibrate these models using observational data to ensure accuracy, identify biases, and account for uncertainty. By combining model-based data with monitoring data, a more comprehensive and robust assessment of heat mitigation can be achieved.[125]

RS. RS-based temperature monitoring entails the use of satellite or airborne sensors to measure and observe temperature fluctuations over extensive regions. Approximately 16% (n = 32) of the total reviewed literature reported varying levels of efficiencies of different GBGI in reducing the LST during very hot days using RS techniques. Among the 16 types of GBGI studied through RS, green walls demonstrated the greatest efficiency, with an LST decrease of 12.6°C (Figures 5A and 5E); however, the uncertainty is very high because only two papers monitored green walls using RS-based techniques. Similarly, street trees, hedges, roof gardens, and wetlands showed relatively high LST decreases of 6.8°C and 7.6°C, respectively. The greatest variability in the reported LST is influenced by spatial resolution because RS platforms (especially satellites) capture data over larger spatial scales, which may include a mix of different land cover types.[126] Consequently, the integration of temperature values from various surfaces, including hot urban areas or exposed surfaces, can lead to higher average temperature readings.[127] Therefore, while RS-based measurements offer extensive coverage and the ability to provide spatially detailed information, their accuracy should be validated and calibrated with ground-based measurements.[34]

Interestingly, mixed initiatives (green-blue), irrigated backyards, allotments, parks, woodland, golf courses, and riparian woodland showed varying levels of efficiencies in surface and air cooling, ranging from 2.8°C to 5.4°C (Figures 5A and 5E). Conversely, some GBGIs such as green roofs demonstrated lower efficiencies with temperature decrease values of 0.9°C due to factors such as soil moisture and vegetation density.[41,128,129] These factors, including shallow soil depths, limited water availability, and high evaporation rates,[36,130] can limit the cooling potential of green roofs.

RS-based monitoring demonstrated that pergolas and green walls exhibited relatively greater temperature reduction efficiencies with high uncertainty emerging from the small number of available publications (n = 2) as opposed to green roofs demonstrating relatively lower efficiencies (n = 20) (Figure 5E). In general, RS studies showed the greatest decreases as compared with the monitoring, modeling, or their combination, and they presented higher uncertainties (as indicated by the CI in Figure 5E). This outcome was expected, as RS studies mostly report LST as opposed to the air temperature used in other cases. To decrease the uncertainty in GBGI performance, it is essential to validate RS-based efficiency assessments against *in situ* measurements.

Combined (in situ, modeling, and RS) studies. Combined temperature monitoring encompasses the use of more than one method (*in situ* measurements, temperature modeling, or RS) to monitor and analyze temperature fluctuations across various scales, ranging from local to global levels.[131] It provides a comprehensive and accurate evaluation of the efficiency of GBGI for heat mitigation, offering spatial and temporal coverage, multi-dimensional insights, and cost-effective data collection. Approximately 9.5% (n = 19) of the reviewed publications covered mixed monitoring and modeling approaches for evaluating the efficiency of different GBGIs in decreasing the temperature (Figures 5A and 5D). Among the 14 types of GBGI assessed through this method, sports fields exhibited greatest highest efficiency of 3°C–3.1°C with high uncertainty/CI, representing a very low publication availability. Other GBGI sub-categories that show a very robust temperature decrease with low uncertainty including woodland,

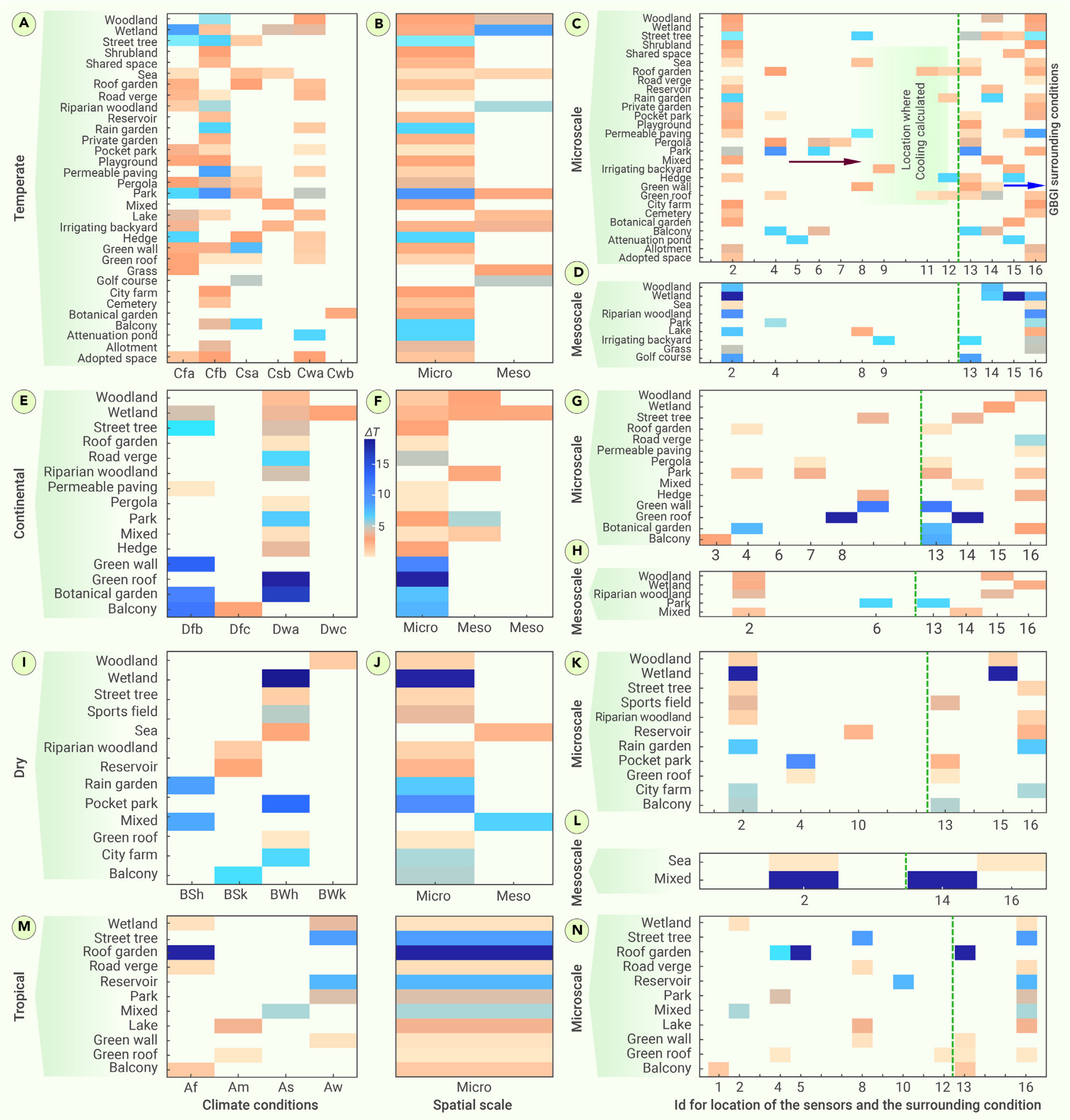

Figure 6. Köppen-Geiger climate conditions Each GBGI's performance is classified into Köppen-Geiger climate conditions (A, E, I), followed by categorization into spatial scales (B, F, J). Cooling performance in the same climate zone at three spatial scales is linked to the location (left: C, D, G, H, K, L, N) and surroundings (right: C, D, G, H, K, L, N) of the GBGIs. The location IDs (1: front, 2: inside, 3: inside and near, 4: inside and outside, 5: inside and top, 6: inside, outside, and near, 7: inside, outside, and top, 8: near, 9: not reported, 10: outside, 11: outside and top, and 12: top) represent where the cooling performance was calculated. The surrounding conditions (13: built-up area, 14: mixed [built-up with nature], 15: nature, and 16: not reported) describe the environment around the GBGIs.

hedges, street trees, seas, green walls, botanical gardens, and parks (with temperature reductions of ΔT = 1.3°C, 1.3°C, 1.5°C, 1.7°C, 2°C, 2.2°C, and 2.4°C, respectively) (Figure 5F). Green roofs, mixed initiatives (green-blue), and road verges showed relatively lower temperature reduction efficiencies of 0.4°C, 0.4°C, and 0.05°C, respectively (Figure 5F). Furthermore, to analyze the impact of uncertainties arising from RS-based measurements, the performance was evaluated both with and without RS studies. Generally, RS-based measurements give higher mean performances with relatively high uncertainty, when compared with other approaches (combined monitoring and modeling) (Figure 5F).

Spatial scale. While meso-scale and macro-scale approaches are vital for comprehensive urban planning, the dominance of micro-scale GBGI implementation (approximately 85%) underscores its pragmatic, community-centric, immediate, and cost-effective impactful role in mitigating heatwaves in specific urban areas (Figure 6). For instance, constructed GBGI on built infrastructure (28%)

such as green roofs, green walls, and roof gardens are the most common features implemented at the micro-scale. Other dominant GBGI categories at the micro-scale are parks (16%), and linear features and routes (e.g., street trees, 16%). GBGI such as street trees, parks, and green roofs offer highly localized, direct, and immediate benefits, including shade provision, lower surface temperatures, localized cooling effects, green spaces, and improved air quality, contributing to a healthier and more livable environment in densely populated urban zones where the heat island effect is most pronounced.[132,133] Compared with meso-scale and micro-scale interventions, these micro-scale interventions can be easier to implement and more cost effective, making them an attractive option for municipalities with budget constraints, facilitating widespread implementation.[134] Micro-scale interventions also foster community engagement and acceptance, as they are more visible and immediately beneficial to local residents.[135] The limited adoption of GBGI at the meso-scale (14%) and macro-scale (0.5%) might be influenced by challenges related to macro-scale implementation, such as land availability, funding, lack of best practices that demonstrate their effectiveness at a larger scale, and complex urban planning considerations. For instance, approximately 11% of the reviewed GBGI (waterbodies: wetlands and lakes [8%]; and linear features and routes: riparian woodland [3%]) were implemented at combined meso-scale and/or macro-scale.

Location and surroundings. The location of measurements is crucial to accurately determine the cooling potential of GBGI. The location where GBGI cooling efficiency is calculated is divided into five main categories (front, inside, near, outside, and top of GBGI intervention). Furthermore, these locations are grouped into six subdivisions based on combinations of the five categories (e.g., inside and near, inside and outside). The first five categories are defined as follows: (a) front: measures temperature differences within a few meters (e.g., 1–5 m) from the front of the GBGI; (b) inside: evaluates temperature reduction and other micro-climate changes within the GBGI; (c) near: assesses micro-climate parameters in the area surrounding the infrastructure, extending to approximately 10–20 m from the intervention; (d) outside: evaluates the impact on temperature in areas just outside the intervention, within a radius of 20–50 m; and (e) top: measures temperatures and assesses cooling effects on the top surfaces of GBG interventions. In this analysis, most studies (47%) measured the cooling potential inside the boundary (i.e., core center of GBGI) followed by outside (i.e., close proximity within a distance of 20–50 m to the GBGI boundary [21%]), both inside and outside (15%), a combination of inside, outside, and nearby (3%), and varied locations such as top and near (12%). Approximately 2% of studies did not report the location of the temperature measurements (Figure 6). As expected, cooling effects (average 3.4°C) measured inside GBGI areas were higher compared with those measured outside or on top (2.2°C) of GBGI areas. This can be attributed to the influence of GBGI on the micro-climate of the surroundings. This influence primarily operates through the regulation of the local energy budget, involving two key mechanisms: evapotranspiration and the absorption and reflection of shortwave radiation. Evapotranspiration, a significant cooling mechanism,[136,137] involves the conversion of vegetational transpired water into water vapor by extracting energy from the local environment. This process contributes to a cooler environment, as evidenced in measurements taken within GBGI. Additionally, at the top canopy, the absorption and reflection of incoming shortwave radiation prevent the shaded areas below from warming up, further enhancing the cooling effect.[138] As one moves away from GBGI, the cooling efficiency decreases in alignment with the temperature-heat gradient concept. This decrease is attributed to the reduced exposure to GBGI's cooling mechanisms, such as shading and evapotranspiration, as observed in measurements taken outside and on top of the infrastructure.

The cooling efficiency of GBGI depends on the surrounding environments, including built-up areas, natural features (such as lakes and parks), and mixed environments that combine built and natural elements.[139] Of the analyzed studies, approximately 42% of GBGI are implemented in built-up areas, approximately 7% in natural areas, and 9% in mixed environmental conditions. However, a notable 42% of the papers did not report any characteristics of the vicinity of the studied GBGI. GBGI implemented in built-up environmental conditions, characterized by high-density infrastructure, presents both challenges and opportunities for GBGI in reducing heatwaves. The prevalence of impervious surfaces contributes to heat retention, exacerbating the UHI effect and decreasing the overall efficiency of the GBGI in place.[140] Conversely, GBGI implemented within natural surroundings, including lakes, parks, and tree-covered spaces, plays a crucial role in enhancing GBGI efficiency for heatwave mitigation. For example, additional trees in parks may aid in the decrease of ambient temperatures.[141]

Overall performance. Figure 7A shows the mean reduction and CIs for both cases, with and without RS studies, while the mean temperature reduction by each GBGI sub-category for the same scenario is depicted in Figure 7C. The overall mean performance of GBGI based on RS studies showed a relatively greater temperature decrease (5.8°C ± 4.4°C) with moderate uncertainty (CI, 4.3°C–7.4°C), followed by monitoring (3.2°C ± 2.4°C) with a very narrow CI (2.7°C–3.7°C), modeling (2.4°C ± 2.2°C; CI, 1.7°C–3.0°C), and mixed (1.7°C ± 1.1°C; CI, 1.1°C–2.2°C) studies (Figure 7A). The overall performance with RS measurements indicates that integrating RS introduced an error of 15.6% compared with monitoring, modeling, and combined studies (Table S7). The pronounced temperature reduction evident in RS studies might be partially attributed to their focus on measuring LST, as opposed to air temperatures measured by *in situ* and numerical modeling studies. Additionally, variations between studies within the same category that impact the outcomes of these three types of research could stem from several factors. These include differences in the deployed instrumentation, the methodologies adopted, the materials used in various global regions, and prevailing climatological conditions.

Figures 7B and 7C present the heatmaps and overall mean performances for each GBGI category, respectively. The results show that several sub-categories, such as green walls, green roofs, street trees, parks, and others, show significant temperature reduction (Figure 7B). However, some sub-categories, including attenuation ponds, pocket parks, cemeteries, shared open spaces, and others showed temperature reduction efficiency with high uncertainty. Certain sub-categories have no values for CIs, indicating an insufficient number of publications to calculate them.

The combined results from all types of efficiency assessments discussed above indicate that the most efficient GBGIs for temperature reduction are attenuation ponds and pergolas, with overall temperature reduction efficiencies of 7.0°C and 5.4°C, respectively. However, no confidence intervals are available for the attenuation pond (Figure 7A), and their efficiencies are associated with high uncertainty because that they are categorized under very low and low publication availability categories. Other GBGI sub-categories that demonstrated relatively greater temperature decrease efficiencies are botanical gardens (4.9°C), wetlands (4.7°C), green walls (4.1°C), street trees (3.8°C), vegetated balconies (3.7°C), hedges (3.4°C), permeable paving (3.3°C), and parks (3.2°C). These GBGI types had a diverse range of publication availability, ranging from low to very high.

Furthermore, botanical gardens, wetlands, and permeable paving have relatively large uncertainty, shown by a wide range of CIs; in contrast, parks, wetlands, green walls, and street trees have a narrow CI range, suggesting low uncertainty with high heat mitigation performance (Figures 7B and 7C). Conversely, sub-categories such as sea and road verge showed lower mean temperature reductions ranging from 1.3°C to 1.8°C with relatively narrow CIs, suggesting more reliable heat mitigation performance.

Considerations for GBGI efficiency assessment

The assessment of GBGI for its cooling benefits involves diverse methodologies, each with unique strengths, implications, and limitations. *In-situ* monitoring captures on-site high-resolution and accurate data for real-time, site-specific insights, but may be limited in scope and sample sizes.[34] Even if the *in situ* approach captures local nuances, enabling real-time responses to change conditions, its limitations include restricted spatial coverage, resource intensity, and potential temporal constraints, as it may not effectively capture long-term trends.[142] Modeling provides cost-effective scenarios for larger scale understanding but might oversimplify complex interactions.[125] Modeling-based assessments contribute predictive capabilities and scenario analyses, allowing for the simulation of future GBGI configurations and their impact on urban heat. These models can operate at different scales, integrating variables like land use and building density. Despite their scalability and policy guidance potential, modeling-based assessments require reliable input data, can be complex, and may introduce uncertainties in their outcomes that necessitate validation efforts. Models may also struggle to capture the full complexity of weather systems and the dynamic nature of heatwaves, leading to potential inaccuracies in predictions.[143]

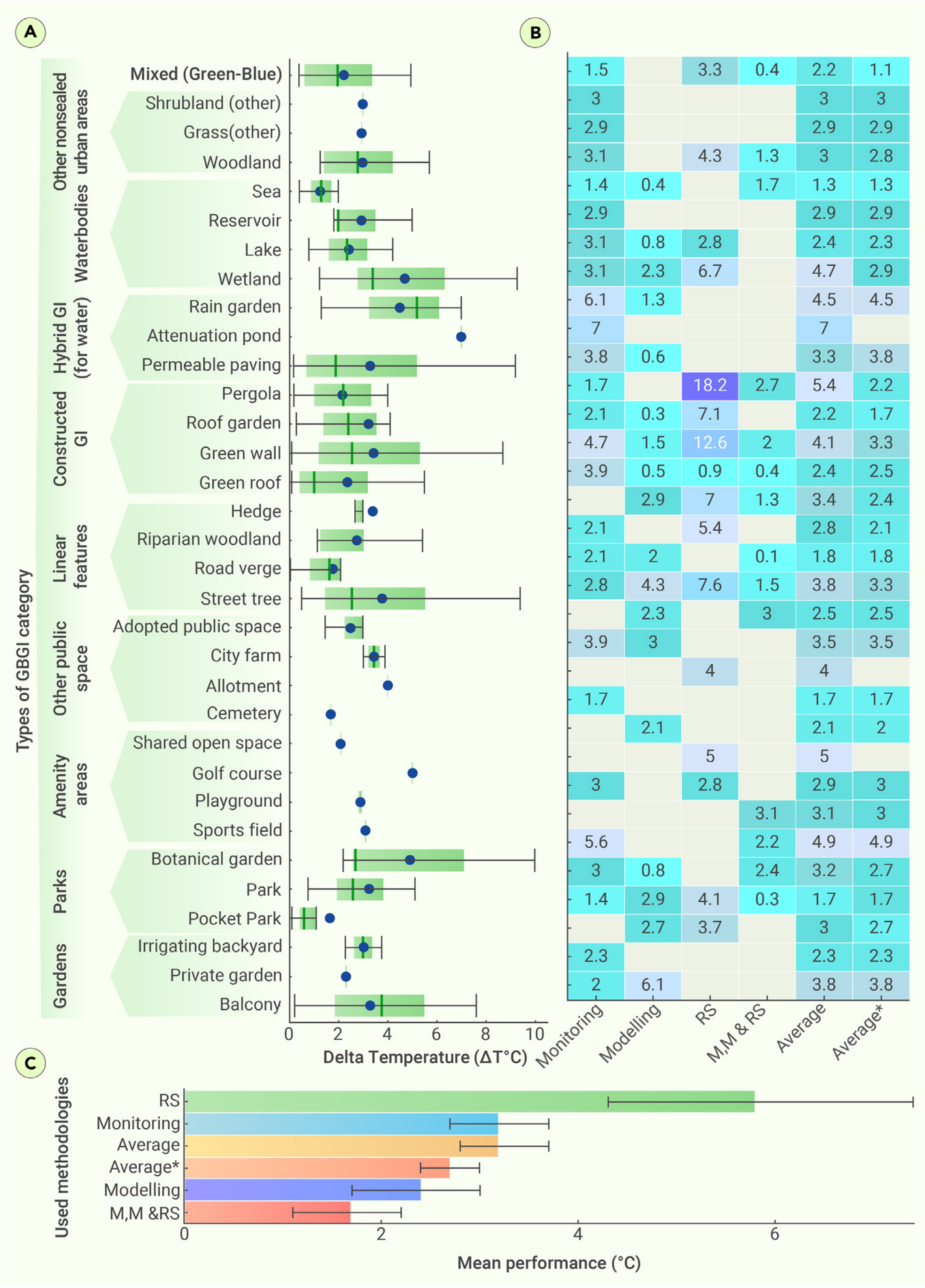

Figure 7. Efficiency of various GBGI types for urban heat mitigation (A) A summary of the overall performance of different GBGI types from all studies, (B) heatmap showing GBGI performances from for different methods and the average values, and (C) overall average of GBGI efficiency for urban heat mitigation. The error bars in all plots represent 95% LCIs and UCIs as computed using the t-test. The Average and Average* values represent the average of all study types with and without RS data, respectively. M&M denotes combined monitoring and modeling studies. The color gradient represents the performance, with gray cells representing studies that did not consider either monitoring, modeling, M&M, or RS. The figure uses a boxplot representation with the median indicated by a thick vertical black line, the mean represented by blue dots, and the upper and lower quartiles indicated by the box boundaries. The circle with a vertical line represents the GBGI categories with only one publication. All numerical data presented is provided in Table S6.

RS methods, such as satellite imagery, allows broad perspective on heatwave patterns and consistent data collection, but faces challenges that include cloud cover, spatial and temporal limitations, potential biases, and interpretation challenges. This method also utilizes various data types, including thermal imagery and vegetation indices, making it efficient for regional and citywide assessments. They may not always provide the detailed, localized information needed for an immediate response. Moreover, since RS measures only LST, there may be some uncertainty when converting these data into heatwave information. While RS is non-intrusive and cost effective, it faces challenges related to spatial resolution, dependence on weather conditions, and the need for validation through ground truthing.[144]

While technology plays a vital role in tracking and addressing heatwaves, these methods have limitations that must be carefully considered. To comprehensively evaluate GBGI's cooling effects in urban settings, an integrated approach utilizing different methodologies is crucial and has advanced our ability to respond to extreme weather events. *In situ* monitoring's detailed information can validate RS and modeling results, while RS's large-scale coverage complements the site-specific insights gained from *in situ* monitoring. Modeling offers a forward-looking perspective with its predictive capabilities, informing policymakers about the potential impacts of different GBGI interventions. These approaches together create a robust framework for urban planning, climate resilience, and sustainable development.

URBAN GBGI AND CLIMATE CHANGE

Climate change is significantly impacting global populations and ecosystems, leading to shifts in temperature and precipitation patterns across various continents that govern the climate types of the region. These changes necessitate a re-evaluation of GBGI to enhance their efficiency in mitigating and adapting to climate variations.[145–147] Nearly all (98%) of the papers focused on the current climate; only 2% specifically examined future GBGI cooling efficiency.

Table S8 outlines the projected influence of future climate change on the choice of GBGI in various climate zones. Figure 8 shows the Köppen-Geiger climate classification and the location of ten GBGI categories for present and the future under the RCP8.5 scenario. Wetlands and green wall and street trees will be ideal prospective GBGI solutions to counteract changing climate patterns, where cooler and wetter continental sub-climates (Dfb and BSk) are projected from current warm summer continental or hemiboreal climates (Dfa and Dfb), respectively. The current emphasis on street trees and permeable paving may need to evolve toward more temperature-regulating structures like green walls under the projected Dfb climate.[98] Wetlands play a pivotal role in climate resilience, particularly in regions transitioning from a humid continental (Dfa) to a fully continental (Dfb) climate. These areas are likely to experience altered precipitation patterns and increased risk of extreme weather events. Wetlands act as natural buffers, absorbing excess water during heavy rainfall and preventing floods. Moreover, they contribute to water purification, biodiversity conservation, and carbon sequestration, enhancing overall ecosystem health.[148] Green walls provide numerous benefits, including temperature regulation, air quality improvement, and aesthetic enhancement. By integrating vegetation vertically onto building structures, green walls contribute to cooling effects, mitigating the UHI effect that can be exacerbated by a shift to a more continental climate.[149] Street trees can provide shade, decrease ambient temperatures, and enhance overall urban micro-climates in regions transitioning from a temperate continental (Dfb) to a cold semi-arid (BSk) climate. They also contribute to carbon

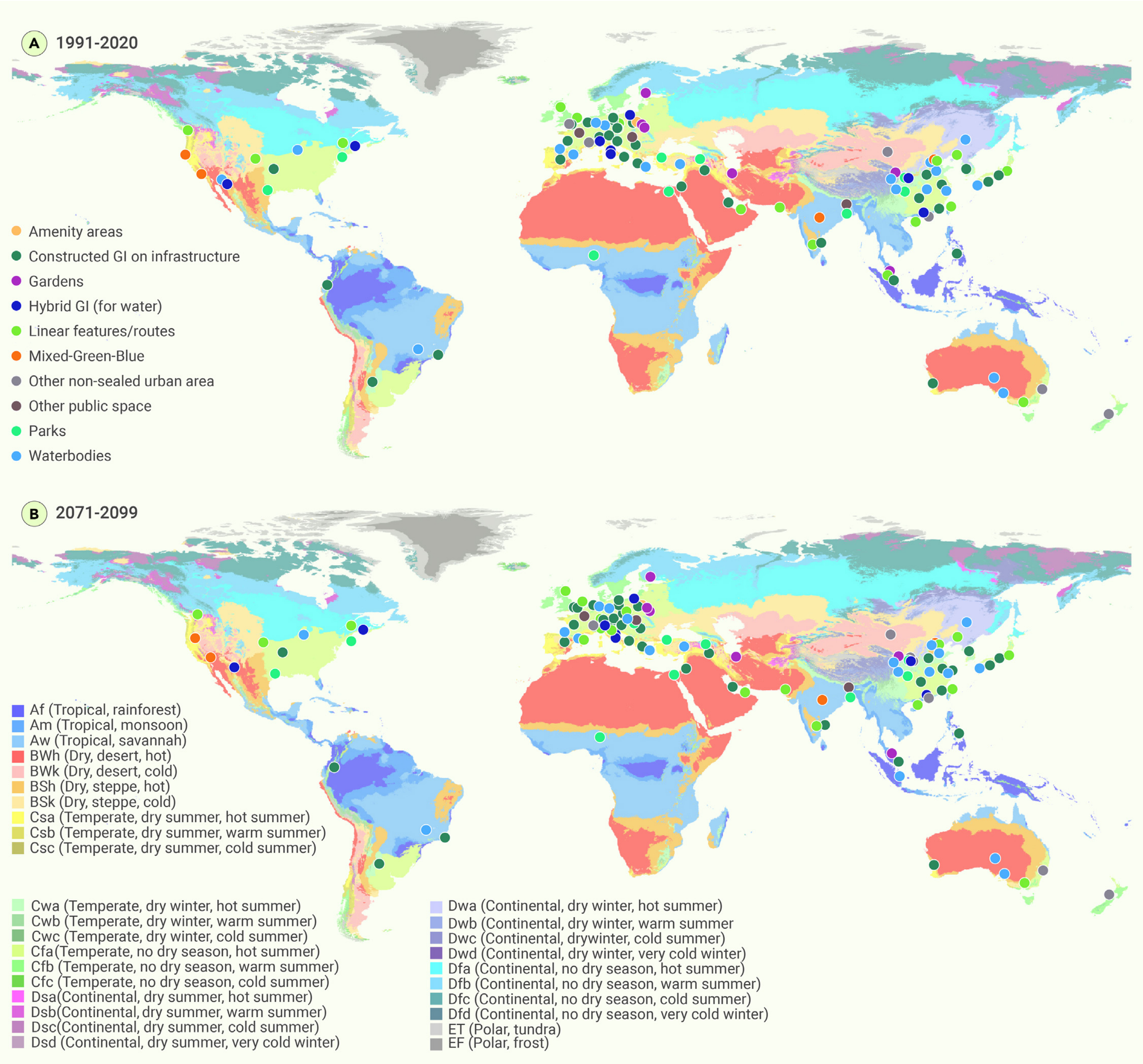

Figure 8. Base maps are Köppen-Geiger classifications, and the point are location of ten GBGI categories (A) The present-day map (1991–2020) and (B) the future map (2071–2100) under the RCP8.5 scenario.

sequestration and improve air quality, addressing multiple aspects of environmental sustainability.[133]

In the dry climate zone (BSk), the future projection suggests a potential transition toward the BSh and BWk sub-climate, requiring more adaptive GBGI measures. That means the current best GBGI solutions of balcony and wetlands need to be updated to green walls and street trees, for future BSh climate, and current wetlands to woodlands to tackle the evolving climate scenarios (BSk to BWk) for improved urban cooling and biodiversity. In dry climates shifting from semi-arid (BSk) to dry arid climate (BWk), woodlands can act as buffers against extreme weather events, enhancing the overall resilience of the ecosystem.[150] However, the tropical climate zone (Af, Aw, and As) is expected to remain relatively stable, indicating limited changes in the potential cooling effects of GBGI in this area.

For temperate zones in Europe (Cfb, Dfb), expected changes may prompt the implementation of parks, pocket parks, green walls, green roofs, lakes, and grass, offering adaptive solutions in response to projected shifts in climate (Cfa). These features contribute to temperature regulation and enhanced water management.[151] Parks and pocket parks contribute to enhanced green spaces, while green walls and roofs provide urban cooling and reduce heat island effects in the future in Europe.[152] Furthermore, lakes and wetlands expanses can aid in temperature regulation, water management, and biodiversity conservation in Europe under ongoing climate change.[153] Green roofs with supportive policies have great potential from niche to mainstream in the near future in temperate European cities for climate change mitigation.[154] However, these roofs encounter adoption challenges linked to incentive structures. Building owners bear the risks, while the public reaps the benefits.

A similar study by Zhou et al.[155] found that, in the temperate regions of China, most urban parks in (Northeast China and the North China Plain) are located in the Dwa climate zone at present, but roughly 71% of these parks would be in the Cwa climate zone in future under the RCP8.5 emission scenario between 2071

and 2100. GBGI measures should evolve from current urban parks to prioritize larger water bodies such as lakes and road verge developments to address water retention needs aligning with the principles of sustainable water management and green roofs, repurposing urban space and wetlands for the Cwa sub-climate shift in the changing climate.[156,157] Additionally, shifts were observed from Aw to tropical monsoon (Am) in both the northern and southern regions, as well as from tropical without a dry season (Af) to Am in the central and southwestern parts of Southeast Asia.[158] This transition could lead to decreased cooling effects due to limitations in the largest park cooling distance and the largest park cooling intensity in the new climate zone. Additionally, urban parks currently situated outside the monsoon climate (Am) and warm semi-arid climate (BSh) zones will undergo changes, with 44% and 20% moving into BSh and Am zones, respectively. This suggests reduced cooling effects due to projected increases in rainfall and higher temperatures. The increase in minimum temperature emerged as a crucial factor driving the climate shift from Cwa to Aw in the north, while increased rainfall was a reason for Aw to Am transition in the north and south. The findings from this study on future climate zones can aid in pinpointing ecologically vulnerable hotspots worldwide affected by climate change. They also contribute to identifying optimal GBGI measures that effectively mitigate adverse impacts such as heatwaves under changing climatic conditions. Collaboration with environmental experts and ongoing community engagement are essential components of successful GBGI implementation under present-day and future climate conditions. In areas transitioning from a humid continental climate (Dwa) to a warm temperate climate (Cwa), adapting GBGI involves prioritizing green roofs and constructed wetlands for temperature control and effective water management, respectively.[159] Thus, considering the expected alterations in climate conditions, these prospective GBGI can aim to provide more sustainable and climate-resilient urban landscapes. Hence, policymakers should consider these projected climate shifts and tailor GBGI strategies to align with these changes, prioritizing features such as green walls, lakes, woodlands, and constructed wetlands accordingly.

CONCEPTUAL FRAMEWORKS FOR GBGI IMPLEMENTATION FOR HEAT MITIGATION

Table S9 presents a qualitative synthesis of the literature in the form of a nine-stage framework for implementing GBGI measures to mitigate heat risks, promote urban climate resilience and provide other co-benefits. Meanwhile, Figure 9 depicts the four stages, along with the processes of co-planning, design, and management, full-scale development, and nine sub-processes in the conceptual framework for GBGI implementation for heat mitigation. The stages, roughly sorted according to their chronological sequence, always accept that circularity and iteration should be inherent in any design process or application of a theory of change[160] (Figure 9), include the following.

Stakeholder engagement

It is crucial to identify and involve all relevant stakeholders and key players including local communities, government agencies, urban planners, environmental organizations, etc. Their active participation in a GBGI project should be ensured from conception to implementation and beyond to gather their input related to the problem of heat, address concerns, and ensure that their needs are considered.[161,162]

Feasibility studies

It is important to conduct assessments to determine the feasibility and viability of implementing GBGI measures in heat-vulnerable areas with high heat risk exposure, particularly in densely populated urban areas lacking green spaces, where sensitive populations such as the elderly, economically disadvantaged, and those with pre-existing health issues reside. These assessments should consider technical, economic, and environmental factors. This evaluation should include an analysis of the existing urban infrastructure, the identification of potential GBGI implementation sites, and a detailed estimation of costs and benefits associated with various options.[163]

Co-benefits and dis-benefits

It is essential to assess the potential co-benefits (positive impacts, e.g., reduced temperature along with improved air and water quality, enhanced biodiversity, and increased recreational opportunities) and unintended consequences (e.g., higher maintenance costs or potential social disruptions, and allergic reactions) associated with GBGI implementation.[119,164]

Design

The plan and design of heat-reducing GBGI measures based on the feasibility study and stakeholder input could include determining the appropriate mix of GI (e.g., parks, urban forests, and green roofs), BI (e.g., ponds, lakes, and wetlands), and gray infrastructure (e.g., permeable pavements) considering local hot climatic conditions, and available space.[125,165]

Policy and planning

Developing supportive policies, guidelines, regulations, and incentives to integrate GBGI heatwave management measures into urban planning and retrofitting frameworks to align with the sustainability agenda of climate change adaptation and mitigation goals.[166,167]

Implementation

During the implementation phase, the designed GBGI measures should be constructed and installed on selected sites or areas with proper project management and coordination.[168]

Monitoring

After installation, heat reduction potential and other co-benefits of GBGI can be monitored through various parameters, such as temperature, air quality, water management, and vegetation health. which also help to identify any required maintenance or operational issues.[169]

Evaluation

The collected data can be analyzed and evaluated in the context of indicators related to heat performance (e.g., thermal index, reduction in heat stress) or other desired co-benefits aiming to assess the heat managing effectiveness of GBGI measures and urban resilience.[170]

Upscaling and replication

After a successful and positive evaluation of the heat mitigation potential of the implemented GBGI measures, efforts can be made to upscale the approach to larger areas or replicate it in other locations.[171] Lessons learned from previous heat mitigation projects, including best practices for securing resources for GBGI expansion, should be used to guide and enhance future GBGI projects.[172]

In summary, the process of implementing GBGI for heat mitigation and adaptation should involve a rationally structured approach, from stakeholder engagement and feasibility studies to design, policy development, implementation, monitoring, evaluation, and eventual upscaling and replication to exploit their full potential. However, it is important to adapt these specific measures according to the context and needs of each city or region.

KNOWLEDGE GAPS

While GBGI can mitigate UHI effects and heat in general, numerous specific and general knowledge gaps exist that still need to be addressed before GBGI designs can be optimized to deliver maximum cooling benefits. For instance, GBGI such as green roofs and walls, street trees, hedges, wetlands, and lakes were found to have the potential to decrease heat and improve urban micro-climates, yet systematic studies assessing their cooling effectiveness under different climatic conditions (e.g., warm summer day vs. extreme heat day), management regimes (e.g., irrigation vs. no irrigation), urban settings (e.g., residential vs. industrial), and scales remain sparse. Moreover, our study has indicated a generalized knowledge gap referring to integrated approaches to planning, designing, and implementing GBGI. The potential of synergistic effects between combined green and BI elements remains under research. Therefore, further research evaluating the effectiveness of different combinations of blue (e.g., water bodies, wetlands), green (e.g., urban parks, green roofs, street trees), and engineered elements (e.g., cool pavements, reflective surfaces) can help to optimize the design and configuration of these elements to maximize their synergistic effects in heat mitigation.

An understanding of the ideal size, shape, density, and location of GBGI is required to maximize the cooling and other associated co-benefits.[173] Numerical and computational modeling techniques can simulate the complex interactions between the GBGI and urban micro-climates to identify their optimal design and

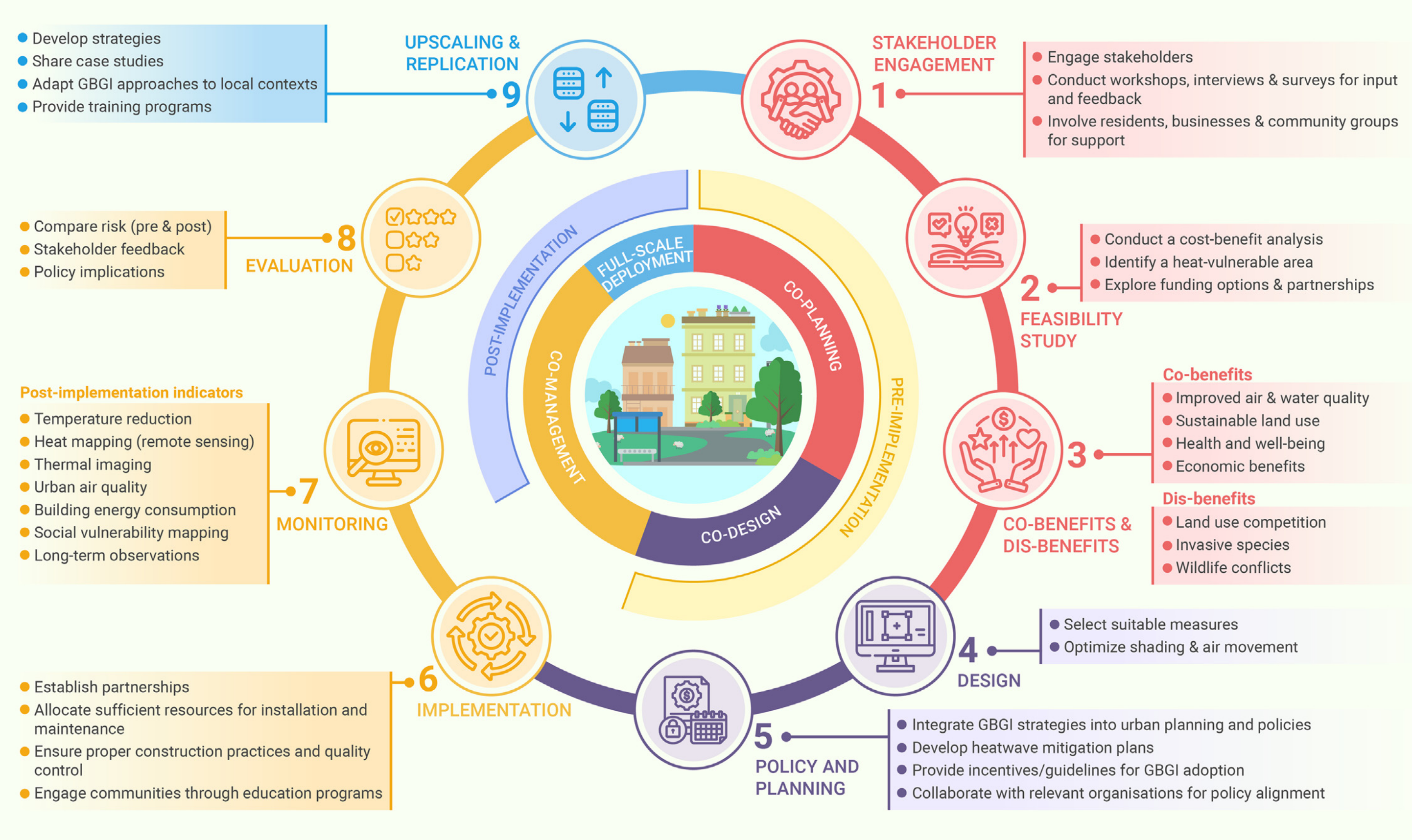

Figure 9. A conceptual framework outlining the implementation of GBGI to mitigate urban overheating A conceptual framework outlinning the GBGI implementation for heat mitigation through four stage processes: co-planning, design, and management, full-scale development, and nine sub-processes.

placement.[125] However, current models often lack the necessary spatial and temporal resolution to capture the fine-scale variations in temperature, relative humidity, and air movement influenced by different infrastructure elements. To address this gap, the development of models that integrate GBGI services, demographic and socio-economic vulnerabilities, and account for the detailed surface characteristics, including vegetation parameters and land use information, is essential, as it will greatly enhance our understanding of the thermal performance of GBGI.

Similarly, the lack of guidelines for GBGI selection and design is another gap that hinders implementation.[34] Research is needed to investigate how the elements such as the growth, health, and type of vegetation, degradation of engineered materials, cost effectiveness, and maintenance practices evolve over time. Such information is required for long-term decision-making in urban planning and policy development to support the wider application, replication, and upscaling of GBGI solutions. Knowledge gaps also exist in identifying effective strategies for engaging and educating communities about the benefits of GBGI,[174,175] as well as promoting sustainable practices, such as water conservation and urban greening, to integrate GBGI into urban design.[176] There is a need for research that explores the governance structures, policy frameworks, stakeholder engagement strategies, and the social, cultural, and economic factors influencing the adoption of GBGI.

The equitable distribution of GBGI and its benefits is an important consideration in complex urban systems.[177,178] Research should examine the potential disparities in access to and the benefits derived from GBGI measures, particularly in disadvantaged, marginalized, and forgotten communities. Evaluating the environmental justice implications of GBGI implementation will help to ensure that the benefits are distributed fairly and address existing social inequities.

Among the reviewed publications (n = 202), the majority (69.8%) did not report other co-benefits for the used GBGI beyond mitigating and adapting to heat. Therefore, assessing the multi-benefits of different GBGI types emerged as a major research gap. In addition, more studies are required to explore the potential of underrepresented GBGI elements (zoological gardens, sparsely vegetated land, shared garden areas, schoolyards, allotments, rivers, railway corridors, ponds, permeable parking, outdoor swimming pools, nursery gardens, heritage gardens, footpaths, flood control channels, estuaries, cycle tracks, canals, bioswales, arable agriculture, golf courses, and sports field) and assess their effectiveness beyond heat mitigation and adaptation, for example, in terms of public health and social wellbeing, or biodiversity implications.[119] Moreover, significant knowledge gaps exist on concerns related to water availability and management of water-based solutions implementation (e.g., irrigation systems, wetlands, ponds, and rain gardens) during extreme heat. These gaps include sustainable water resources, efficient water storage and management techniques, and the impacts of water scarcity on water-based and water-dependent solutions. Research should explore the effects of improved thermal comfort on physical and mental health outcomes, as well as the social and cultural value of green and blue spaces in enhancing community resilience and social cohesion during increasingly hot summers.

Addressing these knowledge gaps through further research, working with stakeholders, and developing the knowledge-sharing platforms and GBGI database[179] can help to overcome barriers and facilitate the effective implementation of GBGI for heat risk adaptation and mitigation.

SUMMARY AND CONCLUSION

This systematic review fills an important gap in the existing literature by its goal of appraisals of the GBGI efficacy for urban heating by bringing together a complex dataset from various studies. This systematic review builds an evidence base on the benefits of GBGI for heat mitigation, associated co-benefits, knowledge gaps, and recommendations for maximizing its potential. A global mapping of GBGI interventions, specifically aimed at urban heat mitigation, was carried out through a systematic literature review that yielded 202 relevant publications. The meta-analysis provided an evidence-based classification for 51 GBGI types. Both the positive and unintended downsides of GBGI measures were discussed along with the mechanisms by which GBGI regulates temperature and mitigates heat. Knowledge gaps in the implementation of GBGI were identified and the critical synthesis of information was used to propose practical recommendations for effective GBGI implementation. Hence, this review paves the

way for future advancements in the realm of GBGI, offering a comprehensive understanding of its potential and implications.

The most common physical mechanisms by which GI regulates urban heating and creates cooler environments were reported to be shading, evapotranspiration, and thermal insulation. This cooling is also associated with decreasing energy consumption in buildings through subsidizing heat gain and decreasing the cooling load. BI is reported to act as a heat sink, regulating temperature through evaporation, and absorbing heat thereby contributing to the mitigation of UHI effects and the creation of more comfortable urban environments.

The analysis of publications indicates a significant increase in research interest and activity in using GBGI for urban heat mitigation worldwide, with a notable focus in Asia and Europe as opposed to South America or Africa due to urban densification and expansion, coupled with rising temperatures affecting cities worldwide and their populations. There are potential regional preferences and priorities in addressing urban heat and its associated impacts. The most studied GBGI types for urban heat mitigation include constructed GI where plant elements are integrated with existing built structures, but also in the form of street trees, parks or green corridors. For private gardens, sports fields, cemeteries and heritage gardens, the cooling potential was less clear, requiring more studies.

GBGI interventions offer co-benefits such as stormwater management and carbon sequestration, as well as a wide range of ecosystem services. However, unintended consequences also exist, such as increased maintenance costs and potential land use conflicts. Optimizing their multifunctional benefits for any specific context is the key. Street trees were reported to have the highest number of co-benefits in addition to their potential effectiveness in heat mitigation and adaptation, followed by green roofs and botanical gardens. Playgrounds, green walls, green roofs, and street trees had the highest number of reported drawbacks such as pollutants trapped in street canyons and allergic rhinitis. This highlights the importance of considering a balanced approach during their implementation for heat mitigation and adaptation.

Among methods, *in situ* monitoring is the most common approach, followed by modeling, RS, and their combinations for evaluating the urban cooling efficacy of GBGI. Regardless of limited data points, *in situ* monitoring offers high temporal resolution, accurate and reliable real-time ground-based measurements for analyzing (a) short-term trends e.g., heatwave and UHI, and (b) long-term historical climate records for validating RS and model data. Modeling offers spatiotemporal variations, but with uncertainties due to assumptions. RS provides extensive spatial coverage but lacks fine-grained details and requires advanced data processing. While there is no universal approach, combined and customized methods can enhance the spatiotemporal resolution for specific objectives, enhancing our ability to respond to extreme weather events and inform sustainable development and climate resilience policies.

Micro-scale GBGI interventions, focusing on built infrastructure, parks, and linear features, play a vital role in mitigating heatwaves in specific urban areas, providing immediate, cost-effective, and community-centric benefits compared with larger scale interventions. The cooling efficiency of GBGI is influenced by location and surroundings, with measurements inside GBGI areas demonstrating greater cooling effects due to mechanisms like evapotranspiration. However, effectiveness diminishes as we are distancing away from GBGI intervention (e.g., outside of the infrastructure). The type of environment also matters, with GBGI in natural surroundings, like parks, enhancing heatwave mitigation more than those in built-up areas. GBGI effectiveness for urban cooling varies based on climatic zones, population density, city area, altitude, and temporal duration of the study. Region-specific strategies, especially in lower density cities and tropical regions, highlight scalability and enhanced effectiveness. Challenges in denser urban settings and decreased effectiveness over longer temporal scales suggest areas for future research in implementing GBGI for urban cooling. Projected climate shifts require tailored GBGI strategies, emphasizing the adaptation of solutions like green walls, lakes, woodlands, and constructed wetlands to enhance urban cooling and resilience locally and at greater scale.

Addressing the existing knowledge gaps through comprehensive research, stakeholder collaboration, and the development of knowledge-sharing platforms and databases is crucial to optimizing the design, implementation, and benefits of GBGI for effective heat risk adaptation and mitigation. More understanding of the synergistic effects of combining green and BI elements is needed to enhance cooling and reduce urban heating. Additionally, gaps exist in water availability and management, GBGI optimal design and placement strategies, long-term performance and maintenance requirements, community engagement and behavior change, equitable distribution of benefits, and supportive policy and governance frameworks.

RECOMMENDATIONS

Ten broader, evidence-based recommendations can be drawn from this study.

- Tailored GBGI typology based on geographical location can aid urban heat mitigation. The GBGI cover in any city should be maximized to decrease UHI effects and the risk of urban overheating. All GBGI types provide cooling benefits, many of them also important co-benefits. Nature needs to be brought back into densifying and expanding cities and any opportunity to expand plant cover on the ground, podiums, walls, and roofs must be taken. All urban development must be nature positive to make our cities and their populations resilient against anticipated stresses and shocks from global climate change. Project-specific optimization of GBGI types is recommended to generate maximum cooling and co-benefits.
- Identifying heat-vulnerable regions can inform targeted GBGI selection to provide heat mitigation solutions. This necessitates a comprehensive evaluation of the heat threat, considering its spatial distribution and severity, the vulnerability of the population, which includes demographic and socio-economic factors, like age and income, and the adaptive capacity, such as education and access to healthcare services. Heat-vulnerable areas might necessitate the creation of communal open green spaces and the adoption of other GBGI types, such as street trees, green roofs, permeable paving, rain gardens, bioswales, and wetlands as appropriate. Where financially feasible, increasing the number of community cooling shelters will be a critical investment to keep the most vulnerable members of an urban population safe.
- A monitoring and evaluation framework is crucial for assessing GBGI's performance in heat mitigation and identifying improvements. Our analyses have shown that the cooling capacity of individual GBGI types varies according to the geographical setting. Hence, it will be important that frameworks are established that will quantify cooling benefits and how these benefits can be optimized through improvements in design and management specific to a geographic region. This will allow decision-makers and operators to make informed decisions, and update projects to deliver the most effective GBGI solution.
- For effective climate-resilient urban heat mitigation, it is crucial to comprehend the characteristics, functionality and constraints of GBGI. This includes understanding the potential uses and benefits of different GBGI, such as recreational parks that can improve air quality and mitigate UHI effects, green roofs, roof gardens, and green walls that enhance biodiversity, reduce energy consumption, manage stormwater, improve air quality, decrease noise pollution and improve aesthetics. Additionally, permeable materials used on sidewalks, parking lots, and roads can facilitate water infiltration, reduce stormwater runoff, and recharge groundwater. However, while implementing GBGI, site-specific factors and local climate conditions should be considered to avoid selecting incompatible GBGI. For example, rain gardens, wetlands, or green roofs with shallow soil depths may not be suitable in areas where water availability is limited and evaporation rates are high.
- The impact of GBGI on urban heat mitigation is largely contingent on spatial scales. These scales can range from individual buildings, where green roofs or walls are used, to entire neighborhoods, where parks and urban forests might be more appropriate, and even city-wide initiatives like green corridors, and extensive tree planting. The existing body of literature indicates a positive, nonlinear (saturating) correlation between the size of GBGIs, especially parks, and their cooling potential. Therefore, customizing GBGI strategies to cater for specific heat risk zones and taking into account distinct location characteristics is imperative for achieving the best results.
- Exploring the potential for integrating various GBGI measures may present unique possibilities for enhancing socio-ecological benefits. Making use of multiple GBGIs, coupled with the inclusion of green or blue elements like irrigation systems and green roofs equipped with rainwater harvesting systems, can amplify cooling benefits. Additionally, this also encourages a range of other co-benefits, including biodiversity

enhancement, habitat connectivity improvement, and bolstering ecosystem resilience.

- It is essential to carefully evaluate potential negative consequences to prevent any unintended side effects when implementing GBGI. For instance, dense trees in narrow or deep street canyons may inadvertently increase exposure to trapped pollutants. In addition, biogenic volatile organic carbons can trigger ozone as a secondary pollutant that can produce respiratory and summer smog issues in hot summers. High maintenance requirements, such as those for botanical, zoological, and heritage gardens must also be considered. Limited access to water for irrigation can decrease the cooling capacity of GBGI. Care must also be taken when implementing new GBGI elements that the integrity of the built infrastructure is not compromised. Being mindful of the potential for unintended ecological impacts and displacement of vulnerable communities is also important. Therefore, before selecting suitable GBGI interventions, it is necessary to assess the local context, environmental conditions, available resources and the budget to ensure their long-term effectiveness and avoid possible drawbacks.
- Comprehensive heat mitigation strategies with straightforward-to-implement practical guidelines are required. Policy frameworks should provide guidance on design standards including building codes and zoning regulations, as well as land use policies. They should provide financial mechanisms to incentivize the adoption of GBGI, particularly in areas with high heat vulnerability, with a special focus on schools, social housing and facilities providing aged care.
- Climate literacy programs and public information campaigns are crucial in promoting positive action on urban heat and GBGI interventions. Incorporating climate-related education into the school curriculum is important for developing a deeper understanding of the causes and consequences of environmental heat, as well as the potential solutions offered by GBGI for urban cooling. In addition to the school curriculum, it is crucial to acknowledge that individuals, both professionals and laypeople, have various levels of awareness, knowledge, attitudes, and behaviors. It is necessary to facilitate progress for all individuals along this spectrum, from left to right. This presents a distinct educational challenge.
- Stakeholder participation plays a pivotal role in promoting the adoption of GBGI solutions in regions susceptible to heat. It is vital to actively involve all stakeholders, including researchers, communities, urban planners, engineers, government bodies, non-profit organizations, and businesses, in the co-creation of GBGI initiatives. A collaborative process to combat the effects of urban heat hotspots not only integrates diverse perspectives but also fosters a sense of shared ownership and collective responsibility. This approach ultimately enhances the effectiveness and acceptance of interventions.

To effectively mitigate urban overheating and harness the co-benefits of GBGI, it is crucial to conduct additional research on the less-studied GBGI types and also in less investigated countries to fill the knowledge gaps. A comprehensive understanding of GBGI's potential in heat mitigation can inform urban planning and design strategies aimed at creating sustainable and resilient cities.

REFERENCES

1. Wang, F., Harindintwali, J.D., Wei, K., et al. (2023). Climate change: Strategies for mitigation and adaptation. Innov. Geosci. ***1***: 100015.
2. Perkins-Kirkpatrick, S.E., and Lewis, S.C. (2020). Increasing trends in regional heatwaves. Nat. Commun. ***11***: 3357.
3. Vogel, M.M., Zscheischler, J., Fischer, E.M., et al. (2020). Development of future heatwaves for different hazard thresholds. J. Geophys. Res. Atmos. ***125***: e2019JD032070.
4. Sahani, J., Kumar, P., Debele, S., et al. (2022). Heat risk of mortality in two different regions of the United Kingdom. Sustain. Cities Soc. ***80***: 103758.
5. Wedler, M., Pinto, J.G., and Hochman, A. (2023). More frequent, persistent, and deadly heat waves in the 21st century over the Eastern Mediterranean. Sci. Total Environ. ***870***: 161883.
6. Meque, A., Pinto, I., Maúre, G., et al. (2022). Understanding the variability of heatwave characteristics in southern Africa. Weather Clim. Extrem. ***38***: 100498.
7. Yenneti, K., Ding, L., Prasad, D., et al. (2020). Urban overheating and cooling potential in Australia: An evidence-based review. Climate ***8***: 126.
8. Li, T., Zhang, C., Ban, J., et al. (2023). Projecting universal health risks under climate change to bridge mitigation and health adaptation objectives. Innovation ***4***: 100427.
9. Yin, Z., Zhou, B., Duan, M., et al. (2023). Climate extremes become increasingly fierce in China. Innovation ***4***: 100406.
10. Perkins, S.E., Alexander, L.V., and Nairn, J.R. (2012). Increasing frequency, intensity and duration of observed global heatwaves and warm spells. Geophys. Res. Lett. ***39***: L20714.
11. Zhang, X., Chen, F., and Chen, Z. (2023). Heatwave and mental health. J. Environ. Manag. ***332***: 117385.
12. Harrington, L.J., and Otto, F.E.L. (2020). Reconciling theory with the reality of African heatwaves. Nat. Clim. Change ***10***: 796–798.
13. EEA (2003). Economic losses from climate-related extremes in Europe (8th EAP). https://www.eea.europa.eu/ims/economic-losses-from-climate-related.
14. WHO. (2023). Word Heath Organisation - Heatwaves. https://www.who.int/health-topics/heatwaves#tab=tab_1.
15. Miranda, V.F.V.V., dos Santos, D.M., Peres, L.F., et al. (2023). Heat stress in South America over the last four decades: a bioclimatic analysis. Theor. Appl. Climatol. ***155***: 911–928. https://doi.org/10.1007/s00704-023-04668-x.
16. Ballester, J., Quijal-Zamorano, M., Méndez Turrubiates, R.F., et al. (2023). Heat-related mortality in Europe during the summer of 2022. Nat. Med. ***29***(7): 1857–1866.
17. Met Office (2022). Unprecedented Extreme Heatwave, 2022. https://shorturl.at/knLNY.
18. NASA (2023). NASA finds June 2023 hottest on record. https://climate.nasa.gov/news/3276/nasa-finds-june-2023-hottest-on-record/#:~:text=June%202023%20was%20the%20hottest,on%20ships%20and%20ocean%20buoys.
19. Wu, Y., Wen, B., Li, S., et al. (2022). Fluctuating temperature modifies heat-mortality association around the globe. Innovation ***3***: 100225.
20. Campbell, S., Remenyi, T.A., White, C.J., et al. (2018). Heatwave and health impact research: A global review. Health Place ***53***: 210–218.
21. Xu, Z., FitzGerald, G., Guo, Y., et al. (2016). Impact of heatwave on mortality under different heatwave definitions: A systematic review and meta-analysis. Environ. Int. ***89–90***: 193–203.
22. Montero, J.C., Mirón, I.J., Criado, J.J., et al. (2010). Comparison between two methods of defining heat waves: a retrospective study in Castile-La Mancha (Spain). Sci. Total Environ. ***408***: 1544–1550.
23. Rasilla, D., Allende, F., Martilli, A., et al. (2019). Heat Waves and Human Well-Being in Madrid (Spain). Atmosphere ***10***: 288.
24. IPCC (2021). International Panel on Climate Change: The Physical Science Basis – the Working Group I Contribution to the Sixth Assessment Report addresses the most up-to-date physical understanding of the climate system and climate change, bringing together the latest advances in climate science. https://www.ipcc.ch/report/sixth-assessment-report-working-group-i/.
25. Wang, F., Harindintwali, J.D., Yuan, Z., Wang, M., Wang, F., Li, S., Yin, Z., Huang, L., Fu, Y., Li, L., et al. (2021). Technologies and perspectives for achieving carbon neutrality. The Innovation ***2***: 100180.
26. Widerynski, S., Schramm, P.J., Conlon, K.C., et al. (2017). Use of cooling centers to prevent heat-related illness: summary of evidence and strategies for implementation. https://shorturl.at/bklqu.
27. Williams, L., Erens, B., Ettelt, S., et al. (2019). Evaluation of the Heatwave Plan for England. Policy Innovation and Evaluation Research Unit. https://shorturl.at/gtGW0.
28. Mücke, H.G., and Litvinovitch, J.M. (2020). Heat extremes, public health impacts, and adaptation policy in Germany. Int. J. Environ. Res. Publ. Health ***17***: 7862.
29. Errett, N.A., Hartwell, C., Randazza, J.M., et al. (2023). Survey of extreme heat public health preparedness plans and response activities in the most populous jurisdictions in the United States. BMC Publ. Health ***23***: 811–11.30.
30. Zonato, A., Martilli, A., Gutierrez, E., et al. (2021). Exploring the effects of rooftop mitigation strategies on urban temperatures and energy consumption. JGR. Atmospheres ***126***: e2021JD035002.
31. Bader, E., Faulkner, M., Gough, M., et al. (2022). The Benefits of Blue-Green Infrastructure. Chapter 2 in the Manual of Blue Green Infrastructure, Institution of Civil Engineers, Thomas Telford, pp. 23–40.
32. Debele, S.E., Kumar, P., Sahani, J., et al. (2019). Nature-based solutions for hydro-meteorological hazards: Revised concepts, classification schemes and databases. Environ. Res. ***179***: 108799.
33. Sahani, J., Kumar, P., Debele, S., et al. (2019). Hydro-meteorological risk assessment methods and management by nature-based solutions. Sci. Total Environ. ***696***: 133936.
34. Kumar, P., Debele, S.E., Sahani, J., et al. (2021a). Nature-based solutions efficiency evaluation against natural hazards: Modelling methods, advantages and limitations. Sci. Total Environ. ***784***: 147058.
35. Almaaitah, T., Appleby, M., Rosenblat, H., et al. (2021). The potential of Blue-Green infrastructure as a climate change adaptation strategy: a systematic literature review. Blue-Green Systems ***3***: 223–248.
36. Le Phuc, C.L., Nguyen, H.S., Dao Dinh, C., et al. (2022). Cooling island effect of urban lakes in hot waves under foehn and climate change. Theor. Appl. Climatol. ***149***: 817–830.
37. Wu, S., Yang, H., Luo, P., et al. (2021). The effects of the cooling efficiency of urban wetlands in an inland megacity: A case study of Chengdu, Southwest China. Build. Environ. ***204***: 108128.
38. Cai, Y., Li, C., Ye, L., et al. (2022). Effect of the roadside tree canopy structure and the surrounding on the daytime urban air temperature in summer. Agric. For. Meteorol. ***316***: 108850.
39. Morabito, M., Crisci, A., Guerri, G., et al. (2021). Surface urban heat islands in Italian metropolitan cities: Tree cover and impervious surface influences. Sci. Total Environ. ***751***: 142334.

40. Fini, A., Frangi, P., Mori, J., et al. (2017). Nature based solutions to mitigate soil sealing in urban areas: Results from a 4-year study comparing permeable, porous, and impermeable pavements. Environ. Res. ***156***: 443–454.
41. Kostadinović, D., Jovanović, M., Bakić, V., et al. (2022). Experimental investigation of summer thermal performance of the green roof system with mineral wool substrate. Build. Environ. ***217***: 109061.
42. Tan, H., Kotamarthi, R., Wang, J., et al. (2023). Impact of different roofing mitigation strategies on near-surface temperature and energy consumption over the Chicago metropolitan area during a heatwave event. Sci. Total Environ. ***860***: 160508.
43. Blanco, I., Schettini, E., and Vox, G. (2019). Predictive model of surface temperature difference between green façades and uncovered wall in Mediterranean climatic area. Appl. Therm. Eng. ***163***: 114406.
44. Coutts, A.M., White, E.C., Tapper, N.J., et al. (2016). Temperature and human thermal comfort effects of street trees across three contrasting street canyon environments. Theor. Appl. Climatol. ***124***: 55–68.
45. Schwaab, J., Meier, R., Mussetti, G., et al. (2021). The role of urban trees in reducing land surface temperatures in European cities. Nat. Commun. ***12***: 6763.
46. Broadbent, A.M., Coutts, A.M., Tapper, N.J., et al. (2018). The microscale cooling effects of water-sensitive urban design and irrigation in a suburban environment. Theor. Appl. Climatol. ***134***: 1–23.
47. Lam, C.K.C., Gallant, A.J., and Tapper, N.J. (2020). Does irrigation cooling effect intensify during heatwaves? A case study in the Melbourne botanic gardens. Urban For. Urban Green. ***55***: 126815.
48. Fogarty, J., van Bueren, M., and Iftekhar, M.S. (2021). Making waves: Creating water sensitive cities in Australia. Water Res. ***202***: 117456.
49. Coutts, A.M., Tapper, N.J., Beringer, J., et al. (2013). Watering our cities: The capacity for Water Sensitive Urban Design to support urban cooling and improve human thermal comfort in the Australian context. Prog. Phys. Geogr. ***37***: 2–28.
50. Völker, S., Baumeister, H., Claßen, T., et al. (2013). Evidence for the temperature-mitigating capacity of urban blue space - A health geographic perspective. Erdkunde ***67***: 355–371.
51. Sanusi, R., Johnstone, D., May, P., et al. (2016). Street orientation and the side of the street greatly influence the microclimatic benefits street trees can provide in summer. J. Environ. Qual. ***45***: 167–174.
52. Qi, J., Ding, L., and Lim, S. (2021). Toward cool cities and communities: A sensitivity analysis method to identify the key planning and design variables for urban heat mitigation techniques. Sustain. Cities Soc. ***75***: 103377.
53. Santamouris, M., Paolini, R., Haddad, S., et al. (2020). Heat mitigation technologies can improve sustainability in cities. A holistic experimental and numerical impact assessment of urban overheating and related heat mitigation strategies on energy consumption, indoor comfort, vulnerability and heat-related mortality and morbidity in cities. Energy Build. ***217***: 110002.
54. Haddad, S., Paolini, R., Ulpiani, G., et al. (2020). The holistic approach to assess co-benefits of local climate mitigation in a hot humid region of Australia. Sci. Rep. ***10***: 14216.
55. Sadeghi, M., Chaston, T., Hanigan, I., et al. (2022). The health benefits of greening strategies to cool urban environments–A heat health impact method. Build. Environ. ***207***: 108546.
56. Sadler, J.P., Grayson, N., Hale, J.D., et al. (2018). The Little Book of Ecosystem Services in the City.
57. Aram, F., Higueras García, E., Solgi, E., et al. (2019). Urban green space cooling effect in cities. Heliyon ***5***: e01339.
58. Rahman, M.A., Stratopoulos, L.M., Moser-Reischl, A., et al. (2020). Traits of trees for cooling urban heat islands: A meta-analysis. Build. Environ. ***170***: 106606.
59. Adegun, O.B., Ikudayisi, A.E., Morakinyo, T.E., et al. (2021). Urban green infrastructure in Nigeria: A review. Scientific African ***14***: e01044.
60. Ampatzidis, P., and Kershaw, T. (2020). A review of the impact of blue space on the urban microclimate. Sci. Total Environ. ***730***: 139068.
61. Jones, L., Anderson, S., Læssøe, J., et al. (2022). A typology for urban green infrastructure to guide multifunctional planning of nature-based solutions. Nature-Based Solutions ***2***: 100041.
62. Ávila-Hernández, A., Simá, E., and Ché-Pan, M. (2023). Research and development of green roofs and green walls in Mexico: A review. Sci. Total Environ. ***856***: 158978.
63. Miyahara, A.A.L., Paixão, C.P., dos Santos, D.R., et al. (2022). Urban dendrochronology toolkit for evidence-based decision-making on climate risk, cultural heritage, environmental pollution, and tree management – A systematic review. Environ. Sci. Pol. ***137***: 152–163.
64. Adnan, M.S.G., Dewan, A., Botje, D., et al. (2022). Vulnerability of Australia to heatwaves: A systematic review on influencing factors, impacts, and mitigation options. Environ. Res. ***213***: 113703.
65. Liu, Z., Cheng, W., Jim, C.Y., et al. (2021). Heat mitigation benefits of urban green and blue infrastructures: A systematic review of modelling techniques, validation and scenario simulation in ENVI-met V4. Build. Environ. ***200***: 107939.
66. Yu, Z., Yang, G., Zuo, S., et al. (2020). Critical review on the cooling effect of urban blue-green space: A threshold-size perspective. Urban For. Urban Green. ***49***: 126630.
67. Heymans, A., Breadsell, J., Morrison, G., et al. (2019). Ecological urban planning and design: A systematic literature review. Sustainability ***11***: 3723.
68. Moher, D., Liberati, A., Tetzlaff, J., et al. (2009). Preferred reporting items for systematic reviews and meta-analyses: the PRISMA statement. Ann. Intern. Med. ***151***: 264–W64.
69. R Core Team (2022). R: A Language and Environment for Statistical Computing (R Foundation for Statistical Computing). https://www.R-project.org/.
70. Beck, H.E., Zimmermann, N.E., McVicar, T.R., et al. (2018). Present and future Köppen-Geiger climate classification maps at 1-km resolution. Sci. Data ***5***: 1–12.
71. (2023). World Cities Database. https://simplemaps.com/data/world-cities.
72. Xi, C., Wang, D., and Cao, S.J. (2023). Impacts of trees-grass area ratio on thermal environment, energy saving, and carbon benefits. Urban Clim. ***47***: 101393.
73. Windbourne, J., Jones, T., Garvey, S., et al. (2020). Tree Transpiration and Urban Temperatures: Current Understanding, Implications, and Future Research Directions. Bioscience ***70***: 576–588.
74. Lu, J., Li, Q., Zeng, L., et al. (2017). A micro-climatic study on cooling effect of an urban park in a hot and humid climate. Sustain. Cities Soc. ***32***: 513–522.
75. Chibuike, E.M., Ibukun, A.O., Abbas, A., et al. (2018). Assessment of green parks cooling effect on Abuja urban microclimate using geospatial techniques. Remote Sens. Appl. ***11***: 11–21.
76. Peng, J., Dan, Y., Qiao, R., et al. (2021). How to quantify the cooling effect of urban parks? Linking maximum and accumulation perspectives. Rem. Sens. Environ. ***252***: 112135.
77. Gunawardena, K.R., Wells, M.J., and Kershaw, T. (2017). Utilising green and bluespace to mitigate urban heat island intensity. Sci. Total Environ. ***584–585***: 1040–1055.
78. Lynn, B.H., and Lynn, I.M. (2020). The impact of cool and green roofs on summertime temperatures in the cities of Jerusalem and Tel Aviv. Sci. Total Environ. ***743***: 140568.
79. Iaria, J., and Susca, T. (2022). Analytic Hierarchy Processes (AHP) evaluation of green roof- and green wall-based UHI mitigation strategies via ENVI-met simulations. Urban Clim. ***46***: 101293.
80. Li, H., Zhao, Y., Sützl, B., et al. (2022). Impact of green walls on ventilation and heat removal from street canyons: Coupling of thermal and aerodynamic resistance. Build. Environ. ***214***: 108945.
81. Castiglia Feitosa, R., and Wilkinson, S.J. (2020). Small-scale experiments of seasonal heat stress attenuation through a combination of green roof and green walls. J. Clean. Prod. ***250***: 119443.
82. Sun, R., Chen, A., Chen, L., et al. (2012). Cooling effects of wetlands in an urban region: The case of Beijing. Ecol. Indicat. ***20***: 57–64.
83. Zhang, Z., Chen, F., Barlage, M., et al. (2022). Cooling Effects Revealed by Modeling of Wetlands and Land-Atmosphere Interactions. Water Resour. Res. ***58***: e2021WR030573.
84. Hathway, E.A., and Sharples, S. (2012). The interaction of rivers and urban form in mitigating the Urban Heat Island effect: A UK case study. Build. Environ. ***58***: 14–22.
85. Wu, C., Li, J., Wang, C., et al. (2019). Understanding the relationship between urban blue infrastructure and land surface temperature. Sci. Total Environ. ***694***: 133742.
86. McCormick, K., Anderberg, S., Coenen, L., et al. (2013). Advancing sustainable urban transformation. J. Clean. Prod. ***50***: 1–11.
87. Lafortezza, R., and Sanesi, G. (2019). Nature-based solutions: Settling the issue of sustainable urbanization. Environ. Res. ***172***: 394–398.
88. Berdejo-Espinola, V., Suárez-Castro, A.F., Amano, T., et al. (2021). Urban green space use during a time of stress: A case study during the COVID-19 pandemic in Brisbane, Australia. People Nat. ***3***: 597–609.
89. Lopez, B., Kennedy, C., Field, C., et al. (2021). Who benefits from urban green spaces during times of crisis? Perception and use of urban green spaces in New York City during the COVID-19 pandemic. Urban For. Urban Green. ***65***: 127354.
90. Pouso, S., Borja, Á., Fleming, L.E., et al. (2021). Contact with blue-green spaces during the COVID-19 pandemic lockdown beneficial for mental health. Sci. Total Environ. ***756***: 143984.
91. Mabon, L., and Shih, W.Y. (2021). Urban greenspace as a climate change adaptation strategy for subtropical Asian cities: A comparative study across cities in three countries. Global Environ. Change ***68***: 102248.
92. Jaung, W., Carrasco, L.R., Shaikh, S.F.E.A., et al. (2020). Temperature and air pollution reductions by urban green spaces are highly valued in a tropical city-state. Urban For. Urban Green. ***55***: 126827.
93. MHURDPRC (2016). Ministry of Housing and Urban-Rural Development, & Ministry of Environmental Protection, the People's Republic of China Result of National Inventory about Urban River Pollution. http://www.mohurd.gov.cn/wjfb/201602/t20160219_226677.html.2016.
94. Sikora, A. (2021). European Green Deal–legal and financial challenges of the climate change. Era Forum ***21***: 681–697.
95. Debele, S.E., Leo, L.S., Kumar, P., et al. (2023). Nature-based Solutions Can Help Reduce the Impact of Natural Hazards: A Global Analysis of NBS Case Studies. Sci. Total Environ. ***902***: 165824.
96. Williams, P.A., Simpson, N.P., Totin, E., et al. (2021). Feasibility assessment of climate change adaptation options across Africa: an evidence-based review. Environ. Res. Lett. ***16***: 073004.
97. Schroeter, B., Zingraff-Hamed, A., Ott, E., et al. (2021). The knowledge transfer potential of online data pools on nature-based solutions. Sci. Total Environ. ***762***: 143074.
98. Kottek, M., Grieser, J., Beck, C., et al. (2006). World Map of the Köppen-Geiger climate classification updated. Metz. ***15***: 259–263.
99. Alikhani, S., Nummi, P., and Ojala, A. (2021). Urban wetlands: A review on ecological and cultural values. Water ***13***: 3301.
100. Johnson, R., and Wang, L. (2021). Green Infrastructure Efficiency in Continental Climates: Insights from Green Walls and Botanical Gardens. Environ. Sci. Technol. ***28***: 451–468.
101. Meerow, S., Natarajan, M., and Krantz, D. (2021). Green infrastructure performance in arid and semi-arid urban environments. Urban Water J. ***18***: 275–285.
102. Tanaka, Y., and Silva, M. (2023). Micro-scale Impact of Roof Gardens in Tropical Climates: A Study on Temperature Reduction in Densely Built-up Areas. Urban Clim. ***18***: 88–101.
103. Manoli, G., Fatichi, S., Schläpfer, M., et al. (2019). Magnitude of urban heat islands largely explained by climate and population. Nature ***573***: 55–60.

104. Santamouris, M., Haddad, S., Saliari, M., et al. (2018). On the energy impact of Urban Heat Island in Sydney: Climate and energy potential of mitigation technologies. Energy Build. **166**: 154–164.
105. Sailor, D.J. (2011). A review of methods for estimating anthropogenic heat and moisture emissions in the urban environment. Int. J. Climatol. **31**: 189–199.
106. Nouri, H., Beecham, S., and Hassanli, A.M. (2019). Urban heat island and its challenges in tropical climates: A case study in Darwin, Australia. Urban For. Urban Green. **40**: 312–322.
107. Gill, S.E., Handley, J.F., Ennos, A.R., et al. (2007). Adapting cities for climate change: the role of the green infrastructure. Built. Environ. **33**: 115–133.
108. Yin, J., Wu, X., Shen, M., et al. (2019). Impact of urban greenspace spatial pattern on land surface temperature: a case study in Beijing metropolitan area, China. Landsc. Ecol. **34**: 2949–2961.
109. Grimmond, C.S.B., Blackett, M., Best, M.J., et al. (2011). Initial results from Phase 2 of the international urban energy balance model comparison. Int. J. Climatol. **31**: 244–272.
110. Imhoff, M.L., Zhang, P., Wolfe, R.E., et al. (2010). Remote sensing of the urban heat island effect across biomes in the continental USA. Rem. Sens. Environ. **114**: 504–513.
111. DeFries, R.S., Foley, J.A., and Asner, G.P. (2004). Land-use choices: Balancing human needs and ecosystem function. Front. Ecol. Environ. **2**: 249–257.
112. Seto, K.C., Güneralp, B., and Hutyra, L.R. (2012). Global forecasts of urban expansion to 2030 and direct impacts on biodiversity and carbon pools. Proc. Natl. Acad. Sci. USA **109**: 16083–16088.
113. Kuang, W., Liu, Y., Dou, Y., et al. (2015). What are hot and what are not in an urban landscape: quantifying and explaining the land surface temperature pattern in Beijing, China. Landsc. Ecol. **30**: 357–373.
114. Zhou, W., Yu, W., Zhang, Z., et al. (2023). How can urban green spaces be planned to mitigate urban heat island effect under different climatic backgrounds? A threshold-based perspective. Sci. Total Environ. **890**: 164422.
115. Borduas, N., and Donahue, N.M. (2018). The natural atmosphere. Green Chem. 131–150.
116. Oke, T.R. (1982). The Energetic Basis of the Urban Heat Island (Quarterly Journal of the Royal Meteorological Society).
117. Grimmond, S.U.E. (2007). Urbanization and global environmental change: local effects of urban warming. Geogr. J. **173**: 83–88.
118. Aflaki, A., Mirnezhad, M., Ghaffarianhoseini, A., et al. (2017). Urban heat island mitigation strategies: A state-of-the-art review on Kuala Lumpur, Singapore and Hong Kong. Cities **62**: 131–145.
119. Ommer, J., Bucchignani, E., Leo, L.S., et al. (2022). Quantifying co-benefits and disbenefits of Nature-based Solutions targeting Disaster Risk Reduction. Int. J. Disaster Risk Reduc. **75**: 102966.
120. Kumar, P., Abhijith, K.V., and Barwise, Y. (2019). Implementing Green Infrastructure for Air Pollution Abatement: General Recommendations for Management and Plant Species Selection. https://doi.org/10.6084/m9.figshare.8198261.v4.
121. Barwise, Y., and Kumar, P. (2020). Designing vegetation barriers for urban air pollution abatement: a practical review for appropriate plant species selection. NPJ Clim. Atmos. Sci. **3**: 12.
122. Song, J., and Hart, J.D. (2010). Bootstrapping in a high dimensional but very low-sample size problem. J. Stat. Comput. Simulat. **80**: 825–840.
123. Grey, C.P., and Tarascon, J.M. (2016). Sustainability and in situ monitoring in battery development. Nat. Mater. **16**: 45–56.
124. Sowińska-Świerkosz, B., and García, J. (2021). A new evaluation framework for nature-based solutions (NBS) projects based on the application of performance questions and indicators approach. Sci. Total Environ. **787**: 147615.
125. Kumar, P., Debele, S.E., Sahani, J., et al. (2021b). An overview of monitoring methods for assessing the performance of nature-based solutions against natural hazards. Earth Sci. Rev. **217**: 103603.
126. Li, X., Xu, X., Wang, X., et al. (2021). Assessing the effects of spatial scales on regional evapotranspiration estimation by the SEBAL model and multiple satellite datasets: a case study in the Agro-Pastoral Ecotone, northwestern China. Rem. Sens. **13**: 1524.
127. Kianmehr, A., Lim, T.C., and Li, X. (2023). Comparison of different spatial temperature data sources and resolutions for use in understanding intra-urban heat variation. Sustain. Cities Soc. **96**: 104619.
128. Dong, J., Lin, M., Zuo, J., et al. (2020). Quantitative study on the cooling effect of green roofs in a high-density urban Area—A case study of Xiamen, China. J. Clean. Prod. **255**: 120152.
129. Fleck, R., Gill, R.L., Saadeh, S., et al. (2022). Urban green roofs to manage rooftop microclimates: A case study from Sydney, Australia. Build. Environ. **209**: 108673.
130. Xu, H., Chen, H., Zhou, X., et al. (2020). Research on the relationship between urban morphology and air temperature based on mobile measurement: A case study in Wuhan, China. Urban Clim. **34**: 100671.
131. Hong, F., Zhan, W., Göttsche, F.M., et al. (2021). A simple yet robust framework to estimate accurate daily mean land surface temperature from thermal observations of tandem polar orbiters. Rem. Sens. Environ. **264**: 112612.
132. Harlan, S.L., Brazel, A.J., Prashad, L., et al. (2006). Neighborhood microclimates and vulnerability to heat stress. Soc. Sci. Med. **63**: 2847–2863.
133. Nowak, D.J., Crane, D.E., and Stevens, J.C. (2006). Air pollution removal by urban trees and shrubs in the United States. Urban For. Urban Green. **4**: 115–123.
134. Berardi, U., GhaffarianHoseini, A., and GhaffarianHoseini, A. (2014). State-of-the-art analysis of the environmental benefits of green roofs. Appl. Energy **115**: 411–428.
135. Kabisch, N., and Haase, D. (2014). Green justice or just green? Provision of urban green spaces in Berlin, Germany. Landsc. Urban Plann. **122**: 129–139.
136. Smithers, R.J., Doick, K.J., Burton, A., et al. (2018). Comparing the relative abilities of tree species to cool the urban environment. Urban Ecosyst. **21**: 851–862.
137. Richter, R., Ballasus, H., Engelmann, R.A., et al. (2022). Tree species matter for forest microclimate regulation during the drought year 2018: disentangling environmental drivers and biotic drivers. Sci. Rep. **12**: 17559.
138. Rahman, M.A., Moser, A., Rötzer, T., et al. (2017). Microclimatic differences and their influence on transpirational cooling of Tilia cordata in two contrasting street canyons in Munich, Germany. Agric. For. Meteorol. **232**: 443–456.
139. Probst, N., Bach, P.M., Cook, L.M., et al. (2022). Blue Green Systems for urban heat mitigation: mechanisms, effectiveness and research directions. Blue-Green Systems **4**: 348–376.
140. Akbari, H., Pomerantz, M., and Taha, H. (2001). Cool surfaces and shade trees to reduce energy use and improve air quality in urban areas. Sol. Energy **70**: 295–310.
141. Chang, C.R., and Li, M.H. (2014). Effects of urban parks on the local urban thermal environment. Urban For. Urban Green. **13**: 672–681.
142. Suits, K., Annus, I., Kändler, N., et al. (2023). Overview of the (Smart) Stormwater Management around the Baltic Sea. Water **15**: 1623.
143. Jezzini, Y., Assaf, G., and Assaad, R.H. (2023). Models and Methods for Quantifying the Environmental, Economic, and Social Benefits and Challenges of Green Infrastructure: A Critical Review. Sustainability **15**: 7544.
144. Aghamohammadi, N., Fong, C.S., Farid, N.D.N., et al. (2022). Heat and Mental Health in Cities. In Urban Overheating: Heat Mitigation and the Impact on Health (Springer Nature Singapore), pp. 81–107.
145. Dore, M.H.I. (2005). Climate change and changes in global precipitation patterns: what do we know? Environ. Int. **31**: 1167–1181.
146. Zhou, X., Okaze, T., Ren, C., et al. (2020). Evaluation of urban heat islands using local climate zones and the influence of sea-land breeze. Sustain. Cities Soc. **55**: 102060.
147. Puppim de Oliveira, J.A., Bellezoni, R.A., Shih, W.Y., et al. (2022). Innovations in Urban Green and Blue Infrastructure: Tackling local and global challenges in cities. J. Clean. Prod. **362**: 132355.
148. EEA (2012). Climate Change, Impacts, and Vulnerability in Europe 2012. https://www.eea.europa.eu/publications/climate-impacts-and-vulnerability-2012/.
149. Benedict, M.A., and McMahon, E.T. (2002). Green Infrastructure: Smart Conservation for the 21st Century. Renew. Resour. J. **20**: 12–17.
150. Bullock, J.M., Aronson, J., Newton, A.C., et al. (2011). Restoration of ecosystem services and biodiversity: conflicts and opportunities. Trends Ecol. Evol. **26**: 541–549.
151. EEA (2017). European Environment Agency. Climate Change, Impacts and Vulnerability in Europe 2016. https://www.eea.europa.eu/publications/climate-change-impacts-and-vulnerability-2016.
152. Mentens, J., Raes, D., and Hermy, M. (2006). Green roofs as a tool for solving the rainwater runoff problem in the urbanized 21st century? Landsc. Urban Plann. **77**: 217–226.
153. Čížková, H., Květ, J., Comín, F.A., et al. (2013). Actual state of European wetlands and their possible future in the context of global climate change. Aquat. Sci. **75**: 3–26.
154. Brudermann, T., and Sangkakool, T. (2017). Green roofs in temperate climate cities in Europe–An analysis of key decision factors. Urban For. Urban Green. **21**: 224–234.
155. Zhou, Y., Zhao, H., Mao, S., et al. (2022). Studies on urban park cooling effects and their driving factors in China: Considering 276 cities under different climate zones. Build. Environ. **222**: 109441.
156. Tao, S., Fang, J., Ma, S., et al. (2020). Changes in China's lakes: Climate and human impacts. Natl. Sci. Rev. **7**: 132–140.
157. Zhou, D., Liu, Y., Hu, S., et al. (2019). Assessing the hydrological behaviour of large-scale potential green roofs retrofitting scenarios in Beijing. Urban For. Urban Green. **40**: 105–113.
158. Hamed, M.M., Nashwan, M.S., Shahid, S., et al. (2023). Future Köppen-Geiger climate zones over Southeast Asia using CMIP6 Multimodel Ensemble. Atmos. Res. **283**: 106560.
159. Mitsch, W.J., Zhang, L., Stefanik, K.C., et al. (2012). Creating wetlands: primary succession, water quality changes, and self-design over 15 years. Bioscience **62**: 237–250.
160. Rogers, C.D.F., Makana, L.O., Leach, J.M., et al. (2023). The Little Book of Theory of Change for Infrastructure and Cities (University of Birmingham).
161. Sherman, M.H., and Ford, J. (2014). Stakeholder engagement in adaptation interventions: an evaluation of projects in developing nations. Clim. Pol. **14**: 417–441.
162. O'Brien, R.M., Phelan, T.J., Smith, N.M., et al. (2021). Remediation in developing countries: A review of previously implemented projects and analysis of stakeholder participation efforts. Crit. Rev. Environ. Sci. Technol. **51**: 1259–1280.
163. Coutts, J., White, T., Blackett, P., et al. (2017). Evaluating a space for co-innovation: Practical application of nine principles for co-innovation in five innovation projects. Outlook Agric. **46**: 99–107.
164. Curt, C., Di Maiolo, P., Schleyer-Lindenmann, A., et al. (2022). Assessing the environmental and social co-benefits and disbenefits of natural risk management measures. Heliyon **8**: e12465.
165. Dumitru, A., Frantzeskaki, N., and Collier, M. (2020). Identifying principles for the design of robust impact evaluation frameworks for nature-based solutions in cities. Environ. Sci. Pol. **112**: 107–116.
166. Green Deal, E. (2021). The European Green Deal and cohesion policy. https://www.europarl.europa.eu/RegData/etudes/BRIE/2021/698058/EPRS_BRI(2021)698058_EN.pdf.
167. Davies, C., Chen, W.Y., Sanesi, G., et al. (2021). The European Union roadmap for implementing nature-based solutions: A review. Environ. Sci. Pol. **121**: 49–67.
168. Di Pirro, E., Sallustio, L., Sgrigna, G., et al. (2022). Strengthening the implementation of national policy agenda in urban areas to face multiple environmental stressors: Italy as a case study. Environ. Sci. Pol. **129**: 1–11.

169. Augusto, B., Roebeling, P., Rafael, S., et al. (2020). Short and medium-to long-term impacts of nature-based solutions on urban heat. Sustain. Cities Soc. ***57***: 102122.
170. Frantzeskaki, N. (2019). Seven lessons for planning nature-based solutions in cities. Environ. Sci. Pol. ***93***: 101–111.
171. Cortinovis, C., Olsson, P., Boke-Olén, N., et al. (2022). Scaling up nature-based solutions for climate-change adaptation: Potential and benefits in three European cities. Urban For. Urban Green. ***67***: 127450.
172. Rosemartin, A., Crimmins, T.M., Gerst, K.L., et al. (2023). Lessons learned in knowledge co-production for climate-smart decision-making. Environ. Sci. Pol. ***141***: 178–187.
173. Graça, M., Cruz, S., Monteiro, A., et al. (2022). Designing urban green spaces for climate adaptation: A critical review of research outputs. Urban Clim. ***42***: 101126.
174. Topal, H.F., Hunt, D.V.L., and Rogers, C.D.F. (2021). Exploring urban sustainability understanding and behavior: A systematic review towards a conceptual framework. Sustainability ***13***: 1139.
175. Kumar, P., Sahani, J., Rawat, N., et al. (2023). Using empirical science education in schools to improve climate change literacy. Renew. Sustain. Energy Rev. ***178***: 113232.
176. Kaur, R., and Gupta, K. (2022). Blue-green infrastructure (BGI) network in urban areas for sustainable storm water management: A geospatial approach. City and Environment Interactions ***16***: 100087.
177. De Sousa Silva, C., Viegas, I., Panagopoulos, T., et al. (2018). Environmental justice in accessibility to green infrastructure in two European cities. Land ***7***: 134.
178. Amaral, M.H., Benites-Lazaro, L.L., Antonio de Almeida Sinisgalli, P., et al. (2021). Environmental injustices on green and blue infrastructure: Urban nexus in macrometropolitan territory. J. Clean. Prod. ***289***: 125829.
179. GeoIKP. (2022). Platform for Nature-based Solutions. https://geoikp.operandum-project.eu/.

ACKNOWLEDGMENTS

This work has been commissioned by the UKRI (EPSRC, NERC, AHRC) and funded by RECLAIM Network Plus project (EP/W034034/1; EP/W033984) under its synthesis review series. The following authors acknowledge the funding received through their grants: P.K. and L.J. (NE/X002799/1; NE/X002772/1); L.J. (H2020 REGREEN, EU Grant agreement No. 821016; 2021YFE93100); G.M.L. (FAPESP 2019/08783-0); C.D.F.R. (EP/R017727); L.M. (ARC Grant No. IC220100012); H.G. (RGC Grant No. C5024-21G); M.F.A. and E.D.F. (FAPESP Grant No. 2016/18438-0; 2022/02365-5); S.J.C. (NSFC Grant No. 52225005), R.Y. (NSFC Grant No. 52278090); F.W. (NKP Grant No. 2020YFC180700); J.E. (NE/X000443/1); and F.C. (NE/M010961/1; NE/V002171/1). The authors thank Andrea Sofia Majjul Fajardo for her contribution to the initial design of certain figures. We also thank the team members of GCARE and its Guildford Living Lab (GLL), as well as the participants in the RECLAIM Network Plus Horizon Scanning Workshop.

AUTHOR CONTRIBUTIONS

P.K.: conceptualization, methods, supervision, project administration, funding, analysis, writing - original draft, writing - reviewing and editing; S.D.: conceptualization, methods, data extraction, data analysis (figures, tables), writing - original draft, writing - reviewing and editing; S.K.: data extraction, writing - reviewing and editing; C.H.: data extraction, writing - reviewing and editing; J.S.: writing - reviewing and editing; N.A.: writing - review and editing; M.d.F.A.: writing - review and editing; M.A.: writing - review and editing; K.B.: writing - review and editing; N.C.: writing - review and editing; S.C.: writing - review and editing; F.C.: writing - review and editing; J.E.E.: writing - review and editing; D.F.: writing - review and editing; E.D.d.F.: writing - review and editing; H.G.: writing - review and editing; M.C.H.: writing - review and editing; M.K.: writing - review and editing; T.R.K.: writing - review and editing; S.L.: writing - review and editing; G.M.L.: writing - review and editing; S.K.M.: writing - review and editing; L.M.: writing - review and editing; R.P.: writing - review and editing; C.R.: writing - review and editing; R.Y.: writing - review and editing; F.W.: writing - review and editing; J.W.: writing - review and editing; L.J.: conceptualization, methods, writing - review and editing. The authors' names appear in alphabetical order between the core writing team and the anchor authors. All authors commented on the draft manuscript and assisted in the conceptual development of the text, tables, figures, and the overall cohesiveness and proofreading of the paper.

DECLARATION OF INTERESTS

The authors declare no competing interests.

SUPPLEMENTAL INFORMATION

It can be found online at https://doi.org/10.1016/j.xinn.2024.100588.

LEAD CONTACT WEBSITE

https://www.surrey.ac.uk/people/prashant-kumar.

The Innovation, Volume *5*

Supplemental Information

Urban heat mitigation by green and blue infrastructure: Drivers, effectiveness, and future needs

Prashant Kumar, Sisay E. Debele, Soheila Khalili, Christos H. Halios, Jeetendra Sahani, Nasrin Aghamohammadi, Maria de Fatima Andrade, Maria Athanassiadou, Kamaldeep Bhui, Nerea Calvillo, Shi-Jie Cao, Frederic Coulon, Jill L. Edmondson, David Fletcher, Edmilson Dias de Freitas, Hai Guo, Matthew C. Hort, Madhusudan Katti, Thomas Rodding Kjeldsen, Steffen Lehmann, Giuliano Maselli Locosselli, Shelagh K. Malham, Lidia Morawska, Rajan Parajuli, Christopher D.F. Rogers, Runming Yao, Fang Wang, Jannis Wenk, and Laurence Jones

Supplementary Information (SI)

for

Urban heat mitigation by green and blue infrastructure: a review of drivers, effectiveness, and future needs

Prashant Kumar[1,2,10,*], Sisay Debele[1], Soheila Khalili[1], Christos H. Halios[3], Jeetendra Sahani[1], Nasrin Aghamohammadi[4,5], Maria de Fatima Andrade[6], Maria Athanassiadou[7], Kamaldeep Bhui[8] , Nerea Calvillo[9], Shi-Jie Cao[1,10], Frederic Coulon[11], Jill L. Edmondson[12], David Fletcher[13], Edmilson Dias de Freitas[6], Hai Guo[14], Matthew C Hort[7], Madhusudan Katti[15], Thomas Rodding Kjeldsen[16], Steffen Lehmann[17], Giuliano Maselli Locosselli[18], Shelagh K Malham[19], Lidia Morawska[1,20], Rajan Parajuli[21], , Christopher DF Rogers[22], Runming Yao[3,23], Fang Wang[24,25], Jannis Wenk[16], Laurence Jones[13,26]

[1]Global Centre for Clean Air Research (GCARE), School of Sustainability, Civil and Environmental Engineering, Faculty of Engineering and Physical Sciences, University of Surrey, Guildford GU2 7XH, United Kingdom

[2]Institute for Sustainability, University of Surrey, Guildford GU2 7XH, Surrey, United Kingdom

[3]School of Built Environment, University of Reading, Shinfield Rd, Whiteknights Rd, Reading RG6 6BU, United Kingdom

[4]School Design and the Built Environment, Curtin University Sustainability Policy Institute, Kent St, Bentley, 6102, Western Australia

[5]Harry Butler Institute, Murdoch University, Murdoch, 6150 Western Australia

[6]Atmospheric Sciences Department, Institute of Astronomy, Geophysics and Atmospheric Sciences, University of Sao Paulo, 05508-090, Sao Paulo, Brazil

[7]Met Office, FitzRoy Road, Exeter, EX1 3PB, United Kingdom

[8]Department of Psychiatry and Nuffield Department of Primary Care Health Sciences, Wadham College, University of Oxford, Oxford, United Kingdom

[9]Centre for Interdisciplinary Methodologies, University of Warwick, United Kingdom

[10]School of Architecture, Southeast University, 2 Sipailou, Nanjing, 210096, China

[11]Cranfield University, School of Water, Environment and Energy, Cranfield, MK43 0AL, United Kingdom

[12]Plants, Photosynthesis, Soil Cluster, School of Biosciences, University of Sheffield, Sheffield S10 2TN, United Kingdom

[13]UK Centre for Ecology & Hydrology, Environment Centre Wales, Deiniol Road, Bangor LL57 2UW, United Kingdom

[14]Air Quality Studies, Department of Civil and Environmental Engineering, The Hong Kong Polytechnic University, Hong Kong, China

[15]Department of Forestry and Environmental Resources, Faculty Excellence Program for Leadership in Public Science, North Carolina State University, Chancellor', Raleigh, NC, 27695, USA

[16]Departments of Architecture & Civil Engineering, and Chemical Engineering, University of Bath, Bath BA2 7AY, United Kingdom

[17]School of Architecture, University of Nevada, Las Vegas, NV 89154, USA

[18]Department of Tropical Ecosystems Functioning, Center of Nuclear Energy in Agriculture, University of São Paulo, Piracicaba, 13416-000, Sao Paulo, Brazil

[19]School of Ocean Sciences, Bangor University, Menai Bridge, Anglesey, LL59 5 AB, United Kingdom

[20]*International Laboratory for Air Quality and Health, Science and Engineering Faculty, Queensland University of Science and Technology, Queensland, Australia*

[21]*Department of Forestry and Environmental Resources, North Carolina State University, Raleigh NC 27695, USA*

[22]*Department of Civil Engineering, School of Engineering, University of Birmingham, Edgbaston, Birmingham B15 2TT, United Kingdom*

[23]*Joint International Research Laboratory of Green Buildings and Built Environments, School of the Civil Engineering, Chongqing University, China*

[24]*State Key Laboratory of Soil and Sustainable Agriculture, Institute of Soil Science, Chinese Academy of Sciences, Nanjing 210008, China*

[25]*University of Chinese Academy of Sciences, Beijing 100049, China*

[26]*Liverpool Hope University, Department of Geography and Environmental Science, Hope Park, Liverpool L16 9JD, United Kingdom*

This document includes:

Sections S1-S2

Figures S1-S7.

Tables S1-S9

S1 Methodology

We screened studies based on the following criteria: (a) addressing urban heat mitigation using one or more GBGI types, (b) clear identification of at least one GBGI sub-category under investigation, (c) a clear link between the primary GBGI category and heat mitigation performance, including temperature reduction and associated co-benefits, and (d) accessibility of full-text articles from the databases for further review and data extraction.

After removing duplicates, 25,974 publications that didn't meet these criteria were eliminated, leaving 1,512 publications for further screening (Figure 2b). We retrieved and assessed the full text of each paper for eligibility (Figure 2c). Articles not meeting inclusion criteria, such as those mainly focusing on heat causes and impacts, methodological aspects of heat mitigation without a direct GBGI connection, or discussions limited to general green and blue spaces without specific GBGI details and cooling effectiveness, were excluded. After this additional screening, 1,250 more papers were discarded, resulting in 262 publications for final review. Out of these, 60 more publications were excluded due to insufficient performance reporting or a failure to mention the GBGI used. In the end, 202 publications (1.8% of the originally identified 27,486 publications) were chosen for meta-analysis and further consideration in this review (Figure 2d, e). First we cataloged data from the selected studies, extracting information from 202 of them, including (1) the study's location (site, city, country, and region), (2) the specific type of GBGI, (3) the nature of the study (monitoring, modeling, remote sensing, or a combination), (4) whether single or multiple GBGIs were studied, (5) qualitative or quantitative data on co-benefits, air temperature, land surface temperature (LST) reduction (in °C), and day or night air temperature reduction (in °C), (6) any identified knowledge gaps, and (7) key findings. We developed a data form to capture this essential information from the selected studies (Table S1). This information was used to address key objectives, including when and where previous articles were published, the types of GBGIs used for urban heat mitigation, and the nature of co-benefits and maximum temperature reductions reported. Following this, we analysed and synthesised the data from selected studies to address the specific review objectives. The information on the benefits of various GBGI subcategories was examined using descriptive statistics with R-project software. To create an evaluation framework for GBGI types and their services in addressing

heat-related challenges and associated co-benefits, we used a min-max normalization approach, scaling the data from 1 (none) to 6 (very high) (see Table 2). This standardized method was then applied to the relevant publications within each GBGI category. Out of the 202 publications, 64.7% primarily focused on heat mitigation as their main ecosystem service, while the remaining 35.3% discussed co-benefits alongside direct heat mitigation benefits.

The review proceeds with a summary of how GBGI mitigates heat stress, followed by mapping study origins, exploring GBGI interventions, and presenting evidence of their cooling advantages and co/dis-benefits. It also provides a conceptual framework for GBGI implementation and addresses existing knowledge gaps. The review concludes with major findings and recommendations for effective GBGI implementation to mitigate urban heat.
Five additional co-benefits are identified, including enhanced recreational opportunities and improvements in air/water quality.

S1.1 GBGI classification, scope, and outline

The conceptualisation and classification of GBGI types can differ due to varying interpretations from country to country, contingent on research contexts and the primary objective of studies. Sustainability objectives are selectively applied within different GBGI development frameworks, leading to a plethora of definitions and interpretations.[1] These variations depend on whether GBGI is applied to ecological resources[2] or includes natural green spaces, or is confined to highly altered landscapes intended for public benefit.[3] Consequently, it becomes challenging to specifically distinguish between green and grey infrastructure, such as cycle paths passing through green areas that provide additional recreational benefits. Often, scientists categorise non-ecological resources as GBGI, for example, permeable pavements, rainwater barrels, and rain gardens. For this review, we

adopt the GBGI classification presented by Jones et al.[4] and expand it further by introducing two new categories, mixed (blue-green) and backyard irrigation,[5-7] giving a total of 51 GBGI types. This classification uses a green-blue-grey continuum to cover natural green or blue, engineered green, blue, or hybrid (green-blue-grey) combinations. The typology was designed to flexibly incorporate all GBGI types within a typical urban environment. Within the scope of this review, we examined 51 GBGI types grouped under 10 broad categories: gardens, parks, amenity areas, linear features/routes, constructed GI on infrastructure, hybrid GI (for water), water bodies, other non-sealed urban areas, other public spaces, and mixed – (green-blue).

The task of examining and integrating the diverse benefits of GBGIs was challenging due to their complex interactions and discipline-specific applications. For instance, public health is directly or indirectly tied to almost all evaluated benefits, encompassing psychological, social, and economic aspects.[8] Therefore, this study focuses on the direct GBGI cooling benefits (heat mitigation), whilst indirect benefits such as management of other natural hazards (floods, droughts), the creation of new infrastructure (such as interconnected green infrastructure corridors to support active travel; Rogers and Hunt[9]) or associated social costs avoided by using specific GBGI types are considered as a secondary objective. In the course of this review, five more co-benefits were identified: (1) enhanced recreational opportunities, (2) ambient noise reduction, (3) flood and drought risk mitigation, (4) improvements in air/water quality, and (5) biodiversity (Section 3.3.2). Detailed GBGI design and implementation principles, along with global GBGI challenges, have been covered in earlier reviews (Table 1) and therefore were beyond the scope of this paper.

The review commences with a concise summary of how GBGI mitigates heat stress (Section 3), followed by a spatial and temporal mapping of study origins, an exploration of various GBGI interventions, and a presentation of quantitative evidence supporting the direct cooling advantages and other co/dis-benefits (Section 4). Section 5 provides a nine-stage conceptual framework for GBGI implementation for heat mitigation based on the qualitative analysis of the reviewed literature and discusses practical recommendations for the design, implementation, monitoring, evaluation, and upscaling of GBGI to mitigate heat risks. Section 6 highlights the existing knowledge gaps. The review culminates with major conclusions and lays out a series of recommendations for the effective implementation of GBGI to mitigate urban heat (Section 7).

S1.2 Search and selection of relevant studies

The goal of this review was to offer an in-depth assessment review and analysis of GBGI's functions and benefits concerning urban cooling, as well as their potential co-benefits and drawbacks. This approach led to an expansion of the scope beyond that of previous reviews covered, enables us to uncover overlooked geographical patterns and examine the temporal trends in the origin of studies, and knowledge voids in the existing literature. PRISMA methodology was adopted for this systematic review.[10] Figure 1 provides a flowchart depicting our search and evaluation methodology, including its resultant findings. Our literature search consisted of five stages:

(1) Development of search terms: To identify a comprehensive range of studies related to urban heat mitigation, relevant search terms were determined based on research gaps, objectives, and predetermined categories and subcategories within the GBGI framework. This approach ensures the inclusion of a diverse set of studies that are pertinent to the field of heat

mitigation. A range of relevant search terms based on keywords for urban heat mitigation was identified based on research gaps, objectives, and predetermined GBGI categories and subcategories to allow the identification of a wide range of studies relevant to heat mitigation. Search term combinations of GBGI type and heat are listed in Supplementary Information (SI) Table S1.

(2) Search and identification of relevant studies: A peer-reviewed literature search was conducted via Boolean search term combinations (Table S1) utilising Web of Science (WoS), as the most comprehensive database with the ability to handle complex keyword searches. Studies published between 2010 and 2023 were included. The chosen timeframe was specifically selected to effectively manage the substantial number of search results, preventing an overwhelming amount of hits. Moreover, this time frame enables a more comprehensive examination, particularly in relation to the GBGI, which gained increased recognition as "nature-based solution" after the year 2010. Cross-checks were performed using other databases such as Science Direct, Scopus, and Google Scholar to verify that no relevant studies were missed from the analysis. To ensure inclusivity, we used the same keywords as in WoS (Table S1) when searching on Google Scholar. We reviewed multiple pages of search results initially, but the relevance of studies decreased as we continued. Therefore, we concluded the search after examining the first 20 pages. Finally, we compared the results with papers from WoS and included any relevant publications that were missed in our analysis. After excluding articles not written in English, the search terms yielded a total of 27,486 publications, including review and research papers (Figure 2a).

(3) Selection of studies: The identified studies were screened against the following criteria: (a) addresses mitigation and/or adaptation to urban heat using one or several types of GBGI,

(b) distinctly identifies at least one GBGI sub-category under investigation, (c) the main GBGI category is clearly linked with heat mitigation performance, including actual or percentage temperature reduction, and any associated co-benefits, (d) the full texts were accessible from the databases for further review and data extractions. After removing duplicates, the remaining articles were reviewed and 25,974 publications that did not meet inclusion criteria were removed, leaving 1512 publications for further screening (Figure 2b). The full text of each paper was retrieved and assessed for eligibility (Figure 2c). Any articles not meeting the inclusion criteria, like those majorly focusing on causes and impacts of heat, methodological aspects of heat mitigation benefits without a direct tie to GBGI categories and temperature reductions or discussions confined to general green and blue spaces or green corridors without specific descriptions of GBGI types and cooling efficacy, were excluded. After this further screening, an additional 1250 papers were discarded, leaving 262 publications for final screening. Out of these, 60 more publications were excluded due to non-reporting of performances or failure to mention the utilised GBGI. Eventually, a set of 202 publications (1.8% of the originally identified 27,486 publications) was chosen for meta-analysis and subsequent deliberation in this review (Figure 2d, e).

(4) Cataloguing the data: Relevant data (e.g., location, type of GBGI, co-and dis-benefits, and knowledge gaps; Section 2.3) were extracted from the selected studies. The following data were extracted from the selected 202 studies: (1) the location of the GBGI study including the site, city, country, and region, (2) the specific type of GBGI, (3) the nature of the study, whether it was monitoring, modelling, remote sensing, or a combination of these, (4) either single or multiple GBGI, (5) qualitative or quantitative information on co-benefits, air temperature and land surface temperature (LST) reduction (in °C), and a day or night time air temperature reduction (in °C), (6) any identified knowledge gaps, and (7) key findings.

This process involved developing a data form to capture key information from the selected studies (Table S1). This extracted information was used to address the key objectives including when and where previous articles were published, the types of GBGI they utilised as mitigation measures for urban heat and the nature of co-benefits and maximum temperature reductions they reported.

(5) Collating, summarising, and reporting the results: The data obtained from the selected studies were analysed and synthesised in a way that addressed the specific questions raised as a part of the review objectives (Section 1). The extracted information on the (co-)benefits of various subcategories of GBGI was analysed using descriptive statistics using R-project software.[11] To develop an evaluation framework for GBGI types and the services offered to tackle heat-related challenges and their associated co-benefits, we adopted a min-max normalisation approach.[12] This approach, also referred to as feature scaling, included a linear transformation of the original data on a scale ranging from 1 (none) to 6 (very high) (see Table 2). Subsequently, this standardised methodology was applied to the pertinent publications within each GBGI category. Of the 202 publications, 64.7% discussed heat mitigation as their main ecosystem service. The remaining 35.3% discussed the co-benefits alongside the direct heat mitigation benefits (Section 3).

S2 Mechanisms of temperature and heat stress regulation by GBGI

S2.1 Mechanisms of temperature and heat stress regulation by green infrastructure

The mechanisms by which GI such as street trees, parks, green roofs, and green walls reduce heat are multifaceted and interconnected. Trees and plants help in the reduction of heat by providing shade and reducing the amount of direct sunlight reaching the ground, therefore lowering surface temperatures and mitigating the urban heat island (UHI) effect via creating a cooler microclimate.[13-15] Additionally, during evapotranspiration plants release

moisture which further cools the surrounding air by converting sensible heat into latent heat.[16] Parks can act as natural air conditioners through several mechanisms,[17-19] including the formation of microscale centripetal thermal system (park-breeze) that generate low-level advection currents which draw air from cooler green towards warmer urban areas.[20] Other GI elements such as green roofs, green walls, and roof gardens provide insulation, reduce heat absorption by buildings, and promote evaporative cooling (heat absorption, as water changes from liquid to a gas state in the air stream.[21-24] Vegetation also contributes to the dissipation of heat by acting as windbreaks, modifying airflow patterns, and facilitating natural ventilation.

S2.2 Mechanisms of temperature and heat stress regulation by blue infrastructure

Blue infrastructure (BI), in the form of water-based natural or constructed features including ponds and wetlands, actively mitigates heat effects by cooling the surrounding environment.[20] This is achieved through processes such as evapotranspiration, shading, the albedo effect, groundwater recharge, and temperature buffering.[25,26] BI can provide cooling during the day (acts as a heat sink by absorbing and storing heat from the surrounding environment) whereas it may lead to warming at night (re-releasing the heat due to water's higher heat capacity compared to the land surface).[27] Evaporation from water bodies also helps cool the air, creating a microclimate with lower temperatures and thereby helping to mitigate the UHI effect.[28] Larger urban water bodies can also generate cool breezes that further lower the ambient temperature and provide relief during hot weather through evaporative cooling.[20] Furthermore, surfaces of blue infrastructure are often highly reflective, especially under calm conditions, thereby increasing surface reflectivity which, in turn, contributes to the reduction of heat absorption,[29] thus helping to mitigate heat build-up and contributing to the cooling of the surrounding area. Some of the blue infrastructure such as

wetlands, ponds/lakes, swales, and rain gardens also act as natural sponges, storing water and releasing it during high air temperatures, thereby moderating temperatures in the vicinity by increasing water availability for evaporation through groundwater recharge.[30]

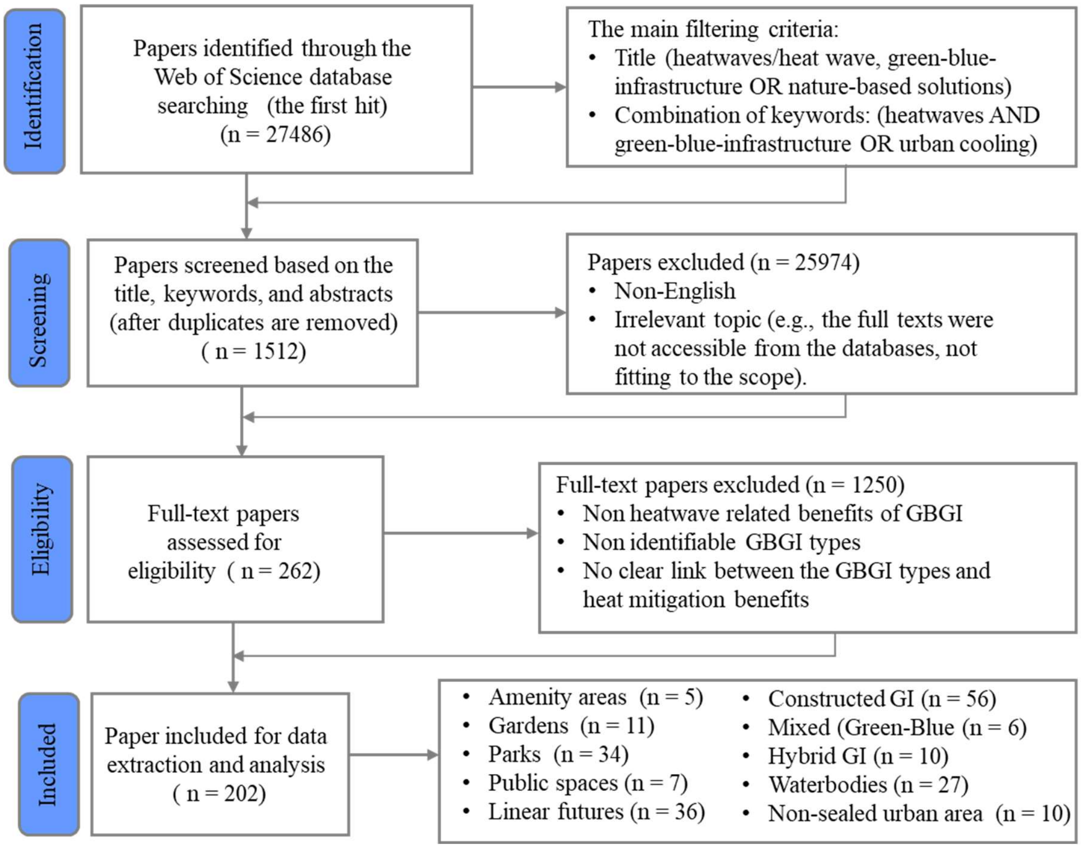

Figure S1. Flowchart of the inclusion and exclusion criteria (e.g. article identification, screening, eligibility check, and inclusion) process and the number of GBGI main categories included.

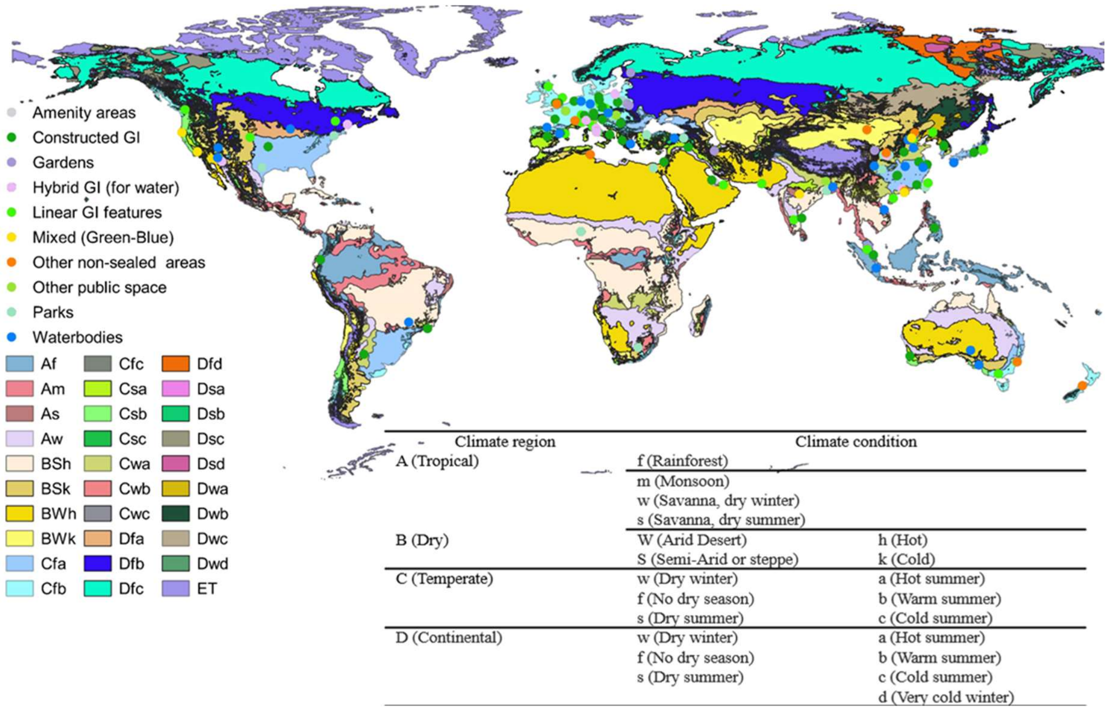

Figure S2. Köppen-Geiger climate classification: the main climate region (A-D) and detailed climate conditions (right column) where GBGs are implemented.

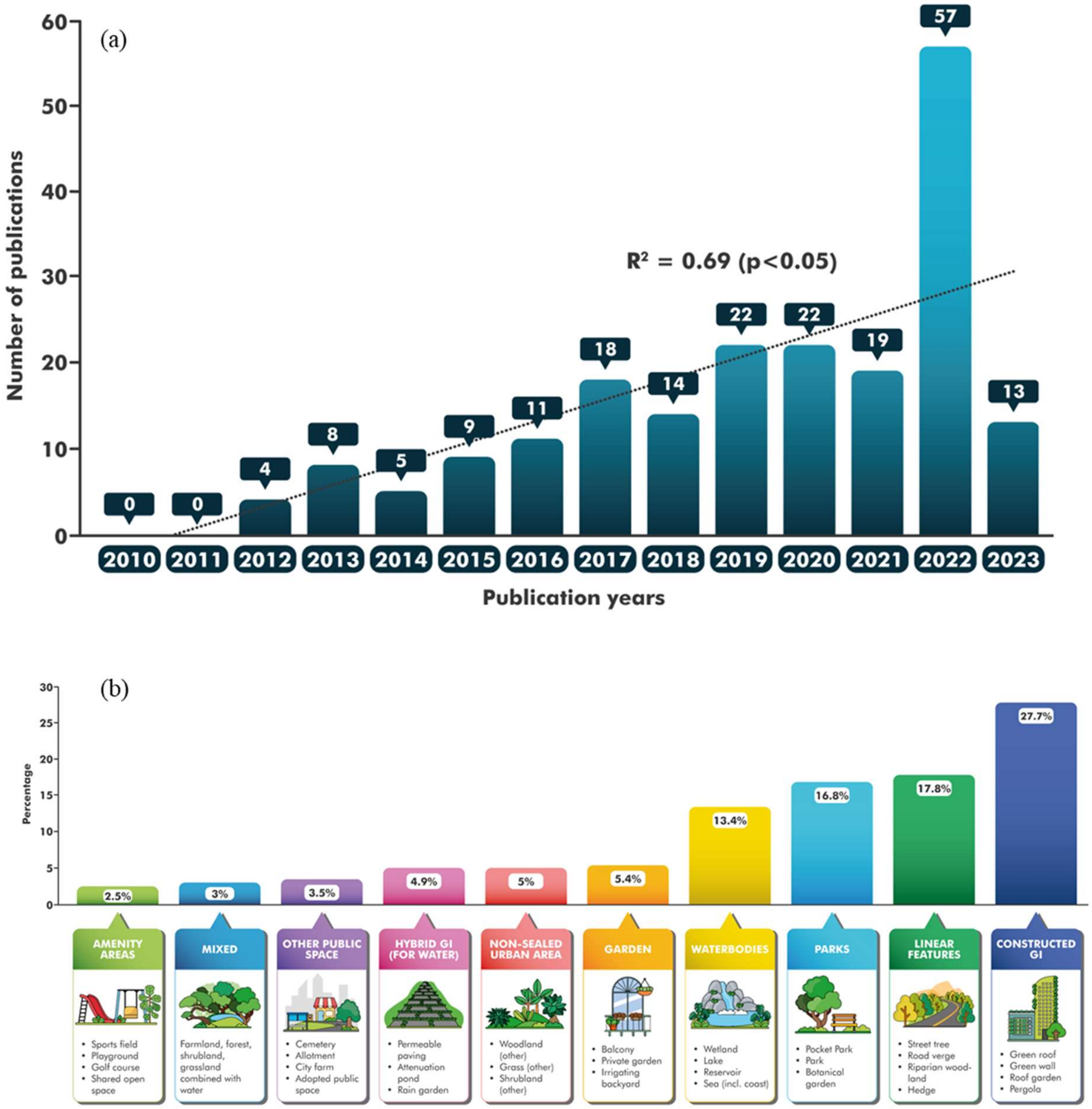

Figure S3. (a) The number of publications exhibiting a significant linear increase over time, with the linear trend (R^2=0.69; p=0.00043) providing the best fit among the various trends (exponential, linear, polynomial, power functions) analysed. Our search in 2023 was limited to the month of 30 March 2023, and the trend line did not incorporate the 2023 data as it did not cover the entire year. (b) The number of publications in each of the 10 main GBGI categories. The number of publications covering all the GBGI sub-categories is shown in Figure 1a.

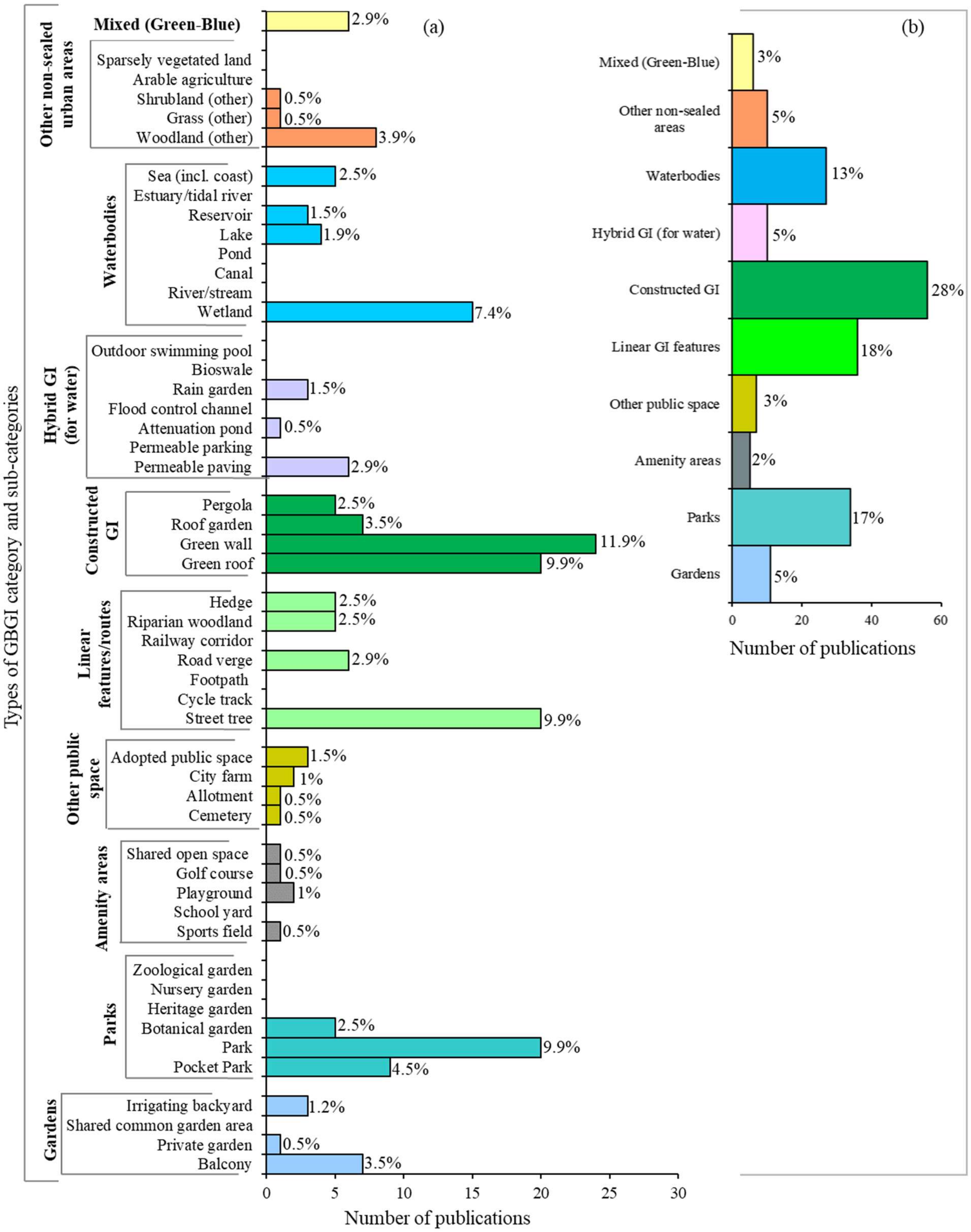

Figure S4. Relevant publications on the benefits of GBGI for heat adaptation and mitigation evidence gathered from the literature: (a) number of publications covering the main categories and sub-categories and (b) number of publications covering the main category (shown by the bold coloured text). The percentage values are printed on the top of each bar.

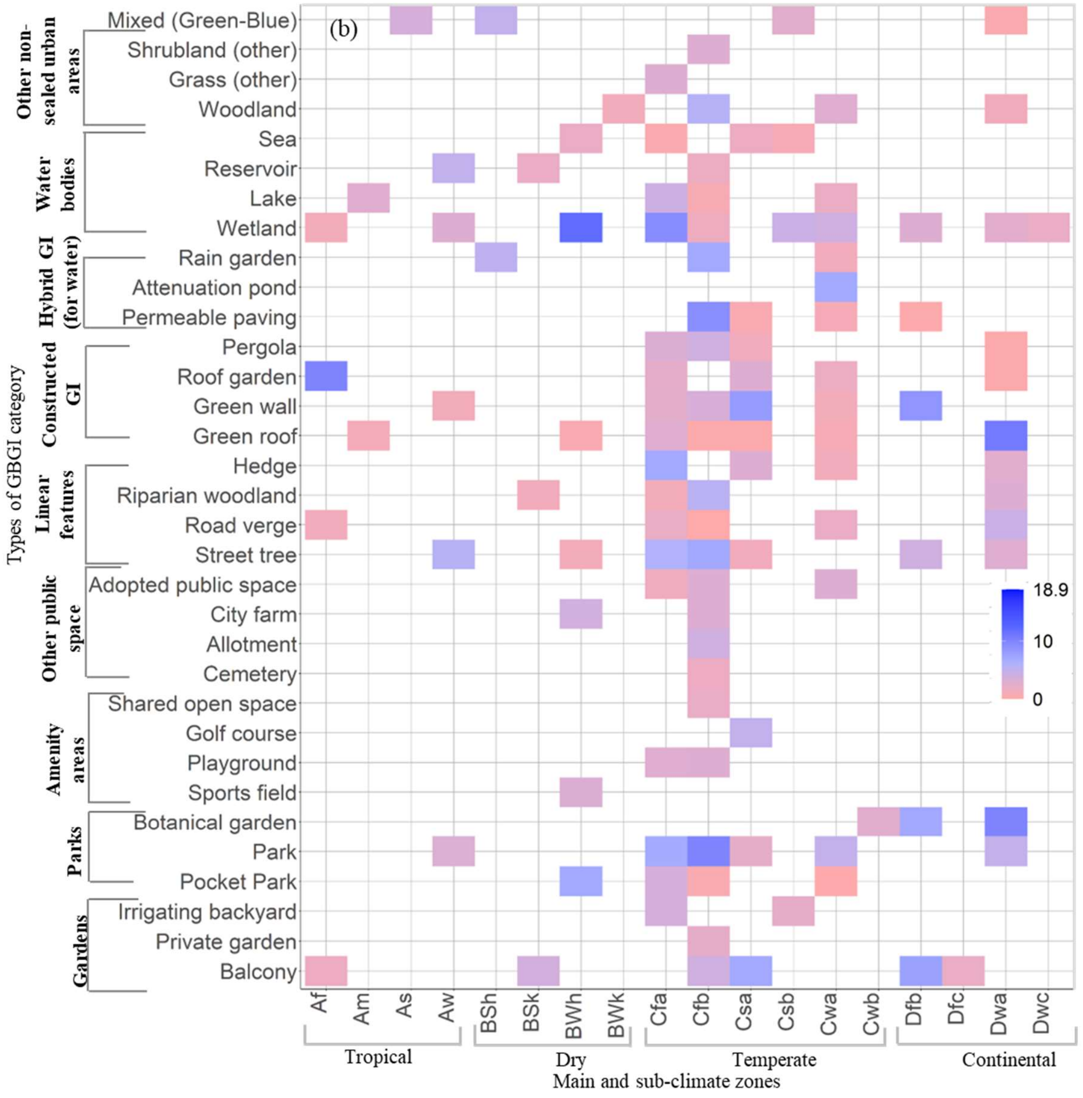

Figure S5. The effectiveness of the main and sub-GBGI categories implemented in tropical (n = 16), dry (n = 15), temperate (n = 137), and continental (n = 34) climate zones of Köppen-Geiger climate classification.

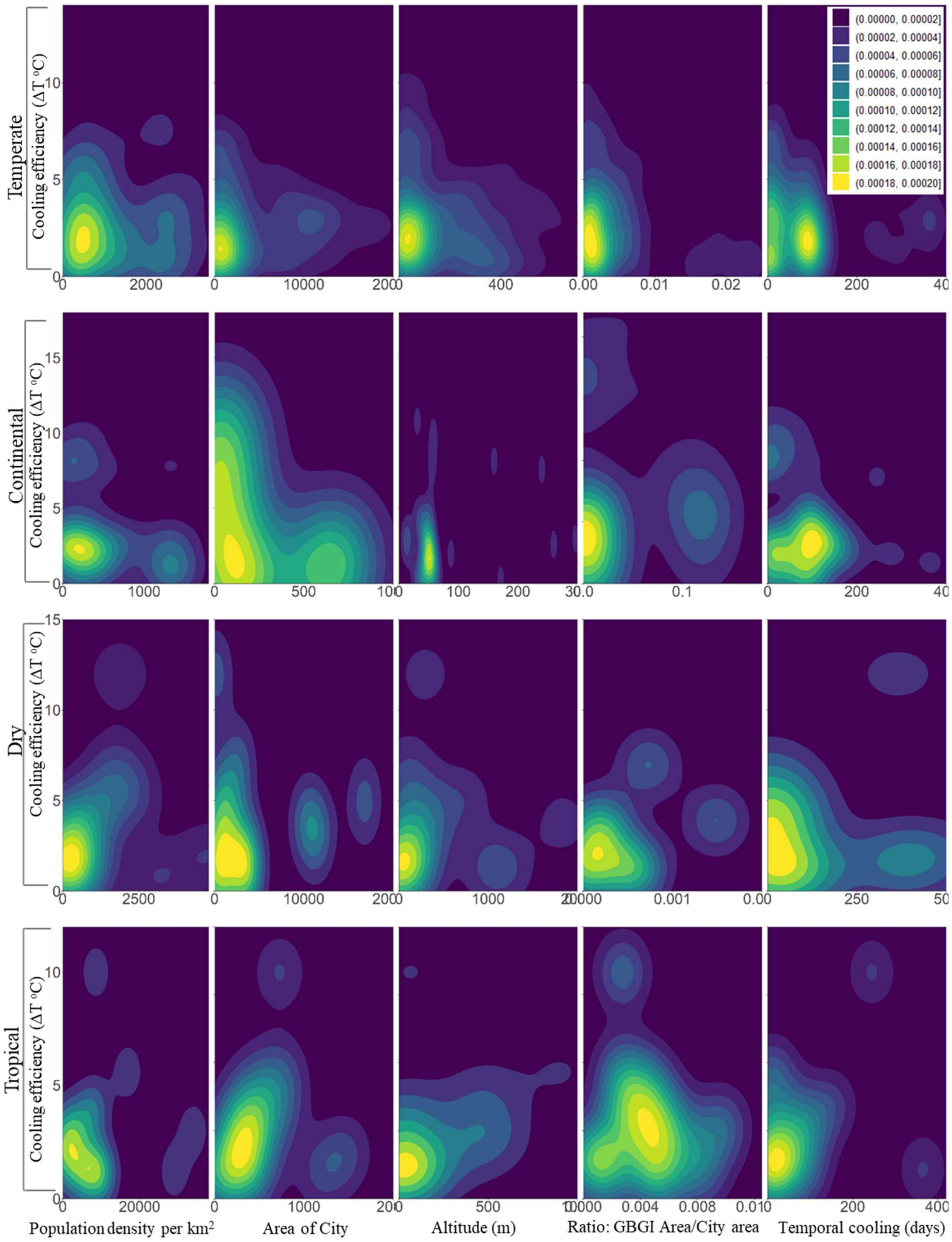

Figure S6. The density of GBGI cooling efficiency in different climate zones and against population density, area of the city, altitude, ratio: area of GBGI/area of the city, and temporal scale of cooling.

292

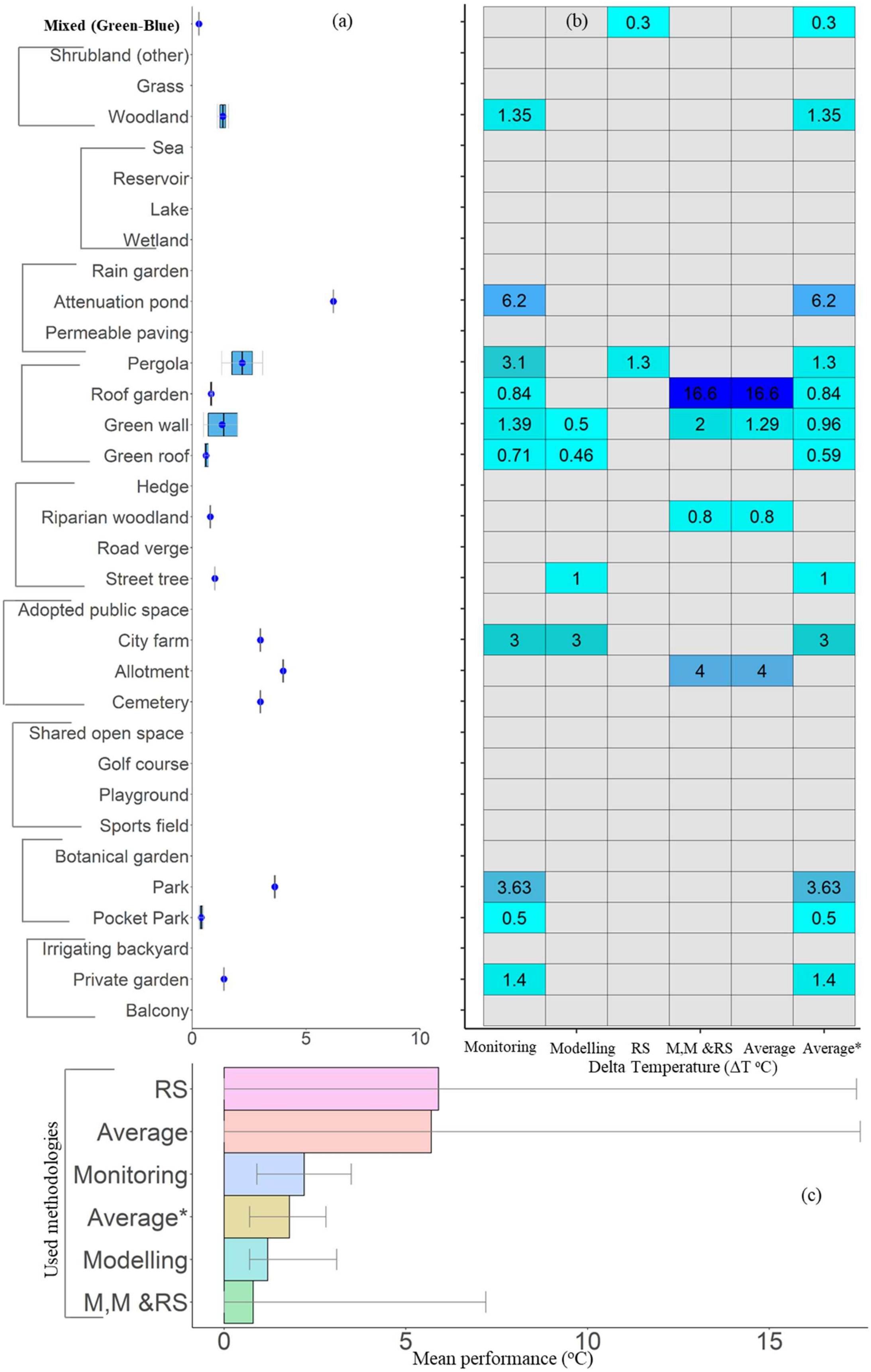
(a)
(b)
(c)
Mixed (Green-Blue)
Shrubland (other)
Grass
Woodland
Sea
Reservoir
Lake
Wetland
Rain garden
Attenuation pond
Permeable paving
Pergola
Roof garden
Green wall
Green roof
Hedge
Riparian woodland
Road verge
Street tree
Adopted public space
City farm
Allotment
Cemetery
Shared open space
Golf course
Playground
Sports field
Botanical garden
Park
Pocket Park
Irrigating backyard
Private garden
Balcony
0.3
0.3
1.35
1.35
6.2
6.2
3.1
1.3
1.3
0.84
16.6
16.6
0.84
1.39
0.5
2
1.29
0.96
0.71
0.46
0.59
0.8
0.8
1
1
3
3
3
4
4
3.63
3.63
0.5
0.5
1.4
1.4
Monitoring
Modelling
RS
M,M &RS
Average
Average*
Delta Temperature (ΔT ºC)
Used methodologies
RS
Average
Monitoring
Average*
Modelling
M,M &RS
Mean performance (ºC)

Figure S7. Night-time temperature reduction efficiency of GBGI sub-categories: (a) a summary of the overall performance of different GBGI types from all studies, (b) heatmap showing GBGI performances from for different methods and the average values, and (c) overall average of GBGI efficiency for urban heat mitigation. The 'Average' and 'Average*' values represent the average of all study types with and without RS data, respectively. M&M denotes combined monitoring and modelling studies. The colour gradient represents the performance, with grey cells representing studies that did not consider either monitoring, modelling, M&M, or RS. The figure uses a boxplot representation with the median indicated by a thick vertical black line, the mean represented by blue dots, and the upper and lower quartiles indicated by the box boundaries. The circle with a vertical line represents the GBGI categories with only one publication.

Table S1. The table below serves as a sample for organising the datasets obtained from the reviewed papers for each of the 51 sub-categories.

Paper Title	Source	Online Link	Study type (modelling, monitoring)	Location (City, Country)	Co-benefits	Multiple GBGIs	ΔT	Day-time ΔT °C	Night-time ΔT °C	Unit	Gap	Dis-benefits	Summary
Evaluating the vertical cooling performances of urban vegetation scenarios in a residential environment	Journal of Building Engineering	https://doi.org/10.1016/j.jobe.2021.102313	Modelling	Changsha, Hunan Province, China ()27°51′-28°40′ N, 111°53′-114°5′E	-	grass, shrubs, trees	0.49 °C	-	-	oC		-	The results of the study show that shadowing can directly affect the cooling effect of vegetation. The cooling effect of vegetation types that provide a large area of shadowing is better, and the green coverage rate cannot directly reflect the shadowing situation of the regional environment.
What's 'cool' in the world of green façades? How plant choice influences the cooling properties of green walls	Building and Environment	https://doi.org/10.1016/j.buildenv.2013.12.005	Monitor	Reading, UK			6.3 oC			oC			Artificial wall sections were used to provide replicated data sets in both outdoor and controlled environmental conditions.
Thermal behavior of a vertical green facade and its impact on the indoor and outdoor thermal environmen	Energy and Building	https://doi.org/10.1016/j.enbuild.2019.109502	Monitoring	Guangzhou, China,			2.7 °C			oC			Measurements and calculations of operative and WBGT temperatures, mass and heat fluxes and energy consumption in two westward identical rooms, one with a green wall system installed. The results indicated that transpiration could consume approximately 50% of solar radiation absorbed by the vegetation canopy.
Impacts of green walls on the characteristics of thermo-flow and photochemical reaction kinetics within street canyons	Urban Forestry and Urban Greening	https://doi.org/10.1016/j.ufug.2022.127568	Numerical modelling				1.02°C			oC			CFD looking on the effect of GWs on wind, temp, CO and NO2/NO/O3 fields in a street cayon. Four scenarios. Results are discussed in tamp reduction and co-benefits cells
A Hedera green façade – Energy performance and saving under different maritime-temperate, winter weather conditions	Building and Environment	https://doi.org/10.1016/j.buildenv.2015.04.011	Monitoring	University of Reading, UK			3 oC			oC			Temperature differences were affected by weather parameters, aspect, diurnal time and canopy density. Largest savings in energy due to vegetation were associated with more extreme weather, such as cold temperatures, strong wind or rain.

Table S2. The string of keywords used to record literature for the review of the efficiency of 51 GBGI categories to mitigate heat.

**GBGI Type**	**Keywords**	**Number of publications Identified**
		**Web of Science**
**Pocket park**	pocket park AND heat waves	-
	pocket park AND urban heat island	14
	pocket park AND temperature reduction	3
	pocket park AND cooling	10
	**Pocket Park (Total)**	27
**Park**	park NOT pocket park AND heat waves	1623
	park NOT pocket park AND urban heat island	907
	park NOT pocket park AND temperature reduction	6804
	park NOT pocket park AND cooling	9741
	**Park (Total)**	**19075**
**Botanical garden**	botanical garden OR arboretum AND heat waves	1
	botanical garden OR arboretum AND urban heat island	10
	botanical garden OR arboretum AND temperature reduction	10
	botanical garden OR arboretum AND cooling	20
	**Botanical garden (Total)**	**41**
**Heritage garden**	heritage garden AND heat waves	3
	heritage garden AND urban heat island	3
	heritage garden AND temperature reduction	1
	heritage garden AND cooling	6
	**Heritage garden (Total)**	13

**Nursery garden**	nursery garden AND heat waves	-
	nursery garden AND urban heat island	-
	nursery garden AND temperature reduction	12
	nursery garden AND cooling	3
	**Nursery garden (Total)**	15
**Zoological garden**	zoo OR zoos OR zoological garden AND heat waves	2
	zoo OR zoos OR zoological garden AND urban heat island	1
	zoo OR zoos OR zoological garden AND temperature reduction	12
	zoo OR zoos OR zoological garden AND cooling	31
	**Zoological garden (Total)**	46
**Street Tree**	street tree AND heat waves	51
	street tree AND urban heat island	326
	street tree AND temperature reduction	123
	street tree AND cooling	246
	**Street Tree (Total)**	746
**Cycle track**	cycle path OR cycle track AND heat waves	6
	cycle path OR cycle track AND urban heat island	14
	cycle path OR cycle track AND temperature reduction	421
	cycle path OR cycle track AND cooling	896
	**Cycle track (Total)**	1337
**Footpath**	footpath AND heat waves	-
	footpath AND urban heat island	6
	footpath AND temperature reduction	0
	footpath AND cooling	1
	**Footpath (Total)**	7

**Road verge**	roadside OR verge AND heat waves	6
	roadside OR verge AND urban heat island	26
	roadside OR verge AND temperature reduction	48
	roadside OR verge AND cooling	103
	**Road verge (Total)**	183
**Railway corridor**	rail AND heat waves	31
	rail AND urban heat island	7
	rail AND temperature reduction	300
	rail AND cooling	456
	**Railway corridor (Total)**	794
**Riparian woodland**	riparian tree OR riparian wood OR riparian forest AND heat waves	4
	riparian tree OR riparian wood OR riparian forest AND urban heat island	4
	riparian tree OR riparian wood OR riparian forest AND temperature reduction	16
	riparian tree OR riparian wood OR riparian forest AND cooling	28
	**Riparian woodland (Total)**	52
**Hedge**	hedge AND heat waves	3
	hedge AND urban heat island	9
	hedge AND temperature reduction	47
	hedge AND cooling	52
	**Hedges (Total)**	111
**Green Roof**	green roof AND heat waves	104
	green roof AND urban heat island	806
	green roof AND temperature reduction	360
	green roof AND cooling	886

	**Green Roof (Total)**	2156
**Green Wall**	green wall OR green facade AND heat waves	57
	green wall OR green façade AND urban heat island	295
	green wall OR green façade AND temperature reduction	298
	green wall OR green façade AND cooling	536
	**Green Wall (Total)**	1186
**Roof garden**	roof garden OR roof terrace AND heat waves	12
	roof garden OR roof terrace AND urban heat island	57
	roof garden OR roof terrace AND temperature reduction	22
	roof garden OR roof terrace AND cooling	80
	**Roof garden (Total)**	171
**Pergola**	pergola AND heat waves	-
	pergola AND urban heat island	5
	pergola AND temperature reduction	5
	pergola AND cooling	7
	**Pergola (Total)**	17
**Road verge**	Road verge AND heat waves	0
	Road verge AND urban heat island	0
	Road verge AND temperature reduction	1
	Road verge AND cooling	2
	(roadside* OR verge*) AND cooling	207
	(roadside* OR verge*) AND heat waves	12
	(roadside* OR verge*) AND urban heat island	29
	(roadside* OR verge*) AND temperature reduction	62
	**Road verge (Total)**	313

**Permeable paving**	Permeable Paving AND Heatwaves	0
	Permeable Paving AND Urban Heat Island	26
	Permeable Paving AND cooling	18
	Permeable Paving AND Temperature reduction	8
	Permeable parking/roadway AND Heatwaves	0
	Permeable parking/roadway AND Urban Heat Island	0
	Permeable parking/roadway AND Temperature reduction	0
	"permeable park*" OR "permeable road Heatwaves reduction"*	13
	"permeable park*" OR "permeable road Urban Heat Island"*	13
	Permeable park*" OR "permeable road Temperature reduction	13
	Permeable Paving AND cooling	18
	**Permeable Paving (Total)**	109
**Attenuation pond**	Attenuation pond AND Heatwaves	0
	Attenuation pond AND Urban Heat Island	1
	Attenuation pond AND Temperature reduction	8
	Attenuation pond AND cooling	6
	**Permeable Paving (Total)**	15
**Permeable paving**	Flood control channel AND Heatwaves	0
	Flood control channel AND Urban Heat Island	1
	Flood control channel AND Temperature reduction	22
	flood* OR channel or Heatwaves*	1
	flood* OR channel or Urban Heat Island*	2
	flood* OR channel or Temperature reduction*	35
	Flood control channel AND cooling	30
	**Flood Control Channel (Total)**	91

**Rain garden**	Rain garden AND Heatwaves	2
	Rain garden AND Urban Heat Island	23
	Rain garden AND Temperature reduction	22
	Rain garden AND cooling	14
	**Rain Garden (Total)**	61
**Bioswale**	Bioswale AND cooling	1
	Bioswale AND Heatwaves	0
	Bioswale AND Urban Heat Island	1
	Bioswale AND Temperature reduction	1
	**Bioswale (Total)**	3
**Outdoor swimming pool**	Outdoor swimming pool AND cooling	16
	Outdoor swimming pool AND Heatwaves	0
	Outdoor swimming pool AND Urban Heat Island	3
	Outdoor swimming pool AND Temperature reduction	3
	**Outdoor swimming pool (Total)**	22
**Canal**	Canal AND cooling	338
	Canal AND Heatwaves	1
	Canal AND Urban Heat Island	4
	Canal AND Temperature reduction	160
	**Canal (Total)**	503
**Estuary/ tidal river**	Estuary/tidal river AND cooling	0
	Estuary/tidal river AND Heatwaves	0
	Estuary/tidal river AND Urban Heat Island	0
	Estuary/tidal river AND Temperature reduction	0
	**Estuary/ tidal river (Total)**	0
**River/ Stream**	River/stream AND cooling	8
	River/stream AND Heatwaves	0

	River/stream AND Urban Heat Island	0
	River/stream AND Temperature reduction	7
	river* OR stream* AND Heatwaves	0
	river* OR stream* AND Urban Heat Island	0
	river* OR stream*AND Temperature reduction	0
	**River/ Stream (Total)**	15
**River/ Stream**	Reservoir AND cooling	3955
	Reservoir AND Heatwaves	15
	Reservoir AND Urban Heat Island	44
	Reservoir AND Temperature reduction	0
	**Reservoir (Total)**	4014
**Lake**	Lake AND Heatwaves	53
	Lake AND Urban Heat Island	167
	Lake AND Temperature reduction	0
	Lake AND cooling	4715
	**Lake (Total)**	4935
**Sea**	Sea (incl. coast) AND Heatwaves	0
	Sea (incl. coast) AND Urban Heat Island	495
	Sea (incl. coast) AND Temperature reduction	6957
	Sea AND cooling	15381
	(sea OR seaside OR coast* OR beach* OR shore and Heatwaves*)	0
	**Sea (Total)**	22833
**Pond**	Pond AND Heatwaves	0
	Pond AND Urban Heat Island	0
	Pond AND cooling	945
	Pond AND Temperature reduction	0

	**Pond (Total)**	945
**Balcony/terrace**	Balcony AND Heatwaves	1
	Balcony AND Urban Heat Island	7
	Balcony AND Temperature reduction	21
	terrace AND Urban Heat Island	16
	terrace AND Urban Heatwaves	1
	terrace AND Temperature reduction	233
	**Balcony/terrace (Total)**	279
**Road verge**	Riparian woodland AND heat waves	1
	Riparian woodland AND urban heat island	0
	Riparian woodland AND temperature reduction	7
	Riparian woodland AND cooling	20
	("riparian tree*" OR "riparian wood*" OR "riparian forest*") AND cooling	66
	("riparian tree*" OR "riparian wood*" OR "riparian forest*") AND heat waves	3
	("riparian tree*" OR "riparian wood*" OR "riparian forest*") AND urban heat island	4
	("riparian tree*" OR "riparian wood*" OR "riparian forest*") AND temperature reduction	30
	**Riparian woodland (Total)**	131
**Playground**	Playground AND Heatwaves	1
	Playground AND Urban Heat Island	9
	Playground AND Temperature reduction	19
	Playground AND cooling	33
	**Playground (Total)**	62
**Golf course**	Golf course AND Heatwaves	0
	Golf course AND Urban Heat Island	4

	Golf course AND Temperature reduction	11
	Golf course AND cooling	111
	**Golf course(Total)**	126
**Shared open space**	Shared open space AND Heatwaves	1
	Shared open space AND Urban Heat Island	4
	Shared open space AND Temperature reduction	10
	Shared open space AND cooling	44
	**Shared open space(Total)**	59
**Cemetery**	Cemetery AND Heatwaves	0
	Cemetery AND Urban Heat Island	5
	Cemetery AND Temperature reduction	1
	Cemetery AND cooling	12
	**Cemetery (Total)**	18
**Allotment**	Allotment AND Temperature reduction	5
	Allotment AND Urban Heat Island	6
	Allotment AND Temperature reduction	5
	Allotment AND cooling	15
	**Allotment (Total)**	31
**City farm**	City farm AND Heatwaves	1
	City farm AND Urban Heat Island	23
	City farm AND Temperature reduction	20
	City farm AND cooling	44
	**City farm (Total)**	88
**Adopted public space**	Adopted public space AND Heatwaves	1
	Adopted public space AND Urban Heat Island	13
	Adopted public space AND Temperature reduction	8

	Adopted public space AND cooling	18
	**Adopted public space (Total)**	40
**Woodland**	Woodland (other) AND Heatwaves	3
	Woodland (other) AND Urban Heat Island	8
	Woodland (other) AND Temperature reduction	34
	Woodland AND cooling	457
	**Woodland (Total)**	502
**Grass (other)**	Grass (other) AND Heatwaves	9
	Grass (other) AND Urban Heat Island	40
	Grass (other) AND Temperature reduction	155
	Grass (other) AND cooling	3041
	**Grass (other) (Total)**	3245
**Arable agriculture**	Arable agriculture AND Heatwaves	0
	Arable agriculture AND Urban Heat Island	0
	Arable agriculture AND Temperature reduction	0
	Arable agriculture AND cooling	24
	**Arable agriculture (Total)**	24
**Private Garden**	Private garden AND Heatwaves	0
	Private garden AND Urban Heat Island	5
	Private garden AND Temperature reduction	1
	Private garden AND cooling	13
	**Private Garden (Total)**	19
**Shared common garden**	Shared common garden area AND Heatwaves	0
	Shared common garden area AND Urban Heat Island	1
	Shared common garden area AND Temperature reduction	0

	Shared common garden area AND cooling	0
	**Shared Common garden (Total)**	1
**Wetland**	Wetland AND Heatwaves	15
	Wetland area AND Urban Heat Island	55
	Wetland AND Temperature reduction	589
	Wetland AND cooling	587
	**Wetland (Total)**	1246
**Estuary**	Estuary AND Heatwaves	22
	Estuary AND Urban Heat Island	13
	Estuary AND Temperature reduction	474
	Estuary AND cooling	428
	**Estuary (Total)**	937
**Sports fields**	Sports field AND Heatwaves	1
	Sports field AND Urban Heat Island	2
	Sports field AND Temperature reduction	25
	Sports field AND cooling	102
	**Sports field (Total)**	130
**School yard**	School yard AND Heatwaves	0
	School yard AND Urban Heat Island	2
	School yard AND Temperature reduction	0
	School yard AND cooling	3
	**School yard (Total)**	5
**Shrubland**	Shrubland (other) AND Heatwaves	0
	Shrubland (other) AND Urban Heat Island	0
	Shrubland (other) AND Temperature reduction	0
	Shrubland (other) AND cooling	16

	**Shrubland (Total)**	16
**Sparsely vegetated land**	Sparsely vegetated land AND Heatwaves	0
	Sparsely vegetated land AND Urban Heat Island	0
	Sparsely vegetated land AND Temperature reduction	0
	Sparsely vegetated land AND cooling	8
	**Sparsely vegetated land (Total)**	8

Table S3. Classification matrix to categorise the number of studies available for each of the GBGI sub-categories in terms of heat mitigation. Zero values indicate that there is negligible empirical evidence available for GBGI's against urban heat mitigation, including heatwaves.

Scale	Conditional performance (%)[a]	Evidence-based classification (%)[b]	Number of GBGI types[c]
1	None	0	18
2	Very low	≥0 ≤20	15
3	Low	≥20 ≤40	9
4	Medium	≥40 ≤ 60	4
5	High	≥60 ≤80	1
6	Very High	≥80	4

[a]The number of publications from negligible to very high under the six-classification scale. [b]The percentage availability of publications for each classification scale. [c]The number of GBGI types found in each of the classification scales.

Table S4. The best performing GBGI types in each climate zone and sub-climate type with reported magnitude and measured scale (i.e. inside/outside of GBGI) of cooling providing details of surroundings.

	GBGI (Sub climate#1, and del T)	GBGI (sub climate#2, and del T)	Inside/outside (scale)	Nearby surrounding
Temperate	Wetland (Cfa, 10)	Park (Cfb, 9.2)	Wetland works well at meso scale (mostly	Near built-up area (park),

			inside) than micro, on the contrary park works better on micro-scale (inside and outside both).	Near nature (wetland)
Continental	Greenwall, Botanical garden (Dfb, 8.7 & 10)	Green roof and Botanical garden (Dwa, 10.8 & 10)	Works well at Microscale but not at meso and macro but the park can be suggested at the mesoscale.<br><br>Green roof (inside, outside and top) botanical garden (inside-outside), green wall (near)	Built-up area (green wall and botanical garden)<br><br>Green roof (mixed environment of grey and nature)
Dry	Wetland, Pocket park (BWh, 12 & 7))	-	Microscale wetland (inside) pocket park (inside-outside)	Wetland (near nature)<br><br>Pocket park (built-up area)
Tropical	Roof garden (Af, 10)	-	Microscale<br><br>(inside and top)	Built up area

Table S5. Sample size (n), correlation of population and p-value density, city area, altitude, ratio of GBGI and city area, and temporal cooling with reported cooling by GBGI in four different climatic conditions i.e. continental, dry, temperate and tropical.

Climate Zone		Correlation	P-value
Population density (n = 34)	Continental	0.0183	0.919
(n = 15)	Dry	0.193	0.4791
(n = 137)	Temperate	-0.013	0.8827
(n = 16)	Tropical	0.2241	0.404
City Area	Continental	0.018	0.9189

(n = 15)	Dry	0.1981	0.4791
(n = 137)	Temperate	-0.013	0.8827
(n = 16)	Tropical	0.22	0.404
Altitude	Continental	0.135	0.4449
(n = 15)	Dry	0.0158	0.9552
(n = 137)	Temperate	-0.089	0.2975
(n = 16)	Tropical	0.325	0.2188
Ratio GBGI/city	Continental	0.0917	0.606
(n = 15)	Dry	-0.117	0.6768
(n = 137)	Temperate	-0.017	0.8386
(n = 16)	Tropical	-0.328	0.2142
Temporal cooling	Continental	0.208	0.2367
(n = 15)	Dry	0.1132	0.6879
(n = 137)	Temperate	0.0399	0.6427
(n = 16)	Tropical	0.2824	0.2892

Table S6. The location, study types (in-situ, modelling, combined (in-situ and modelling), and remote sensing), and performance in reducing temperature (**ΔT °C**)) of different types of GBGI categories against extreme heat extracted from 202 papers.

**GBGI Type**		**GBGI Categories**	**Location (city, country)**	**Study type**	**Performance ΔT (°C)**	**Reference (Year)**
Gardens		Balcony	Vienna	Monitoring	4	Teichmann et al.[31]
			Malaysia	Monitoring	1.7	Toe and Kubota[32]
			Tehran, Iran	Modelling	7	Aghasizadeh et al.[33]
			China	Modelling	3.8	Cui and Zheng[34]
			Lublin,	Monitoring	0.22	Grudzińska[35]

			Poland			
			Tampere, Finland	Monitoring	2	Hilliaho et al.[36]
			Zamo, Poland	Modelling	7.6	Grudzińska[37]
		Private garden	Melbourne, Australia	Monitoring	2.3	Cheung et al.[38]
		Irrigating backyard	Sydney, Australia	Modelling	3	Gao et al.[39]
			Adelaide, Australia	Modelling	2.3	Broadbent et al.[40]
			United States	Remote Sensing	3.74	Wang et al.[41]
Parks		Pocket Park	Hong Kong	Monitoring	0.38	Lau et al.[42]
			Hong Kong	Monitoring	1.09	Lin et al.[433]
			New York	Monitoring	0.5	Rosso et al.[44]
			Veszprém, Hungary	Modelling	0.6	Trájer et al.[45]
			Xi'an, China	Modelling	1.1	Hou et al.[46]
			Xi'an, China	Monitoring & Modelling	0.43	Ma et al.[47]
			Shanghai, China	Monitoring	3.6	Wu et al.[48]
			Cairo Metropolitan Area, Egypt	Modelling	7	Ibrahim[49]
			Hong Kong	Monitoring & Modelling	0.13	Huang et al.[50]
		Park	Chongqing, Southwest China	Modelling	0.8	Lu et al.[51]

			Abuja, Nigeria	Remote Sensing	2.04	Chibuike et al.[52]
			Shenzhen City, China	Monitoring	5.15	Zhang et al.[53]
			Yreb, China	Remote Sensing	2.34	Shi et al.[54]
			Xian, China	Monitoring	0.78	Du et al.[55]
			Shenzhen, China	Remote Sensing	3.02	Peng et al.[56]
			Taipei, Taiwan	Monitoring & Modelling	2.42	Yang et al.[57]
			Wuhan, China	Monitoring	3.5	Chen et al.[58]
			Kolkata Metropolitan Area, India	Remote Sensing	3.15	Das et al.[59]
			Beijing, China	Monitoring	1.38	Zhou et al.[60]
			Melbourne, Australia	Remote Sensing	3.28	Algretawee[61]
			Beijing, China	Remote Sensing	1.71	Qiu and Jia[62]
			Beijing, China	Monitoring	1.09	Li et al.[63]
			Austin, US	Remote Sensing	6.89	Gao et al.[64]
			Taiwan	Monitoring & Modelling	2.42	Yang et al.[65]
			Özgürlük Park, Istanbul, Turkey	Monitoring & Modelling	2.3	Şimşek et al.[66]
			Beijing, China	Monitoring	2.71	Li et al.[67]

			Hong Kong	Monitoring	4.9	Cheung er al.[68]
			Melbourne, Australia	Remote Sensing	10	Algretawee[69]
			Beijing, China	Monitoring	4.8	Yan et al.[70]
		Botanical garden	Baoji, China	Monitoring	2.7	Chang and Li[71]
			Erzurum, Turkey	Monitoring	2.6	Irmak et al.[72]
			Beijing, China	Monitoring	10	Su [73]
			Erzurum, Turkey	Monitoring & Modelling	2.2	Yilmaz et al.[74]
			Erzurum, Turkey	Monitoring	7.1	Yilmaz et al.[75]
Amenity areas		Sports field	Khalifa stadium in Doha, Qatar	Monitoring & Modelling	3.1	Ghani et al.[76]
		Playgroun d	Warsaw, Poland	Monitoring	5	Kuchcik et al.[77]
			United States	Remote Sensing	5.5	Vanos et al.[78]
		Golf course	Perth, Australia	Remote Sensing	6	Nguyen et al.[79]
		Shared open space	Maxvorstadt, Munich	Modelling	2.1	Zölch et al.[80]
Other public spaces		Cemetery	Budapest's, Hungary	Monitoring	1.7	Sallay et al.[81]
		Allotment	Berlin, Germany	Remote Sensing	4	Rost et al.[82]
		City farm	Phoenix, Arizona	Monitoring	3.9	Hawkins et al.[83]
			Paris, France	Modelling	3	Masson et al.[84]

		Adopted public space	Raiganj, West Bengal, India	Monitoring & Modelling	3	Basu and Das[85]
			Bologna, Italy	Modelling	3	Boeri et al.[86]
			Liverpool, NSW, Australia	Modelling	1.5	Abdollahzadeh and Biloria[87]
Linear features/routes		Street tree	Hangzhou city, Zhejiang Province, China	Monitoring	1.8	Cai et al.[88]
			Turin, Italy	Monitoring	0.5	Morabito et al.[89]
			Florence, Italy	Modelling	9.4	Napoli et al.[90]
			Nanjing , Jiangsu Province, China	Modelling	5.5	Xi et al.[91]
			Karachi, Pakistan	Modelling	1.2	Zeeshan et al.[92]
			Abu Dhabi	Monitoring & Modelling	0.9	Abu Ali et al.[93]
			Rome, Italy	Remote Sensing	3.2	Marando et al.[94]
			Karachi, Pakistan	Modelling	1.2	Zeeshan et al.[95]
			Montreal, Canada	Modelling	4	Wang et al.[96]
			Vancouver, Canada	Remote Sensing	12	Lachapelle et al.[97]
			Barcelona, Spain	Modelling	1.3	Segura et al.[98]
			Shenyang,	Monitoring	2.9	Miao et al.[699]

			China			
			Prague Czech Republic	Modelling	5	Geletic et al.[100]
			Basel, Switzerland	Monitoring & Modelling	2	Mussetti et al.[101]
			Bangalore, India	Monitoring	5.6	Valishery et al.[102]
			Dresden, Germany	Monitoring	2.22	Gillner et al.[103]
			Melbourne, Australia	Monitoring	1.5	Coutts et al.[104]
			Richmond, Australia	Monitoring	2.1	Sanusi et al.[105]
			Vancouver, Canada	Modelling	7.1	Aminipour et al.[106]
			Tsukuba City, Japan	Monitoring	5.9	Kusaka et al.[107]
		Road verge	Jongro, Seoul, Republic of Korea	Monitoring	4.44	Cho[108]
			Taipei, Taiwan	Monitoring	0.68	Huang and Li[109]
			New Belgrade, Serbia	Monitoring	2.1	Stojanovic et al.[110]
			Kuala Lumpur, Malaysia	Monitoring	1.3	Zaki et al.[111]
			Haikou, China	Modelling	2	Zheng et al.[8112]
			Czech Republic	Monitoring & Modelling	0.05	Žižlavská et al.[113]

		Riparian woodland	Sydney, Australia	Monitoring	1.16	Adams and Smith[1144]
			Ejina basin, China	Monitoring	1.28	Yonghong et al.[115]
			Yorkshire, England	Monitoring	3	Tsai et al.[116]
			Glen Girnock, UK	Remote sensing	5.4	Dugdale et al.[117]
			Beijing, China	Monitoring	3	Zheng et al.[118]
		Hedge	Beijing, China	Modelling	2.68	Zhang and Hu[119]
			Lazio, Italy	Modelling	3	Peluso et al.[120]
			Rome, Italy	Modelling	3	Del Serrone et al.[121]
			Shenzhen, China	Monitoring & Modelling	1.29	Zou et al.[1222]
			Sakai, Japan	Remote Sensing	7	Yoshida et al.[123]
		Green roof	Berlin, Germany	Modelling	0.44	Wang et al.[124]
			Mandaue, Philippines	Modelling	1.1	Cortes et al.[125]
			Sydney, Australia	Monitoring	9.63	Fleck et al.[126]
			Xiamen, China	Remote Sensing	0.91	Dong et al.[127]
			Belgrade, Serbia	Monitoring	5.5	Kostadinovic et al.[128]
			Nanjing, China	Monitoring	1.1	Peng et al.[129]
			Tseung Kwan O New Town,	Monitoring	4.9	Lee and Jim[130]

			Hong Kong, China			
			Neubrandenburg, Germany	Monitoring	1.5	Kohler and Kaiser[131]
			Gangnam-gu, Seoul, South Korea	Monitoring	10.8	Park et al.[132]
			Jerusalem and Tel Aviv	Monitoring & Modelling	0.4	Lynn and Lynn[133]
			Shenzhen, China	Monitoring	4.03	Chen et al.[134]
			Mandaue, Philippines	Modelling	1.1	Cortes et al.[135]
			Utrecht, The Netherlands	Monitoring	0.2	Solcerova[136]
			Lodz, Poland	Modelling	0.19	Bochenek and Klemm[137]
			Guangzhou, China	Modelling	0.1	Chen et al.[138]
			Hamad, Northern Bahrain	Modelling	0.72	Elnabawi and Saber[139]
			Chengdu, China	Monitoring	0.94	Zuo et al.[140]
			Rome, Italy	Modelling	0.16	Iaria and Susca[141]
			Sydney, Australia	Monitoring	2.92	Fleck et al.[142]
			Cordoba, Argentina	Monitoring	0.892	Robbiati et al.[143]
Constructed GI on infrastructure		Green wall	Changsha, Hunan Province, China	Modelling	0.49	Liao et al.[144]

			Reading, UK	Remote Sensing	6.3	Cameron et al.[145]
			Guangzhou, China	Monitoring	3.6	Zhang et al.[146]
			Shanghai, China	Modelling	1.02	Liu et al.[147]
			Reading, UK	Monitoring	3	Cameron et al.[148]
			Madrid, Spain	Monitoring	2.7	Jesus et al.[149]
			Hong-Kong	Monitoring	1.19	Lee and Jim[150]
			Rio de Janeiro, Brazil	Modelling	1.16	Feitosa and Wilkinson[151]
			Zürich, Switzerland	Modelling	0.1	Li et al.[152]
			Prague, Czech Republic	Monitoring & modelling	2	Geletič et al.[153]
			Ljubljana, Slovenia	Remote Sensing	18.9	Šuklje et al.[154]
			Tyrol, Austria	Monitoring	8.7	Medl et al.[155]
			Sydney, Australia	Monitoring	7.7	Feitosa and Wilkinson[156]
			Bari,Valenzano, Italy	Monitoring	7	Blanco et al.[157]
			Pertth, Western Australia	Monitoring	8.1	Bakhshoodeh et al.[158]
			Quito, Ecuador	Modelling	1.43	Davis et al.[159]
			London Olympic Park	Monitoring	1.5	Hosseinzadeh et al.[160]

			La Rochelle, France	Modelling	1.9	Djedjig et al.[161]
			Chennai, India	Modelling	1.2	Pragati et al.[162]
			Guangzhou, China	Monitoring	8	Lin et al.[163]
			United States	Monitoring	4.3	Price et al.[164]
			Hong Kong	Monitoring	1.2	Lee and Jim [165]
			Munich, Germany	Modelling	3.5	Lin et al.[166]
			Chenzhou, Hunan, China	Modelling	2.56	Li et al.[167]
		Roof garden	Nanjing, China	Monitoring	1	Peng et al.[168]
			Xinxiang, Henan, China	Monitoring	1	Shen [169]
			Duhok, Iraq	Monitoring	3	AbdulBaqi [170]
			Seoul, South Korea	Modelling	0.3	Kim et al.[171]
			Hong Kong	Monitoring	1.8	Lee and Jim [172]
			Singapore	Monitoring	17.7	Tan et al.[173]
			Singapore	Remote Sensing	10	Tan et al.[174]
		Pergola	Nagoya, Japan	Remote Sensing	16.2	Watanabe et al.[175]
			Arta, Greece	Monitoring & Modelling	1.3	Katsoulas et al.[176]
			Lleida, Spain	Monitoring	3.1	Chafer et al.[177]

			Suwon, Republic of Korea	Monitoring	0.2	Kong et al.[178]
			Vienna, Austria	Monitoring & Modelling	4	Teichmann et al.[179]
Hybrid GI		Permeable paving	Vertemate con Minoprio, CO, Italy	Monitoring	2.8	Fini et al.[180]
			Perugia, Italy	Monitoring	9.2	Kousis et al.[181]
			Zhouzhi County, Xi'An, Shaanxi, China	Monitoring	6	Lu et al.[182]
			Rome, Italy	Modelling	0.6	Moretti et al.[183]
			Guangzhou, China	Monitoring	1	Wang et al.[184]
			Changping China	Monitoring	0.19	Wang et al.[185]
		Attenuation pond	Guangzhou, China	Monitoring	7	Yang et al.[186]
		Rain garden	Yau Tsim Mong district, Hong Kong	Modelling	1.3	An et al.[187]
			Tucson, Arizona	Monitoring	5.2	Buzzard et al.[188]
			Gdansk, Poland	Monitoring	7	Kasprzyk et al.[189]
Waterbodies		Wetland	Zoige Plateau, China	Monitoring	2	Bai et al.[190]
			Beijing, China	Remote Sensing	7.83	Cai et al.[191]

			Vienna, Austria	Monitoring	3.4	Pucher et al.[192]
			Avondale, Arizona	Remote Sensing	12	Ruiz-Aviles et al.[193]
			Dhaka, Bangladesh, Anatolia	Modelling	3	Shahjahan et al.[194]
			Central Anatolia, Turkey	Remote Sensing	4.38	Şimşek and Ödül[195]
			Beijing, China	Monitoring	3.15	Sun et al.[196]
			Eastern Germany	Modelling	1.6	Sušnik et al.[197]
			Palembang City, Indonesia	Monitoring	1.2	Triyuly et al.[198]
			Chengdu, China	Monitoring	4.08	Wu et al.[199]
			Wuhan, China	Monitoring	4.8	Xu et al.[200]
			Hangzhou, China	Remote Sensing	9.27	Zhang et al.[201]
			Northeast China	Remote Sensing	8.15	Wenguang et al.[202]
			Prairie Pothole Region, North America	Remote Sensing	3	Zhang et al.[203]
			Beijing, China	Remote Sensing	2.6	Sun et al.[204]
		Lake	Hue Citadel, Hue City, Vietnam	Remote Sensing	2.82	Le Phuc et al.[205]
			Altenberge, Germany	Modelling	0.8	Theeuwes et al.[206]

			Wuhan, China	Monitoring	4.2	Xu et al.[207]
			Daming lake, Jinan, China	Monitoring	1.9	Yang et al.[208]
		Reservoir	São José do Rio Preto, Brazil	Monitoring	5	Masiero and de Souza[209]
			Northern, Spain	Monitoring	2	Novo et al.[210]
			Santander, Spain	Monitoring	1.82	Novo et al.[2111]
		Sea	Athens, Greece	Monitoring & Modelling	1.7	Dandou et al.[212]
			Sendai, Japan	Monitoring	1.3	Zhou et al.[213]
			Adelaide, Australia	Monitoring	0.9	Zhou et al.[214]
			South Australia	Monitoring	2	Zhou et al.[215]
			Wuhan, China	Modelling	0.4	Zhu et al.[216]
Other non-sealed urban area		Woodland	Guildford, UK	Monitoring	5.7	Sahani et al.[217]
			Hong Kong Golf Course	Monitoring	1.43	Fung and Jim[218]
			Hong Kong Golf Course	Monitoring	4.2	Fung and Jim[219]
			Ejina basin	Monitoring & Modelling	1.28	Yonghong et al.[220]
			Baoji Botanical Garden	Monitoring	2.7	Chang and Li[221]
			Beijing, China	Monitoring	1.32	Liu et al.[222]

			Xi’an, China	Remote Sensing	4.32	Ma et al.[223]
			Hong Kong	Monitoring	2.9	Fung and Jim[224]
		Grass (other)	Sydney, Australia	Monitoring	2.94	Adams and Smith[225]
		Shrubland (other)	Howard Valley, South Island, New Zealand	Monitoring	3	Callard et al.[226]
Mixed (Green-Blue)		Mixed (Green-Blue)	Olympic Forest Park, Beijing, China	Monitoring	0.4	Amani-Beni et al.[227]
			Beijing, China	Remote Sensing	1.32	Liu et al.[228]
			Nagpur, Maharashtra	Remote Sensing	3.6	Jain et al.[229]
			Igapó Lak, Latin American city	Monitoring	2.63	Targino et al.[230]
			Beijing, China	Monitoring & Modelling	0.4	Cheung and Jim[231]
			Olympic Area, Beijing, China	Remote Sensing	4.95	Dai et al.[232]

Table S7. The average performance of different types of GBGI categories, which were evaluated using in-situ, modelling, in-situ combined modelling, and remote sensing techniques for heat risk adaptation and mitigations. The '-' symbol indicates 'no data available'.

GBGI	GBGI Category	Monitoring ΔT (°C)	Modelling ΔT (°C)	RS ΔT (°C)	MM ΔT (°C)	Overall ΔT (°C)	Availability
Gardens	Balcony	2.0	6.1	-	-	4.06	Medium
	Private garden	2.3	-	-	-	2.30	Very low
	Irrigating backyard	-	2.7	3.7	-	3.20	Very low
Parks	Pocket Park	1.4	2.9	4.1	0.28	2.16	Medium
	Park	3.0	0.8	-	2.38	2.07	Very High
	Botanical garden	5.6	-	-	2.2	3.90	Low
Amenity areas	Sports field	-	-	-	3.1	3.10	Very low
	Playground	3.0	-	2.8	-	2.90	Very low
	Golf course	-	-	5.0	-	5.00	Very low
	Shared open space	-	2.1	-	-	2.10	Very low
Other public space	Cemetery	1.7	-	-	-	1.70	Very low
	Allotment	-	-	4.0	-	4.00	Very low
	City farm	3.9	3.0	-	-	3.45	Very low
	Adopted public space	-	2.3	-	3	2.63	Very low
Linear GI features/ routes	Street tree	2.8	4.3	7.6	1.45	4.05	Very High
	Road verge	2.1	2.0	-	0.05	1.39	Low

	Riparian woodland	2.1	-	5.4	-	3.76	Low
	Hedge	-	2.9	7.0	1.29	3.73	Low
Constructed GI on infrastructure	Green roof	3.9	0.5	0.9	0.4	1.43	Very High
	Green wall	4.7	1.5	12.6	2	5.21	Very High
	Roof garden	2.1	0.3	7.1	-	3.13	Medium
	Pergola	1.7	-	18.2	2.65	7.50	Low
Hybrid GI (for water)	Permeable paving	3.8	0.6	-	-	2.22	Low
	Attenuation pond	7.0	-	-	-	7.00	Very low
	Rain garden	6.1	1.3	-	-	3.70	Very low
Waterbodies	Wetland	3.1	2.3	6.7	-	4.05	High
	Lake	3.1	0.8	2.8	-	2.22	Low
	Reservoir	2.9	-	-	-	2.94	Very low
	Sea (incl. coast)	1.4	0.4	-	1.7	1.17	Low
Other non-sealed urban areas	Woodland (other)	3.1	-	4.3	1.28	2.89	Medium
	Grass (other)	2.9	-	-	-	2.94	Very low
	Shrubland (other)	3.0	-	-	-	3.00	Very low
Mixed (Green-Blue)	Mixed (Green-Blue)	1.5	-	3.3	0.4	1.74	Low

Table S8. The projected influence of future climate change on the choice of GBGI in various climate zones.

Climate zone	Previous/Current climate	Future climate	Present GBGI	Future GBGI
Continent	Dfa	Dfb	Street trees<br>Permeable paving	Wetland
	Dfb	BSk	Botanical garden	Green wall<br>Street trees
Dry	BSk	BSh	Balcony	Green wall, street trees
	BSk	BWk	Wetland	Woodland
Temperate (Europe)	Cfb	Cfa	Green roofs,<br>Green walls,<br>Woodland<br>Reservoir<br>City farm<br>Riparian woodland	Parks<br>Pocket parks<br>Green walls<br>Green roofs<br>Lakes<br>Grass
	Dfb	Cfa	Green roof<br>Balcony<br>Road verge<br>Playground	Parks<br>Pocket parks<br>Green walls<br>Green roofs<br>Lakes<br>Grass
Temperate (China)	Cwa	Am	Park<br>Green wall<br>Green roof<br>Rain garden	Lakes<br>Road verge
	Dwa	Cwa	Woodland<br>Lake<br>Green roof<br>Road verge<br>Pergola<br>Roof garden	Green roof<br>Adopted space<br>wetland

Table 9. Summary of key stages and action points for implementing, replicating, and upscaling GBGI to mitigate urban heat.

Stages	Action points
Stakeholder engagement[a]	● Engage stakeholders early on, and from various sectors such as urban planning, public health, environmental agencies, and community organisations to identify and frame the heat risk problem and understand their concerns and priorities.<br>● Foster collaboration and participatory decision-making processes to ensure diverse perspectives are considered.<br>● Conduct workshops, interviews, and surveys to gather input and feedback from stakeholders.<br>● Involve residents, local businesses, and community groups to increase awareness and support.
Feasibility study of GBGI[b]	● Conduct a preliminary cost-benefit analysis to assess the feasibility and potential effectiveness of different GBGI measures.<br>● Consider factors such as implementation costs, maintenance requirements, technical feasibility, and expected benefits in terms of heat reduction and other co-benefits.<br>● Identify suitable locations for implementation based on the analysis of UHI intensity and vulnerability maps/zones.<br>● Explore funding options and potential partnerships to support implementation.
Assess co-benefits and dis-benefits of the GBGI[c]	● Consider the multiple co-benefits associated with GBGI, such as improved air quality, reduced stormwater runoff, enhanced biodiversity, and increased recreational opportunities.<br>● Assess potential dis-benefits, such as increased maintenance requirements, potential conflicts with existing infrastructure, allergic reactions, and displacement of vulnerable populations due to gentrification.<br>● Conduct a comprehensive cost-benefit analysis to evaluate the overall value and trade-offs of implementing GBGI.

Design GBGI measures[d]	● Select suitable GBGI measures based on the local context, including the climate, topography, available space, and community preferences.<br>● Incorporate GBGI elements such as trees, green roofs, green walls, and permeable surfaces to maximise shade, evapotranspiration, and cooling effects.<br>● Consider the use of native and drought-tolerant plant species for long-term sustainability and reduced water demand.<br>● Ensure proper placement and spacing of vegetation to optimise shading and air movement.
Policy and planning[e]	● Integrate GBGI strategies into urban planning and policy frameworks, such as comprehensive plans, zoning ordinances, and building codes.<br>● Develop heat mitigation plans that prioritise the implementation of GBGI measures in high-risk areas.<br>● Provide incentives, regulations, and guidelines to encourage the adoption of GBGI in private and public developments.<br>● Collaborate with relevant organisations to ensure coordination and alignment of policies, goals, and levelling up of sustainability agenda (e.g., SDGs, European Green Deal, Paris Climate Agreement).
Implementation[f]	● Establish partnerships and collaborations between governmental agencies, private sector organisations, and community groups for effective implementation.<br>● Allocate sufficient resources, including funding, staff, and technical expertise, for the installation and maintenance of the selected GBGI measures.<br>● Ensure proper construction practices and quality control to maximise the performance and longevity of implemented measures.<br>● Incorporate community engagement and education programs to foster stewardship and long-term support for the solutions in place.

Monitoring[g]	● Utilise relevant or a combination of in-situ measurements, remote sensing, and modelling methods to monitor the performance and effectiveness of GBGI used against heatwaves.<br>● Deploy and use weather stations, sensors, and satellite imagery to evaluate the efficacy of the GBGI measures.<br>● Collect data on temperature, humidity, air quality, and vegetation health to evaluate the impact of implemented measures.<br>● Employ modelling tools to simulate the cooling effects and assess potential future scenarios.
Evaluation[h]	● Conduct a comprehensive evaluation of the implemented GBGI measures to assess their effectiveness and cost-effectiveness.<br>● Compare the heat risk before and after implementation using temperature data, health indicators, and energy consumption.<br>● Analyse the economic, social, and environmental benefits achieved through the implementation of GBGI.<br>● Incorporate feedback from stakeholders and learn from the implementation process to inform future improvements.
Upscaling and replication[i]	● Develop strategies for upscaling and replicating successful GBGI measures in different neighbourhoods and cities.<br>● Share successful case studies and best practices to encourage replication in other areas and facilitate upscaling of GBGI measures.<br>● Adapt the GBGI approach to suit local contexts, considering factors like climate, social dynamics, and available resources.<br>● Develop training programs and capacity-building initiatives to support the replication and upscaling of GBGI measures.<br>● Foster knowledge exchange among cities and regions.

[a]Sherman and Ford[233]; O'Brien et al.[234]; [b]Coutts et al.[235]; [c]Curt et al.[236]; Ommer et al.[237]; [d]Dumitru et al.[238]; Kumar et al.[239]; [e]Davies et al.[240]; [e]European Green Deal[241]; [f]Di Pirro et al.[242]; Topal et al.[243]; [g]Augusto et al.[244]; [h]Frantzeskaki[245]; [i]Cortinovis et al.[246].

References

1. Mell, I., Allin, S., Reimer, M., & Wilker, J. (2017). Strategic green infrastructure planning in Germany and the UK: a transnational evaluation of the evolution of urban greening policy and practice. International Planning Studies 22, 333–349.
2. Wright, H. (2011). Understanding green infrastructure: the development of a contested concept in England. Local Environment 16, 1003-1019.
3. Byrne, J.A., Lo, A.Y., & Jianjun, Y. (2015). Residents' understanding of the role of green infrastructure for climate change adaptation in Hangzhou, China. Landscape and urban planning 138, 132-143.
4. Jones, L., Anderson, S., Læssøe, J., Banzhaf, E., Jensen, A., Bird, D.N., Miller, J., Hutchins, M.G., Yang, J., Garrett, J., & Taylor, T. (2022). A typology for urban green infrastructure to guide multifunctional planning of nature-based solutions. Nature-Based Solutions 2, 100041.
5. Debele, S.E., Kumar, P., Sahani, J., Marti-Cardona, B., Mickovski, S.B., Leo, L.S., Porcù, F., Bertini, F., Montesi, D., Vojinovic, Z., & Di Sabatino, S. (2019). Nature-based solutions for hydro-meteorological hazards: Revised concepts, classification schemes and databases. Environmental Research 179, 108799.
6. Cheung, P.K., Jim, C.Y., Tapper, N., Nice, K.A., & Livesley, S.J. (2022). Daytime irrigation leads to significantly cooler private backyards in summer. Urban Climate 46, 101310.
7. Castellar, J.A., Popartan, L.A., Pueyo-Ros, J., Atanasova, N., Langergraber, G., Säumel, I., Corominas, L., Comas, J., & Acuna, V. (2021). Nature-based solutions in the urban context: Terminology, classification and scoring for urban challenges and ecosystem services. Science of the Total Environment 779, 146237.
8. Demuzere, M., Orru, K., Heidrich, O., Olazabal, E., Geneletti, D., Orru, H., Bhave, A.G., Mittal, N., Feliú, E., & Faehnle, M. (2014). Mitigating and adapting to climate change: Multi-functional and multi-scale assessment of green urban infrastructure. Journal of Environmental Management 146, 107-115.
9. Rogers, C.D.F., & Hunt, D.V.L. (2019). Realising Visions for Future Cities: An Aspirational Futures Methodology. Proceedings of the Institution of Civil Engineers – Urban Design and Planning 172, 125-140.
10. Moher, D., Liberati, A., Tetzlaff, J., & Altman, D.G. (2009). Preferred reporting items for systematic reviews and meta-analyses: the PRISMA statement. Annals of Internal Medicine 151, 264-269.
11. R Core Team (2022). R: A language and environment for statistical computing. R Foundation for Statistical Computing, Vienna, Austria. URL https://www.R-project.org/.
12. Mazziotta, M., & Pareto, A. (2022). Normalization methods for spatio-temporal analysis of environmental performance: Revisiting the Min–Max method. Environmetrics 33, e2730.
13. Cai, Y., Li, C., Ye, L., Xiao, L., Gao, X., Mo, L., Du, H., Zhou, Y., & Zhou, G. (2022). Effect of the roadside tree canopy structure and the surrounding on the daytime urban air temperature in summer. Agricultural and Forest Meteorology 316, 108850.
14. Morabito, M., Crisci, A., Guerri, G., Messeri, A., Congedo, L. & Munafò, M. (2021). Surface urban heat islands in Italian metropolitan cities: Tree cover and impervious surface influences. Science of the Total Environment 751, 142334.
15. Xi, C., Wang, D., & Cao, S.J. (2023). Impacts of trees-grass area ratio on thermal environment, energy saving, and carbon benefits. Urban Climate 47, 101393.

16. Windbourne, J., Jones, T., Garvey, S., Harrison, J., Wang, L., Li, D., Templer, P., & Hutyra, L. (2020). Tree Transpiration and Urban Temperatures: Current Understanding, Implications, and Future Research Directions. BioScience 70, 576–588.
17. Lu, J., Li, Q., Zeng, L., Chen, J., Liu, G., Li, Y., Li, W., & Huang, K. (2017). A micro-climatic study on cooling effect of an urban park in a hot and humid climate. Sustainable cities and society 32, 513-522.
18. Chibuike, E.M., Ibukun, A.O., Abbas, A., & Kunda, J.J. (2018). Assessment of green parks cooling effect on Abuja urban microclimate using geospatial techniques. Remote Sensing Applications: Society and Environment 11, 11-21.
19. Peng, J., Dan, Y., Qiao, R., Liu, Y., Dong, J., & Wu, J. (2021). How to quantify the cooling effect of urban parks? Linking maximum and accumulation perspectives. Remote Sensing of Environment 252, 112135.
20. Gunawardena, K.R., Wells, M.J., & Kershaw, T. (2017). Utilising green and bluespace to mitigate urban heat island intensity. Science of the Total Environment 584, 1040-1055.
21. Lynn, B.H. & Lynn, I.M. (2020). The impact of cool and green roofs on summertime temperatures in the cities of Jerusalem and Tel Aviv. Science of the Total Environment 743, 140568.
22. Iaria, J., & Susca, T. (2022). Analytic Hierarchy Processes (AHP) evaluation of green roof-and green wall-based UHI mitigation strategies via ENVI-met simulations. Urban Climate 46, 101293.
23. Li, H., Zhao, Y., Sützl, B., Kubilay, A., & Carmeliet, J. (2022). Impact of green walls on ventilation and heat removal from street canyons: Coupling of thermal and aerodynamic resistance. Building and Environment 214, 108945.
24. Feitosa, R.C., & Wilkinson, S.J. (2020). Small-scale experiments of seasonal heat stress attenuation through a combination of green roof and green walls. Journal of Cleaner Production 250, 119443.
25. Sun, R., Chen, A., Chen, L., & Lü, Y. (2012). Cooling effects of wetlands in an urban region: The case of Beijing. Ecological Indicators 20, 57-64.
26. Zhang, Z., Chen, F., Barlage, M., Bortolotti, L.E., Famiglietti, J., Li, Z., Ma, X., & Li, Y. (2022). Cooling Effects Revealed by Modeling of Wetlands and Land-Atmosphere Interactions. Water Resources Research 58, e2021WR030573.
27. Ampatzidis, P, & Kershaw, T. (2020). A review of the impact of blue space on the urban microclimate. Science of the Total Environment 730, 139068.
28. Hathway, E.A., & Sharples, S. (2012). The interaction of rivers and urban form in mitigating the Urban Heat Island effect: A UK case study. Building and Environment 58, 14-22.
29. Wu, C., Li, J., Wang, C., Song, C., Chen, Y., Finka, M., & La Rosa, D. (2019). Understanding the relationship between urban blue infrastructure and land surface temperature. Science of the Total Environment 694, 133742.
30. Wu, S., Yang, H., Luo, P., Luo, C., Li, H., Liu, M., Ruan, Y., Zhang, S., Xiang, P., Jia, H., & Cheng, Y. (2021). The effects of the cooling efficiency of urban wetlands in an inland megacity: A case study of Chengdu, Southwest China. Building and Environment 204, 108128.
31. Teichmann, F., Horvath, A., Luisser, M., & Korjenic, A. (2022). The Impact of Small-Scale Greening on the Local Microclimate—A Case Study at Two School Buildings in Vienna. Sustainability 14, 13089.

32. Toe, D. H. C., & Kubota, T. (2015). Comparative assessment of vernacular passive cooling techniques for improving indoor thermal comfort of modern terraced houses in hot–humid climate of Malaysia. Solar Energy 114, 229–258.
33. Aghasizadeh, S., Kari, B. M., & Fayaz, R. (2022). Thermal performance of balcony thermal bridge solutions in reinforced concrete and steel frame structures. Journal of Building Engineering 48, 103984.
34. Cui, Y., & Zheng, H.-C. (2016). Impact of Three-Dimensional Greening of Buildings in Cold Regions in China on Urban Cooling Effect. Procedia Engineering 169, 297–302.
35. Grudzińska, M. (2021). Overheating assessment in flats with glazed balconies in warm-summer humid continental climate. Building Services Engineering Research and Technology 42, 583–602.
36. Hilliaho, K., Köliö, A., Pakkala, T., Lahdensivu, J., Vinha, J. (2016). Effects of added glazing on Balcony indoor temperatures: Field measurements. Energy and Buildings 128, 458-472.
37. Grudzińska, M., 2021. Overheating assessment in flats with glazed balconies in warm-summer humid continental climate. Building Services Engineering Research and Technology 42, 583-602.
38. Cheung, P. K., Jim, C. Y., Tapper, N., Nice, K. A., & Livesley, S. J. (2022). Daytime irrigation leads to significantly cooler private backyards in summer. Urban Climate 46, 101310.
39. Gao, K., Santamouris, M., & Feng, J. (2020). On the cooling potential of irrigation to mitigate urban heat island. Science of the Total Environment 740, 139754.
40. Broadbent, A. M., Coutts, A. M., Tapper, N. J., & Demuzere, M. (2018). The cooling effect of irrigation on urban microclimate during heatwave conditions. Urban Climate 23, 309–329.
41. Wang, C., Wang, Z.-H., & Yang, J. (2019). Urban water capacity: Irrigation for heat mitigation. Computers, Environment and Urban Systems 78, 101397.
42. Lau, S. S., Lin, P., & Qin, H. (2012). A preliminary study on environmental performances of pocket parks in high-rise and high-density urban context in Hong Kong. International Journal of Low-Carbon Technologies 7, 215–225.
43. Lin, P., Lau, S. S. Y., Qin, H., & Gou, Z. (2017). Effects of urban planning indicators on urban heat island: a case study of pocket parks in high-rise high-density environment. Landscape and Urban Planning 168, 48–60.
44. Rosso, F., Pioppi, B., & Pisello, A. L. (2022). Pocket parks for human-centered urban climate change resilience: Microclimate field tests and multi-domain comfort analysis through portable sensing techniques and citizens' science. Energy and Buildings 260, 111918.
45. Trájer, A. J., Sebestyén, V., Domokos, E., & Abonyi, J. (2022). Indicators for climate change-driven urban health impact assessment. Journal of Environmental Management 323, 116165.
46. Hou, J., Wang, Y., Zhou, D., & Gao, Z. (2022). Environmental Effects from Pocket Park Design According to District Planning Patterns—Cases from Xi'an, China. Atmosphere 13, 300.
47. Ma, D., Wang, Y., Zhou, D., & Zhu, Z. (2023). Cooling effect of the pocket park in the built-up block of a city: a case study in Xi'an, China. Environmental Science and Pollution Research 30, 23135–23154.

48. Wu, C., Li, J., Wang, C., Song, C., Haase, D., Breuste, J., & Finka, M. (2021). Estimating the Cooling Effect of Pocket Green Space in High Density Urban Areas in Shanghai, China. Frontiers in Environmental Science 9, 657969.
49. Ibrahim, M. S. (2021). Mitigation strategies of the urban heat island over Greater Cairo Metropolitan Area, Egypt utilizing ENVI-met model. Catrina: The International Journal of Environmental Sciences 24, 35-47.
50. Huang, J., Hao, T., Wang, Y., & Jones, P. (2022). A street-scale simulation model for the cooling performance of urban greenery: Evidence from a high-density city. Sustainable Cities and Society 82, 103908.
51. Lu, J., Li, Q., Zeng, L., Chen, J., Liu, G., Li, Y., Li, W., & Huang, K. (2017). A micro-climatic study on cooling effect of an urban park in a hot and humid climate. Sustainable Cities and Society 32, 513–522.
52. Chibuike, E. M., Ibukun, A. O., Abbas, A., & Kunda, J. J. (2018). Assessment of green parks cooling effect on Abuja urban microclimate using geospatial techniques. Remote Sensing Applications: Society and Environment 11, 11–21.
53. Zhang, Z., Lv, Y., & Pan, H. (2013). Cooling and humidifying effect of plant communities in subtropical urban parks. Urban Forestry & Urban Greening 12, 323–329.
54. Shi, M., Chen, M., Jia, W., Du, C., & Wang, Y. (2023). Cooling effect and cooling accessibility of urban parks during hot summers in China's largest sustainability experiment. Sustainable Cities and Society 93, 104519.
55. Du, C., Jia, W., Chen, M., Yan, L., & Wang, K. (2022). How can urban parks be planned to maximize cooling effect in hot extremes? Linking maximum and accumulative perspectives. Journal of Environmental Management 317, 115346.
56. Peng, J., Dan, Y., Qiao, R., Liu, Y., Dong, J., & Wu, J. (2021). How to quantify the cooling effect of urban parks? Linking maximum and accumulation perspectives. Remote Sensing of Environment 252, 112135.
57. Yang, A.-S., Juan, Y.-H., Wen, C.-Y., & Chang, C.-J. (2017). Numerical simulation of cooling effect of vegetation enhancement in a subtropical urban park. Applied Energy 192, 178–200.
58. Chen, M., Jia, W., Yan, L., Du, C., & Wang, K. (2022). Quantification and mapping cooling effect and its accessibility of urban parks in an extreme heat event in a megacity. Journal of Cleaner Production 334, 130252.
59. Das, M., Das, A., & Momin, S. (2022). Quantifying the cooling effect of urban green space: A case from urban parks in a tropical mega metropolitan area (India). Sustainable Cities and Society 87, 104062.
60. Zhou, Y., Zhao, H., Mao, S., Zhang, G., Jin, Y., Luo, Y., Huo, W., Pan, Z., An, P., & Lun, F. (2022). Studies on urban park cooling effects and their driving factors in China: Considering 276 cities under different climate zones. Building and Environment 222, 109441.
61. Algretawee, H. (2022). The effect of graduated urban park size on park cooling island and distance relative to land surface temperature (LST). Urban Climate 45, 101255.
62. Qiu, K., & Jia, B. (2020). The roles of landscape both inside the park and the surroundings in park cooling effect. Sustainable Cities and Society 52, 101864.
63. Li, Y., Fan, S., Li, K., Zhang, Y., Kong, L., Xie, Y., & Dong, L. (2021). Large urban parks summertime cool and wet island intensity and its influencing factors in Beijing, China. Urban Forestry and Urban Greening 65.

64. Gao, Z., Zaitchik, B. F., Hou, Y., & Chen, W. (2022). Toward park design optimization to mitigate the urban heat Island: Assessment of the cooling effect in five U.S. cities. Sustainable Cities and Society 81, 103870.
65. Yang, A.S., Juan, Y.H., Wen, C.Y., Chang, C.J. (2017). Numerical simulation of cooling effect of vegetation enhancement in a subtropical urban park. Applied energy 192, 178-200.
66. Şimşek, Ç. K., Serter, G., & Ödül, H. (2022). A Study on the Cooling Capacities of Urban Parks and Their Interactions with the Surrounding Urban Patterns. Applied Spatial Analysis and Policy 15, 1287–1317.
67. Li, Y., Fan, S., Li, K., Zhang, Y., & Dong, L. (2021). Microclimate in an urban park and its influencing factors: a case study of Tiantan Park in Beijing, China. Urban Ecosystems 24, 767-778.
68. Cheung, P. K., Jim, C. Y., & Siu, C. T. (2021). Effects of urban park design features on summer air temperature and humidity in compact-city milieu. Applied Geography 129, 102439.
69. Algretawee, H., Rayburg, S., & Neave, M. (2019). Estimating the effect of park proximity to the central of Melbourne city on Urban Heat Island (UHI) relative to Land Surface Temperature (LST). Ecological Engineering 138, 374–390.
70. Yan, H., Wu, F., & Dong, L. (2018). Influence of a large urban park on the local urban thermal environment. Science of the Total Environment 662, 882–891.
71. Chang, N., & Li, D. W. (2016). A study of the temperature-humidity effect and luminous environment design for urban green space. Chemical Engineering Transactions 51, 103–108.
72. Irmak, M. A., Yilmaz, S., & Dursun, D. (2017). Effect of different pavements on human thermal comfort conditions. Atmósfera 30, 355–366.
73. Su, X. (2018). Study on Humidification and Cooling Effect of Garden Plants. 4th International Conference on Education, Management and Information Technology (ICEMIT 2018).
74. Yilmaz, S., Mutlu, E., & Yilmaz, H. (2018). Alternative scenarios for ecological urbanizations using ENVI-met model. Environmental Science and Pollution Research 25, 26307–26321.
75. Yilmaz, S., Irmak, M. A., & Qaid, A. (2022). Assessing the effects of different urban landscapes and built environment patterns on thermal comfort and air pollution in Erzurum city, Turkey. Building and Environment 219, 109210.
76. Ghani, S., ElBialy, E. A., Bakochristou, F., Gamaledin, S. M. A., Rashwan, M. M., & Hughes, B. (2017). Thermal performance of stadium's Field of Play in hot climates. Energy and Buildings 139, 702–718.
77. Kuchcik, M., Dudek, W., Błażejczyk, K., Milewski, P., & Błażejczyk, A. (2016). Two faces to the greenery on housing estates–mitigating climate but aggravating allergy. A Warsaw case study. Urban Forestry & Urban Greening 16, 170–181.
78. Vanos, J. K., Middel, A., McKercher, G. R., Kuras, E. R., & Ruddell, B. L. (2016). Hot playgrounds and children's health: A multiscale analysis of surface temperatures in Arizona, USA. Landscape and Urban Planning 146, 29–42.
79. Nguyen, T. T., Eslick, H., Barber, P., Harper, R., & Dell, B. (2022). Cooling effects of urban vegetation: The role of golf courses. Remote Sensing 14, 4351.
80. Zölch, T., Maderspacher, J., Wamsler, C., & Pauleit, S. (2016). Using green infrastructure for urban climate-proofing: An evaluation of heat mitigation measures at the micro-scale. Urban Forestry & Urban Greening 20, 305–316.

81. Sallay, Á., Mikházi, Z., Gecséné Tar, I., & Takács, K. (2022). Cemeteries as a Part of Green Infrastructure and Tourism. Sustainability 14, 2918.
82. Rost, A. T., Liste, V., Seidel, C., Matscheroth, L., Otto, M., Meier, F., & Fenner, D. (2020). How cool are allotment gardens? A case study of nocturnal air temperature differences in Berlin, Germany. Atmosphere 11, 500.
83. Hawkins, T. W., Brazel, A. J., Stefanov, W. L., Bigler, W., & Saffell, E. M. (2004). The role of rural variability in urban heat island determination for Phoenix, Arizona. Journal of Applied Meteorology and Climatology 43, 476-486.
84. Masson, V., Lion, Y., Peter, A., Pigeon, G., Buyck, J., & Brun, E. (2013). "Grand Paris": regional landscape change to adapt city to climate warming. Climatic Change 117, 769-782.
85. Basu, T., & Das, A. (2023). Urbanization induced degradation of urban green space and its association to the land surface temperature in a medium-class city in India. Sustainable Cities and Society 90, 104373.
86. Boeri, A. N. D. R. E. A., Gaspari, J. A. C. O. P. O., Gianfrate, V. A. L. E. N. T. I. N. A., & Longo, D. A. N. I. L. A. (2017). Accelerating urban transition: An approach to greening the built environment. WIT Transactions on Ecology and the Environment 223, 3-14.
87. Abdollahzadeh, N., & Biloria, N. (2021). Outdoor thermal comfort: Analyzing the impact of urban configurations on the thermal performance of street canyons in the humid subtropical climate of Sydney. Frontiers of Architectural Research 10, 394–409.
88. Cai, Y., Li, C., Ye, L., Xiao, L., Gao, X., Mo, L., Du, H., Zhou, Y., & Zhou, G. (2022). Effect of the roadside tree canopy structure and the surrounding on the daytime urban air temperature in summer. Agricultural and Forest Meteorology 316, 108850.
89. Morabito, M., Crisci, A., Guerri, G., Messeri, A., Congedo, L., & Munafò, M. (2021). Surface urban heat islands in Italian metropolitan cities: Tree cover and impervious surface influences. Science of The Total Environment 751, 142334.
90. Napoli, M., Massetti, L., Brandani, G., Petralli, M., & Orlandini, S. (2016). Modeling Tree Shade Effect on Urban Ground Surface Temperature. Journal of Environmental Quality 45, 146–156.
91. Xi, C., Wang, D., & Cao, S.-J. (2023). Impacts of trees-grass area ratio on thermal environment, energy saving, and carbon benefits. Urban Climate 47, 101393.
92. Zeeshan, M., Ali, Z., Sajid, M., Ali, M., & Usman, M. (2022). Modelling the cooling effectiveness of street trees with actual canopy drag and real transpiration rate under representative climatic conditions. Journal of Building Performance Simulation, 1–14.
93. Abu Ali, M., Alawadi, K., & Khanal, A. (2021). The role of green infrastructure in enhancing microclimate conditions: A case study of a low-rise neighborhood in Abu Dhabi. Sustainability 13, 4260.
94. Marando, F., Salvatori, E., Sebastiani, A., Fusaro, L., & Manes, F. (2019). Regulating Ecosystem Services and Green Infrastructure: assessment of Urban Heat Island effect mitigation in the municipality of Rome, Italy. Ecological Modelling 392, 92–102.
95. Zeeshan, M., Ali, Z., Ud Din, E. (2023). Thermal performance prediction of street trees inside isolated open spaces–evaluations from real scale retrofitting project. Journal of Building Performance Simulation 16, 381-397.
96. Wang, Y., & Akbari, H. (2016). The effects of street tree planting on Urban Heat Island mitigation in Montreal. Sustainable Cities and Society 27, 122–128.

97. Lachapelle, J. A., Scott Krayenhoff, E., Middel, A., Coseo, P., & Warland, J. (2023). Maximizing the pedestrian radiative cooling benefit per street tree. Landscape and Urban Planning 230, 104608.
98. Segura, R., Krayenhoff, E. S., Martilli, A., Badia, A., Estruch, C., Ventura, S., & Villalba, G. (2022). How do street trees affect urban temperatures and radiation exchange? Observations and numerical evaluation in a highly compact city. Urban Climate 46, 101288.
99. Miao, C., Li, P., Huang, Y., Sun, Y., Chen, W., & Yu, S. (2023). Coupling outdoor air quality with thermal comfort in the presence of street trees: a pilot investigation in Shenyang, Northeast China. Journal of Forestry Research 34, 831-839.
100. Geletič, J., Lehnert, M., Resler, J., Krč, P., Middel, A., Krayenhoff, E. S., & Krüger, E. (2022). High-fidelity simulation of the effects of street trees, green roofs and green walls on the distribution of thermal exposure in Prague-Dejvice. Building and Environment 223, 109484.
101. Mussetti, G., Brunner, D., Henne, S., Allegrini, J., Scott Krayenhoff, E., Schubert, S., Feigenwinter, C., Vogt, R., Wicki, A., & Carmeliet, J. (2020). COSMO-BEP-Tree v1.0: A coupled urban climate model with explicit representation of street trees. Geoscientific Model Development 13, 1685–1710.
102. Vailshery, L. S., Jaganmohan, M., & Nagendra, H. (2013). Effect of street trees on microclimate and air pollution in a tropical city. Urban Forestry & Urban Greening 12, 408–415.
103. Gillner, S., Vogt, J., Tharang, A., Dettmann, S., & Roloff, A. (2015). Role of street trees in mitigating effects of heat and drought at highly sealed urban sites. Landscape and Urban Planning 143, 33–42.
104. Coutts, A. M., White, E. C., Tapper, N. J., Beringer, J., & Livesley, S. J. (2016). Temperature and human thermal comfort effects of street trees across three contrasting street canyon environments. Theoretical and Applied Climatology 124, 55–68.
105. Sanusi, R., Johnstone, D., May, P., & Livesley, S. J. (2016). Street Orientation and Side of the Street Greatly Influence the Microclimatic Benefits Street Trees Can Provide in Summer. Journal of Environmental Quality 45, 167–174.
106. Aminipouri, M., Knudby, A. J., Krayenhoff, E. S., Zickfeld, K., & Middel, A. (2019). Modelling the impact of increased street tree cover on mean radiant temperature across Vancouver's local climate zones. Urban Forestry & Urban Greening 39, 9–17.
107. Kusaka, H., Nakamura, Y., & Asano, Y. (2022). UV Parasol, Dry-Mist Spraying, and Street Trees as Tools for Heat Stress Mitigation. Journal of the Meteorological Society of Japan. Ser. II 100, 677–685.
108. Cho, H. (2022). Effects of Road Components and Roadside Vegetation on Temperature Reduction in Seoul Considering Air, Wet-Bulb Globe, and Surface Temperatures. Sustainability 14, 16663.
109. Huang, K. T., & Li, Y. J. (2017). Impact of street canyon typology on building's peak cooling energy demand: A parametric analysis using orthogonal experiment. Energy and Buildings 154, 448-464.
110. Stojanovic, N., Vasiljevic, N., Mešicek, M., & Lisica, A. (2018). The influence of roadside green spaces on thermal conditions in the urban environment. Journal of Architectural and Planning Research 35, 165-178.
111. Zaki, S. A., Toh, H. J., Yakub, F., Mohd Saudi, A. S., Ardila-Rey, J. A., & Muhammad-Sukki, F. (2020). Effects of roadside trees and road orientation on thermal environment in a tropical city. Sustainability 12, 1053.

112. Zheng, X., Chen, L., & Yang, J. (2023). Simulation framework for early design guidance of urban streets to improve outdoor thermal comfort and building energy efficiency in summer. Building and Environment 228, 109815.
113. Žižlavská, N., Mikita, T., & Patočka, Z. (2021). The Effects of Roadside Woody Vegetation on the Surface Temperature of Cycle Paths. Land 10, 483.
114. Adams, M. P., & Smith, P. L. (2014). A systematic approach to model the influence of the type and density of vegetation cover on urban heat using remote sensing. Landscape and Urban Planning 132, 47-54.
115. Yonghong, S., Fandi, L., Gaofeng, Z., Zhang, K., & Qi, Z. (2023). The biophysical climate mitigation potential of riparian forest ecosystems in arid Northwest China. Science of The Total Environment 862, 160856.
116. Tsai, C. W., Young, T., Warren, P. H., & Maltby, L. (2017). Riparian thermal conditions across a mixed rural and urban landscape. Applied geography 87, 106-114.
117. Dugdale, S.J., Malcolm, I.A., Hannah, D.M. (2019). Drone-based Structure-from-Motion provides accurate forest canopy data to assess shading effects in river temperature models. Science of the Total Environment 678, 326-340.
118. Zheng, X., Zhang, N., & Wang, X. (2022). Development of a modified thermal humidity index and its application to human thermal comfort of urban vegetation patches. Ecosystem Health and Sustainability 8, 2130095.
119. Zhang, J., & Hu, D. (2022). Research of practical heat mitigation strategies in a residential district of Beijing, North China. Urban Climate 46, 101314.
120. Peluso, P., Persichetti, G., & Moretti, L. (2022). Effectiveness of Road Cool Pavements, Greenery, and Canopies to Reduce the Urban Heat Island Effects. Sustainability 14, 16027.
121. Del Serrone, G., Peluso, P., & Moretti, L. (2022). Evaluation of Microclimate Benefits Due to Cool Pavements and Green Infrastructures on Urban Heat Islands. Atmosphere 13, 1586.
122. Zou, Z., Yang, Y., Qiu, G. Y. (2019). Quantifying the evapotranspiration rate and its cooling effects of urban hedges based on three-temperature model and infrared remote sensing. Remote Sensing 11, 202.
123. Yoshida, A., Hayashi, D., Shimazaki, Y., & Kinoshita, S. (2019). Evaluation of thermal sensation in various outdoor radiation environments. Architectural Science Review 62, 261–270.
124. Wang, X., Li, H., & Sodoudi, S. (2022). The effectiveness of cool and green roofs in mitigating urban heat island and improving human thermal comfort. Building and Environment 217, 109082.
125. Cortes, A., Rejuso, A. J., Santos, J. A., & Blanco, A. (2022). Evaluating mitigation strategies for urban heat island in Mandaue City using ENVI-met. Journal of Urban Management 11, 97–106.
126. Fleck, R., Gill, R. L., Saadeh, S., Pettit, T., Wooster, E., Torpy, F., & Irga, P. (2022). Urban green roofs to manage rooftop microclimates: A case study from Sydney, Australia. Building and Environment 209, 108673.
127. Dong, J., Lin, M., Zuo, J., Lin, T., Liu, J., Sun, C., & Luo, J. (2020). Quantitative study on the cooling effect of green roofs in a high-density urban Area—A case study of Xiamen, China. Journal of Cleaner Production 255, 120152.
128. Kostadinović, D., Jovanović, M., Bakić, V., Stepanić, N., & Todorović, M. (2022). Experimental investigation of summer thermal performance of the green roof system with mineral wool substrate. Building and Environment 217, 109061.

129. Peng, L. L. H., Yang, X., He, Y., Hu, Z., Xu, T., Jiang, Z., & Yao, L. (2019). Thermal and energy performance of two distinct green roofs: Temporal pattern and underlying factors in a subtropical climate. Energy and Buildings 185, 247–258.
130. Lee, L. S. H., & Jim, C. Y. (2019). Urban woodland on intensive green roof improved outdoor thermal comfort in subtropical summer. International Journal of Biometeorology 63, 895–909.
131. Köhler, M., & Kaiser, D. (2019). Evidence of the climate mitigation effect of green roofs-A 20-year weather study on an Extensive Green Roof (EGR) in Northeast Germany. Buildings 9, 157.
132. Park, J., Kim, J.-H., Dvorak, B., & Lee, D. K. (2018). The role of green roofs on microclimate mitigation effect to local climates in summer. International Journal of Environmental Research 12, 671–679.
133. Lynn, B. H., & Lynn, I. M. (2020). The impact of cool and green roofs on summertime temperatures in the cities of Jerusalem and Tel Aviv. Science of The Total Environment 743, 140568.
134. Chen, X., Jeong, S., Zheng, Y., Park, H., Park, C. E., Joo, J., Choi, W., Chen, X., & Zheng, C. (2020). Evaluation of Different Roof Materials for the Mitigation of Urban Warming in a Subtropical Monsoon Climate. Journal of Geophysical Research: Atmospheres 125, e2019JD031972.
135. Cortes, A., Rejuso, A. J., Santos, J. A., & Blanco, A. (2022). Evaluating mitigation strategies for urban heat island in Mandaue City using ENVI-met. Journal of Urban Management 11, 97–106.
136. Solcerova, A., van de Ven, F., Wang, M., Rijsdijk, M., & van de Giesen, N. (2017). Do green roofs cool the air? Building and Environment 111, 249–255.
137. Bochenek, A. D., & Klemm, K. (2020). The impact of passive green technologies on the microclimate of historic urban structures: The case study of Lodz. Atmosphere 11, 974.
138. Chen, B., Wang, W., You, Y., Zhu, W., Dong, Y., Xu, Y., Chang, M., & Wang, X. (2023). Influence of rooftop mitigation strategies on the thermal environment in a subtropical city. Urban Climate 49, 101450.
139. Elnabawi, M. H., & Saber, E. (2023). A numerical study of cool and green roof strategies on indoor energy saving and outdoor cooling impact at pedestrian level in a hot arid climate. Journal of Building Performance Simulation 16, 72–89.
140. Zuo, J., Ma, J., Lin, T., Dong, J., Lin, M., & Luo, J. (2022). Quantitative valuation of green roofs' cooling effects under different urban spatial forms in high-density urban areas. Building and Environment 222, 109367.
141. Iaria, J., & Susca, T. (2022). Analytic Hierarchy Processes (AHP) evaluation of green roof- and green wall-based UHI mitigation strategies via ENVI-met simulations. Urban Climate 46, 101293.
142. Fleck, R., Gill, R. L., Saadeh, S., Pettit, T., Wooster, E., Torpy, F., & Irga, P. (2022). Urban green roofs to manage rooftop microclimates: A case study from Sydney, Australia. Building and Environment 209, 108673.
143. Robbiati, F. O., Cáceres, N., Hick, E. C., Suarez, M., Soto, S., Barea, G., Matoff, E., Galetto, L., & Imhof, L. (2022). Vegetative and thermal performance of an extensive vegetated roof located in the urban heat island of a semiarid region. Building and Environment 212, 108791.

144. Liao, J., Tan, X., & Li, J. (2021). Evaluating the vertical cooling performances of urban vegetation scenarios in a residential environment. Journal of Building Engineering 39, 102313.
145. Cameron, R. W., Taylor, J. E., & Emmett, M. R. (2014). What's ‘cool’in the world of green façades? How plant choice influences the cooling properties of green walls. Building and environment 73, 198-207.
146. Zhang, L., Deng, Z., Liang, L., Zhang, Y., Meng, Q., Wang, J., & Santamouris, M. (2019). Thermal behavior of a vertical green facade and its impact on the indoor and outdoor thermal environment. Energy and Buildings 204, 109502.
147. Liu, Y., Huang, Y., Zhang, Z., Wang, K., Luo, Y., & Cui, P. (2022). Impacts of green walls on the characteristics of thermo-flow and photochemical reaction kinetics within street canyons. Urban Forestry & Urban Greening 72, 127568.
148. Cameron, R. W., Taylor, J., & Emmett, M. (2015). A Hedera green façade–energy performance and saving under different maritime-temperate, winter weather conditions. Building and Environment 92, 111-121.
149. Jesus, M. P., Lourenço, J. M., Arce, R. M., & Macias, M. (2017). Green façades and in situ measurements of outdoor building thermal behaviour. Building and Environment 119, 11–19.
150. Lee, L. S. H, & Jim, C. Y. (2017). Subtropical summer thermal effects of wirerope climber green walls with different air-gap depths. Building and Environment 126, 1-12.
151. Feitosa, R.C., Wilkinson, S.J. (2018). Attenuating heat stress through green roof and green wall retrofit. Building and Environment 140, 11-22.
152. Li, H., Zhao, Y., Sützl, B., Kubilay, A., & Carmeliet, J. (2022). Impact of green walls on ventilation and heat removal from street canyons: Coupling of thermal and aerodynamic resistance. Building and Environment 214, 108945.
153. Geletič, J., Lehnert, M., Resler, J., Krč, P., Middel, A., Krayenhoff, E.S. and Krüger, E. (2022). High-fidelity simulation of the effects of street trees, green roofs and green walls on the distribution of thermal exposure in Prague-Dejvice. Building and Environment 223, 109484.
154. Šuklje, T., Medved, S., & Arkar, C. (2016). On detailed thermal response modeling of vertical greenery systems as cooling measure for buildings and cities in summer conditions. Energy 115, 1055-1068.
155. Medl, A., Mayr, S., Rauch, H. P., Weihs, P., & Florineth, F. (2017). Microclimatic conditions of ‘Green Walls’, a new restoration technique for steep slopes based on a steel grid construction. Ecological engineering 101, 39-45.
156. Feitosa, R. C., & Wilkinson, S. J. (2020). Small-scale experiments of seasonal heat stress attenuation through a combination of green roof and green walls. Journal of Cleaner Production 250, 119443.
157. Blanco, I., Schettini, E., & Vox, G. (2019). Predictive model of surface temperature difference between green façades and uncovered wall in Mediterranean climatic area. Applied Thermal Engineering 163, 114406.
158. Bakhshoodeh, R., Ocampo, C., & Oldham, C. (2022). Exploring the evapotranspirative cooling effect of a green façade. Sustainable Cities and Society 81, 103822.
159. Davis, M. M., Vallejo Espinosa, A. L., & Ramirez, F. R. (2019). Beyond green façades: active air-cooling vertical gardens. Smart and Sustainable Built Environment 8, 243-252.

160. Hosseinzadeh, A., Bottacin-Busolin, A., & Keshmiri, A. (2022). A parametric study on the effects of green roofs, green walls and trees on air quality, temperature and velocity. Buildings 12, 2159.
161. Djedjig, R., Bozonnet, E., & Belarbi, R. (2015). Experimental study of the urban microclimate mitigation potential of green roofs and green walls in street canyons. International Journal of Low-Carbon Technologies 10, 34-44.
162. Pragati, S., Shanthi Priya, R., Pradeepa, C., & Senthil, R. (2023). Simulation of the Energy Performance of a Building with Green Roofs and Green Walls in a Tropical Climate. Sustainability 15, 2006.
163. Lin, H., Xiao, Y., Musso, F., & Lu, Y. (2019). Green façade effects on thermal environment in transitional space: Field measurement studies and computational fluid dynamics simulations. Sustainability 11, 5691.
164. Price, A., Jones, E. C., & Jefferson, F. (2015). Vertical greenery systems as a strategy in urban heat island mitigation. Water, Air, & Soil Pollution 226, 1-11.
165. Lee, L. S. H, & Jim, C. Y. (2020). Multidimensional analysis of temporal and layered microclimatic behavior of subtropical climber green walls in summer. Urban Ecosystems 23, 389-402.
166. Lin, H., Musso, F., & Xiao, Y. (2018). Shading Effect and Heat Reflection of the Green Façade. PLEA 2018: Smart and Healthy Within the Two-Degree Limit, 931.
167. Li, J., Zheng, B., Shen, W., Xiang, Y., Chen, X., & Qi, Z. (2019). Cooling and energy-saving performance of different green wall design: A simulation study of a block. Energies 12, 2912.
168. Peng, L.L., Yang, X., He, Y., Hu, Z., Xu, T., Jiang, Z., Yao, L. (2019). Thermal and energy performance of two distinct green roofs: Temporal pattern and underlying factors in a subtropical climate. Energy and Buildings 185, 247-258.
169. Shen, Z. (2022). Green Roof Design of Residential Area Based on Sponge City Theory. Wireless Communications and Mobile Computing, 2022.
170. AbdulBaqi, F. K. (2022). The effect of afforestation and green roofs techniques on thermal reduction in Duhok city. Trees, Forests and People 8, 100267.
171. Kim, J., Lee, S. Y., & Kang, J. (2020). Temperature reduction effects of rooftop garden arrangements: A case study of Seoul National University. Sustainability 12, 6032.
172. Lee, L. S. H., & Jim, C. Y. (2018). Thermal-cooling performance of subtropical green roof with deep substrate and woodland vegetation. Ecological Engineering 119, 8–18.
173. Tan, C. L., Tan, P. Y., Wong, N. H., Takasuna, H., Kudo, T., Takemasa, Y., Lim, C. V. J., & Chua, H. X. V. (2017). Impact of soil and water retention characteristics on green roof thermal performance. Energy and Buildings 152, 830–842.
174. Tan, C. L., Wong, N. H., Jusuf, S. K., & Chiam, Z. Q. (2015). Impact of plant evapotranspiration rate and shrub albedo on temperature reduction in the tropical outdoor environment. Building and Environment 94, 206–217.
175. Watanabe, S., Nagano, K., Ishii, J., & Horikoshi, T. (2014). Evaluation of outdoor thermal comfort in sunlight, building shade, and pergola shade during summer in a humid subtropical region. Building and Environment 82, 556–565.
176. Katsoulas, N., Antoniadis, D., Tsirogiannis, I. L., Labraki, E., Bartzanas, T., & Kittas, C. (2017). Microclimatic effects of planted hydroponic structures in urban environment: measurements and simulations. International Journal of Biometeorology 61, 943–956.

177. Chàfer, M., Pisello, A. L., Piselli, C., & Cabeza, L. F. (2020). Greenery system for cooling down outdoor spaces: Results of an experimental study. Sustainability 12, 5888.
178. Kong, H., Choi, N., & Park, S. (2021). Thermal environment analysis of landscape parameters of an urban park in summer - A case study in Suwon, Republic of Korea. Urban Forestry and Urban Greening 65, 127377.
179. Teichmann, F., Horvath, A., Luisser, M. and Korjenic, A. (2022). The Impact of Small-Scale Greening on the Local Microclimate—A Case Study at Two School Buildings in Vienna. Sustainability 14, 13089.
180. Fini, A., Frangi, P., Mori, J., Donzelli, D., & Ferrini, F. (2017). Nature based solutions to mitigate soil sealing in urban areas: Results from a 4-year study comparing permeable, porous, and impermeable pavements. Environmental research 156, 443-454.
181. Kousis, I., Fabiani, C., & Pisello, A. L. (2022). Could a bio-resin and transparent pavement improve the urban environment? An in field thermo-optical investigation and life-cycle assessment. Sustainable Cities and Society 79, 103597.
182. Lu, R., Jiang, W., Xiao, J., Xing, C., Ruan, C., Li, Y., & Wu, W. (2022). Temperature characteristics of permeable asphalt pavement: Field research. Construction and Building Materials 332, 127379.
183. Moretti, L., Cantisani, G., Carpiceci, M., D'Andrea, A., Del Serrone, G., Di Mascio, P., & Loprencipe, G. (2021). Effect of sampietrini pavers on urban heat islands. International journal of environmental research and public health 18, 13108.
184. Wang, J., Meng, Q., Tan, K., Zhang, L., & Zhang, Y. (2018). Experimental investigation on the influence of evaporative cooling of permeable pavements on outdoor thermal environment. Building and Environment 140, 184-193.
185. Wang, X., Wang, X., Chen, Y., & Berlyn, G. P. (2019). Photosynthetic parameters of urban greening trees growing on paved land. iForest-Biogeosciences and Forestry 12, 403.
186. Yang, W., Wang, Z., & Zhao, X. (2015). Experimental investigation of the thermal isolation and evaporative cooling effects of an exposed shallow-water-reserved roof under the sub-tropical climatic condition. Sustainable Cities and Society 14, 293-304.
187. An, K. J., Lam, Y. F., Hao, S., Morakinyo, T. E., & Furumai, H. (2015). Multi-purpose rainwater harvesting for water resource recovery and the cooling effect. Water Research 86, 116-121.
188. Buzzard, V., Gil-Loaiza, J., Grachet, N. G., Talkington, H., Youngerman, C., Tfaily, M. M., & Meredith, L. K. (2021). Green infrastructure influences soil health: biological divergence one year after installation. Science of The Total Environment 801, 149644.
189. Kasprzyk, M., Szpakowski, W., Poznańska, E., Boogaard, F. C., Bobkowska, K., & Gajewska, M. (2022). Technical solutions and benefits of introducing rain gardens–Gdańsk case study. Science of The Total Environment 835, 155487.
190. Bai, J., Lu, Q., Zhao, Q., Wang, J., & Ouyang, H. (2013). Effects of alpine wetland landscapes on regional climate on the Zoige Plateau of China. Advances in Meteorology, 2013.
191. Cai, Z., Guldmann, J. M., Tang, Y., & Han, G. (2022). Does city-water layout matter? Comparing the cooling effects of water bodies across 34 Chinese megacities. Journal of Environmental Management 324, 116263.
192. Pucher, B., Zluwa, I., Spörl, P., Pitha, U., & Langergraber, G. (2022). Evaluation of the multifunctionality of a vertical greening system using different irrigation strategies on

cooling, plant development and greywater use. Science of The Total Environment 849, 157842.

193. Ruiz-Aviles, V., Brazel, A., Davis, J. M., & Pijawka, D. (2020). Mitigation of urban heat island effects through "green infrastructure": integrated design of constructed wetlands and neighborhood development. Urban Science 4, 78.
194. Shahjahan, A. T. M., Ahmed, K. S., & Said, I. B. (2020). Study on Riparian Shading Envelope for Wetlands to Create Desirable Urban Bioclimates. Atmosphere 11, 1348.
195. Şimşek, Ç. K., & Ödül, H. (2018). Investigation of the effects of wetlands on micro-climate. Applied geography 97, 48-60.
196. Sun, R., Chen, A., Chen, L., & Lü, Y. (2012). Cooling effects of wetlands in an urban region: The case of Beijing. Ecological Indicators 20, 57-64.
197. Sušnik, J., Masia, S., Kravčík, M., Pokorný, J., & Hesslerová, P. (2022). Costs and benefits of landscape-based water retention measures as nature-based solutions to mitigating climate impacts in eastern Germany, Czech Republic, and Slovakia. Land Degradation & Development 33, 3074-3087.
198. Triyuly, W., Triyadi, S., & Wonorahardjo, S. (2021). Synergising the thermal behaviour of water bodies within thermal environment of wetland settlements. International Journal of Energy and Environmental Engineering 12, 55-68.
199. Wu, S., Yang, H., Luo, P., Luo, C., Li, H., Liu, M., Ruan, Y., Zhang, S., Xiang, P., Jia, H., & Cheng, Y. (2021). The effects of the cooling efficiency of urban wetlands in an inland megacity: A case study of Chengdu, Southwest China. Building and Environment 204, 108128.
200. Xu, H., Chen, H., Zhou, X., Wu, Y., & Liu, Y. (2020). Research on the relationship between urban morphology and air temperature based on mobile measurement: A case study in Wuhan, China. Urban Climate 34, 100671.
201. Zhang, W., Jiang, J., & Zhu, Y. (2015). Change in urban wetlands and their cold island effects in response to rapid urbanization. Chinese Geographical Science 25, 462-471.
202. Wenguang, Z., Wenjuan, W., Guanglei, H., Chao, G., Ming, J., & Xianguo, L. (2020). Cooling effects of different wetlands in semi-arid rural region of Northeast China. Theoretical and Applied Climatology 141, 31-41.
203. Zhang, Z., Chen, F., Barlage, M., Bortolotti, L. E., Famiglietti, J., Li, Z., Ma. X., & Li, Y. (2022). Cooling effects revealed by modeling of wetlands and land-atmosphere interactions. Water Resources Research 58, e2021WR030573.
204. Sun, R., Chen, A., Chen, L., Lü, Y. (2012). Cooling effects of wetlands in an urban region: The case of Beijing. Ecological Indicators 20, 57-64.
205. Le Phuc, C. L., Nguyen, H. S., Dao Dinh, C., Tran, N. B., Pham, Q. B., & Nguyen, X. C. (2022). Cooling island effect of urban lakes in hot waves under foehn and climate change. Theoretical and Applied Climatology 149, 817-830.
206. Theeuwes, N. E., Solcerova, A., & Steeneveld, G. J. (2013). Modeling the influence of open water surfaces on the summertime temperature and thermal comfort in the city. Journal of Geophysical Research: Atmospheres 118, 8881-8896.
207. Xu, H., Chen, H., Zhou, X., Wu, Y., Liu, Y. (2020). Research on the relationship between urban morphology and air temperature based on mobile measurement: A case study in Wuhan, China. Urban Climate 34, 100671.
208. Yang, S. J., Ran, G. P., Zhang, W., & Wang, Z. H. (2020). The cooling effect of an urban lake landscape on the urban heat island: a case study in Jinan, China. Applied Ecology & Environmental Research 18, 2197-2211.

209. Masiero, É., & de Souza, L. C. L. (2016). Improving urban thermal profile with trees and water features. Proceedings of the Institution of Civil Engineers-Urban Design and Planning 169, 66-77.
210. Novo, A. V., Bayon, J. R., Castro-Fresno, D., & Rodriguez-Hernandez, J. (2013). Temperature performance of different pervious pavements: rainwater harvesting for energy recovery purposes. Water resources management 27, 5003-5016.
211. Novo, A. V., Bayon, J. R., Castro-Fresno, D., & Rodriguez-Hernandez, J. (2013). Monitoring and evaluation of the thermal behavior of permeable pavements for energy recovery purposes in an experimental parking lot: Preliminary results. Journal of Energy Engineering 139, 230-237.
212. Dandou, A., Papangelis, G., Kontos, T., Santamouris, M., & Tombrou, M. (2021). On the cooling potential of urban heating mitigation technologies in a coastal temperate city. Landscape and Urban Planning 212, 104106.
213. Zhou, X., Okaze, T., Ren, C., Cai, M., Ishida, Y., Watanabe, H., & Mochida, A. (2020). Evaluation of urban heat islands using local climate zones and the influence of sea-land breeze. Sustainable Cities and Society 55, 102060.
214. Zhou, Y., Guan, H., Gharib, S., Batelaan, O., & Simmons, C. T. (2021). Cooling power of sea breezes and its inland penetration in dry-summer Adelaide, Australia. Atmospheric Research 250, 105409.
215. Zhou, Y., Guan, H., Huang, C., Fan, L., Gharib, S., Batelaan, O., & Simmons, C. (2019). Sea breeze cooling capacity and its influencing factors in a coastal city. Building and Environment 166, 106408.
216. Zhu, D., Zhou, X., & Cheng, W. (2022). Water effects on urban heat islands in summer using WRF-UCM with gridded urban canopy parameters — A case study of Wuhan. Building and Environment 225, 109528.
217. Sahani, J., P, Kumar., SE., Debele. (2023). Efficacy assessment of green-blue nature-based solutions against environmental heat mitigation. Environment International. Under review.
218. Fung, C. K., & Jim, C. Y. (2017). Assessing the cooling effects of different vegetation settings in a Hong Kong golf course. Procedia Environmental Sciences 37, 626-636.
219. Fung, C. K., & Jim, C. Y. (2019). Microclimatic resilience of subtropical woodlands and urban-forest benefits. Urban Forestry & Urban Greening 42, 100-112.
220. Yonghong, S., Fandi, L., Gaofeng, Z., Zhang, K., Qi, Z. (2023). The biophysical climate mitigation potential of riparian forest ecosystems in arid Northwest China. Science of The Total Environment 862, 160856.
221. Chang, N., Li, D.W. (2016). A study of the temperature-humidity effect and luminous environment design for urban green space. Chemical Engineering Transactions 51, 103-108.
222. Liu, W., Zhao, H., Sun, S., Xu, X., Huang, T., & Zhu, J. (2022). Green space cooling effect and contribution to mitigate heat island effect of surrounding communities in beijing metropolitan area. Frontiers in Public Health 10, 870403.
223. Ma, Y., Zhao, M., Li, J., Wang, J., & Hu, L. (2021). Cooling effect of different land cover types: a case study in Xi'an and Xianyang, China. Sustainability 13, 1099.
224. Fung, C.K., Jim, C.Y. (2017). Assessing the cooling effects of different vegetation settings in a Hong Kong golf course. Procedia Environmental Sciences 37, 626-636.
225. Adams, M.P., Smith, P.L. (2014). A systematic approach to model the influence of the type and density of vegetation cover on urban heat using remote sensing. Landscape and Urban Planning 132, 47-54.

226. Callard, S. L., Newnham, R. M., Vandergoes, M. J., Alloway, B. V., & Smith, C. (2013). The vegetation and climate during the Last Glacial cold period, northern South Island, New Zealand. Quaternary Science Reviews 74, 230-244.
227. Amani-Beni, M., Zhang, B., Xie, G. D., & Shi, Y. (2019). Impacts of urban green landscape patterns on land surface temperature: Evidence from the adjacent area of Olympic Forest Park of Beijing, China. Sustainability 11, 513.
228. Liu, W., Zhao, H., Sun, S., Xu, X., Huang, T., Zhu, J. (2022). Green space cooling effect and contribution to mitigate heat island effect of surrounding communities in beijing metropolitan area. Frontiers in Public Health 10, 870403.
229. Jain, S., Sannigrahi, S., Sen, S., Bhatt, S., Chakraborti, S., & Rahmat, S. (2020). Urban heat island intensity and its mitigation strategies in the fast-growing urban area. Journal of Urban Management 9, 54-66.
230. Targino, A. C., Coraiola, G. C., & Krecl, P. (2019). Green or blue spaces? Assessment of the effectiveness and costs to mitigate the urban heat island in a Latin American city. Theoretical and Applied Climatology 136, 971-984.
231. Cheung, P. K., & Jim, C. Y. (2019). Differential cooling effects of landscape parameters in humid-subtropical urban parks. Landscape and urban planning 192, 103651.
232. Dai, Z., Guldmann, J. M., & Hu, Y. (2019). Thermal impacts of greenery, water, and impervious structures in Beijing's Olympic area: A spatial regression approach. Ecological indicators 97, 77-88.
233. Sherman, M.H., & Ford, J. (2014). Stakeholder engagement in adaptation interventions: an evaluation of projects in developing nations. Climate Policy 14, 417-441.
234. O'Brien, R.M., Phelan, T.J., Smith, N.M., & Smits, K.M. (2021). Remediation in developing countries: A review of previously implemented projects and analysis of stakeholder participation efforts. Critical Reviews in Environmental Science and Technology 51, 1259-1280.
235. Coutts, J., White, T., Blackett, P., Rijswijk, K., Bewsell, D., Park, N., Turner, J.A., & Botha, N. (2017). Evaluating a space for co-innovation: Practical application of nine principles for co-innovation in five innovation projects. Outlook on Agriculture 46, 99-107.
236. Curt, C., Di Maiolo, P., Schleyer-Lindenmann, A., Tricot, A., Arnaud, A., Curt, T., Parès, N., & Taillandier, F. (2022). Assessing the environmental and social co-benefits and disbenefits of natural risk management measures. Heliyon 8, e12465.
237. Ommer, J., Bucchignani, E., Leo, L.S., Kalas, M., Vranić, S., Debele, S., Kumar, P., Cloke, H.L. & Di Sabatino, S. (2022). Quantifying co-benefits and disbenefits of Nature-based Solutions targeting Disaster Risk Reduction. International Journal of Disaster Risk Reduction 75, 102966.
238. Dumitru, A., Frantzeskaki, N., & Collier, M. (2020). Identifying principles for the design of robust impact evaluation frameworks for nature-based solutions in cities. Environmental Science & Policy 112, 107-116.
239. Kumar, P., Debele, S. E., Sahani, J., Rawat, N., Marti-Cardona, B., Alfieri, S. M., Basu, B., Basu, A. S., Bowyer, P., Charizopoulos, N., Gallotti, G., Jaakko, J., Leo, L. S., Loupis, M., Menenti, M., Mickovski, S. B., Mun, S.-J., Gonzalez-Ollauri, A., Pfeiffer, J., Pilla, F., Proll, J., Rutzinger, M., Santo, M. A., Sannigrahi, S., Spyrou, C., Tuomenvirta, H., & Zieher, T. (2021a). Nature-based solutions efficiency evaluation against natural hazards: Modelling methods, advantages and limitations. Science of The Total Environment 784, 147058.

240. Davies, C., Chen, W.Y., Sanesi, G., & Lafortezza, R. (2021). The European Union roadmap for implementing nature-based solutions: A review. Environmental Science & Policy 121, 49-67.
241. European Green Deal (2021). The European Green Deal and cohesion policy. Available at: https://www.europarl.europa.eu/RegData/etudes/BRIE/2021/698058/EPRS_BRI(2021)698058_EN.pdf (retrieved on 23 June 2023).
242. Di Pirro, E., Sallustio, L., Sgrigna, G., Marchetti, M., & Lasserre, B. (2022). Strengthening the implementation of national policy agenda in urban areas to face multiple environmental stressors: Italy as a case study. Environmental Science & Policy 129, 1-11.
243. Topal, H.F., Hunt, D.V.L., & Rogers, C.D.F. (2021). Exploring urban sustainability understanding and behavior: A systematic review towards a conceptual framework. Sustainability 13, 1139.
244. Augusto, B., Roebeling, P., Rafael, S., Ferreira, J., Ascenso, A., & Bodilis, C. (2020). Short and medium-to long-term impacts of nature-based solutions on urban heat. Sustainable Cities and Society 57, 102122.
245. Frantzeskaki, N. (2019). Seven lessons for planning nature-based solutions in cities. Environmental Science & Policy 93, 101-111.
246. Cortinovis, C., Olsson, P., Boke-Olén, N., & Hedlund, K. (2022). Scaling up nature-based solutions for climate-change adaptation: Potential and benefits in three European cities. Urban Forestry & Urban Greening 67, 127450.
